# Automated virtual reality therapy to treat agoraphobic avoidance and distress in patients with psychosis (gameChange): a multicentre, parallel-group, single-blind, randomised, controlled trial in England with mediation and moderation analyses

**DOI:** 10.1016/S2215-0366(22)00060-8

**Published:** 2022-05

**Authors:** Daniel Freeman, Sinéad Lambe, Thomas Kabir, Ariane Petit, Laina Rosebrock, Ly-Mee Yu, Robert Dudley, Kate Chapman, Anthony Morrison, Eileen O'Regan, Charlotte Aynsworth, Julia Jones, Elizabeth Murphy, Rosie Powling, Ushma Galal, Jenna Grabey, Aitor Rovira, Jennifer Martin, Chris Hollis, David M Clark, Felicity Waite, on behalf of the gameChange Trial Group, James Altunkaya, Humma Andleeb, Aislinn Bergin, Emily Bold, Jessica Bond, Kate Bransby-Adams, Susan Brown, Cindy Chan, Nisha Chauhan, Michael Craven, Jason Freeman, John Geddes, Andrew Goodsell, Lucy Jenner, Alex Kenny, José Leal, Joanna Mitchell, Heather Peel, Maryam Pervez, Eloise Prouten, Eva Roberts, Dan Robotham, Harry Walker, Jonathan West

**Affiliations:** a Department of Psychiatry, University of Oxford, Oxford, UK; b Oxford Primary Care Clinical Trials Unit, Nuffield Department of Primary Care Health Sciences, University of Oxford, Oxford, UK; c Department of Experimental Psychology, University of Oxford, Oxford, UK; d Oxford Health NHS Foundation Trust, Oxford, UK; e McPin Foundation, London, UK; f Cumbria, Northumberland, Tyne, and Wear NHS Foundation Trust, Newcastle upon Tyne, UK; g Population Health Sciences Institute, Newcastle University, Newcastle upon Tyne, UK; h Avon and Wiltshire Mental Health Partnership NHS Trust, Bath, UK; i Greater Manchester Mental Health Foundation Trust, Manchester, UK; j Division of Psychology and Mental Health, University of Manchester, Manchester, UK; k Nottinghamshire Healthcare NHS Foundation Trust, Nottingham, UK; l National Institute for Health Research MindTech MedTech Co-operative, Nottingham, UK; m Mental Health and Clinical Neurosciences, School of Medicine, Institute of Mental Health, University of Nottingham, Nottingham, UK; n National Institute for Health Research Nottingham Biomedical Research Centre, Mental Health & Technology Theme, Nottingham, UK

## Abstract

**Background:**

Automated delivery of psychological therapy using immersive technologies such as virtual reality (VR) might greatly increase the availability of effective help for patients. We aimed to evaluate the efficacy of an automated VR cognitive therapy (gameChange) to treat avoidance and distress in patients with psychosis, and to analyse how and in whom it might work.

**Methods:**

We did a parallel-group, single-blind, randomised, controlled trial across nine National Health Service trusts in England. Eligible patients were aged 16 years or older, with a clinical diagnosis of a schizophrenia spectrum disorder or an affective diagnosis with psychotic symptoms, and had self-reported difficulties going outside due to anxiety. Patients were randomly assigned (1:1) to either gameChange VR therapy plus usual care or usual care alone, using a permuted blocks algorithm with randomly varying block size, stratified by study site and service type. gameChange VR therapy was provided in approximately six sessions over 6 weeks. Trial assessors were masked to group allocation. Outcomes were assessed at 0, 6 (primary endpoint), and 26 weeks after randomisation. The primary outcome was avoidance of, and distress in, everyday situations, assessed using the self-reported Oxford Agoraphobic Avoidance Scale (O-AS). Outcome analyses were done in the intention-to-treat population (ie, all participants who were assigned to a study group for whom data were available). We performed planned mediation and moderation analyses to test the effects of gameChange VR therapy when added to usual care. This trial is registered with the ISRCTN registry, 17308399.

**Findings:**

Between July 25, 2019, and May 7, 2021 (with a pause in recruitment from March 16, 2020, to Sept 14, 2020, due to COVID-19 pandemic restrictions), 551 patients were assessed for eligibility and 346 were enrolled. 231 (67%) patients were men and 111 (32%) were women, 294 (85%) were White, and the mean age was 37·2 years (SD 12·5). 174 patients were randomly assigned to the gameChange VR therapy group and 172 to the usual care alone group. Compared with the usual care alone group, the gameChange VR therapy group had significant reductions in agoraphobic avoidance (O-AS adjusted mean difference –0·47, 95% CI –0·88 to –0·06; n=320; Cohen's *d* –0·18; p=0·026) and distress (–4·33, –7·78 to –0·87; n=322; –0·26; p=0·014) at 6 weeks. Reductions in threat cognitions and within-situation defence behaviours mediated treatment outcomes. The greater the severity of anxious fears and avoidance, the greater the treatment benefits. There was no significant difference in the occurrence of serious adverse events between the gameChange VR therapy group (12 events in nine patients) and the usual care alone group (eight events in seven patients; p=0·37).

**Interpretation:**

Automated VR therapy led to significant reductions in anxious avoidance of, and distress in, everyday situations compared with usual care alone. The mediation analysis indicated that the VR therapy worked in accordance with the cognitive model by reducing anxious thoughts and associated protective behaviours. The moderation analysis indicated that the VR therapy particularly benefited patients with severe agoraphobic avoidance, such as not being able to leave the home unaccompanied. gameChange VR therapy has the potential to increase the provision of effective psychological therapy for psychosis, particularly for patients who find it difficult to leave their home, visit local amenities, or use public transport.

**Funding:**

National Institute of Health Research Invention for Innovation programme, National Institute of Health Research Oxford Health Biomedical Research Centre.

**Research in context**

**Evidence before this study**

Immersive virtual reality (VR) has been tested in a small number of clinical studies in patients with psychosis. VR has been used in these studies by cognitive-behavioural therapists as a supplementary aid in treatment. Our aim was to advance the use of VR for patients with psychosis by automating the delivery of therapy. Successful automation within VR could enable the provision of psychological interventions on a large scale. We searched PubMed on Dec 4, 2021, with no date or language restrictions, using the terms (“Psychosis” OR “Psychotic” OR “Schizophrenia”) AND (“Virtual reality” OR “VR”) AND (“head mounted display” OR “HMD” OR “CAVE”) AND “Automated” AND “Treatment”. 63 papers were identified. There were no studies reporting the use of automated VR therapy for patients with psychosis. We did a second search of PubMed on the same date using the terms (“Psychosis” OR “Psychotic” OR “Schizophrenia”) AND (“agoraphobia” OR “anxious avoidance”) AND (“treatment” OR “therapy”) AND “randomised controlled”, and 42 papers were identified. There were no randomised controlled trials that had evaluated the treatment of agoraphobic avoidance and distress in patients with psychosis. However, there were two potentially informative studies. A randomised trial in 116 patients with paranoia that tested 16 sessions of VR-based cognitive behavioural therapy with a therapist did not find a significant end-of-treatment difference in social participation (assessed by time spent with other people) compared with a waiting-list control group, but did find a significant difference at 6-month follow-up. A randomised controlled trial in 20 patients with schizophrenia that tested an eight-session group-based cognitive behavioural therapy intervention indicated that the intervention was associated with potential reductions in avoidance of social phobia situations compared with a waiting-list control group.

**Added value of this study**

To our knowledge, this is the first randomised controlled trial of automated VR therapy for patients with psychosis. The VR therapy in this study, gameChange, was designed to reduce agoraphobic avoidance and distress, which had not previously been the target of intervention for patients with psychosis. This study is also the largest test of any VR therapy for a mental health condition. The simulations in gameChange VR therapy helped trial participants practise leaving the home, being in a café, shop, doctor's surgery, or pub, and getting on a bus. The situations had been identified during the treatment design stage of the project by individuals with lived experience of psychosis. Compared with usual care alone, gameChange VR therapy significantly reduced agoraphobic avoidance of, and distress in, everyday situations. The therapy was especially effective in reducing agoraphobia for patients with severe difficulties, who had moderate-to-large improvements that persisted for 6 months. The therapy worked via reduction of a wide range of threat cognitions and within-situation defence behaviours, which are key elements in the cognitive account of anxiety.

**Implications of all the available evidence**

gameChange VR therapy works for patients with psychosis who have anxiety about entering everyday situations. However, we recommend that it is best used with patients with substantial avoidance of or distress in everyday situations (eg, finding it difficult to leave the home). With suitable supervision arrangements, the therapy can be supported by peer support workers, assistant psychologists, or cognitive-behavioural therapists. The increasing availability and affordability of standalone VR headsets means that patients could keep the device at home for some time, thereby substantially increasing the amount of therapy received. Automated therapy delivered in easy-to-use standalone VR headsets, with support possible from a large proportion of the mental health workforce, means that gameChange is a therapy that could be delivered on a large scale in clinical services.

## Introduction

Providing effective psychological therapy on a large scale to patients with psychosis is a recognised challenge.^1^, ^2^ There is a limited number of therapists and there are issues of adherence and competence in the delivery of current evidence-based approaches. Internationally, clinical services are seldom organised to give therapists the time to carry out the direct active learning in real-world situations with patients that is often important for clinical improvement. Immersive virtual reality (VR)—interactive three-dimensional computer-generated worlds that produce the sensation of actually being in life-sized new environments—is a potentially powerful therapeutic tool that can overcome these barriers. Patients more readily partake and learn in simulations of anxiety-provoking situations because they know the recreations are not real. By automating delivery of therapy in VR, the reliance on trained therapists is removed.^3^ In automated delivery, techniques are implemented consistently and trial outcomes are highly likely to be replicated. Automated VR therapies are therefore scalable. In this Article, we report the evaluation of an automated VR therapy designed to help patients with psychosis re-engage with everyday situations.

Everyday situations can be anxiety-provoking for many patients with psychosis. Patients might fear, for example, negative judgements, observation, embarrassment, failure, rejection, panicking, deliberate social or physical harm from others, or being unable to cope with verbal auditory hallucinations. The result is that patients avoid everyday situations, such as walking in the street, going to a local shop, or getting on a bus, or find these activities intensely distressing. This anxiety leads to withdrawal from everyday situations, which adversely affects both mental and physical health. Many patients find it difficult even to leave their home. Lives can become restricted to locations and activities that are perceived as sufficiently safe because they can be left easily. When situations are feared or avoided because of thoughts that escape might be difficult or help might not be available in the event of developing panic-like symptoms or other incapacitating or embarrassing symptoms, this is defined as agoraphobia.^4^ We view agoraphobic avoidance of, and distress in, everyday situations as a final common pathway for a variety of fears experienced by most patients with psychosis. In a survey of 1809 patients with non-affective psychosis who were attending mental health services in the UK National Health Service (NHS), we found anxious avoidance classed as agoraphobia in 64·5% of patients.^5^

We used a user-centred design process to create a six-session automated VR cognitive therapy (gameChange) to directly target anxiety about everyday situations.^6^, ^7^ The cognitive perspective on problematic anxiety places unfounded fearful thoughts as the central cause.^8^ Importantly, the fearful thoughts persist because of the use of defence (or safety-seeking) behaviours that block the processing of disconfirmatory evidence.^9^ For example, people avoid entering feared situations or, when in them, rush to leave early, are vigilant for danger, or avoid eye contact (within-situation defences). The threat beliefs are not updated because the absence of harm is attributed to the use of defences. The treatment implication is that defences must be dropped so that the anxious cognitions can be fully evaluated in the feared situations. When this occurs in behavioural experiments, new beliefs and memories of safety are formed that counteract the old fear-based memories and thoughts.^10^ In two previous studies in patients with psychosis, therapists have successfully conducted behavioural experiments using VR simulations of anxiety-provoking situations to reduce fears.^11^, ^12^ This is the traditional use of VR in mental health, as an aid within cognitive therapy. gameChange was designed so that the therapy is embedded in the VR programme, and a virtual therapist guides the participant through simulations intended to limit the use of defences. A variety of mental health staff, including peer support workers, can then support patients to use the VR therapy, which greatly expands the size of the mental health workforce who could contribute to the provision of psychological therapy for patients with psychosis.

We aimed to determine the potential benefit of the automated VR therapy in patients with psychosis. The primary hypothesis was that, compared with usual care alone, gameChange VR cognitive therapy added to usual care would reduce agoraphobic avoidance of, and distress in, real-world situations.^13^ The secondary hypotheses were that, compared with usual care alone, gameChange VR cognitive therapy added to usual care would reduce psychiatric symptoms (eg, paranoia, depression, suicidal ideation), increase activity, and improve quality of life (after end of treatment). It was also hypothesised that treatment effects would be maintained at follow-up. Mediation and moderation were built into the trial design to test how the VR therapy might work and in whom it might work. It was hypothesised that the mediators of VR therapy would be safety beliefs, threat cognitions, and defence behaviours, and that the moderators would be the occurrence of negative auditory hallucinations in social situations, hopelessness, appearance concerns, and threat cognitions.

## Methods

### Study design and participants

We did a parallel-group, single-blind, randomised, controlled trial across nine NHS trusts in England. The trial was approved by an NHS Research Ethics Committee (NHS South Central—Oxford B Research Ethics Committee, 19/SC/0075) and was registered prospectively, and the protocol (appendix pp 2–51) was published at the start of the trial.^13^ A Lived Experience Advisory Panel (LEAP), comprising individuals from all trial sites, advised on the conduct of the trial throughout.

Research assistants sought referrals from nine NHS mental health trusts associated with the five trial sites (Bristol, Manchester, Newcastle, Nottingham, Oxford): Avon and Wiltshire Mental Health Partnership NHS Trust; Oxford Health NHS Foundation Trust; Berkshire Healthcare NHS Foundation Trust; Northamptonshire Healthcare NHS Foundation Trust; Central and North West London NHS Foundation Trust (Milton Keynes); Cumbria, Northumberland, Tyne, and Wear NHS Foundation Trust; Greater Manchester Mental Health NHS Foundation Trust; Pennine Care NHS Foundation Trust; and Nottinghamshire Healthcare NHS Foundation Trust.

Eligible patients were adults aged 16 years or older, who were attending an NHS mental health trust for the treatment of psychosis, with a clinical diagnosis of schizophrenia spectrum psychosis (ICD-10 codes F20–29) or an affective diagnosis with psychotic symptoms (F31.2, 31.5, 32.3, 33.3),^14^ had self-reported difficulties going outside the home primarily due to anxiety (for which they would like to have treatment), and were willing and able to provide informed consent for participation in the trial. Exclusion criteria were an inability to attempt an Oxford-Behavioural Assessment Task (O-BAT)^11^ at baseline for practical reasons (eg, due to not being permitted to leave a psychiatric ward); photosensitive epilepsy; substantial visual, auditory, or balance impairment; current receipt of another intensive psychological therapy; insufficient comprehension of English; currently in a forensic setting or Psychiatric Intensive Care Unit; organic syndrome; primary diagnosis of alcohol or substance use disorder or personality disorder; clinically significant learning disability; or current active suicidal intent with plans (ie, a crisis point). Written informed consent was obtained before participation.

### Randomisation and masking

Participants were randomly assigned (1:1) to gameChange VR therapy plus usual care or usual care alone. Randomisation was done by the trial coordinator in each centre using a validated online system (Sortition) developed by the University of Oxford Primary Care Clinical Trials Unit. Randomisation used a permuted blocks algorithm, with randomly varying block size, stratified by site (Bristol, Manchester, Newcastle, Nottingham, or Oxford) and service type (inpatient, early intervention, or community mental health team).

Trial assessors were masked to group allocation. If group allocation was revealed, another masked assessor replaced the unmasked assessor. Assessors were unmasked on 35 occasions (29 by 6 weeks and six by 26 weeks) and all assessments were successfully remasked.

### Procedures

gameChange is a VR application that is recommended for adults (age ≥16 years) who are anxious about everyday situations because of agoraphobic-type fears. The software is intended to reduce anxiety around other people. The software was programmed by Oxford VR. The application is a CE-marked class I active medical device (device code Z301; standalone software), in conformity with the essential requirements and provisions of the European Commission Directive 93/42/EEC (medical devices). The hardware used was an HTC Vive Pro headset (HTC Corporation, New Taipei City, Taiwan) and Dell G5 15 5590 laptop (Dell Technologies, Round Rock, TX, USA).

The therapy was designed to be delivered in approximately six sessions, each involving 30 min of VR, over 6 weeks. The prespecified minimum (protocol-adherent) dose of VR therapy was three sessions. A mental health worker (either peer support worker, assistant psychologist, or clinical psychologist) was in the room when the therapy was provided. Staff who delivered the therapy did not need to have previous experience of cognitive therapy. There was a wide range of clinical experience in the delivery team, including staff who had not been involved before in any type of therapy provision. The staff members had half a day of training in the delivery of the VR therapy, and weekly supervision during the trial. There was a written therapy manual for staff and a video showing how to set up the hardware used in the trial. A number of staff had opportunities to observe more experienced members of the team deliver the VR therapy. Staff members set up the hardware, briefly introduced the therapy concepts (including explaining fearful thoughts, defences, and the rationale for the use of VR), helped the participant put on the VR headset, and started the programme. Staff members encouraged participants to apply the learning from VR in the real world by setting homework tasks to be carried out between sessions. Completion of homework tasks was then reviewed by the staff member. Staff members were tasked with helping participants maximise the learning from the VR programme. The VR sessions were conducted in the participant's home or in an NHS clinic room.

The gameChange VR therapy aims for participants to relearn safety by testing their fear expectations around other people. The therapy is not designed as simple exposure therapy (participants are not asked to remain in situations until anxiety reduces), but instead as repeated behavioural experiments in which defences are reduced in order to create belief change (ie, to learn that they are safer than they had thought). The therapy was designed in collaboration with the LEAP team, who, for example, helped choose the scenarios to be programmed.^6^, ^7^ Participants usually stand during the VR therapy, and are able to walk a few paces in the scenarios. A virtual coach, within the VR environments, guided the participant through the therapy. The coach encouraged participants to let go of defence behaviours, and elicited feedback to tailor the progression of the therapy. When first entering the VR environment, participants entered the coach's virtual office and were guided in how to use VR (eg, the basic functions). At the beginning of the first session, the virtual coach explained the rationale behind the therapy, and the participant selected one of six VR social scenarios (café, general practice waiting room, pub, bus, opening the front door of the home onto the street, and small local shop). Each scenario comprised five levels of difficulty (based on the number and proximity of people in the social situation and the degree of social interaction) and participants worked their way through each level. Getting closer to other people and making eye contact are encouraged by the coach in many of the scenarios, and sometimes participants were the centre of attention within a situation (eg, being asked to ring the bell at the front of the bus). Therapeutic game-type tasks are included within several levels. These tasks are designed to help the participant let go of defence behaviours and thereby make new learning. For example, in the café, the participant is asked to burst bubbles blown by a child from a wand; the virtual characters all look at the participant who is required to move closer to the characters, and the participant has the opportunity to learn that they can cope even when they are close to people and the centre of attention. In a small number of the scenarios, the participant was asked to speak to a computer character (eg, respond to a barista asking whether they would like tea or coffee to drink or to call to someone that a wallet had been left on a counter) and voice recognition detected that a response had been made. The participant could choose a different scenario in each session or repeat a previous scenario (and level within the scenario). Throughout the sessions, participants responded to questions from the virtual coach by moving a virtual slider or touching a virtual ball labelled with the option that appeared at the appropriate time. A belief rating for confidence in social situations was repeated within VR at the beginning and end of each treatment session. Pictures of the gameChange VR programme are provided in the appendix (pp 243–46).

Usual care was recorded using the Client Service Receipt Inventory,^15^ and usually comprised prescription of antipsychotic medications, regular visits from a community mental health worker, and occasional outpatient appointments with a psychiatrist.

Assessments were done at 0, 6 (at end of treatment), and 26 weeks after randomisation.

### Outcomes

The primary outcome was avoidance of, and distress in, everyday situations, assessed using the Oxford Agoraphobic Avoidance Scale (O-AS).^16^ Secondary outcomes were agoraphobia measured by the Agoraphobia Mobility Inventory-Avoidance scale,^17^ suicidal ideation measured by the Columbia Suicide Severity Rating Scale,^19^ paranoia measured by the Revised Green et al Paranoid Thoughts Scale,^20^ paranoia worries measured by the Paranoia Worries Questionnaire,^21^ depression measured by the Patient Health Questionnaire-9,^22^ and activity levels measured using actigraphy (over 7 days), and a time budget assessing meaningful activity (that considers complexity of activities and effort required).^23^ Agoraphobic avoidance was also assessed using a behavioural assessment task, the O-BAT.^11^ The O-BAT was administered by a research assistant. A personalised hierarchy of five real-world situations was created and participants were asked to enter them in order of difficulty (stopping when unable to progress). Ratings of distress were obtained for each step completed. Quality of life was assessed with the five-level EQ-5D,^24^ Recovering Quality of Life questionnaire,^25^ and Questionnaire about the Process of Recovery.^26^ For mediation, we assessed threat cognitions and use of within-situation defence behaviours (using the Oxford Cognitions and Defences Questionnaire [O-CDQ])^27^ and strength of safety beliefs.^28^ Moderators were assessed at baseline by a brief assessment of negative hallucinations when outside, the Beck Hopelessness Scale,^29^ the Body-Esteem Scale for Adolescents and Adults,^30^ and the O-CDQ.^27^ The O-CDQ has two key subscales of threat cognitions and within-situation defences. The threat cognitions scale assesses 14 threat cognitions when outside, including “I will embarrass myself”, “People will judge me negatively”, “I will panic”, “Everyone will watch me”, “People will try to upset me”, and “I won't be able to cope with voices”. The within-situation defences scale assesses ten behaviours when outside, including “I avoid making eye contact”, “When out, I did everything as quickly as possible”, and “When out, I kept my distance from other people”. Each subscale forms a single factor and each has very high internal reliability.

At the end of trial participation, we checked medical notes for serious adverse events, defined by the ISO14155:2011 guidelines for medical device trials as serious if the event resulted in death or was a life-threatening illness or injury, required hospitalisation or prolongation of existing hospitalisation, resulted in persistent or clinically significant disability or incapacity, required medical or surgical intervention to prevent any of the aforementioned outcomes, led to foetal distress, foetal death, or a congenital anomaly or birth defect, or was otherwise considered medically significant by the investigator. An independent data monitoring and ethics committee chair rated whether any serious adverse event was related to treatment or trial procedures. We also checked medical notes for adverse events that were not serious.

### Choice of primary measure

Developed with the guidance of people with lived experience of psychosis, the O-AS is a new scale designed to capture the chronic difficulties with anxiety often seen in patients attending mental health services. The scale is brief, easily understandable, free to use without permissions, and has severity ranges that can guide assessment in clinical services. The O-AS was designed using the principles of behavioural avoidance tasks. It lists eight simple tasks progressing in difficulty from “Stand outside your home on your own for 5 minutes” through “Travel on your own on the bus for several stops” to “Sit in a café on your own for 10 minutes”. Participants were asked whether they could do the task now or whether they could not because of anxiety (score 0 for yes, 1 for no), which provided the avoidance score (0–8). A reduction of 1 point for an individual on the avoidance scale (ie, being able to do a new discrete task) is considered a clinically meaningful improvement. Avoidance scores can be interpreted as: 0=average avoidance, 1–2=moderate avoidance, 3–5=high avoidance, and 6 or greater=severe avoidance. For each task, participants were also asked how anxious they would feel doing that task, on a scale from 0 (no distress) to 10 (extreme distress). These distress scores were summed to provide an overall distress score; 23 or less was interpreted as average distress, 24–46 as moderate distress, 46–66 as high distress, and 67 or greater as severe distress. The psychometric properties of the scale are excellent,^16^ including the test-retest reliability. The avoidance and distress scores have been shown to correlate positively with O-BAT scores,^11^ Agoraphobia Mobility Inventory scores,^17^ Generalised Anxiety Disorder-7 scores,^18^ and overall activity levels assessed by actigraphy. We did not choose the commonly used Agoraphobia Mobility Inventory^17^ (which was developed in the context of anxiety disorder research and asks about anxious avoidance of 26 situations) as the primary outcome measure, because many of the situations assessed in the scale, such as theatres, restaurants, museums, auditoriums, aeroplanes, and boats, are remote from everyday life experiences for many patients with psychosis.

### Statistical analysis

We aimed to enrol 432 participants into the trial, with 216 in each group. This sample size took into consideration an attrition rate of 20%, and would provide 90% power to detect a difference of around 8 (SD 23) in O-BAT anxiety score (using the 0–100 scaling from Freeman and colleagues^11^) from randomisation to 6 weeks (ie, a standardised effect size of 0·35) at a two-sided 5% level of significance. The sample size was reconsidered due to recruitment being interrupted by the COVID-19 pandemic and a lower attrition rate being observed. 350 participants, 175 in each group, with an attrition rate of 10%, would provide 87% power to detect the same effect size. The statistical analysis plan was approved before any analysis of post-randomisation data and it is provided with the full statistical report in the appendix (pp 52–239). The primary analysis included all participants for whom data were available and according to the group participants were randomly assigned to, regardless of any deviation from the protocol. Analyses were done after the last follow-up assessment was completed (with no interim analyses).

Analysis of the primary outcome was done using linear mixed-effects regression, modelling the response at 6 weeks and 26 weeks simultaneously. The baseline outcome measure, stratification variables, time (ie, 6 weeks or 26 weeks), and treatment assignment were fitted as fixed effects with a patient-specific random intercept. An interaction between time and randomised group was also fitted as a fixed effect to allow estimation of treatment effect at each timepoint. A similar approach was used for the secondary outcome analysis. The linear mixed-effects model implicitly accounted for missing data, assuming data were missing at random. A p value of less than 0·05 was used as the level of significance for all tests. Results are reported as mean differences between treatment groups, with 95% CIs. Treatment differences estimated from the linear mixed-effects models were additionally reported as standardised mean differences (mean group difference divided by whole group SD at baseline).

Structural equation models and linear mixed-effects regression models were used to test for mediation of VR therapy effects on the outcome through the putative mediators. Analyses were adjusted for baseline measures of the mediator, outcomes, and possible measured confounders. We included repeated measurement of mediators and outcomes to account for classical measurement error and baseline confounding. The moderation analyses were done using linear regression, modelling the baseline outcome measure, treatment assignment, stratification factors, the moderator, and an interaction between randomised group and the moderator as a fixed effect. The p value for the interaction is reported.

We also performed post-hoc moderation analyses testing whether age, gender, and severity of agoraphobic avoidance and distress at baseline affected treatment response.

Stata (SE) version 16.1 was used for all analyses. This trial is registered with the ISRCTN registry, 17308399.

### Changes to the protocol

Due to the COVID-19 pandemic, three main changes to the protocol were required. First, the original primary outcome measure, a real-world behavioural assessment task (O-BAT),^11^ had to be replaced part way through the trial. Due to the COVID-19 lockdown measures implemented in March, 2020, we were not allowed to continue to administer the O-BAT. The assessment involved face-to-face contact with research staff members, which was prohibited by the NHS trusts. Also, many locations (eg, cafés, shops) used in the O-BAT were closed. We had already developed a new self-report questionnaire version of the O-BAT—the O-AS^16^—for clinical services to use after the trial. The O-AS was being completed as a secondary outcome measure, including in the period before the pandemic, and became the primary outcome measure on Sept 3, 2020. The need to change the primary outcome was approved by the trial Data Monitoring and Ethics Committee on April 22, 2020, and the protocol amendment was approved by the trial sponsor on Aug 5, 2020, NHS South Central—Oxford B Research Ethics Committee on Aug 24, 2020, and the Health Research Authority on Sept 3, 2020. The amendment was made before any analysis of trial outcome data, and the O-BAT data collected before the lockdown began are reported as secondary outcomes. Second, because recruitment had to be suspended for several months in 2020 during the first lockdown, we extended the recruitment period and brought forward the 26-week assessment by up to 6 weeks for the last patients enrolled into the trial (a sensitivity analysis indicated this had no effect on our results; appendix p 140). Third, when recruitment resumed in September, 2020, we added another exclusion criterion: moderate or high risk for a severe course of COVID-19. This criterion was modified in February, 2021, so that when an individual at moderate or high risk had been vaccinated, they could then enter the trial.

### Role of the funding source

The funders of the study had no role in study design, data collection, data analysis, data interpretation, or writing of the report.

## Results

Recruitment took place from July 25, 2019, to May 7, 2021. During this period, recruitment was suspended on March 16, 2020, due to the COVID-19 pandemic, and a staggered return across sites, depending on local circumstances, began from Sept 14, 2020. Final follow-up data were collected on Sept 30, 2021. 551 patients were assessed for eligibility and 346 were enrolled and randomly assigned to the gameChange VR therapy plus usual care group (n=174) or the usual care alone group (n=172; figure). 231 (67%) patients were men and 111 (32%) were women, 294 (85%) were White, the mean age was 37·2 years (SD 12·5), and most patients were single, unemployed, and receiving prescribed antipsychotic medication (table 1).

**Figure:**
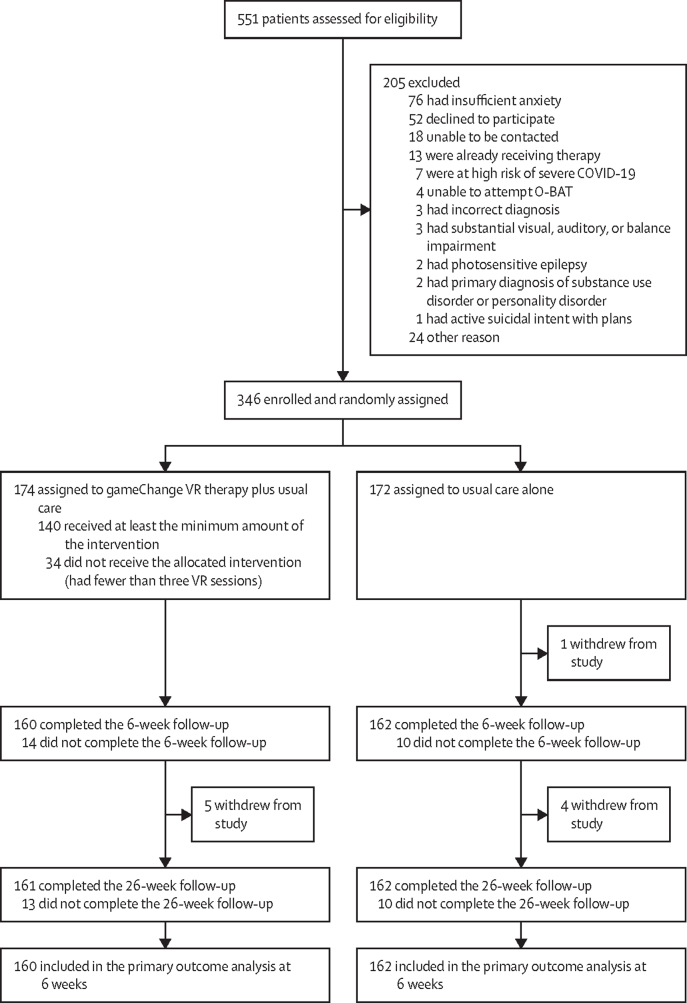
Trial profile O-BAT=Oxford-Behavioural Assessment Task. VR=virtual reality.

**Table 1:** Baseline characteristics

		**gameChange VR therapy plus usual care group (n=174)**	**Usual care alone group (n=172)**
Age, years		36·6 (12·8)	37·8 (12·2)
Gender			
	Women	58 (33%)	53 (31%)
	Men	116 (67%)	115 (67%)
	Other	0	1 (1%)
	Prefer not to say	0	2 (1%)
	Missing	0	1 (1%)
Marital status			
	Single	131 (75%)	138 (80%)
	Married or civil partnership	21 (12%)	14 (8%)
	Cohabiting	6 (3%)	10 (6%)
	Separated	2 (1%)	1 (1%)
	Divorced	9 (5%)	7 (4%)
	Widowed	3 (2%)	1 (1%)
	Missing	2 (1%)	1 (1%)
Ethnicity			
	White	152 (87%)	142 (83%)
	Black British	1 (1%)	1 (1%)
	Black African	1 (1%)	2 (1%)
	Black Caribbean	0	4 (2%)
	Black Other	1 (1%)	0
	Indian	0	2 (1%)
	Pakistani	3 (2%)	3 (2%)
	Other	16 (9%)	17 (10%)
	Missing	0	1 (1%)
Service type			
	Community mental health team	107 (61%)	102 (59%)
	Early intervention	64 (37%)	69 (40%)
	Inpatient	3 (2%)	1 (1%)
Employment status			
	Employed full-time (paid)	10 (6%)	9 (5%)
	Employed part-time (paid)	4 (2%)	4 (2%)
	Working full-time (voluntary)	0	0
	Working part-time (voluntary)	2 (1%)	3 (2%)
	Unemployed (claiming benefits)	112 (64%)	122 (71%)
	Unemployed (not claiming benefits)	8 (5%)	5 (3%)
	Student or in training full-time	5 (3%)	6 (3%)
	Student or in training part-time	3 (2%)	1 (1%)
	Self-employed	4 (2%)	1 (1%)
	Homemaker	2 (1%)	1 (1%)
	Carer	1 (1%)	1 (1%)
	Retired	5 (3%)	2 (1%)
	Other	0	3 (2%)
	Missing	18 (10%)	14 (8%)
Usual living arrangement			
	Living alone (with or without children)	72 (41%)	72 (42%)
	Living with husband or wife	16 (9%)	13 (8%)
	Living with partner	8 (5%)	9 (5%)
	Living with parents	40 (23%)	42 (24%)
	Living with other relatives	9 (5%)	10 (6%)
	Living with others (eg, friends)	10 (6%)	11 (6%)
	Missing	19 (11%)	15 (9%)
Mental health diagnosis (F-code)			
	Schizophrenia	74 (43%)	64 (37%)
	Schizotypal disorder	1 (1%)	2 (1%)
	Delusional disorder	2 (1%)	2 (1%)
	Brief psychotic disorders	5 (3%)	9 (5%)
	Schizoaffective disorder	15 (9%)	11 (6%)
	Other psychotic disorder	2 (1%)	3 (2%)
	Unspecified psychosis	62 (36%)	54 (31%)
	Bipolar disorder with psychotic features	3 (2%)	5 (3%)
	Depressive disorders with psychotic features	7 (4%)	13 (8%)
	Major depressive disorder with psychotic features	3 (2%)	3 (2%)
Prescribed an antipsychotic medication			
	Yes	161 (93%)	156 (91%)
	No	12 (7%)	16 (9%)
	Missing	1 (1%)	0
Prescribed an antidepressant medication			
	Yes	103 (59%)	96 (56%)
	No	70 (40%)	76 (44%)
	Missing	1 (1%)	0
Prescribed an anxiolytic medication			
	Yes	15 (9%)	13 (8%)
	No	157 (90%)	159 (92%)
	Missing	2 (1%)	0

Data are mean (SD) or n (%). Percentages might not sum to 100% due to rounding.

Provision of VR therapy was unaffected by NHS trust pandemic restrictions on face-to-face contact for 147 (84%) of 174 patients in the VR therapy group. These patients received a mean of 5·6 VR sessions (SD 2·0; median 6, range 0–9), with a mean total time spent in VR of 145·2 min (SD 63·4) and total session time of 392·3 min (170·9), and entered a mean of 4·6 (SD 1·6; median 5) of the six VR scenarios. Only five of these patients attended none of the VR sessions. 131 (89%) of these 147 patients had at least the minimum dose of VR therapy (ie, attended three or more sessions). Eight patients assigned to VR therapy could not have the intervention because of COVID-19 pandemic restrictions. They were offered support over the telephone and received a mean of 2·6 telephone sessions (SD 3·6; median 0) for a mean total time of 103·1 min (SD 145·0). 19 patients had their VR therapy curtailed by COVID-19 restrictions. These patients had a mean of 2·8 VR sessions (SD 1·5; median 2) and 4·2 telephone sessions (3·7; 4). Their mean total therapy time was 361·4 min (SD 184·5), with a mean of 71·7 min (35·7) spent in VR.

Overall, 13 (7%) of 174 patients in the VR therapy group attended no VR sessions, 74 (43%) had VR sessions at home, 74 (43%) had VR sessions at trust sites, four (2%) had VR sessions at home and trust sites, and nine (5%) had VR sessions in other settings. The likelihood of home visits varied by whether patients had severe avoidance (n=23, 61%), high avoidance (n=21, 49%), moderate avoidance (n=21, 41%), or average avoidance (n=9, 32%). The most commonly tried VR scenarios were practising opening the front door and being on a street (n=133), visiting a shop (n=133), visiting a café (n=132), getting on a bus (n=113), being in a doctor's surgery (n=120), and being in a pub (n=103).

The level of agoraphobic avoidance at baseline in the usual care alone group was average in 33 (19%) of 172 patients, moderate in 40 (23%), high in 51 (30%), and severe in 48 (28%), and in the VR therapy group was average in 29 (17%) of 174 patients, moderate in 55 (32%), high in 50 (29%), severe in 39 (22%), and missing in one (1%). Compared with the usual care alone group, the VR therapy group had a significant reduction in both agoraphobic avoidance (O-AS adjusted mean difference –0·47, 95% CI –0·88 to –0·06; Cohen's *d* –0·18; p=0·026) and distress (–4·33, –7·78 to –0·87; Cohen's *d* –0·26; p=0·014) at 6 weeks (table 2). There were larger effect size reductions in agoraphobic avoidance (adjusted mean difference –0·89, 95% CI –1·38 to –0·39; Cohen's *d* 0·68; p=0·0004) and distress (–0·86, –1·72 to 0·01; Cohen's *d* 0·43; p=0·052) as assessed by the original primary outcome measure, the O-BAT, in the VR therapy group compared with the usual care alone group among patients who provided end-of-treatment data before the COVID-19 pandemic than with the O-AS. The differences between groups in O-AS scores were not significant at 26 weeks (appendix pp 122–123). There were no significant differences in secondary outcomes between the study groups, except for improvements in the VR therapy group (*vs* the usual care alone group) in recovery as assessed by the Questionnaire about the Process of Recovery at 6 weeks and in O-BAT avoidance scores at 6 weeks and 26 weeks, in the small number of patients who provided such data (table 2, appendix pp 123–124).

**Table 2:** Summary statistics for the primary and secondary outcomes

		**gameChange VR therapy plus usual care group**	**Usual care alone group**	Adjusted treatment difference (95% CI)*	**p value**
**Primary outcomes**					
O-AS avoidance					
	Baseline	3·2 (2·5) [173]	3·4 (2·7) [172]	..	..
	6 weeks	1·9 (2·2) [160]	2·5 (2·6) [160]	−0·47 (−0·88 to −0·06); standardised effect size −0·18 (−0·34 to −0·02)	0·026
O-AS distress					
	Baseline	51·4 (16·4) [174]	52·6 (17·2) [172]	..	..
	6 weeks	41·3 (18·8) [160]	45·8 (20·4) [162]	−4·33 (−7·78 to −0·87); standardised effect size −0·26 (−0·46 to −0·05)	0·014
**Secondary outcomes**					
O-BAT avoidance					
	Baseline	2·7 (1·3) [95]	2·8 (1·3) [96]	..	..
	6 weeks	1·6 (1·7) [59]	2·3 (1·6) [55]	−0·89 (−1·38 to −0·39)	0·0004
	26 weeks	1·1 (1·8) [9]	2·2 (2·1) [11]	−0·87 (−1·63 to −0·11)	0·025
O-BAT distress					
	Baseline	5·5 (1·9) [94]	5·5 (2·1) [95]	..	..
	6 weeks	3·1 (2·4) [55]	3·9 (2·6) [52]	−0·86 (−1·72 to 0·01)	0·052
	26 weeks	2·4 (2·9) [8]	2·6 (2·8) [8]	−0·32 (−1·97 to 1·33)	0·70
O-AS avoidance					
	26 weeks	2·0 (2·3) [157]	2·5 (2·6) [159]	−0·37 (−0·78 to 0·05); standardised effect size −0·14 (−0·30 to 0·02)	0·083
O-AS distress					
	26 weeks	40·7 (20·6) [156]	43·9 (21·6) [161]	−2·50 (−5·98 to 0·97); standardised effect size −0·15 (−0·36 to 0·06)	0·16
Agoraphobia Mobility Inventory-Avoidance					
	Baseline	3·3 (0·7) [167]	3·2 (0·8) [164]	..	..
	6 weeks	2·9 (0·8) [152]	3·0 (0·9) [152]	−0·13 (−0·27 to 0·00)	0·055
	26 weeks	2·9 (0·8) [146]	3·0 (0·9) [145]	−0·11 (−0·25 to 0·03)	0·12
R-GPTS-A (paranoia, reference)					
	Baseline	14·1 (9·3) [158]	12·6 (9·1) [161]	..	..
	6 weeks	10·6 (8·5) [142]	10·7 (8·4) [146]	−1·37 (−2·94 to 0·20)	0·087
	26 weeks	11·0 (9·6) [133]	10·4 (9·1) [134]	−0·39 (−2·00 to 1·22)	0·64
R-GPTS-B (paranoia, persecution)					
	Baseline	17·3 (12·7) [158]	14·2 (12·9) [161]	..	..
	6 weeks	13·0 (11·9) [142]	12·2 (12·6) [146]	−1·66 (−3·73 to 0·40)	0·12
	26 weeks	12·8 (12·6) [133]	11·7 (12·6) [134]	−0·62 (−2·74 to 1·49)	0·57
R-GPTS total (paranoia)					
	Baseline	31·3 (20·7) [158]	26·7 (20·8) [161]	..	..
	6 weeks	23·6 (19·3) [142]	22·9 (19·9) [146]	−3·14 (−6·49 to 0·21)	0·066
	26 weeks	23·8 (21·3) [133]	22·1 (20·4) [134]	−1·10 (−4·52 to 2·33)	0·53
Paranoia Worries Questionnaire					
	Baseline	9·8 (6·2) [158]	8·9 (6·2) [156]	..	..
	6 weeks	7·7 (6·1) [141]	7·5 (6·1) [145]	−0·47 (−1·60 to 0·66)	0·42
	26 weeks	7·3 (6·1) [127]	7·1 (6·5) [134]	−0·15 (−1·32 to 1·01)	0·79
Patient Health Questionnaire-9 (depression)					
	Baseline	15·1 (6·0) [166]	14·1 (6·5) [162]	..	..
	6 weeks	12·5 (6·2) [147]	12·1 (6·0) [150]	−0·24 (−1·48 to 0·99)	0·70
	26 weeks	12·5 (6·7) [134]	11·6 (6·6) [137]	0·11 (−1·17 to 1·39)	0·87
Columbia Suicide Severity Rating Scale (suicidal ideation)					
	Baseline	1·1 (1·3) [155]	0·9 (1·3) [154]	..	..
	6 weeks	0·9 (1·3) [121]	0·8 (1·4) [123]	−0·14 (−0·33 to 0·04)	0·13
	26 weeks	0·8 (1·3) [110]	0·7 (1·3) [113]	−0·06 (−0·25 to 0·14)	0·57
Time budget					
	Baseline	51·9 (17·4) [151]	53·2 (16·8) [142]	..	..
	6 weeks	55·8 (16·0) [124]	57·6 (15·5) [119]	−1·75 (−4·73 to 1·23)	0·25
	26 weeks	57·3 (18·2) [85]	57·7 (17·5) [85]	−1·01 (−4·55 to 2·52)	0·58
Actigraphy, mean number of steps per day					
	Baseline	4727·4 (3016·6) [95]	4942·9 (3107·3) [89]	..	..
	6 weeks	5260·7 (3528·8) [57]	4717·4 (3647·5) [63]	578·7 (−333·8 to 1491·1)	0·21
	26 weeks	5856·1 (2568·1) [26]	5603·9 (2838·3) [27]	751·1 (−533·6 to 2035·8)	0·25
EQ-5D-5L index					
	Baseline	0·5 (0·3) [172]	0·5 (0·3) [170]	..	..
	6 weeks	0·6 (0·3) [152]	0·6 (0·3) [155]	0·03 (−0·02 to 0·08)	0·23
	26 weeks	0·6 (0·3) [142]	0·6 (0·3) [145]	−0·00 (−0·05 to 0·05)	0·86
EQ-5D visual analogue scale					
	Baseline	51·6 (19·2) [171]	53·2 (19·1) [170]	..	..
	6 weeks	56·6 (19·7) [153]	54·9 (20·7) [156]	3·06 (−1·18 to 7·30)	0·16
	26 weeks	56·1 (21·3) [145]	56·7 (22·4) [146]	−0·29 (−4·65 to 4·06)	0·89
Recovering Quality of Life total					
	Baseline	33·6 (13·3) [166]	35·5 (13·2) (163]	..	..
	6 weeks	38·1 (13·8) [149]	39·4 (14·5) [147]	1·06 (−1·53 to 3·65)	0·42
	26 weeks	39·5 (15·1) [136]	40·8 (15·2) [137]	0·57 (−2·10 to 3·25)	0·67
Questionnaire about the Progress of Recovery total					
	Baseline	27·2 (10·7) [173]	28·1 (11·1) [170]]	..	..
	6 weeks	32·4 (11·2) [159]	31·0 (11·3) [159]	2·83 (0·90 to 4·75)	0·0039
	26 weeks	33·1 (11·7) [148]	32·6 (12·1) [151]	1·71 (−0·25 to 3·67)	0·088

Data are mean (SD) [n assessed] unless otherwise stated. VR=virtual reality. O-AS=Oxford Agoraphobic Avoidance Scale. O-BAT=Oxford-Behavioural Assessment Task. R-GPTS=Revised Green et al Paranoid Thoughts Scale.

* VR versus control; mean difference estimated from a linear mixed-effects model adjusting for site, service type, and baseline values of the outcome as fixed effects and participant as the random effect for all outcomes. Standardised effect size=estimated mean difference divided by baseline SD.

As the structural equation models failed to converge, mediation analyses based on parametric regression models alone are shown in table 3. For avoidance and distress, there was significant mediation of treatment outcomes at 6 weeks by threat cognitions and within-situation defence behaviours but not safety beliefs. VR therapy reduced threat cognitions and use of within-situation defence behaviours and each of these mechanisms separately explained approximately one-third of the VR treatment effect. The pattern was similar at 26 weeks but did not reach significance.

**Table 3:** Treatment mediation

		**6 weeks**	**26 weeks**
**O-AS avoidance**			
Threat cognitions			
	Total effect	−0·53 (−0·95 to −0·12); p=0·011	−0·43 (−0·85 to −0·02); p=0·042
	Direct effect	−0·38 (−0·79 to 0·03); p=0·066	−0·30 (−0·72 to 0·12); p=0·16
	Indirect effect	−0·14 (−0·27 to −0·02); p=0·022	−0·11 (−0·23 to 0·02); p=0·089
	Proportion mediated*	0·27	0·25
Within-situation defence behaviours			
	Total effect	−0·50 (−0·91 to −0·08); p=0·019	−0·40 (−0·81 to 0·02); p=0·063
	Direct effect	−0·35 (−0·76 to 0·05); p=0·087	−0·23 (−0·65 to 0·18); p=0·27
	Indirect effect	−0·15 (−0·29 to −0·02); p=0·027	−0·14 (−0·28 to 0·00); p=0·054
	Proportion mediated*	0·31	0·35
Safety beliefs			
	Total effect	−0·57 (−0·99 to −0·15); p=0·0082	−0·50 (−0·92 to −0·08); p=0·021
	Direct effect	−0·50 (−0·92 to −0·08); p=0·019	−0·45 (−0·89 to −0·02); p=0·040
	Indirect effect	−0·04 (−0·12 to 0·04); p=0·32	−0·06 (−0·15 to 0·02); p=0·15
	Proportion mediated*	0·07	0·13
**O-AS distress**			
Threat cognitions			
	Total effect	−4·58 (−8·06 to −1·09); p=0·010	−2·89 (−6·40 to 0·61); p=0·11
	Direct effect	−2·54 (−5·86 to 0·79); p=0·14	−1·36 (−4·78 to 2·05); p=0·43
	Indirect effect	−1·62 (−2·96 to −0·27); p=0·018	−1·21 (−2·58 to 0·16); p=0·084
	Proportion mediated*	0·35	0·42
Within-situation defence behaviours			
	Total effect	−4·38 (−7·87 to −0·89); p=0·014	−2·60 (−6·11 to 0·91); p=0·15
	Direct effect	−2·17 (−5·42 to 1·07); p=0·19	−0·61 (−3·94 to 2·72); p=0·72
	Indirect effect	−1·81 (−3·38 to −0·24); p=0·024	−1·60 (−3·21 to 0·00); p=0·050
	Proportion mediated*	0·41	0·62
Safety beliefs			
	Total effect	−4·89 (−8·49 to −1·29); p=0·0077	−3·14 (−6·75 to 0·47); p=0·088
	Direct effect	−4·23 (−7·80 to −0·67); p=0·020	−2·23 (−5·90 to 1·45); p=0·24
	Indirect effect	−0·45 (−1·31 to 0·42); p=0·31	−0·68 (−1·60 to 0·23); p=0·14
	Proportion mediated*	0·09	0·22

Data are point estimate (95% CI); p value, unless otherwise stated. O-AS=Oxford Agoraphobic Avoidance Scale.

* Indirect effect divided by total effect.

Greater severity of threat cognitions (assessed by the O-CDQ) at baseline resulted in greater treatment benefits with the VR therapy at 6 weeks (avoidance interaction effect –0·06, 95% CI –0·11 to –0·01; p=0·012; distress interaction effect –0·37, –0·74 to 0·01; p=0·054; appendix pp 135–136), which indicates that severity of agoraphobia is likely to moderate outcomes. In the post-hoc analysis of the outcome effects on the primary outcome measure by severity of agoraphobic avoidance and distress, treatment benefits with VR therapy were only seen in the groups with severe and high agoraphobia at baseline, and these benefits were maintained at 26 weeks (table 4). There was no evidence of moderation of treatment effects by the occurrence of negative verbal auditory hallucinations (avoidance interaction effect 0·01, 95% CI –0·05 to 0·07; p=0·68; distress interaction effect 0·08, –0·40 to 0·56; p=0·74), hopelessness (avoidance interaction effect 0·05, –0·02 to 0·13; p=0·17; distress interaction effect 0·17, –0·44 to 0·79; p=0·58), or appearance concerns (avoidance interaction effect 0·01, –0·01 to 0·04; p=0·36; distress interaction effect 0·10, –0·12 to 0·32; p=0·35; appendix p 135). Post-hoc analyses provided no evidence of moderation of treatment effects by age (avoidance interaction effect –0·03, 95% CI –0·06 to 0·01; p=0·11; distress interaction effect –0·12, –0·39 to 0·14; p=0·35) or gender (avoidance interaction effect in women –0·73, –1·45 to –0·00; avoidance interaction effect in men –0·22, –0·73 to 0·29; p=0·26; distress interaction effect in women –5·79, –11·56 to –0·03; distress interaction effect in men –2·95, –7·03 to 1·13; p=0·43; appendix p 242).

**Table 4:** Moderation of VR therapy outcomes by baseline severity of O-AS avoidance and distress

	**gameChange VR therapy plus usual care group**	**Usual care alone group**	**Adjusted mean difference (95% CI); Cohen's** d	**Interaction p value**
**O-AS avoidance at 6 weeks**				
0: average avoidance	27	32	0·26 (−0·74 to 1·25); 0·10	..
1–2: moderate avoidance	53	37	0·08 (−0·75 to 0·91); 0·03	..
3–5: high avoidance	43	48	−0·34 (−1·14 to 0·47); −0·13	..
≥6: severe avoidance	36	43	−1·63 (−2·49 to −0·77); −0·63	0·014
**O-AS avoidance at 26 weeks**				
0: average avoidance	25	31	−0·00 (−1·02 to 1·01); 0·00	..
1–2: moderate avoidance	50	38	0·10 (−0·73 to 0·93); 0·04	..
3–5: high avoidance	45	48	0·33 (−0·45 to 1·12); 0·13	..
≥6: severe avoidance	36	42	−2·06 (−2·91 to −1·20); −0·79	0·0003
**O-AS distress at 6 weeks**				
≤23: average distress	7	11	−0·78 (−15·51 to 13·95); −0·50	..
24–46: moderate distress	56	43	−0·30 (−6·49 to 5·88); −0·02	..
46–66: high distress	63	71	−4·11 (−9·44 to 1·22); −0·24	..
≥67: severe distress	34	37	−10·17 (−17·34 to −3·00); −0·61	0·22
**O-AS distress at 26 weeks**				
≤23: average distress	7	11	7·36 (−9·48 to 24·20); 0·44	..
24–46: moderate distress	53	44	3·78 (−3·33 to 10·88); 0·23	..
46–66: high distress	63	69	−6·37 (−12·48 to −0·27); −0·38	..
≥67: severe distress	33	37	−8·47 (−16·73 to −0·21); −0·50	0·050

Data are n unless otherwise stated; n is the number of participants assessed. VR=virtual reality. O-AS=Oxford Agoraphobic Avoidance Scale.

There were 25 adverse events (in 21 patients) in the VR therapy group and 29 adverse events (in 19 patients) in the usual care alone group (p=0·66; table 5). There were 12 serious adverse events (in nine patients) in the VR therapy group and eight serious adverse events (in seven patients) in the usual care alone group (p=0·37). Of the serious adverse events in the VR therapy group, ten were rated by the Data Monitoring and Ethics Committee as “Not related-definitely not”, two as “Not related-probably not”, and none as “Related-possibly related”, “Related-probably related”, or “Related-definitely related”.

**Table 5:** Adverse events

	**gameChange VR therapy plus usual care group, events (patients)**	**Usual care alone group, events (patients)**
**Adverse events**		
Men	17 (14)	25 (15)
Women	8 (7)	4 (4)
**Serious adverse events**		
Men	10 (8)	8 (7)
Women	2 (1)	0

Data are n (n). VR=virtual reality.

## Discussion

The treatment target of one of the first randomised controlled trials testing psychological therapy, published in 1966, was severe agoraphobia.^31^ Three-quarters of the patients in the pioneering trial were unable to leave their home unaccompanied. After more than 60 sessions of an early form of face-to-face behavioural therapy, provided over 6 months, four of ten patients showed good improvements in symptoms of agoraphobia. In the gameChange trial, significant benefits in agoraphobic avoidance and distress were obtained after six sessions of automated VR therapy supported by various mental health professionals. Uptake of the VR therapy was very high. Overall, the treatment effect sizes were small, due in part to a high proportion of the intervention group scoring low at baseline on anxious avoidance, leading to floor effects (ie, there was little or no room to show improvement on the scale). The effects were specific to agoraphobia symptoms, with the majority of secondary outcomes showing no significant differences between the groups, although there were improvements in perceptions of recovery. Moderation analyses indicated that the VR therapy principally helped patients with severe agoraphobia. Patients with severe avoidance at baseline showed large effect size benefits with VR therapy at the 6-month follow-up. On average, patients with severe avoidance at baseline were able to complete two more O-AS activities, such as walking down the street or going to a shopping centre on their own, 26 weeks after VR therapy. There were also broader outcome benefits with VR therapy in the severe avoidance group, notably improvements in paranoia and recovering quality of life. There were also treatment benefits for reducing distress in everyday situations for those with high baseline agoraphobia. The therapy was designed for patients with difficulties leaving the home or visiting a local shop or taking a local bus due to agoraphobia, and for this group of patients, gameChange VR therapy showed significant benefit. For patients with lower baseline anxiety, who were able to go out locally and perhaps only struggled with complex social interactions, there was less evidence for treatment benefit on agoraphobia symptoms. Our recommendation would be to focus gameChange VR therapy provision initially for patients with high or severe avoidance. gameChange VR therapy is an intervention that has the potential to be deliverable on a large scale for patients with some of the most severe mental health symptoms. The primary outcome measure, the O-AS, has established cutoff scores, which enable services to identify the most suitable patients. The development of this measure, the design of the therapy, and the conduct of the trial all greatly benefited from having the lived experience perspective embedded into every stage of the project from the beginning.

This trial aimed to reduce agoraphobic avoidance of everyday situations and distress when in those situations. Approximately halfway into recruitment, the COVID-19 pandemic occurred. Avoidance of social situations by the public was actively encouraged, which had multiple effects on the trial. First, recruitment was paused, which meant the original planned sample size could not be reached, although the effects of this pause in recruitment were mitigated by our high participant retention rate. Second, there was an enforced change to the primary outcome measure. Fortunately, we were able to substitute a self-report version of the original behavioural avoidance task outcome measure. There is clear consistency in the outcome results obtained by the behavioural avoidance task and the self-report questionnaire, although treatment effects with the behavioural avoidance task were somewhat greater. Behavioural avoidance tasks are more likely to be sensitive to change, because fewer people will show floor effects as an individual hierarchy is constructed. Third, it is plausible that during the pandemic, as trial procedures moved online, fewer patients with severe avoidance were recruited. Fourth, VR therapy delivery was greatly affected for patients who were enrolled into the trial immediately before the lockdown. Several patients were prevented from receiving a dose of therapy because of prohibition of face-to-face contact with staff for research. Opportunities to practise the learning made in VR became more limited. Together, these factors could have reduced the overall treatment effects. Finally, it is not possible to determine whether the overall public messaging to decrease in-person contact, and the long periods when access to public spaces was restricted, might have reduced the treatment effects.

The VR therapy was derived from a cognitive theoretical perspective on anxiety. The therapy reduced a wide range of threat cognitions concerning being outside and the within-situation defence behaviours (eg, avoiding eye contact, scanning faces for signs of judgement or criticism, leaving when starting to get anxious) that maintain them. The change in these two psychological processes mediated treatment benefits, although reverse causation cannot be ruled out from the study design. The therapy therefore seems to have largely worked as intended. However, the development of new beliefs concerning safety did not mediate outcomes. Future research on how to increase such safety beliefs might be a key route to improve clinical effects. The hypothesised psychological processes only explained a proportion of the treatment effects. The contribution of the contact with the mental health professional is unknown, but we presume it was important. Guided digital interventions have greater engagement than unsupported approaches.^32^ The degree to which the outcome effects are explained by the VR or by the contact with the mental health professional could not be determined by this trial. A qualitative peer-research investigation with 20 trial participants in the VR therapy group will detail the gameChange experience.^33^ It is also unknown whether there are any longer-term benefits for patients beyond the 6-month time period assessed in this trial. With the availability of inexpensive, easy-to-use, standalone headsets, which do not need a computer, we believe that there is now the opportunity to leave VR headsets with patients, which would allow patients to increase treatment time and to use the programme at the most opportune times (eg, to gain confidence immediately before going out). This delivery method could be combined with regular review with mental health staff, to help apply the learning to everyday life. Staff support to patients is likely to be important in delivery of successful VR therapy, combined with regular supervision for delivery staff to ensure that the best guidance is provided to patients. gameChange VR therapy could be of great benefit for patients preparing to be discharged from psychiatric hospital,^34^ and for patients with other diagnoses that involve severe anxious avoidance. The therapy could be developed further by expanding the range of everyday scenarios simulated in VR, including those with greater social interaction, and adding further challenges to consolidate the learning. Providing a greater range of content and allowing patients more time with that content is likely to produce even greater clinical benefits. VR is inherently a therapeutic digital medium; patients know they are experiencing simulations, which enables a psychological distance from problematic reactions and provides an opportunity to practise new responses. The process of finding the best uses and implementation methods of this immersive technology at scale in mental health is only beginning.

## Data sharing

Deidentified participant data will be available in anonymised form from the corresponding author upon reasonable request (including a study outline), subject to review and contract with Oxford Health NHS Foundation trust, following the publication of results. The trial protocol has been published and is available in the appendix (pp 2–51). The statistical analysis plan and the full statistical report are available in the appendix (pp 52–239).

## Declaration of interests

DF is a founder and a non-executive director of Oxford VR, which will commercialise the therapy; holds equity in and receives personal payments from Oxford VR; holds a contract for his university team to advise Oxford VR on treatment development; and reports grants from National Institute for Health Research (NIHR), Medical Research Council (MRC), and International Foundation. CH reports grants from NIHR and MRC; and was chair of the National Institute for Health and Care Excellence Guideline for psychosis and schizophrenia in children and young people (CG155). The University of Oxford, Oxford Health NHS Foundation Trust, McPin Foundation, NIHR MindTech MedTech Co-operative, and the Royal College of Art received a share of the licencing fee from Oxford VR for the gameChange software. DMC reports a Senior Investigator Award from the Wellcome Trust and is National Clinical and Informatic Advisor for the NHS Improving Access to Psychological Therapy (IAPT) programme. RD reports a grant from the NIHR, is a clinician working in the NHS delivering cognitive therapy, and receives payments for workshops of cognitive behavioural therapy and royalties from books on cognitive behavioural therapy. SL reports consultancy work and fees from Oxford VR. AP reports a grant from the NIHR. All other authors declare no competing interests.
