# Supplementary material for: Automated virtual reality therapy to treat agoraphobic avoidance and distress in patients with psychosis (gameChange): a multicentre, parallel-group, single-blind, randomised, controlled trial in England with mediation and moderation analyses
Source: Lancet Psychiatry. 2022 May;9(5):375–88. doi: 10.1016/S2215-0366(22)00060-8 (PMC9010306; doi:10.1016/S2215-0366(22)00060-8)

THE LANCET
Psychiatry

Supplementary appendix

This appendix formed part of the original submission and has been peer reviewed. We post it as supplied by the authors.

Supplement to: Freeman D, Lambe S, Kabir T, et al. Automated virtual reality therapy to treat agoraphobic avoidance and distress in patients with psychosis (gameChange): a multicentre, parallel-group, single-blind, randomised, controlled trial in England with mediation and moderation analyses. *Lancet Psychiatry* 2022; published online April 5. https://doi.org/10.1016/S2215-0366(22)00060-8.

Appendix

1. Trial Protocol. Pages 2-51
2. Statistical Analysis Plan. Pages 52-90
3. Statistical Analysis Report. Pages 91-239
4. Additional statistical analysis for reviewers. Pages 240-242
5. gameChange VR images. Pages 243-246

gameChange
Improving lives through VR therapy

Trial Title: The gameChange Trial: A randomised controlled trial testing automated virtual reality cognitive therapy for patients with fears in everyday social situations.

Ethics Ref: 19/SC/0075

IRAS ID: 256895

Chief Investigator:	Professor Daniel Freeman	University of Oxford & Oxford Health NHS Foundation Trust
Investigators:	Dr Felicity Waite	University of Oxford & Oxford Health NHS Foundation Trust
	Professor Ly-Mee Yu	University of Oxford
	Professor David Clark	University of Oxford
	Professor John Geddes	University of Oxford & Oxford Health NHS Foundation Trust
	Dr Jose Leal	University of Oxford
	Andrew Goodsell	University of Oxford & Oxford Health NHS Foundation Trust
	Dr Aitor Rovira	University of Oxford & Oxford Health NHS Foundation Trust
	Dr Thomas Kabir	The McPin Foundation
	Tillie Cryer	The McPin Foundation
	Dr Dan Robotham	The McPin Foundation
	Humma Andleeb	The McPin Foundation
	Dr Jennifer Martin	NIHR Mental Health MedTech Co-Operative & University of Nottingham (UoN)
	Dr Sue Brown	NIHR Mental Health MedTech Co-Operative & University of Nottingham (UoN)
	Dr Michael Craven	NIHR Mental Health MedTech Co-Operative & University of Nottingham (UoN)
	Professor Chris Hollis	NIHR Mental Health MedTech Co-Operative & University of Nottingham (UoN)
	Dr Aislinn Bergin	NIHR Mental Health MedTech Co-Operative & University of Nottingham (UoN)
	Professor Tony Morrison	University of Manchester
	Dr Rob Dudley	Northumberland, Tyne and Wear NHS Foundation Trust
	Dr Eileen O'Regan	Nottinghamshire Healthcare NHS Trust
	Dr Kate Chapman	Avon and Wiltshire Mental Health Partnership NHS Trust
Trial Coordinators:		
	Dr Sinead Lambe	University of Oxford
	Dr Laina Rosebrock	University of Oxford
	Ariane Petit	University of Oxford

Study Sponsor: University of Oxford
Funder: NHS National Institute of Health Research (NIHR) i4i programme and the NIHR Oxford Health Biomedical Research Centre.

© Copyright: The University of Oxford and Oxford University Hospitals NHS Foundation Trust 2016

Chief Investigator Signature:

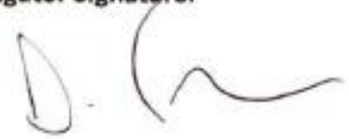

Statistician Signature:

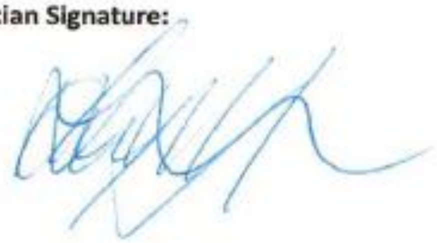

Potential conflicts of interest:

The principal investigator (Daniel Freeman) is a founder and non-executive director of the University of Oxford spin-out company, Oxford VR. Additionally, the sponsor (University of Oxford) is a stakeholder in this company. The VR treatment will be commercialised by Oxford VR.

Confidentiality Statement

This document contains confidential information that must not be disclosed to anyone other than the Sponsor, the investigator's team, HRA, host organisation, NIHR, the DMEC, and members of the Research Ethics Committee, unless authorised to do so.

© Copyright: The University of Oxford and Oxford University Hospitals NHS Foundation Trust 2016

TABLE OF CONTENTS

1. KEY TRIAL CONTACTS 7
2. SYNOPSIS 8
3. ABSTRACT 12
4. BACKGROUND AND RATIONALE 13
5. OBJECTIVES AND OUTCOME MEASURES 14
  5.1. Qualitative sub-study 15
  5.2. Summary of Objectives and Outcome Measures 15
6. TRIAL DESIGN 17
7. PARTICIPANT IDENTIFICATION 19
  7.1. Trial Participants 19
  7.2. Inclusion Criteria 19
  7.3. Exclusion Criteria 19
8. TRIAL PROCEDURES 19
  8.1. Changes due to coronavirus (COVID-19) 20
  8.2. Recruitment 20
  8.3. Screening and eligibility assessment 20
  8.4. Informed consent 21
  8.5. Baseline Assessments 21
  8.6. Randomisation, blinding and code-breaking 22
  8.7. VR therapy 22
  8.8. Control procedure 22
  8.9. Subsequent Visits 22
  8.10. Adverse events will be monitored.Compliance with Trial Interventions 23
  8.11. Discontinuation/Withdrawal of Participants from Trial 23
  8.12. Definition of End of Trial 23
9. QUALITATIVE SUB-STUDY PROCEDURES 23
  9.1. Design 23
  9.2. Participants 24
  9.3. Inclusion criteria: 24
  9.4. Exclusion criteria: 24
  9.5. Sustainability 25
  9.6. Implementation Study 25
  9.7. Data collection 25
  9.8. Timing of data collection 25

© Copyright: The University of Oxford and Oxford University Hospitals NHS Foundation Trust 2016

9.9. Focus groups on mental health inpatient wards 25
10. PSYCHOLOGICAL TREATMENT 26
10.1. Description 26
10.2. Device details 27
11. SAFETY REPORTING 28
11.1. Definitions of Adverse Events 29
11.2. Definitions of Serious Adverse Events 29
11.3. Adverse Device Effect (ADE) 30
11.4. Serious Adverse Device Effect (SADE) 30
11.5. Device Deficiencies 30
11.6. Anticipated Serious Adverse Device Effect 30
11.7. Unanticipated Serious Adverse Device Effect 30
11.8. Causality 31
11.9. Reporting Procedures for Adverse Events 31
11.10. Reporting Procedures for Serious Adverse Events 31
11.11. Reporting to the DMEC 31
11.12. Reporting to the Research Ethics Committee 32
11.13. Device deficiencies 32
11.14. Safety monitoring 32
12. STATISTICS 32
12.1. Power Calculations 32
12.2. Data Analysis 32
12.3. Missing data 34
12.4. Qualitative sub-study 34
13. DATA MANAGEMENT 35
13.1. Source Data 35
13.2. Access to Data 35
13.3. Data Recording and Record Keeping 36
13.4. Data sharing 36
14. QUALITY ASSURANCE PROCEDURES 36
14.1. Risk assessment 36
14.2. Study Monitoring 37
Data Monitoring and Ethics Committee (DMEC) 37
Research Steering Group (RSG) 37
Research Management Group (RMG) 37

© Copyright: The University of Oxford and Oxford University Hospitals NHS Foundation Trust 2016

15. PROTOCOL DEVIATIONS 37
15.1. SERIOUS BREACHES 37
16. PATIENT AND PUBLIC INVOLVEMENT (PPI) 38
16.1. Past PPI 38
16.2. Future PPI 38
17. ETHICAL AND REGULATORY CONSIDERATIONS 38
17.1. Declaration of Helsinki 38
17.2. Guidelines for Good Clinical Practice 39
17.3. Approvals 39
17.4. Reporting 39
17.5. Participant Confidentiality 39
17.6. Expenses and Benefits 39
17.7. Ethical Considerations 39
17.8. Other Considerations 40
18. FINANCE AND INSURANCE 40
18.1. Funding 40
18.2. Insurance 41
18.3. Contractual arrangements 41
18.4. Generation of intellectual property 41
19. PUBLICATION POLICY 41
20. GANTT CHART 42
21. REFERENCES 45
22. APPENDIX A: SCHEDULE OF PROCEDURES 49
23. APPENDIX B: AMENDMENT HISTORY 50

© Copyright: The University of Oxford and Oxford University Hospitals NHS Foundation Trust 2016

1. KEY TRIAL CONTACTS

**Chief Investigator**	Prof Daniel Freeman, Department of Psychiatry, University of Oxford, Warneford Hospital, Oxford OX3 7JX. Email: Daniel.freeman@psych.ox.ac.uk. Tel. 01865 226490.
**Sponsor**	Heather House, Clinical Trials Research Governance, Joint Research Office, University of Oxford, Boundary Brook House, Churchill Drive, Headington, Oxford, OX3 7LQ Email: ctrg@admin.ox.ac.uk. Tel 01865 616480.
**Statistician**	Professor Ly-Mee Yu, Nuffield Department of Primary Care, University of Oxford, Radcliffe Observatory Quarter, Woodstock Road, Oxford. OX2 6GG Email: ly-mee.yu@phc.ox.ac.uk. Tel 01865 617199
**Monitor**	Clinical Trials & Research Governance<br>Joint Research Office, University of Oxford<br>Boundary Brook House<br>Churchill Drive, Headington<br>Oxford, OX3 7LQ
**Committees**	Data Monitoring and Ethics Committee (DMEC).<br><br>Research Steering Committee (RSC).<br><br>Research Management Group (RMG).

© Copyright: The University of Oxford and Oxford University Hospitals NHS Foundation Trust 2016

2. SYNOPSIS

Trial Title	The gameChange Trial: A randomised controlled trial testing automated virtual reality cognitive therapy for patients with fears in everyday social situations.	
Funding	NHS National Institute of Health Research (NIHR), i4i Mental Health Challenge Award and the NIHR Oxford Health Biomedical Research Centre.	
Trial Design	Randomisation to automated virtual reality cognitive therapy (in addition to treatment as usual) or to treatment as usual.	
Trial Participants	Patients with fears in social situations who are diagnosed with psychosis and attending NHS mental health services.	
Planned Sample Size	432 patients (216 TAU, and 216 VR therapy and TAU). A subset of 25 participants will be invited to complete exit interviews. We will also recruit 25-40 staff to take part in an implementation study and 10 stakeholders to explore sustainability.	
Treatment duration	6 weeks.	
Follow up duration	26 weeks.	
Planned Trial Period	24 months for the trial overall.	
	Objectives	Outcome Measures
Primary	Test whether the virtual reality treatment leads to reduction in distress and avoidance of everyday situations.	Oxford Agoraphobic Avoidance Scale (O-AS) (Lambe et al., submitted).
Secondary	1. Test clinical improvements by treatment type in activity levels, psychiatric symptoms, quality of life.	Activity levels: Actigraphy, time-budget measure (Jolley et al., 2006).<br><br>Psychiatric symptoms: Agoraphobia Mobility Inventory-Avoidance (Chambless et al., 1985), O-BAT (Freeman et al., 2016), Revised-Green et al Paranoid Thoughts Scale (Green et al., 2008; Freeman et al, 2019); Paranoia Worries Questionnaire (Freeman et al., 2019), PHQ-9 (Kroenke et al., 2001), Columbia-Suicide Severity

© Copyright: The University of Oxford and Oxford University Hospitals NHS Foundation Trust 2016

		Rating Scale (Posner et al., 2011; Posner et al., 2008).<br><br>Quality of life: EQ-5D-5L (http://www.euroqol.org/), ReQol (Keetharuth et al, 2018), Questionnaire on the Progress of Recovery (Neil et al., 2009).
	2. Test clinical improvements at follow-up.	The above primary and secondary clinical measures.
	3. Determine the cost-effectiveness of the virtual reality treatment.	Client Service Receipt Inventory (Beecham & Knapp, 1992).
	4. Test mediation of treatment effects by changes in safety beliefs, threat cognitions (vulnerability and threat anticipation), and defence behaviours.	Cognition and Defence Behaviours Questionnaire (Lambe et al, in prep) and strength of safety beliefs, vulnerability belief, and threat anticipation assessed using three visual analogue scales (Freeman et al., 2016).
	5. Test moderation of treatment effects (negative auditory hallucinations, hopelessness, appearance concerns, and threat cognitions).	Hallucinations scale (Rosebrock et al, in prep); Beck Hopelessness Scale (Beck, 1988); Body-esteem Scale for Adolescents and Adults (Mendelson et al, 2001); Cognition and Defence Behaviours Questionnaire (Lambe et al, in prep).
Tertiary	Implementation study.<br><br>1a. To carry out a qualitative study of the experience of the virtual reality therapy.<br><br>1b. To explore and compare the challenges of implementing VR therapy in clinical and home settings.<br><br>1c. To assess the feasibility of implementing VR therapy into NHS mental health services.	Qualitative interview / focus group.<br><br>Video recordings of inpatient use of VR

© Copyright: The University of Oxford and Oxford University Hospitals NHS Foundation Trust 2016

		NoMAD questionnaire (Finch et al, 2015).
	2. Assess patient satisfaction with the VR therapy.	Modified version of the Client Satisfaction Questionnaire (Attkisson and Zwick, 1982).
	3. Develop self-report questionnaire measures of the primary outcome measure (O-BAT).	O-AS Questionnaire.
	4. Examine the value proposition of virtual reality treatment for psychosis within the NHS post-trial through exploration of stakeholder priorities.	Secondary analysis of data collected during the trial and interviews with key stakeholders.
Exploratory	1. Examine additional characteristics associated with the experience of psychosis, which may be future treatment targets (mindfulness, self-concept, anhedonia, self-criticism and self-reassurance), at the last follow-up. These are not outcome measures.<br><br>2. Examine cognitions related to coronavirus (COVID-19).	Questionnaires: Southampton Mindfulness Questionnaire (SMQ; Chadwick et al., 2008); Brief Core Schema Scale (BCSS; Fowler et al., 2006); Savoring Beliefs Inventory (SBI; Bryant, 2003), The Forms of Self-Criticising/Attacking & Self-Reassuring Scale (FSCRS; Glibert et al., 2004)<br><br>TOPIC Q (Rosebrock et al., 2021)

ABBREVIATIONS

ADE	Adverse Device Event
AE	Adverse Event
AR	Adverse Reaction
BAT	Behavioural Avoidance Task

© Copyright: The University of Oxford and Oxford University Hospitals NHS Foundation Trust 2016

CBT	Cognitive Behaviour Therapy
CI	Chief Investigator
CONSORT	Consolidated Standards of Reporting Trials
CPMS	Central Portfolio Management System
CRF	Case Report Form
CTRG	Clinical Trials and Research Governance
DMEC	Data Monitoring and Ethics Committee
GCP	Good Clinical Practice
GP	General Practitioner
HRA	Health Research Authority
LEAP	Lived Experience Advisory Panel
MHRA	Medicines and Healthcare products Regulatory Agency
NHS	National Health Service
NNT	Number Needed to Treat
PI	Principal Investigator
PICU	Psychiatric Intensive Care Unit
REC	Research Ethics Committee
SADE	Serious Adverse Device Effect
SAE	Serious Adverse Event
SAR	Serious Adverse Reaction
SOP	Standard Operating Procedure
SPIRIT	Standard Protocol Items: Recommendations for Interventional Trials
SUSAR	Suspected Unexpected Serious Adverse Reactions
TAU	Treatment as Usual
VAS	Visual Analogue Scale
VR	Virtual Reality

© Copyright: The University of Oxford and Oxford University Hospitals NHS Foundation Trust 2016

3. ABSTRACT

Background: Many patients with psychosis find it frightening or difficult to be in everyday social situations. The fears can arise, for example, from paranoia, auditory hallucinations, social anxiety, or negative self-beliefs. These fears lead patients to withdraw from activities, and this isolation and inactivity leads to a cycle of worsening physical and mental health. Breaking this cycle requires highly active treatment that enables patients to learn that they can safely and confidently enter everyday situations. The most efficacious treatment method involves direct coaching in the troubling everyday situations, akin to having a personal trainer. However patients with psychosis seldom receive these life-changing interventions.

To solve this problem we have developed automated psychological treatment using Virtual Reality (VR). VR allows the individual to repeatedly experience computer simulations of the situations they find difficult. A virtual coach helps patients overcome their fears. Importantly, patients are much more willing to go into VR simulations because they know that they are not real but the learning still transfers to the real world. With people with lived experience of psychosis, psychologists, designers, and a programming team, we have developed a new six session virtual reality cognitive treatment to help patients overcome fears in everyday social situations. The VR treatment includes engaging tasks that maximise learning of safety. We will test this treatment in a randomised controlled trial.

Methods: 432 patients attending psychosis services and having anxieties going outside will be randomised (1:1) to the VR cognitive treatment added to treatment as usual or treatment as usual. Blind assessments will be conducted at 0, 6 (post treatment), and 26 weeks. The primary outcome is distress and avoidance of real-life social situations (using a self-report behavioural assessment task). The secondary outcomes are activity levels, psychiatric symptoms, and quality of life. Mediation will also be tested. All main analyses will be intention-to-treat. An economic evaluation will assess cost-effectiveness and there will be qualitative implementation and sustainability studies. The trial is funded by the National Institute of Health Research i4i programme.

Discussion: The trial will determine the clinical effectiveness of a high quality automated VR treatment for patients in psychosis services.

© Copyright: The University of Oxford and Oxford University Hospitals NHS Foundation Trust 2016

4. BACKGROUND AND RATIONALE

Too many patients with psychosis, despite standard treatment, become isolated and inactive, with negative effects on both mental and physical health. Approximately 80% of patients with schizophrenia experience an episode of depression (Upthegrove et al, 2017). Physical activity levels in patients with schizophrenia are reduced on average by approximately two thirds (Lindamer et al, 2008). Over 90% of patients with schizophrenia are unemployed and spend "less time in functional but also in social and leisure activities and more time resting and 'doing nothing' compared to the general population" (Cella et al, 2016). Life expectancy is on average 14.5 years shorter (Hjorthøj et al, 2017), due to largely preventable conditions such as high blood pressure, diabetes, and heart disease. Partly this physical ill health reflects unhealthy lifestyles including inactivity. Our view is that a substantial part of this inactivity arises from avoidance due to anxiety. In a clinical assessment study of 1800 patients with non-affective psychosis, two-thirds of the patients had levels of anxious avoidance equivalent to patients diagnosed with agoraphobia (Freeman et al, submitted). The anxiety in patients with psychosis can arise from a number of sources: fears that others will harm them, voices telling them of danger, social anxiety fears of humiliation and rejection, and negative beliefs about the self that cause a lack of confidence and a sense of vulnerability. But withdrawal from activities because of anxiety need not be inevitable. Appropriate treatment, as seen in the anxiety disorders (e.g. Mayo-Wilson et al, 2014), can produce excellent outcomes. Such treatment involves identifying fearful thoughts and the safety-seeking (or defence) behaviours that maintain those cognitions by preventing receipt and processing of disconfirmatory evidence. The thoughts must then be tested in behavioural experiments in the troubling situations while the defence behaviours are dropped (Clark, 1999). However, there is a dearth of therapists to carry out this skilled work for patients with schizophrenia. It is well-recognised that there is considerable under-provision of psychological therapy for patients with schizophrenia (National Clinical Audit of Psychosis, 2018). There is the additional problem that the sometimes very fearful beliefs of patients with psychosis mean that they can be much less likely to engage in behavioural experiments in the real world before their fears have been somewhat reduced by other means. Our solution is the provision of automated psychological therapy using Virtual Reality (VR).

Virtual reality (interactive computer-generated environments) has been used since the early 1990's to treat anxiety (Rothbaum al, 1994). Meta-analyses indicate that VR treatments for anxiety disorders can produce large treatment effects (Carl et al, 2018) that generalise to the real world (Morina et al, 2015). Previous uses of VR for mental health problems have all depended on a therapist providing the psychological therapy (Freeman, Reeve et al, 2017). In a trial of one hundred patients with a fear of heights, we have shown that the provision of cognitive therapy can be automated using VR by the incorporation of a virtual coach (Freeman, Haselton et al, 2018). The treatment effect sizes in this trial were very large (effect size Cohen's d=2.0; the number of patients needed to treat (NNT) to at least halve fear of heights was 1.3). Automated treatment has the potential to be scalable, removing a key cause of the highly limited access to psychological therapy for patients with psychosis.

VR may also be especially suited the difficulties of patients with psychosis. Patients with strong fears are much more likely to test out their fear expectations in VR because they know it is a simulation but the learning that they make then transfers to the real world (Morina et al, 2015). VR treatment can also include engaging tasks that make the treatment experience much more pleasurable. A graded approach can easily be applied in VR, allowing the individual to repeatedly experience the situations they find difficult and

© Copyright: The University of Oxford and Oxford University Hospitals NHS Foundation Trust 2016

make new learning. Our view is that VR treatments have the potential to be faster, more efficacious, and appealing to patients than traditional face-to-face approaches. We conducted a first test of VR to treat persecutory delusions in patients with psychosis (Freeman et al, 2016). Just thirty minutes in graded VR environments, with the psychological advice provided by a therapist, led to a large reduction in distress in a real-world situation (e.g. going into a shop). VR has been shown to be safe to use with patients with psychosis (e.g. Freeman, Pugh et al, 2010). A recent randomised controlled trial of over one hundred patients with psychosis showed that 16 sessions with a therapist using VR led at follow-up led to a moderate increase in time spent with other people as assessed by an experience sampling method (Pot-Kolder et al, 2018). In the THRIVE trial our team is currently testing an automated four session VR cognitive treatment for patients specifically with persecutory delusions (ISRCTN12497310) (Freeman, Lister et al, 2019).

We have recently developed - using a socially-inclusive design process - a new automated VR cognitive treatment for patients for psychosis having difficulties being in everyday social situations. It is designed to be easy to use, engaging for patients and staff, and delivered with the latest consumer equipment. Therefore this VR treatment has the potential to be widely implemented in treatment services. Psychological treatment that involves direct coaching in the situations that trouble patients with psychosis is rarely available in mental health services. Therefore we set out to determine the *in toto* effects of adding the VR treatment to treatment as usual. This entails a test that randomises patients to receive the VR treatment in addition to usual care or to usual care. We aim to determine the clinical effects on real-world performance, activity levels, psychiatric symptoms, and quality of life. We will also carry out a mixed methods evaluation of the VR treatment in a sub-set of patients (n=25) and staff (n=25-40), the priorities of key stakeholders including commissioners and providers (up to 10), and an overall health economic evaluation of the VR treatment.

Please note: The automated VR treatment will be CE marked before the study starts. We work closely with a regulatory consultant.

5. OBJECTIVES AND OUTCOME MEASURES

The primary research question is: Does automated VR cognitive treatment added to treatment as usual, compared to treatment as usual alone, lead to a post-treatment reduction in real world distress and avoidance for patients with psychosis attending NHS mental health services?

Our primary hypothesis is that:

1. Compared to treatment as usual, VR cognitive therapy added to treatment as usual will reduce distress and avoidance of real world situations (post treatment).

Our secondary hypotheses are:

1. Compared to treatment as usual, VR cognitive therapy added to treatment as usual will increase activity, reduce psychiatric symptoms (paranoia, anxious avoidance, depression, suicidal ideation), and improve quality of life (post-treatment).

2. Treatment effects will be maintained at follow-up.

© Copyright: The University of Oxford and Oxford University Hospitals NHS Foundation Trust 2016

3. The mediators of VR treatment will be safety beliefs, threat cognitions (vulnerability and threat anticipation), and defence behaviours.

4. Treatment effects will be moderated by the occurrence of negative auditory hallucinations in social situations, hopelessness, appearance concerns, and social phobia.

We also include a health economic evaluation of the VR treatment. It will focus on determining the cost of the VR treatment using a microcosting approach, performing a within-trial cost-effectiveness analysis, and extrapolating the within-trial results to a 10 years horizon using a state-transition model.

5.1. Qualitative sub-study

The objectives of the qualitative sub-study are as follows:

1a. To carry out qualitative studies of the implementation and experience of the virtual reality therapy.

1b. To explore and compare the challenges of implementing VR therapy in clinical and home settings.

1c. To assess the feasibility of implementing VR therapy into NHS mental health services.

2. Examine the value proposition of virtual reality treatment for psychosis within the NHS post-trial through exploration of stakeholder priorities.

5.2. Summary of Objectives and Outcome Measures

**Objectives**	**Outcome Measures**	**Time-points of evaluation of this outcome measure**
**Primary objective:**<br><br>1. VR treatment leads to reduction in distress and avoidance of everyday situations.	The primary outcome will be the O-AS (Lambe at al., submitted).	Weeks 0, 6, and 26.
**Secondary objective:**<br><br>1. Test clinical improvements by treatment type in activity levels, psychiatric symptoms, quality of life.	1. Activity levels: Actigraphy, time-budget measure (Jolley et al, 2006).<br><br>Psychiatric symptoms: Agoraphobia Mobility Inventory-Avoidance (Chambless et al., 1985), O-BAT (Freeman et al., 2016), Revised-Green et al Paranoid Thoughts Scale (Green et al., 2008; Freeman et al., 2019; Paranoia Worries Questionnaire (Freeman et al, 2019), PHQ-9 (Kroenke et al, 2001), Columbia-Suicide Severity	1. Weeks 0, 6, and 26.

© Copyright: The University of Oxford and Oxford University Hospitals NHS Foundation Trust 2016

Objectives	Outcome Measures	Time-points of evaluation of this outcome measure
	Rating Scale (Posner et al., 2011, Posner et al., 2008).<br><br>Quality of life: EQ-5D-5L (http://www.euroqol.org/), ReQol (Keetharuth et al, 2018), Questionnaire on the Progress of Recovery (Neil et al., 2009).	
2. Determine the cost-effectiveness of the virtual reality treatment.	2. Client Service Receipt Inventory (Beecham & Knapp, 1992).	2. Weeks 0, 6, and 26.
3. Test mediation of treatment effects by changes in safety beliefs, threat cognitions (vulnerability and threat anticipation), and defence behaviours.	3. Cognition and Defence Behaviours Questionnaire (Lambe et al, in prep) and strength of safety beliefs, vulnerability belief, and threat anticipation assessed using three visual analogue scales (Freeman et al., 2016).	3. Weeks 0, 6, and 26.
4. Test moderation of treatment effects (negative auditory hallucinations, hopelessness, appearance concerns, and social phobia).	4. Moderator variables assessed at 0 weeks: Hallucinations scale (Rosebrock et al, in prep); Beck Hopelessness Scale (Beck, 1988); Body-esteem Scale for Adolescents and Adults (Mendelson et al, 2001); Cognition and Defence Behaviours Questionnaire (Lambe et al, in prep). These are tested for the moderation of the primary outcome at 6 weeks.	4. Week 6.
**Tertiary objective:**<br><br>1a. To carry out a qualitative study of the experience of the virtual reality therapy.<br><br>1b. To explore and compare the challenges of implementing VR therapy in clinical and home settings.<br><br>1c. To assess the feasibility of implementing VR therapy into NHS mental health services.	1a. Semi-structured interview.<br><br>1c. NoMAD questionnaire (Finch et al, 2015).<br><br>2. Revised therapy satisfaction scale.	6-10 weeks

© Copyright: The University of Oxford and Oxford University Hospitals NHS Foundation Trust 2016

Objectives	Outcome Measures	Time-points of evaluation of this outcome measure
2. Assess patient satisfaction with the VR therapy.<br><br>3. Examine the value proposition of virtual reality treatment for psychosis within the NHS post-trial through exploration of stakeholder priorities.	3. Secondary analysis of data collected during the trial and interviews with key stakeholders.	6 weeks<br><br>Throughout.
**Exploratory Objective:**<br><br>1. Examine additional characteristics associated with the experience of psychosis, which may be future treatment targets (mindfulness, self-concept, anhedonia, self-criticism and self-reassurance). These are not outcome measures.<br><br>2. Examine cognitions related to coronavirus.	1. Mindfulness: Southampton Mindfulness Questionnaire (SMQ; Chadwick et al., 2008); Self-Concept: Brief Core Schema Scale (BCSS; Fowler et al., 2006); Anhedonia: Savoring Beliefs Inventory (SBI; Bryant, 2003), The Forms of Self-Criticising/Attacking & Self-Reassuring Scale (FSCRS; Glibert et al., 2004)<br>2. TOPIC Q (Rosebrock et al., 2021).	26 weeks.<br><br>0, 6, 26 weeks.

6. TRIAL DESIGN

The design is a multicentre, parallel group randomised controlled trial with single blind assessment to test whether the automated VR cognitive treatment added to treatment as usual, compared to treatment as usual alone, leads to a post-treatment reduction in real world distress and avoidance for patients with psychosis attending NHS mental health services. Treatment as usual will be measured but remain as usual in both groups. Assessments will be carried out at 0 (baseline), 6 (post treatment), and 26 (follow-up) weeks by a researcher blind to treatment allocation. See figure 1 below. The trial will be registered with the ISRCTN registry and the protocol submitted for publication.

© Copyright: The University of Oxford and Oxford University Hospitals NHS Foundation Trust 2016

Figure 1. Trial flow diagram

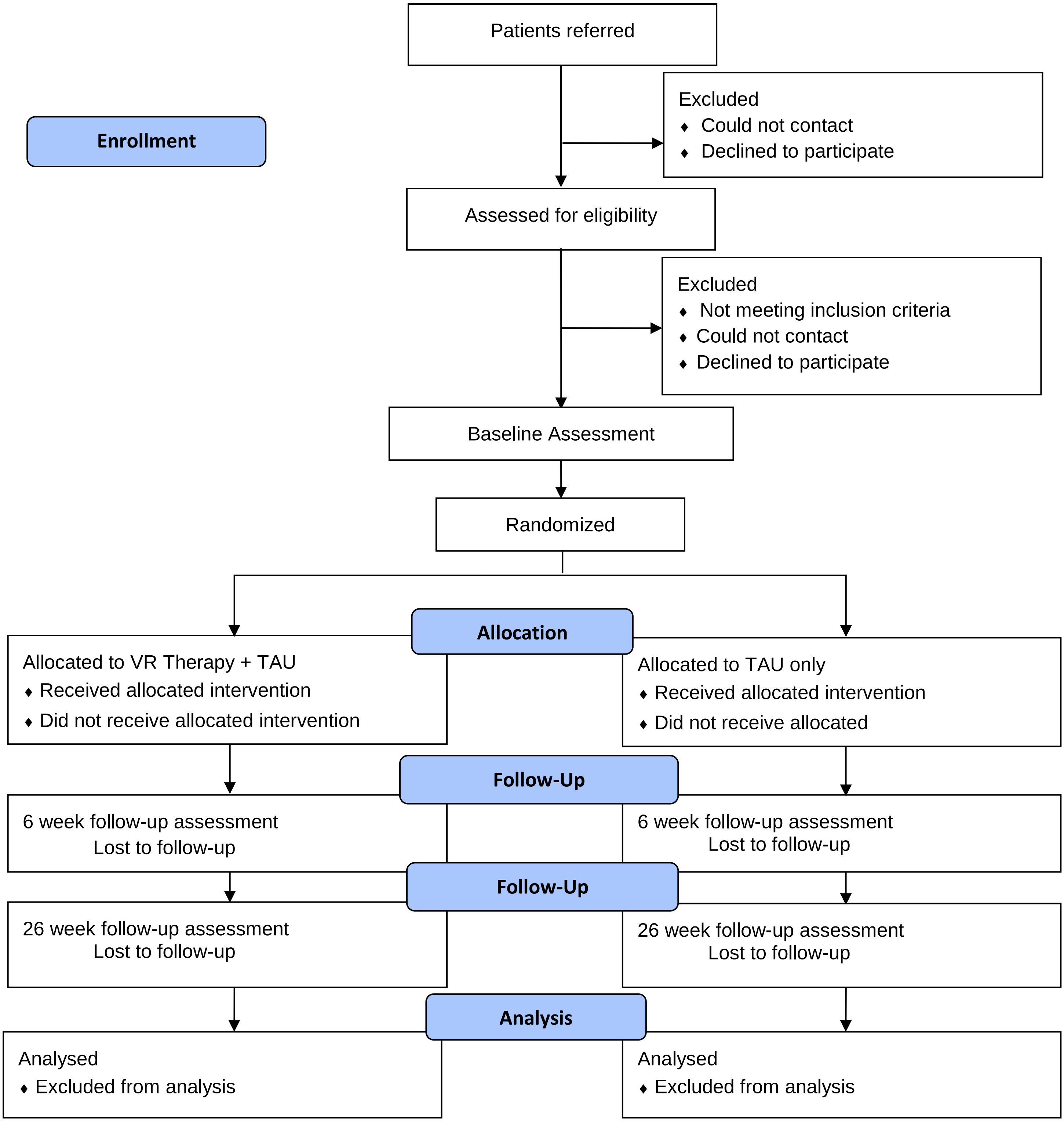

© Copyright: The University of Oxford and Oxford University Hospitals NHS Foundation Trust 2016

7. PARTICIPANT IDENTIFICATION

7.1. Trial Participants

The trial participants will be patients with psychosis and self-reported difficulties going outside among other people primarily due to anxiety (assessed using a screening version of the Oxford - Behavioural Assessment Task (O-BAT) self-report questionnaire).

7.2. Inclusion Criteria

- Adults aged 16 years or older;
- Attending a NHS mental health trust for the treatment of psychosis;
- Clinical diagnosis of schizophrenia spectrum psychosis (F20-29) or an affective diagnosis with psychotic symptoms (F31.2, 31.5, 32.3, 33.3) (ICD-10, WHO, 2010);
- Having self-reported difficulties going outside their home primarily due to anxiety (and hence would score on the primary outcome) that they would like treated;
- And participant is willing and able to give informed consent for participation in the trial.

7.3. Exclusion Criteria

- Unable to attempt an Oxford - Behavioural Assessment Task (O-BAT) at baseline (e.g. due to being unpermitted to leave a psychiatric ward);
- Photosensitive epilepsy;
- Significant visual, auditory, or balance impairment;
- Current receipt of another intensive psychological therapy (or about to start it within the 6 week trial therapy window);
- Insufficient comprehension of English;
- In forensic settings or Psychiatric Intensive Care Unit (PICU);
- Organic syndrome;
- Primary diagnosis of alcohol or substance disorder or personality disorder;
- Significant learning disability;
- Or current active suicidal plans.
- A participant may also not enter the trial if there is another factor, which, in the judgement of the investigator, would preclude the participant from providing informed consent or from safely engaging with the trial procedures. We will suspend recruitment for people who have any of the conditions that would make them high or moderate risk (clinically vulnerable) for a severe course of COVID-19 (https://www.nhs.uk/conditions/coronavirus-covid-19/people-at-higher-risk/whos-at-higher-risk-from-coronavirus/). However, people who are at moderate or high risk for a severe course of COVID-19 will be able to join the trial if they have received the COVID-19 vaccine (subject to medical advice). Reason for exclusion will be recorded in line with CONSORT 2010 Statement (Schulz et al, 2010).

8. TRIAL PROCEDURES

The schedule of procedures is summarised in Appendix A.

© Copyright: The University of Oxford and Oxford University Hospitals NHS Foundation Trust 2016

For all trial home visits (assessments and/or therapy sessions), the lone worker Standard Operating Procedure (SOP) should be followed. For further information on recruitment and assessment, please see the relevant SOP.

8.1. Changes due to coronavirus (COVID-19)

The O-BAT will no longer be used as social distancing guidelines prevent its administration. The O-AS will replace the O-BAT as the primary outcome measure. Where it is not possible for the questionnaires to be administered face-to-face, they can be administered online, on the phone or via post. We will offer flexibility for participants to conduct any of the follow-up assessments by phone, online or via post, following an appropriate risk assessment. For remote assessments, abbreviated or self-report versions of interview measures will be used as necessary e.g. the self-report C-SSRS (Posner et al., 2008). See Remote Assessment SOP (Version 1, 24/07/20).

The TOPIC Q will be administered at all timepoints to examine coronavirus-related cognitions.

We will suspend recruitment for people who have any of the conditions that would make them high or moderate risk (clinically vulnerable) for a severe course of COVID-19 (https://www.nhs.uk/conditions/coronavirus-covid-19/people-at-higher-risk/whos-at-higher-risk-from-coronavirus/). However, people who are at moderate or high risk for a severe course of COVID-19 will be able to join the trial if they have received the COVID-19 vaccine (subject to medical advice).

An addendum to the Participation Information Sheet will indicate the steps the research team has taken to reduce the risk of COVID-19 transmission.

8.2. Recruitment

The principal method of recruitment will be via seeking referrals to the trial from the relevant clinical teams in the participating mental health Trusts (adult community mental health teams; early intervention services, and inpatient units). Patients interested in taking part will then be approached by the research team with the approval of the clinical team, given information about the trial, and screening conducted. All suitable patients will be given at least 24 hours to consider taking part in the trial, although in practice it is typically a week. Our Lived Experience Advisory Panel (LEAP) have also emphasised the importance of patients of Trusts self-initiating referral to the trials, in order to minimise the chances that particular patients are overlooked by clinical teams or the clinician was not present at a referral meeting. Hence we will also advertise the study (including via social media, press releases, online, posters present in NHS buildings and other relevant sites). Hence, patients will be able to self-refer to take part in the study. However, in all instances we will also seek to confirm that a member of the clinical team gives approval for a patient to enter the trial and to complete the necessary screening of eligibility and risk status.

8.3. Screening and eligibility assessment

The key screening with the patient is for self-reported difficulties going outside their home due to anxiety (and hence would score on the primary outcome) that they would like treated. We need to know that the person has the sorts of difficulties treated in the VR therapy, which focuses on anxiety in everyday situations, such as being on the street, in a shop, or on a bus. This is established in a brief discussion with the patient and the use of a screening version of the Oxford - Behavioural Assessment Task (O-BAT) self-report guide.

© Copyright: The University of Oxford and Oxford University Hospitals NHS Foundation Trust 2016

8.4. Informed consent

Written versions of the Participant Information and Informed Consent will be presented to the participants detailing no less than: the exact nature of the trial; what it will involve for the participant; the implications and constraints of the protocol; the known side effects and any risks involved in taking part. An addendum to the Participation Information Sheet will indicate the steps the research team has taken to reduce the risk of COVID-19 transmission. We will discuss this document with participants when we meet with them. It will be clearly stated that the participant is free to withdraw from the trial at any time for any reason without prejudice to future care, without affecting their legal rights and with no obligation to give the reason for withdrawal.

The participant will be allowed as much time as wished to consider the information, and the opportunity to question the Investigator, their mental health team or other independent parties to decide whether they will participate in the trial. Written informed consent will then be obtained by means of participant-dated signature and dated signature of the person who presented and obtained the Informed Consent Form. We will also accept written informed consent electronically. Oral consent can be taken over the phone using the oral consent form, which will be signed and dated by the person who obtained the informed consent orally. The person who obtained the consent must be suitably qualified and experienced, and have been authorised to do so by the Chief Investigator. A copy of the signed Informed Consent Form will be given to the participant. If collecting orally or electronically, the Consent Form will be sent to the participant via secure post or password-protected email. The original signed form will be retained at the trial site and uploaded to the participant's clinical notes.

8.5. Baseline Assessments

The measures have been successfully used in the previous pilot study and other clinical studies by the research team. Assessments are in person, typically in clinic rooms or at home (for patients who find it difficult to leave their residence), or remotely. Basic demographic and clinical data will be collected (e.g. age, gender, ethnicity, clinical diagnosis). The primary outcome, avoidance and distress of everyday situations as measured by the Oxford Agoraphobic Avoidance Scale (O-AS) (Lambe et al., submitted), will be measured at baseline, six weeks, and 26 weeks. The O-AS is a questionnaire that comprises eight real-world tasks. For each task the person is asked to decide if 'Yes, I could do this now' or 'No, I'd get too anxious' and also to rate the anxiety they would feel doing each task. This therefore produces an avoidance score (with higher scores indicating lower avoidance) and a distress score (0-10, with higher scores indicating greater distress) for each level. Other outcomes that will be collected at baseline include, as in the THRIVE Trial (ISRCTN12497310), activity levels assessed using actigraphy (over 7 days), complemented with a time-budget assessing meaningful activity (Jolley et al, 2006). The EQ-5D-5L (http://www.euroqol.org/) and ReQol (Keetharuth et al., 2018) will assess quality of life. Suicidal ideation (Columbia Scale; Posner, 2011; Posner, 2008), overall paranoia (R-GPTS; Green, 2008; Freeman et al., 2019), Paranoia Worries Questionnaire (Freeman et al, 2019, anxious avoidance (AMI-A; Chambless et al., 1985 and where possible the O-BAT; Freeman et al, 2016), and levels of depression (PHQ-9; Kroenke et al, 2001) will be assessed. Additionally, quality of life will be assessed using the Questionnaire about the Process of Recovery (QPR) (Neil et al., 2009). For mediation, we will assess threat cognitions and use of defence behaviours (CDBQ; Lambe et al, in prep) and strength of safety, vulnerability and threat anticipation beliefs (Freeman et al, 2016). Moderators will be assessed at baseline only by a brief assessment of negative hallucinations when outside (Rosebrock et al, in prep), the Beck Hopelessness Scale (Beck, 1988), the Body-esteem Scale for Adolescents and Adults (Mendelson et al, 2001), and the Cognition

© Copyright: The University of Oxford and Oxford University Hospitals NHS Foundation Trust 2016

and Defence Behaviours Questionnaire (Lambe et al, in prep). Adverse events will be monitored. We will record service use, and other relevant health economic data, using the Client Service Receipt Inventory (Beecham & Knapp, 1992).

8.6. Randomisation, blinding and code-breaking

Participants will be randomised once they have completed the baseline assessment. Participants will be allocated to one of the trial arms using a 1:1 allocation ratio. Randomisation will be carried out by a validated online system, Sortition, designed by the University of Oxford Primary Care Clinical Trials Unit. Randomisation using a permuted blocks algorithm, with randomly varying block size, will be stratified by site (Bristol/Manchester/Newcastle/Nottingham/Oxford) and service type (in-patient/early intervention/community mental health team).

The research assessors will be blinded to group allocation, but the patients and staff member present will not be (they cannot be blinded to what psychological intervention is delivered or received). The staff members setting up and running the VR software will inform patients of the randomisation outcome, to ensure the research assessors remain blinded to group allocation. Precautionary strategies to prevent unblinding of allocation include: the staff member and assessor considering room use and booking arrangements; patients being reminded by the assessor not to talk about their allocation result; and, after the initial assessment, the assessor not looking at the patient's clinical notes. If an allocation is revealed between assessment sessions, this is logged by the trial coordinator and re-blinding will occur using another assessor.

8.7. VR therapy

See section 10.1.

8.8. Control procedure

Participants who are allocated to the control arm will continue to receive their usual care. No additional interventions will be offered by the research team. Treatment as usual for the participants within this trial will typically consist of long term prescription of psychiatric medications, and meetings with a mental health practitioner. Treatment as usual will vary across individuals and mental health trusts. We will collect detailed data on treatment as usual (which will also inform the health economic evaluation).

8.9. Subsequent Visits

There are two further trial assessments: at 6 and 26 weeks. The 6 week and 26 week assessments will be conducted up to 10 weeks after the assessment due date i.e. up to 16 weeks post-randomisation for the 6 week assessment and up to 36 weeks post-randomisation for the 26 week assessment. If the follow-up is not completed within these windows, it will be considered missing data. For participants who were randomised in the last four months of recruitment, 26 week assessments will be brought forward by up to 6 weeks i.e. they will be conducted from 20 weeks post-randomisation. This is to ensure data collection is completed within the study schedule.

The full battery of assessments, described above in 'Baseline Assessments', will be completed at all time points. Three additional measures will be included at the 26 week assessment based on patient interest of additional characteristics associated with psychosis (see Section 5.2): Mindfulness, or paying attention to

© Copyright: The University of Oxford and Oxford University Hospitals NHS Foundation Trust 2016

the present moment without judgement, will be examined with the Southampton Mindfulness Questionnaire (SMQ; Chadwick et al., 2008). The SMQ is a 16 item self-report questionnaire. Self-concept (how we think about, evaluate, and perceive ourselves) will be assessed with the 12-item self-report questionnaire the Brief Core Schema Scale (BCSS: Fowler et al., 2006). We will investigate beliefs about focusing on the positive aspects of experiences, which is related to the experience of anhedonia, using the Savoring Beliefs Inventory (SBI; Bryant, 2003), a 24 item self-report measure. The Forms of Self-Criticising/Attacking & Self-Reassuring Scale (FSCRS; Glibert et al., 2004) will be used to explore self-critical and self-reassuring cognitions.

8.10. Adverse events will be monitored.Compliance with Trial Interventions

Participants will be supported to engage in the interventions by either a research team member (with honorary trust contract) or other NHS mental health staff member, under supervision of the trial team. For the intervention, the number of sessions and time in VR will be recorded. As the VR interventions use preprogramed virtual environments, possible deviation from the intervention protocol within the VR should be minimal. Three sessions will be considered the minimum adherent treatment dose.

8.11. Discontinuation/Withdrawal of Participants from Trial

Withdrawal of participants from the trial assessments

Each participant has the right to withdraw from the trial at any time. Withdrawal from the trial or qualitative sub-studies will not result in exclusion of the previously collected data for that participant from analysis (unless this is specifically requested). The reason for withdrawal will be recorded in the CRF.

Withdrawal of participants from the trial intervention

Each participant has the right to withdraw the trial intervention at any time. In addition, the Investigator may discontinue a participant from the VR intervention at any time if the Investigator considers it necessary for any reason including:

- An adverse event which requires discontinuation of the trial intervention
- Ineligibility e.g. presence of photosensitive epilepsy, which was not known to the team at screening

If the participant is withdrawn due to an adverse event, the Investigator will arrange for follow-up visits or telephone calls until the adverse event has resolved or stabilised.

The reason for withdrawal will be recorded in the CRF.

8.12. Definition of End of Trial

The end of trial is the date of the last assessment of the last participant.

9. QUALITATIVE SUB-STUDY PROCEDURES

9.1. Design

© Copyright: The University of Oxford and Oxford University Hospitals NHS Foundation Trust 2016

Interviews and quantitative data will be used to explore staff, NHS stakeholders, and patient experience of the intervention, at each site.

9.2. Participants

Interviews will be conducted with between 5 and 10 stakeholders, selected for their rich in-depth experience of purchasing or providing services for people with psychosis.

Interviews will be conducted with healthcare staff involved in delivering the intervention (approximately 5-8 staff at each of the 5 sites, up to 40 in total), mostly healthcare staff involved in its delivery, but also clinicians recruiting to the study, service leads and research staff supporting the trial. Service users who received the VR intervention will also be interviewed (approximately 5 people at each of the 5 sites – 25 in total), including participants who withdrew or did not wholly complete the intervention.

Focus groups will also be conducted with NHS staff employed to work on inpatient wards in any capacity, and patients staying on inpatient wards (approximately 3-8 staff and 3-8 patients per ward, 1-2 wards per site). Patients will not be participants in the full randomised controlled trial but will be given a demonstration of the VR therapy. Patients will be first approached about the study by their clinical care team.

9.3. Inclusion criteria:

For staff and stakeholders to be eligible to take part in the qualitative sub-project(s) they must meet the following criteria:

- Be employed or contracted for employment by the National Health Service;
- Be working within or alongside mental health services;
- (For the trial experience interviews/questionnaires) have referred or delivered virtual reality therapy as part of gameChange;
- (For the stakeholder interviews) have responsibility for providing, purchasing or commissioning mental health services.

For staff and patients to be eligible to take part in the focus groups on wards they must meet the following criteria:

- **Be employed on the ward as a staff member or staying on the ward as a patient**
- Willing to consent to being audio recorded (to allow analysis of the findings).

9.4. Exclusion criteria:

People will not be eligible to take part in the qualitative sub-study if they meet any of the following criteria:

- Work exclusively for a private healthcare service;
- (For trial experience interviews/questionnaires) are based at an NHS site outside of those involved in the trial.

© Copyright: The University of Oxford and Oxford University Hospitals NHS Foundation Trust 2016

- **(For the focus groups on wards) photo-sensitive epilepsy**

9.5. Sustainability

To ensure that gameChange is useful to the NHS, we will use qualitative methodology that seeks to explore concepts constructed from data collected in previous work packages and from extant documents. Whilst additional data may need to be collected, it is anticipated that the primary data collection methods will be qualitatively through interviews with key stakeholders, such as mental health commissioners, to explore the priorities of those purchasing and delivering services for people with psychosis.

9.6. Implementation Study

The aim of this study is to identify and understand the variety of issues that will affect the implementation of the gameChange intervention into healthcare services. The aim is to provide insights that are relevant to the potential future implementation of gameChange within services, and to identify potential refinements that may be helpful for the future. This study will explore the implementation (deployment) of gameChange, including how its deployment varies and what adaptations are made to its use. It will also explore the experience of delivering the gameChange intervention and how it fits into the working lives of staff.

Semi-structured interview schedules will be prepared and piloted with relevant stakeholders (including the study Lived Experience Advisory Panel (LEAP)).

9.7. Data collection

The implementation study will make use of data relating to outcomes and other variables that are encompassed within the main trial design. In addition, quantitative measures will be used (NoMAD questionnaire (Finch et al. 2015)) alongside customised items (e.g. time taken to set up equipment) to understand its impact on staff working processes and patient experience. Quantitative measures that are in addition to the NoMAD questionnaire will be explored and designed with input from staff and patients, after establishing feasibility and utility with these groups. Any additional measures would be submitted to the ethics committee as amendments.

9.8. Timing of data collection

Interviews with service users will be conducted throughout the duration of the study, soon after their completion of the intervention and 6 week assessment. Healthcare staff will predominantly be interviewed in the second half of the study, so that study processes have become embedded and staff have had the chance to reflect on the intervention’s use over time; however, a small number of initial interviews will take place nearer the start of the trial to pick up on challenges, barriers and adaptations, and to see if these can inform better roll out of the trial across all sites. Quantitative data collection will take place according to the data being sought (i.e. data relating to a specific point of use will be collected at that time, whereas the NoMAD questionnaire will be collected towards the end of the study to allow for some routinisation).

9.9. Focus groups on mental health inpatient wards

© Copyright: The University of Oxford and Oxford University Hospitals NHS Foundation Trust 2016

These focus groups aim to identify specific barriers and facilitators to implementing VR therapy on mental health wards. The focus groups will be held on 1-2 inpatient wards at each of the trial sites. Each group will have 3-8 participants. Separate focus groups with staff and patients will be held. If a participant prefers they can complete an interview alone with the researcher rather than in a group. During the focus groups, participants will be asked open questions that aim to elicit staff and patient attitudes to VR therapy in inpatient settings. Staff and service users are well-placed to address the lived experience of being situated on the ward. In order to ensure as many staff can participate as possible, staff focus groups will not be stratified by role, which may limit the quality of the data. Participants will be given the option to try out wearing the VR headset and see some of the therapy. It is not recommended to use VR if you have photosensitive epilepsy. Therefore, patients with such a diagnosis will not be approached by their clinical care team about the study, and before staff consent to the study they will be asked if they ever been diagnosed with photosensitive epilepsy, and if they have, it will be explained that they are not able to take part. The focus groups will be conducted in months 4-18 of the trial.

10. PSYCHOLOGICAL TREATMENT

10.1. Description

A mental health professional will be in the room when the treatment is given. This person will help the patient put on the VR headset and start the programme. The staff member will also encourage the person to take the learning from VR into the real world. The applications will run through the Steam software application on a laptop computer connected to a head-mounted display and accessories. All hardware is already commercially available and has not been modified for the trial. Number of sessions, treatment time, and staff grade of person administering the treatment, will be recorded. Satisfaction will be assessed after completion of the last treatment session using a modified version of the Client Satisfaction Questionnaire (Attkisson and Zwick, 1982) and we will also assess negative effects from VR using a checklist. Participants are free to withdraw from treatment at any point.

VR Cognitive therapy (VRCT). This treatment aims for patients to test their fear expectations around other people in order to relearn safety. The treatment is not designed as exposure therapy (participants are not asked to remain in situations until anxiety reduces) but as repeated behavioural experiment tests (to learn that they are safer than they had thought). The treatment is designed to be delivered in approximately 6 sessions of thirty minutes. Three sessions will be considered the minimum (adherent) dose of therapy. However participants can proceed at their own pace, meaning that a fewer or more number of sessions is allowed. A virtual coach guides the person through the treatment, including encouraging the dropping of defence behaviours, and elicits feedback to tailor the progression of the treatment. When first entering VR, the patient is guided in a calm VR space how to use VR (i.e. the basic functions). They then go into the coach’s virtual office. At the beginning of the first session, the virtual coach explains the rationale behind the treatment, and the participant selects which one of six virtual reality situations that they would like to begin in. The six virtual reality scenarios are a: café, GP waiting room, pub, bus, street scene, and newsagent. Each scenario has five degrees of difficulty (e.g. the number and proximity of people in the social situation increases) and participants work their way through each level of difficulty. There are game type tasks within a number of the levels. The participant can choose a different scenario in each session or repeat a previous situation. Throughout the sessions, participants’ responses to questions from the virtual coach are given by means of a virtual watch. Belief ratings are repeated within VR at the end of each treatment session. Figure 2 provides a summary of the treatment design.

© Copyright: The University of Oxford and Oxford University Hospitals NHS Foundation Trust 2016

Figure 2. The structure of the VR treatment.

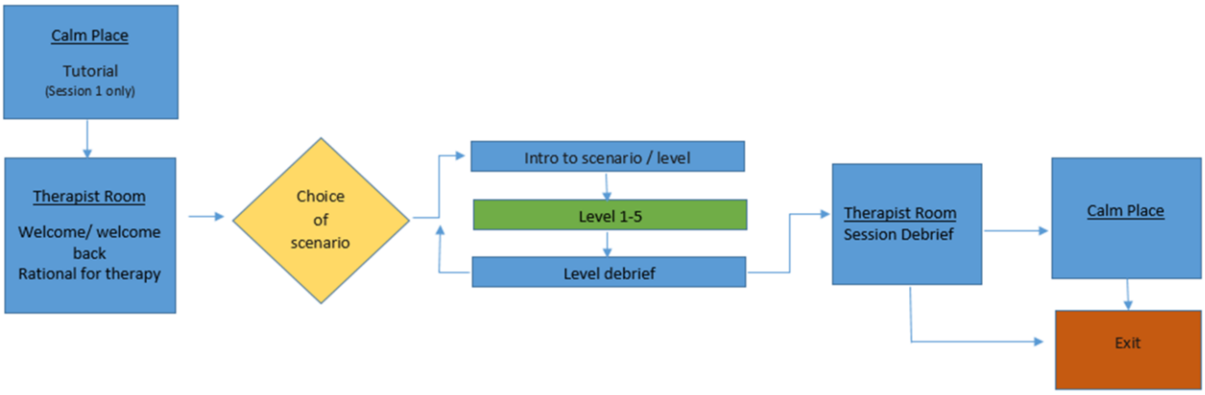

10.2. Device details

Description of device

The gameChange VR treatment is a virtual-reality application recommended for adults (16+) attending psychosis services who have anxieties when outside in everyday situations. This software is intended to reduce anxieties around other people and therefore to help participants feel safer and more comfortable around people. The aim for the outcome is that patients feel more able to go outside into everyday situations. The treatment was programmed by the University of Oxford spin-out company, Oxford VR (www.oxfordvr.org). The treatment will be a CE marked Class I Active Medical Device- Z301 (Standalone Software), in conformity with the essential requirements and provisions of the EC Directive 93/42/EEC (Medical Devices).

Manufacturer details

Oxford VR is the manufacturer for this device.

Details of software

The VR gameChange software application is composed of a set of virtual environments, including different scenes created using 3D models, ambient audio, and 3D computer characters, with animations and speech. The environments are driven by source code which handles the logic of the program, the behavior of the computer characters, as well as the user interaction and data storage. The code is implemented on top of Oxford VR background libraries and third-party libraries. The software is built using Unity (Unity Technologies©). Unity acts as a render engine, displaying the virtual environments to the user through the headset.

Use of device

© Copyright: The University of Oxford and Oxford University Hospitals NHS Foundation Trust 2016

Detailed guidance on use is included within the application; participants will have a short tutorial showing them how to interact with the virtual environment and advising them of safety precautions. The application will be run and monitored by a staff member, who will remain present throughout the session.

Details of accessories required

The application will run through the Steam® software application on a laptop computer connected to a headset and accessories. All technical requirements will be as per headset requirements. Accessory hardware and software are already commercially available and have not been modified for the trial.

Anonymised data collected during the session (e.g. participant number, time in VR, difficulty level reached) will be automatically stored by the application on the password protected laptop computer hard disk.

Handling, maintenance, storage & accountability

Physical accessories will be maintained as per manufacturer's instructions. The headset will use a wipeable insert, and both this and the handheld controllers will be cleaned after each use, using recommended hygienic wipes, by the staff member present during the session. Participants will not come into direct contact with the laptop computer or 'lighthouses'. Due to coronavirus, we will enhance our cleaning and hygiene procedures for using the VR equipment. Details can be found in the VR Decontamination SOP.

The clinician will check that the laptop computer has the most up to date version of the software before each session, and if necessary, download this to the computer prior to the session.

Labelling

The software will contain appropriate labels, such as the version number and manufacturer. The hardware will be labelled for traceability.

Contraindications

Contraindications for use include:

- Photosensitive epilepsy
- Significant auditory or visual impairment
- Insufficient comprehension of English

For full details, see 'Exclusions' section. All participants will be screened prior to entry to the study.

Precautionary measures

A staff member will set up the hardware for the use of the device and while the device is in use, a staff member will be present at all times. Participants will only be entered into the study with the permission of their clinical teams and following screening to ensure that no contraindications to use are present. Adverse events will be monitored. Further details on the device may be found in the device specification.

11. SAFETY REPORTING

Adverse events are rare in our studies, even though this patient group has a higher rate than the general population for the occurrence of adverse events. For example, suicide attempts occur at a higher rate, as do physical health problems. In our Worry Intervention Trial with 150 patients with persistent persecutory

© Copyright: The University of Oxford and Oxford University Hospitals NHS Foundation Trust 2016

delusions followed for six months, no patients died or were admitted to secure units during the study but there were six suicide attempts (two in the psychological treatment intervention group, and four in the t control group), and two serious violent incidents (one in each group). None were deemed by the DMEC to be related to the trial. Serious adverse events related to psychological reactions (i.e. SAR or SUSAR) are extremely rare and have not occurred in any of our studies.

In order to monitor for adverse events, we maintain close links with the participant's clinical team throughout, have a staff member present throughout while the device/VR equipment is in use, and record events that we become aware of during a participant's participation. For the full list of adverse events we record, see the SOP. We also check medical notes at the end of a patient's participation for the following events pre-specified as adverse: (1) All deaths; (2) Suicide attempts; (3) Serious violent incidents; (4) Admissions to secure units; (5) Formal complaints about therapy.

If significant concerns about possible future risk to self or others are raised in assessment or intervention sessions, we risk assess the nature, severity, and likelihood of the risk. This is discussed with a team clinical psychologist (if not already present) and the patient's clinical team is informed as a matter of urgency.

11.1. Definitions of Adverse Events

Any untoward medical occurrence, unintended disease or injury, or untoward clinical signs in participants, whether or not related to the investigational medical device. This includes adverse events related to the VR intervention group and to the control and also to all research procedures involved.

We note that a temporary increase in anxiety symptoms is expected in any psychological treatment involving confronting a feared situation and this would not be considered an adverse event.

11.2. Definitions of Serious Adverse Events

An adverse event is defined by the ISO14155:2011 guidelines for medical device trials as serious if it:

- Results in death or,
- Is a life-threatening illness or injury or,
- Requires [voluntary or involuntary] hospitalisation or prolongation of existing hospitalisation or,
- Results in persistent or significant disability or incapacity or,
- Medical or surgical intervention required to prevent any of the above,
- Leads to foetal distress, foetal death or consists of a congenital anomaly or birth defect or,
- Is otherwise considered medically significant by the investigator.

Life threatening in the definition of an SAE refers to an event in which the subject was at risk of death at the time of the event; it does not refer to an event that hypothetically might have caused death if it were more severe. Clinical judgement should be exercised in deciding whether an SAE is serious in other situations.

Important: AE's that are not immediately life-threatening or do not result in death or hospitalisation but may jeopardise the subject or may require intervention to prevent one or the other outcomes listed, should be considered serious.

© Copyright: The University of Oxford and Oxford University Hospitals NHS Foundation Trust 2016

A planned hospitalization for a pre-existing condition, without a serious deterioration in health, is not considered to be a serious adverse event. We note that admissions to psychiatric hospital are expected in this client group.

To ensure no confusion or misunderstanding of the difference between the terms "serious" and "severe", which are not synonymous, the following note of clarification is provided: The term "severe" is used to describe the intensity (severity) of a specific event (as in mild, moderate, or severe myocardial infarction); the event itself, however, may be of relatively minor medical significance (such as severe headache). This is not the same as "serious," which is based on patient/event outcome or action criteria usually associated with events that pose a threat to a participant's life or functioning. Seriousness (not severity) serves as a guide for defining regulatory reporting obligations.

11.3. Adverse Device Effect (ADE)

Adverse event related to VR Investigational Medical Device. This includes adverse events resulting from insufficient or inadequate instructions for use, deployment, installation, or operation, or any malfunction of the software. It also includes any event resulting from user error or intentional misuse of VR.

User error refers to an act or omission of an act that results in a different device response than intended by the manufacturer or expected by the user.

11.4. Serious Adverse Device Effect (SADE)

Adverse device effect that has resulted in any of the consequences characteristic of a serious adverse event. This includes Device Deficiencies that might have led to a serious adverse event if:

- Suitable action had not been taken or,
- Intervention had not been made or,
- If circumstances had been less fortunate.

11.5. Device Deficiencies

Inadequacy of the VR medical device (i.e. the VR application) with respect to its identity, quality, reliability, safety or performance. Device Deficiencies include malfunctions, end user errors, and inadequate labelling. Some deficiencies may have led to an adverse device effect or a serious adverse device effect, and should be treated as ADEs/SADEs.

11.6. Anticipated Serious Adverse Device Effect

A serious adverse device effect which by its nature, incidence, severity or outcome has been previously identified in the risk analysis report or the Investigator’s Brochure. For VR we do not anticipate any Serious Adverse Device Effects.

11.7. Unanticipated Serious Adverse Device Effect

Serious adverse device effect which by its nature, incidence, severity or outcome has not been identified in the current version of the risk analysis report or the Investigator’s Brochure.

© Copyright: The University of Oxford and Oxford University Hospitals NHS Foundation Trust 2016

11.8. Causality

The relationship between the investigational medical device or other research procedure and the occurrence of each adverse event will be assessed and categorised. The investigator will use clinical judgement to determine the relationship. Alternative causes, such as natural history of the participant's underlying condition, concomitant therapy, other risk factors etc. will be considered. The Investigator will also consult the current version of the risk analysis report and/or the investigator's brochure.

Classification	Relationship	Definition
Related	Definitely	• Starts within a time related to the study device/procedure *and*<br>• No obvious alternative medical explanation.
	Probably	• Starts within a time related to the study device/procedure *and*<br>• Cannot be reasonably explained by known characteristics of the patient's clinical state.
	Possibly	• Starts within a time related to the study device/procedure *and*<br>• A causal relationship between the intervention and the adverse event is at least a reasonable possibility.
Not related	Probably not	• The time association or the patient's clinical state is such that the study device/procedure is not likely to have had an association with the observed effect.
	Definitely not	• The AE is definitely not associated with the study device/procedure.

11.9. Reporting Procedures for Adverse Events

See relevant SOP for full details of reporting procedures.

11.10. Reporting Procedures for Serious Adverse Events

All serious adverse events that come to our attention are reviewed by the study team. These include serious events which are:

- Related to the device and those which are non-device related;
- Anticipated and unanticipated serious events;
- Device Deficiencies that might have led to a serious adverse event if:
    - Suitable action had not been taken or, intervention had not been made or,
    - If circumstances had been less fortunate.

SAEs are recorded using the Serious Adverse Event Report Form. The study team will make an initial assessment of whether the SAE is potentially related to the device and report to the regulatory authorities within the appropriate timescales. The decision about relatedness will later be ratified by the DMEC.

11.11. Reporting to the DMEC

© Copyright: The University of Oxford and Oxford University Hospitals NHS Foundation Trust 2016

For all Serious Adverse Events, the study Chief Investigator or clinical trial co-ordinator will notify the Chair of the Data Monitoring and Ethics Committee for a decision on whether it is potentially related to the device (VR application), and/or trial procedures more generally. Any initial decision on relatedness to the device, will be ratified by the DMEC.

11.12. Reporting to the Research Ethics Committee

A serious adverse event (SAE) occurring to a participant will be reported to the REC that gave a favourable opinion of the study where the event was 'related' (resulted from administration of any of the research procedures) and 'unexpected' in relation to those procedures. Reports of related and unexpected SAEs will be submitted within 15 working days of the Chief Investigator becoming aware of the event, using the HRA report of serious adverse event form (see HRA website).

11.13. Device deficiencies

Device deficiencies, which are not considered to be contributory toward SADEs shall be noted by any member of the research team using the Device Deficiency Report. Any new device deficiencies identified, which were not considered in the initial risk analysis, will be added to the risk analysis and to the Investigators Brochure.

11.14. Safety monitoring

We will form a Data Monitoring and Ethics Committee (DMEC) with an independent clinician chair, and which will at the minimum also include an independent statistician and further independent clinician. The data and management of Serious Adverse Events will be overseen by the chair of the Data Monitoring and Ethics Committee (DMEC).

12. STATISTICS

12.1. Power Calculations

We will recruit around 432 participants into this trial, with 216 in each arm. This sample size takes into consideration a maximum attrition rate of 20%, and provide 90% power to detect a difference of around 8 (standard deviation =23) in O-BAT score, from randomisation to 6 weeks (i.e. standardised effect size of 0.35) at 5% level of significance (2-sided).

12.2. Data Analysis

A full statistical analysis plan will be written prior to recruitment. All analyses will be conducted in accordance with Oxford Primary Care CTU statistics SOPs.

We will report data in line with the CONSORT 2010 Statement showing attrition rates and loss to follow-up. The primary analyses will be carried out using the intention-to-treat principle. That is, after randomisation, participants will be analysed according to their allocated intervention arm irrespective of what intervention they actually receive, and with data available from all participants included in the

© Copyright: The University of Oxford and Oxford University Hospitals NHS Foundation Trust 2016

analysis including those who do not complete therapy. Every effort will be made to follow up all participants in both arms for research assessments.

Baseline variables will be presented by randomised group using frequencies (with percentages) for binary and categorical variables and means (and standard deviations) or medians (with lower and upper quartiles) for continuous variables, along with minimum and maximum values and counts of missing values. There will be no tests of statistical significance nor confidence intervals for differences between groups on any baseline variables. There will be no planned interim analysis for efficacy or futility on the primary outcome.

We will test the primary hypothesis for between-group difference in the primary outcome (O-AS score at 6 weeks) using a linear mixed effects model which models the response at 6 weeks, and 26 weeks, with baseline outcome measure, stratification variables, and treatment assignment as fixed effects, with a patient specific random intercept. An interaction between time and randomised group will be fitted as a fixed effect to allow estimation of treatment effect at all time points. The linear mixed effects model will account for missing data assuming data are missing-at-random (MAR). Standard residual diagnostics will be assessed for the appropriateness of the model. $P<0.05$ will be used as the level of statistical significance. Similar mixed effect models will be used to analyse secondary outcomes.

The mediation analysis will investigate putative mediational factors using modern causal inference methods. This involves using parametric regression models to test for mediation of VRCT on outcome through the putative mediators. Analyses will adjust for baseline measures of the mediator, outcomes, and possible measured confounders. We will include repeated measurement of mediators and outcomes to account for classical measurement error and baseline confounding.

The identified moderator variables (negative auditory hallucinations, hopelessness, appearance concerns, and social phobia) will be considered for moderation of the intervention effect on the primary outcome. Any other subgroup analyses will be pre-specified in the analysis plan prior to the final analysis.

A microcosting approach will be used to inform the cost per patient of the VR treatment. This will make use of trial data collected on staff training, sessions, number of patients per device as well as the expected lifetime of the device, capital, equipment, maintenance and software required to provide the intervention. The within-trial health economic analysis will describe and compare the costs and outcomes of the two trial arms. Incremental cost per activity gained (primary outcome) will be estimated and the costs and remaining outcomes (utilities, psychiatric symptoms, and wellbeing) assessed separately. This will be informed by a health economics statistical plan written prior to the economic analysis. The health economics will use an NHS and social care services perspective with resource utilisation valued using national cost datasets and EQ-5D-5L data converted into utilities using the UK tariffs. A broader perspective including lost earnings, patient out-of-pocket costs, and criminal justice costs will also be considered. A state-transition model will be developed to extrapolate the within-trial analysis and estimate the incremental costs per quality-adjusted life years (QALYs) gained from using the VR treatment, supported by the trial data, literature reviews, and discussions with clinical experts. Uncertainty around the incremental cost-effectiveness ratio will be reported using the cost-effectiveness plane and the cost-effectiveness acceptability curve. The maximum reimbursable price of the VR treatment conditional on the willingness to pay per QALY will be determined. We will then estimate the affordability to the NHS of a decision to implement the VR treatment (33-36 months). This will take the form of budget impact analysis using a time horizon of 3 years to be consistent with NICE, informed by the results of the trial health economics analysis.

© Copyright: The University of Oxford and Oxford University Hospitals NHS Foundation Trust 2016

12.3. Missing data

Missing data on individual measures will be pro-rated if more than 80% of the items are completed; otherwise the measure will be considered as missing.

We will explore the mechanism of missing data by looking for associations between participant characteristics and the likelihood of non-response to questionnaires at different time points. This can be done using a regression model for binary outcomes (1=response; 0=non-response) with independent variables measured at baseline.

We will also carry out sensitivity analysis for the primary outcome using methods which do not assume a MAR mechanism such as pattern-mixture models, to assess the robustness of this assumption. If different results are obtained from a pattern-mixture model compared to the mixed effects model then it is likely that the MAR assumption is not valid. As part of our exploration of treatment effects we will also produce a per-protocol or Complier Average Causal Effect (CACE) analysis with appropriate caveats.

12.4. Qualitative sub-study

Sustainability

The interviews will be analysed using a qualitative methodology to explore the value proposition alongside existing data.

Implementation study

Qualitative analysis will be conducted on an ongoing basis so that emergent findings can feed into subsequent data collection; thus data collection and analysis will be iterative processes that are somewhat concurrent. The same will apply to some aspects of quantitative data collection (e.g. time taken to set up equipment) which will be available to the team during the study. Other aspects of quantitative data collection (e.g. NoMAD questionnaire) will be analysed descriptively, when available, and will be considered alongside corresponding qualitative data to provide richer insight into the patterns being observed.

The study has been designed to explore typical issues related with implementation and sustainability, drawing from normalisation process theory (NPT). Interviews with service users will explore: the experience of using the VR intervention and any support they required/received alongside the intervention; expected outcomes, and whether they have been felt or noticed; any unanticipated changes or consequences; expectations of the VR intervention, and extent of their engagement with it; understanding around how the intervention worked; experience of trial procedures; impact of the receipt of intervention on care delivery, including relationship with staff; views around the intervention's future use, and suggested changes. Interviews with healthcare staff will explore: their role in delivering the intervention, and experience of it; processes, timing and routines of delivery; how VR sits alongside / within care delivery (and the wider imperatives of service delivery); suitability of training; barriers and facilitators; adaptations; contextual factors; who the intervention reached (and didn't reach), suited (and didn't suit); how it affects working practices; monitoring of intervention outcomes (and feeding this back into existing care); views around the intervention's future use.

Focus groups on inpatient wards

© Copyright: The University of Oxford and Oxford University Hospitals NHS Foundation Trust 2016

A thematic analysis of the focus group data will be conducted. Some observation and ethnographic analysis of participants trying out the VR for the first time will also be used by analysing any video footage collected. This is in order to gain greater understanding as to participants' experience of the VR (tertiary objective), for example their immediate reactions to the VR and how they interact with the VR space, for example the extent to which they feel confident to walk around.

13. DATA MANAGEMENT

13.1. Source Data

Source documents are where data are first recorded, and from which participants' CRF data are obtained. These include, but are not limited to, hospital records (from which medical history and previous and concurrent medication may be summarised into the CRF), interview recordings, and trial assessment measures.

We keep data from the assessments, collected on paper from the participant assessments. Additionally, belief ratings made within the VR environment will be collected. All documents, including electronic files, will be stored safely in confidential conditions. On all trial-specific documents, other than the signed consent, the participant will be referred to by the trial participant number, not by name. CRF entries will be considered source data if the CRF is the site of the original recording (i.e. there is no other written or electronic record of data) (e.g. self-report questionnaires).

CRFs will be transferred to the Clinical Trials Unit whom will complete the data quality checks. Hard copies will be transferred via a secure courier service, and electronic copies will be emailed using encrypted email addresses where possible, and if not available, in password protected files. Once the data quality checks are complete, the CRFs and any source data will be stored in a locked cabinet in a locked room for ten years post publication of the trial results. Interview and focus group audio recordings for the inpatient ward study will be taken using dictaphones. Once the interview or focus group is complete, the recording will be downloaded onto a University or NHS computer, and protected with a password. Once the recording has been downloaded, the recording will be deleted from the dictaphone. Similarly, video recordings will be saved onto a University or NHS computer, protected with a password, and permanently deleted from the video recorder. The audio files will be sent securely via OxFile to a professional transcription service who will be required to sign a confidentiality agreement. Transcriptions will be received back via OxFile and stored in a password protected file on a University or NHS computer. Transcriptions will be anonymised. The potentially identifiable audio recordings will remain password protected on the University or NHS computer as they will need to be listened to during analysis in order to capture the emotional tone of participant responses. For data confidentiality procedures during COVID-19 and remote working, see the Remote Assessment SOP (Version 1, 24/07/20).

13.2. Access to Data

Direct access will be granted to authorised representatives from the Sponsor, host institution and the regulatory authorities to permit trial-related monitoring, audits and inspections.

© Copyright: The University of Oxford and Oxford University Hospitals NHS Foundation Trust 2016

13.3. Data Recording and Record Keeping

All trial data will be entered on paper or electronic CRFs and transcribed or entered directly to the clinical data management system. This clinical database will be built and managed by the PC-CTU in line with PC-CTU SOPs and will hold and allow data management of all data points required to conduct the final analysis. The clinical database will be built on an externally validated secure web-based platform allowing for data tracking by use of date stamped audit logs. Within this database participants will be identified only by a unique study ID to offer patient confidentiality and protect against bias. A separate database will be used to securely store identifiable patient information required to contact patients and permit follow up. Access to these data will be strictly on a need to know basis. The database will ensure secure login for staff at participating sites and facilities for manual entry of data and upload of files where appropriate.

Data will be entered from paper CRFs to the clinical database, or recorded directly on eCRFs as soon as possible after the study visit. Validation of all data entered into the clinical database is achieved by programming study specific checks or through manual review. All discrepancies generated are reviewed by the Data Manager and a query sent to site for clarification if necessary. Prior to database lock a dataset review will be undertaken by the Data Manager and Trial Statistician. All critical data items are 100% checked against original source documents, where applicable, to ensure accuracy and an error rate is established across all fields to ensure a consistently accurate dataset.

Interview recordings will be taken using Dictaphones. Once the interview is complete, the interview recording will be downloaded onto a University or NHS computer, and protected with a password. Once the recording has been downloaded, the recording will be deleted from the Dictaphone.

Details of the data management procedure will be documented in a trial specific data management plan (DMP) which will be reviewed and signed by all applicable parties prior to the first patient being enrolled. A clinical data manager will be assigned to the study supervised by the PC-CTU senior data manager and PC-CTU SOPs will be followed.

13.4. Data sharing

Where identifiable information needs to be shared (i.e. in relation to safeguarding) this will be done primarily via emails between two NHS email accounts. Where this isn’t possible, emails may be sent with password protected attachments, or via an encrypted USB stick.

14. QUALITY ASSURANCE PROCEDURES

The trial will be conducted in accordance with the current approved protocol, GCP, relevant regulations and standard operating procedures. We employ a regulatory consultant to advise us on compliance with medical device specific regulations. Data will be evaluated for compliance with the protocol and accuracy in relation to source documents. All electronic data entry is double checked against the source documents. A DMEC will meet before the start of the trial, with the subsequent meeting frequency to be agreed by committee members. The committee will be chaired by a clinician with relevant expertise.

14.1. Risk assessment

A risk assessment and monitoring plan will be prepared before the study opens and will be reviewed as necessary over the course of the study to reflect significant changes to the protocol or outcomes of monitoring activities. A formal risk assessment of the VR therapy will also be conducted.

© Copyright: The University of Oxford and Oxford University Hospitals NHS Foundation Trust 2016

14.2. Study Monitoring

Monitoring will be performed according to the trial specific Monitoring Plan. Study committees

Data Monitoring and Ethics Committee (DMEC)

A DMEC has been formed to monitor the safety and progress of the trial. The DMEC is comprised of independent experts in areas relevant to the trial: clinical expertise, medical device expertise, and trial methodology and statistics expertise. The DMEC will meet at least twice a year, and will be governed by a charter that adheres to the MRC DMEC guidance. The DMEC will be chaired by an independent expert.

The DMEC will conduct a review of all SAEs for the trial reported during the quarter and cumulatively. The aims of this committee include:

- To pick up any trends, such as increases in un/expected events, and take appropriate action
- To seek additional advice or information from investigators where required
- To evaluate the risk of the trial continuing and take appropriate action where necessary

Research Steering Group (RSG)

As per the requirements of the funder, we have formed an RSG. The RSG includes the senior members of the research team, and includes expertise in all areas relevant to the trial: clinical expertise, trial expertise, implementation expertise, qualitative expertise, and lived experience involvement expertise. The RSG is tasked with keeping oversight of the whole project, and progress according to the GANTT chart. The RSG will meet quarterly.

Research Management Group (RMG)

The research management group was formed to oversee the trial processes. The RMG includes representation from the lead research site and the CTU. The RMG will meet monthly in the lead up to the start of the trial.

15. PROTOCOL DEVIATIONS

A study related deviation is a departure from the ethically approved study protocol or other study document or process (e.g. consent process or administration of study intervention) or from Good Clinical Practice (GCP) or any applicable regulatory requirements. Any deviations from the protocol will be documented in a protocol deviation form and filed in the study master file.

15.1. SERIOUS BREACHES

A “serious breach” is a breach of the protocol or of the conditions or principles of Good Clinical Practice which is likely to affect to a significant degree –

(a) the safety or physical or mental integrity of the trial subjects; or

(b) the scientific value of the research.

In the event that a serious breach is suspected the Sponsor must be contacted within 1 working day. In collaboration with the C.I., the serious breach will be reviewed by the Sponsor and, if appropriate, the

© Copyright: The University of Oxford and Oxford University Hospitals NHS Foundation Trust 2016

Sponsor will report it to the approving REC committee and the relevant NHS host organisation within seven calendar days.

16. PATIENT AND PUBLIC INVOLVEMENT (PPI)

16.1. Past PPI

The project has had extensive PPI. Principally this has occurred via The McPin Foundation, a charity that exists to “transform mental health research by putting the lived experience of people affected by mental health problems at the heart of research methods and the research agenda”. A co-applicant on the grant application is from The McPin Foundation. Three other people with lived experience commented on the grant application and a focus group of people with lived experience was convened so that they could try VR and comment upon the application. Following the award of the grant there has been considerable PPI into the design of the VR treatment.

A Lived Experience Advisory Panel (LEAP) has been formed to advise and shape the development of the treatment, the trial protocol, and implementation into services. The LEAP is organised by the McPin Foundation. It comprises of 12 individuals with lived experience of psychosis drawn from each of the study sites (Bristol, Manchester, Newcastle, Nottingham, Oxford). The LEAP have already met a number of times. For the protocol they have advised on: the choice of outcome measures, recruitment methods, the format of recruitment materials, and the content and wording of study materials. The LEAP have also reviewed and commented on this document. In the period after the submission of the protocol for HRA approval the LEAP will work further on the study materials and any changes made will be submitted for approval as amendments.

In addition to the LEAP, we have also worked with people with lived experience from each of the trial sites to develop the VR treatment. A number of workshops were held. Through these workshops, people have contributed to the selection of the VR scenarios, the therapeutic tasks within the scenarios, and style of VR coach. These workshops entailed people with lived experience sharing their ideas, reviewing design concepts, and testing these out within VR. In addition to these workshops, there has been weekly input from a smaller group of individuals with lived experience to gain prompt feedback on details of design.

16.2. Future PPI

PPI will continue throughout the trial. First, LEAP meetings will occur over the course of the trial. The LEAP will advise on any difficulties that occur in the trial. The LEAP will also contribute to the dissemination strategy. Second, there will be a qualitative evaluation of the VR treatment, with the interviews carried out by lived experience researchers. This work will be run by The McPin Foundation. Third, a McPin staff member sits on fortnightly gameChange review meetings and on the Research Steering Committee (RSC) comprised of senior team members.

17. ETHICAL AND REGULATORY CONSIDERATIONS

17.1. Declaration of Helsinki

© Copyright: The University of Oxford and Oxford University Hospitals NHS Foundation Trust 2016

The Investigator will ensure that this trial is conducted in accordance with the principles of the Declaration of Helsinki.

17.2. Guidelines for Good Clinical Practice

The Investigator will ensure that this trial is conducted in accordance with relevant regulations and with Good Clinical Practice.

17.3. Approvals

The protocol, informed consent form, participant information sheet and any proposed advertising material will be submitted to an appropriate Research Ethics Committee (REC), HRA (where required) and host institution(s) for written approval.

The Investigator will submit and, where necessary, obtain approval from the above parties for all substantial amendments to the original approved documents.

The VR treatment will be CE marked before use within the trial.

17.4. Reporting

The CI shall submit, on request, an Annual Progress Report to the REC, HRA (where required), host organisation and Sponsor. Regular reports on the progress of the study will also be submitted to the NIHR as per the terms of the funding agreement. In addition, an End of Trial notification and final report will be submitted to the REC, host organisations, and Sponsor.

17.5. Participant Confidentiality

The study will comply with the General Data Protection Regulation (GDPR) and Data Protection Act 2018, which require data to be de-identified as soon as it is practical to do so. The processing of the personal data of participants will be minimized by making use of a unique participant study number only on all study documents and any electronic database(s). All documents will be stored securely and only accessible by study staff and authorised personnel. The study staff will safeguard the privacy of participants' personal data.

17.6. Expenses and Benefits

For each trial assessment time point (i.e. three times), patients will be reimbursed £15 for their time and effort. Patients who complete an interview will also be reimbursed with a £20 shopping voucher. Reasonable travel expenses for any visits additional to normal care will be reimbursed on production of receipts, or a mileage allowance provided as appropriate.

Patients who take part in the focus groups or interview on wards will be reimbursed with a £15 shopping voucher.

17.7. Ethical Considerations

We anticipate few ethical concerns for patients entering this study.

© Copyright: The University of Oxford and Oxford University Hospitals NHS Foundation Trust 2016

Participation in the trial does not change existing treatment receipt. However half of the patients will receive an additional intervention: the VR treatment. VR has been used with patients with psychosis but this automated six session VR treatment has not been tested before, and not with this broad patient group, so clinical efficacy remains to be determined i.e. clinical equipoise exists. Half of the patients will not receive the additional intervention. The overall aim of the gameChange project is to implement VR in NHS services if it is shown to be effective, with the trial sites being lead services, so that if the project is successful it should mean that the VR treatment can become available at a later date to those people in the control arm.

We also anticipate mild and transient levels of anxiety to occur in trial patients. In the behavioural assessment task we will be asking patients to enter the kinds of everyday environments that make them anxious (e.g. a local shop). The assessment makes sure that people only start in an environment that they are happy to enter (perhaps causing 4-5 out of 10 for anxiety) and they can stop whenever they wish. Also in VR we will be asking half of the patients to enter virtual recreations of the types of environments that can make them anxious (e.g. going to a café). These are everyday environments that they are encountering routinely and that they would like to feel less anxious in. The VR environments will feel safer because the patient will know they are computer generated.

The other main ethical issue is the burden of the assessments for the participants. These typically take around 90 minutes. However, we have successfully used these assessments before (indeed have used much longer assessments in trials). It is generally a patient group who have limited social contact, who often have few activities during the day, and who appreciate the time spent with our staff. Hence in our clinical trials there is always improvement in the control condition even when that just comprises the additional monitoring. Patients can take breaks and also complete the assessments over several meetings. Nevertheless, if a patient does find the assessments too long then the battery can always be shortened to the primary measure. However, our data completion rates are typically very high, as are our follow-up rates, indicating that patients are fully informed about what the trial will involve. The LEAP have reviewed the trial assessments.

17.8. Other Considerations

Safety of researchers is very important; therefore, we follow a standard operating procedure for lone working.

Where one-to-one interviews occur as part of the qualitative study on wards, the safety of the researcher will be ensured through following ward procedure for carrying a pit alarm.

For details on safety of the equipment, see section 9.2 above.

As noted above, a potential conflict of interest exists due to the CI and Sponsor’s involvement in a Spin out company, to which the treatment will be licensed in the future. There is a conflict of interest plan in place for the CI in the Department of Psychiatry.

18. FINANCE AND INSURANCE

18.1. Funding

© Copyright: The University of Oxford and Oxford University Hospitals NHS Foundation Trust 2016

The trial is funded by the NHS National Institute of Health Research (NIHR) invention for innovation (i4i) programme. It is also supported by the NIHR Oxford Health Biomedical Research Centre.

18.2. Insurance

The University has a specialist insurance policy in place which would operate in the event of any participant suffering harm as a result of their involvement in the research (Newline Underwriting Management Ltd, at Lloyd's of London). NHS indemnity operates in respect of the clinical treatment that is provided.

18.3. Contractual arrangements

The appropriate contractual arrangements will be put in place with all collaborating organisations. Financial contracts will be drafted and arranged by Oxford Health NHS Foundation Trust. The University of Oxford will draft and arrange model Non-Commercial Agreements (mNCA) with all trial sites.

18.4. Generation of intellectual property

A separate intellectual property agreement is in place from Oxford Health NHS Foundation Trust and relevant parties.

19. PUBLICATION POLICY

The results of the trial will be published in a journal.

© Copyright: The University of Oxford and Oxford University Hospitals NHS Foundation Trust 2016

20. GANTT CHART

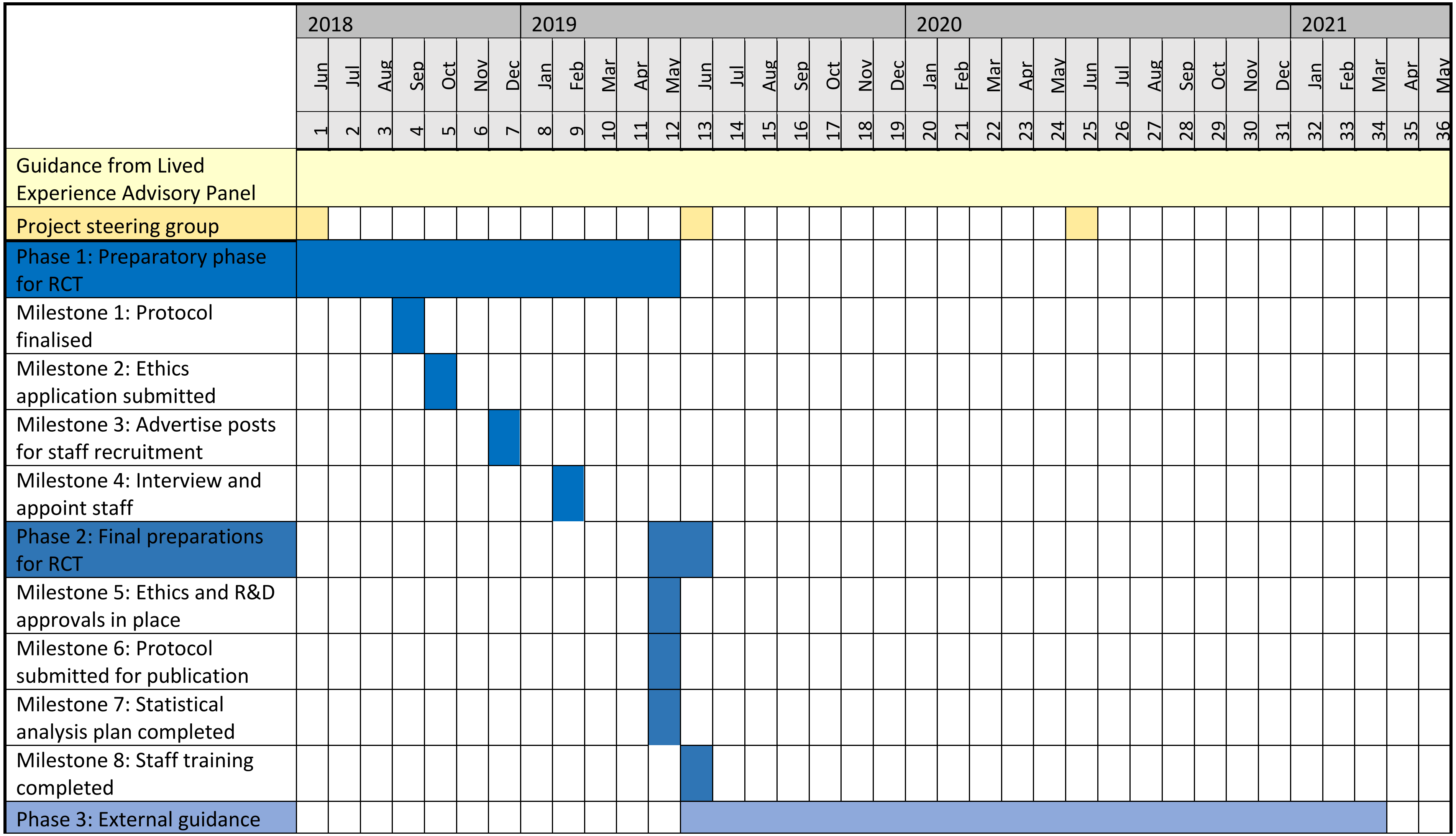

© Copyright: The University of Oxford and Oxford University Hospitals NHS Foundation Trust 2016

Milestone 9: First DMEC meeting																																				
Milestone 10: Ongoing DMEC meetings (frequency to be confirmed by DMEC)																																				
Milestone 11: Final DMEC meeting																																				
Phase 4: Participant recruitment (and baseline assessment)																																				
Milestone 12: Participant recruitment commences (5 sites)																																				
Milestone 13: 100 participants recruited																																				
Milestone 14: 200 participants recruited																																				
Milestone 15: 300 participants recruited																																				
Milestone 16: Recruitment completed (432 participants)																																				
Phase 5: Trial intervention delivered																																				
Milestone 17: Delivery of trial therapy commences (5 sites)																																				
Milestone 18: Treatment adherence and competence checks started																																				
Milestone 19: Delivery of trial therapy completed																																				
Milestone 20: Treatment adherence and competence checks completed																																				

© Copyright: The University of Oxford and Oxford University Hospitals NHS Foundation Trust 2016

Phase 6: Participant follow-up assessment
Milestone 21: First 6 weeks assessments completed
Milestone 22: First 26 weeks assessments completed
Milestone 23: Final 6 weeks assessments completed
Milestone 24: Final 26 weeks assessments completed
Phase 7: Database completion
Milestone 25: Data cleaning (baseline, 6 weeks and 26 weeks assessments)
Milestone 26: Databases completed
Phase 8: Analysis and write-up
Milestone 27: Data analysis completed
Milestone 28: Draft final report

© Copyright: The University of Oxford and Oxford University Hospitals NHS Foundation Trust 2016

21. REFERENCES

Attkisson, C. C., & Zwick, R. (1982). The client satisfaction questionnaire. Evaluation and Program Planning, 5, 233–237. https://doi.org/10.1155/2013/427827

Beck, A., & Steer, R. (1988). BHS, Beck Hopelessness Scale: Manual.

Beecham, J., & Knapp, M. (1999). Costing psychiatric interventions. In G. Thornicroft, C. Brewin, & J. Wing (Eds.), Measuring Mental Health Needs (2nd ed.). London.

Bryant, F. (2003). Savoring Beliefs Inventory (SBI): A scale for measuring beliefs about savouring. *Journal of mental health*, *12*(2), 175-196.

Carl, E., Stein, A. T., Levihn-Coon, A., Pogue, J. R., Rothbaum, B., Emmelkamp, P., Asmundson, G. J. G., Carlbring, P., & Powers, M. B. (2018). Virtual reality exposure therapy for anxiety and related disorders: A meta- analysis of randomized controlled trials. Journal of Anxiety Disorders. https://doi.org/10.1002/da.22728

Cella, M., Edwards, C., & Wykes, T. (2016). A question of time: A study of time use in people with schizophrenia. Schizophrenia Research, 176(2–3), 480–484.

Chadwick, P., Hember, M., Symes, J., Peters, E., Kuipers, E., & Dagnan, D. (2008). Responding mindfully to unpleasant thoughts and images: reliability and validity of the Southampton mindfulness questionnaire (SMQ). *British Journal of Clinical Psychology*, *47*(4), 451-455.

Chambless, D. L., Caputo, G. C., Jasin, S. E., Gracely, E. J., & Williams, C. (1985). The Mobility Inventory for Agoraphobia. Behaviour Research and Therapy, 23(1), 35–44. https://doi.org/10.1016/0005-7967(85)90140-8

Clark, D. M. (1999). Anxiety disorders: why they persist and how to treat them. Behaviour Research and Therapy, 37, S5–S27. https://doi.org/10.1016/S0005-7967(99)00048-0

Connor, K. M., Davidson, J. R. T., Churchill, L. E., Sherwood, A. W., Foa, E., & Weisler, R. H. (2000). Psychometric properties of the Social Phobia Inventory (SPIN). British Journal of Psychiatry, 176, 379–386.

EuroQol Group. (2009). EQ-5D-5L Health Questionnaire: English version for the UK.

Finch, T.L., Girling, M., May, C.R., Mair, F.S., Murray, E., Treweek, S., Steen, I.N., McColl, E.M., Dickinson, C., Rapley, T. (2015). NoMAD: Implementation measure based on Normalization Process Theory. [Measurement instrument]. Retrieved from http://www.normalizationprocess.org

Fowler, D., Freeman, D., Smith, B. E. N., Kuipers, E., Bebbington, P., Bashforth, H., ... & Garety, P. (2006). The Brief Core Schema Scales (BCSS): psychometric properties and associations with paranoia and grandiosity in non-clinical and psychosis samples. Psychological medicine, 36(6), 749-759.

Freeman, D., Bird, J., Loe, B., Kingdon, D., Startup, H., Clark, D., Ehlers, A., Černis, E., Wingham, G., Evans, N., Lister, R., Pugh, K., Cordwell, J., & Dunn, G. (2019). The Dunn Worry Questionnaire and the Paranoia

© Copyright: The University of Oxford and Oxford University Hospitals NHS Foundation Trust 2016

Worries Questionnaire: new assessments of worry. Psychological Medicine. doi: 10.1017/S0033291719000588.

Freeman, D., Loe, B., Kingdon, D., Startup, H., Molodynski, A., Rosebrock, L., Brown, P., Sheaves, B., Waite, F., Bird, J. (2019). The revised Green et al., Paranoid Thoughts Scale (R-GPTS): Psychometric properties, severity ranges, and clinical cut-offs. Psychological Medicine, 1-10. doi:10.1017/S0033291719003155

Freeman, D., Bradley, J., Antley, A., Bourke, E., DeWeever, N., Evans, N., Černis, E., Sheaves, B., Waite, F., Dunn, G., Slater, M., & Clark, D. M. (2016). Virtual reality in the treatment of persecutory delusions: Randomised controlled experimental study testing how to reduce delusional conviction. British Journal of Psychiatry, 209(1), 62–67. https://doi.org/10.1192/bjp.bp.115.176438

Freeman, D., Garety, P. A., & Kuipers, E. (2001). Persecutory delusions: developing the understanding of belief maintenance and emotional distress. Psychological Medicine, 31(7), 1293–1306. https://doi.org/10.1017/S003329170100455X

Freeman, D., Haselton, P., Freeman, J., Spanlang, B., Kishore, S., Albery, E., Denne, M., Brown, P., Slater, M., & Nickless, A. (2018). Automated psychological therapy using immersive virtual reality for treatment of fear of heights: a single-blind, parallel-group, randomised controlled trial. The Lancet Psychiatry, 5(8), 625–632. https://doi.org/10.1016/S2215-0366(18)30226-8

Freeman, D., Lister, R., Waite, F., Yu, L-M., Slater, M., Dunn, G., Clark, D. (2019). Automated psychological therapy using virtual reality (VR) for patients with persecutory delusions: study protocol for a single-blind parallel group randomised controlled trial (THRIVE). Trials, 20: 87.

Freeman, D., Pugh, K., Vorontsova, N., Antley, A., & Slater, M. (2010). Testing the Continuum of Delusional Beliefs: An Experimental Study Using Virtual Reality. Journal of Abnormal Psychology, 119(1), 83–92. https://doi.org/10.1037/a0017514

Freeman, D., Reeve, S., Robinson, A., Ehlers, A., Clark, D., Spanlang, B., & Slater, M. (2017). Virtual reality in the assessment, understanding, and treatment of mental health disorders. Psychological Medicine, 47(14), 2393–2400. https://doi.org/10.1017/S003329171700040X

Freeman, D., Taylor, K., Molodynski, A., & Waite, F. (submitted). Treatable clinical intervention targets for patients with schizophrenia.

Gilbert, P., Clarke, M., Hempel, S., Miles, J. N. V., & Irons, C. (2004). Criticizing and reassuring oneself: An exploration of forms, styles and reasons in female students. British Journal of Clinical Psychology, 43, 31– 50. https://doi.org/10.1348/014466504772812959

Green, C. E. L., Freeman, D., Kuipers, E., Bebbington, P., Fowler, D., Dunn, G., & Garety, P. A. (2008). Measuring ideas of persecution and social reference: The Green et al. Paranoid Thought Scales (GPTS). Psychological Medicine, 38(1), 101–111. https://doi.org/10.1017/S0033291707001638

Hjorthøj, C., Stürup, A. E., McGrath, J. J., & Nordentoft, M. (2017). Years of potential life lost and life expectancy in schizophrenia: a systematic review and meta-analysis. The Lancet Psychiatry, 4(4), 295–301. https://doi.org/10.1016/S2215-0366(17)30078-0

© Copyright: The University of Oxford and Oxford University Hospitals NHS Foundation Trust 2016

Jolley, S., Garety, P. A., Ellett, L., Kuipers, E., Freeman, D., Bebbington, P. E., Fowler, D. G., & Dunn, G. (2006). A validation of a new measure of activity in psychosis. Schizophrenia Research, 85(1–3), 288–295. https://doi.org/10.1016/j.schres.2006.03.012

Keetharuth, A., Brazier, J., Connell, J., Bjorner, J., Carlton, J., Taylor Buck, E., Ricketts, T., McKendrick, K., Browne, J., Croudace, T., & Barkham, M. (2018). Recovering Quality of Life (ReQoL): a new generic self-reported outcome measure for use with people experiencing mental health difficulties. The British Journal of Psychiatry, 212(1).

Kroenke, K., Spitzer, R., & Williams, J. (2001). The PHQ-9. Journal of General Internal Medicine, 16, 606-613.

Lindamer, L. A., McKibbin, C., Norman, G. J., Jordan, L., Harrison, K., Abeyesinhe, S., & Patrick, K. (2008). Assessment of physical activity in middle-aged and older adults with schizophrenia. Schizophrenia Research, 104(1–3), 294–301. https://doi.org/10.1016/j.schres.2008.04.040

May, C. R., Mair, F., Finch, T., MacFarlane, A., Dowrick, C., Treweek, S., Rapley, T., Ballini, L., Ong, B. N., Rogers, A., Murray, E., Elwyn, G., Légaré, F., Gunn, J., & Montori, V. M. (2009). Development of a theory of implementation and integration: Normalization Process Theory. Implementation Science : IS, 4, 29. https://doi.org/10.1186/1748-5908-4-29

Mayo-Wilson, E., Dias, S., Mavranezouli, I., Kew, K., Clark, D. M., Ades, A. E., & Pilling, S. (2014). Psychological and pharmacological interventions for social anxiety disorder in adults: A systematic review and network meta-analysis. The Lancet Psychiatry, 1(5), 368–376. https://doi.org/10.1016/S2215-0366(14)70329-3

Mendelson, B. K., Mendelson, M. J., & White, D. R. (2001). Body-esteem scale for adolescents and adults. Journal of Personality Assessment, 76, 90–106. https://doi.org/10.1207/S15327752JPA7601

Morina, N., Ijntema, H., Meyerbröker, K., & Emmelkamp, P. M. G. (2015). Can virtual reality exposure therapy gains be generalized to real-life? A meta-analysis of studies applying behavioral assessments. Behaviour Research and Therapy, 74, 18–24. https://doi.org/10.1016/j.brat.2015.08.010

Neil, S. T., Kilbride, M., Pitt, L., Nothard, S., Welford, M., Sellwood, W., & Morrison, A. P. (2009). The questionnaire about the process of recovery (QPR): A measurement tool developed in collaboration with service users. Psychosis, 1(2), 145–155. https://doi.org/10.1080/17522430902913450

Posner K, Brent D, Lucas C, Gould M, Stanley B, Brown G, et al. Columbia-Suicide Severity Rating Scale (C-SSRS) Self Report Version 6/30/10. The Research Foundation for Mental Hygiene, Inc. New York; 2008.

Posner, K., Brown, G. K., Stanley, B., Brent, D. A., Yershova, K. V, Oquendo, M. A., Currier, G. W., Melvin, G. A., Greenhill, L., Shen, S., & Mann, J. J. (2011). The Columbia–Suicide Severity Rating Scale: Initial Validity and Internal Consistency Findings From three Multisite Studies With Adolescents and Adults. American Journal of Psychiatry, 168, 1266–1277. Retrieved from www.cssrs.columbia.edu

Pot-Kolder, R. M. C. A., Geraets, C. N. W., Veling, W., van Beilen, M., Staring, A. B. P., Gijsman, H. J., Delespaul, P. A. E. G., & van der Gaag, M. (2018). Virtual-reality-based cognitive behavioural therapy versus waiting list control for paranoid ideation and social avoidance in patients with psychotic disorders:

© Copyright: The University of Oxford and Oxford University Hospitals NHS Foundation Trust 2016

a single-blind randomised controlled trial. The Lancet Psychiatry, 5(3), 217–226. https://doi.org/10.1016/S2215-0366(18)30053-1

Rosebrock, L., Cernis, E., Lambe, S., Waite, F., Rek, S., Petit, A., Ehlers, A., Clark, D. M., Freeman, D. (2021). Catastrophic Cognitions about Coronavirus: The Oxford Psychological Investigation of Coronavirus Questionnaire [TOPIC-Q]. Psychological Medicine, 1-34. doi:10.1017/S0033291721000283

Rothbaum, B. O., Hodges, L. F., Kooper, R., Opdyke, D., Williford, J. S., & North, M. (1995). Virtual reality graded exposure in the treatment of acrophobia: A case report. Behavior Therapy. Vol 26(3), 26, 547–554. https://doi.org/10.1016/S0005-7894(05)80100-5

Schulz, K. F., Altman, D. G., & Moher, D. (2010). CONSORT 2010 Statement: updated guidelines for reporting parallel group randomised trials. BMC Medicine, 8(1), 18. https://doi.org/10.1186/1741-7015-8-18

The National Clinical Audit of Psychosis Project Team. (2018). National Clinical Audit of Psychosis. Royal College of Psychiatrists. London.

Upthegrove, R., Marwaha, S., & Birchwood, M. (2017). Depression and Schizophrenia: Cause, Consequence, or Trans-diagnostic Issue? Schizophrenia Bulletin, 43(2), 240–244. https://doi.org/10.1093/schbul/sbw097

© Copyright: The University of Oxford and Oxford University Hospitals NHS Foundation Trust 2016

22. APPENDIX A: SCHEDULE OF PROCEDURES

Procedures	Screening	Baseline (0 weeks)	VR session 1*	VR session 2*	VR session 3*	VR session 4*	VR session 5*	VR session 6*	Post-therapy (6-16 weeks)	Follow-up (20-36 weeks)
Eligibility assessment	X	X (brief)								
Informed consent		X								
Demographics		X								
Randomisation		X								
O-AS		X							X	X
Activity (actigraphy, time-budget)		X							X	X
R-GPTS		X							X	X
Columbia scale		X							X	X
EQ-5D-5L		X							X	X
ReQol		X							X	X
AMI-A		X							X	X
PHQ-9		X							X	X
PWQ		X							X	X
Mediators (e.g. safety beliefs)		X							X	X
Moderators (e.g. voices, hopelessness, body-esteem)		X								
Exploratory (coronavirus cognitions)		X							X	X
Exploratory (mindfulness, self-concept, anhedonia, self-criticism and self-reassurance)										X
QPR		X							X	X
Service receipt		X							X	X
VR treatment*			X	X	X	X	X	X		
VR negative effects scale, satisfaction								X		
Adverse event monitoring	X	X	X	X	X	X	X	X	X	X
Treatment as usual	X	X	X	X	X	X	X	X	X	X

In addition to close contact with clinical teams and reporting of adverse events when they are brought to the attention of the team as required, medical notes will be checked at the end of the trial to assess for adverse events and check service receipt. *Only participants allocated to receive virtual reality therapy will attend VR sessions.

23. APPENDIX B: AMENDMENT HISTORY

Amendment No.	Protocol Version No.	Date issued	Author(s) of changes	Details of Changes made
1	2.1	17.05.2019	Daniel Freeman	Removal of measures:<br>- DASS<br>- SBQ;<br>- SBQ self-report;<br>- CAPS-hallucinations;<br>- SPIN;<br>- IAPT digital health PEQ.<br>Addition of measures:<br>- PHQ-9Cognition and Defence Behaviours Questionnaire;<br>- SR-OBAT;<br>- Hallucinations scale<br>- .<br>Revision of measure:<br>- GPTS to R-GPTS<br>- Revised therapy satisfaction scale<br><br>Insertion that software will be CE marked before use in the trial.
2	3	06.06.2019	Daniel Freeman	Amendment to details of qualitative sub-study: additional focus groups to be held on inpatient wards.
3	4	26.07.2020	Daniel Freeman	Addition of measures:<br>- SMQ<br>- BCSS<br>- SBI

				Insertion of time frame for completion of follow-ups.<br><br>Addition of new project-coordinator (Ariane Petit).<br><br>Reference to self-report C-SSRS.<br><br>Coronavirus (COVID-19) changes:<br><br>- Suspension of administration of O-BAT and change of primary outcome measure to Self-report O-BAT. Change of O-BAT to secondary outcome measure.<br>- Addition of TOPIC Q.<br>- Temporary suspension of recruitment of people at moderate or high risk for a severe course of COVID-19.<br>- Coronavirus addendum to PIS.<br>- Remote assessments.<br>- Enhanced cleaning and hygiene procedures for using the VR equipment.<br>- Data confidentiality procedures whilst working remotely.<br>- Updates to Gantt chart.
4	4.3	10.12.2020	Daniel Freeman, Ariane Petit	We have updated the number of interviews with service users who received the VR and the details of the implementation and sustainability studies.
5	4.5	XX.XX.XXX	Daniel Freeman, Ariane Petit	Addition of measure: FSCRS.<br><br>Coronavirus (COVID-19) change: people at moderate or high risk for a severe course of COVID-19 can take part if vaccinated.<br><br>Minor corrections and clarifications to Synopsis, Summary of Objectives and Outcome Measures and Appendix A: Schedule of Procedures.

Protocol amendments must be submitted to the Sponsor for approval prior to submission to the REC committee, and Health Research Authority.

Primary Care
Clinical Trials Unit

STATISTICAL ANALYSIS PLAN

gameChange
Improving lives through VR therapy

The gameChange Trial: A randomised controlled trial testing automated virtual reality cognitive therapy for patients with fears in everyday situations.

Version 1.0, 2 November 2021

Based on version 4.6 of Protocol (24 June 2021)

	NAME	TITLE	Signature	Date
Written by:	Ushma Galal	Trial Statistician		02/11/2021
Reviewed by:	Nicola Williams	Senior Trial Statistician		02/11/2021
Approved by:	Daniel Freeman	Chief Investigator		02/11/2021

Version History

Version:	Version Date:	Changes:
0.1	23 April 2019	Original
0.2-0.4		Updated with comments
0.5	1 April 2021	Updated after major protocol amendment in which Primary outcome was changed
0.6	13 May 2021	Updated after review by NW, including update to Avoidance score derivation and analysis. Addition of sensitivity analysis for shortened 26-week follow-up visit

0.7	29 June 2021	Updated after review by NW and according to updated protocol
0.8	1 September 2021	Updated after review by trial team
0.9	16 September 2021	Updated with comment from VH regarding mediation analysis
0.10	29 September 2021	Updated after review by NW, MS (CACE) & trial team
0.11	5 October 2021	Update after comments from DF on CACE analysis and SL on O-AS definition
0.12	15 October 2021	Updated after query discussion
0.13	02 Nov 2021	Final cleaned up draft version

Table of Contents

1 Introduction ... 56
1.1 Preface ... 56
1.2 Purpose and scope of the plan ... 56
1.3 Trial overview ... 56
1.4 Objectives ... 57
2 Trial design ... 59
2.1 Outcome measures ... 60
2.1.1 Primary outcome ... 60
2.1.2 Secondary outcomes ... 60
2.2 Target population ... 62
2.2.1 INCLUSION CRITERIA ... 62
2.2.2 EXCLUSION CRITERIA ... 62
2.3 Sample size ... 63
2.4 Randomisation and blinding in the analysis stage ... 63
3 Analysis – General considerations ... 64
3.1 Descriptive statistics ... 64
3.2 Baseline characteristics of participants ... 64
3.3 Definition of population for analysis ... 67
3.4 Pooling of investigational sites ... 67
3.5 Data Monitoring Committee And Interim Analyses ... 67
4 Derivation of variables ... 68
4.1 Primary outcome - Oxford Agoraphobic Avoidance Scale (O-AS) ... 68
4.2 Secondary outcomes ... 69
4.2.1 Actigraphy ... 69
4.2.2 Time Budget ... 69
4.2.3 Agoraphobia Mobility Inventory-Avoidance [AMI-A] ... 69
4.2.4 Oxford Behavioural Avoidance Task [O-BAT] ... 69
4.2.5 Revised-Green et al Paranoid Thoughts Scale [R-GPTS] ... 70
4.2.6 Paranoia Worries Questionnaire [PWQ] ... 70
4.2.7 Patient Health Questionnaire [PHQ-9] ... 70
4.2.8 Columbia Suicide Severity Rating Scale [C-SSRS] ... 70
4.2.9 EQ-5D-5L - index and the VAS ... 71
4.2.10 Recovering quality of life [ReQol-20] ... 71
4.2.11 Questionnaire on progress of recovery [QPR] ... 72
4.2.12 Oxford Cognitions and defences Questionnaire [O-CDBQ] ... 72
4.2.13 Visual Analog Scales of safety ... 72

4.2.14 Hallucinations scale 72
4.2.15 Beck Hopelessness Scale (Beck, 1988) [BHS] 73
4.2.16 Body-esteem Scale for Adolescents and Adults (Mendelson et al, 2001) 73
4.2.17 Oxford Cognitions and defences questionnaire [O-CDBQ] 73
4.3 Sensitivity analysis 73
4.3.1 CACE analysis 73
5 PRIMARY ANALYSIS 74
5.1 Primary outcome 74
5.2 Handling missing data 74
5.3 Handling outliers 74
5.4 Handling multi-centre/clustered data 74
5.5 Multiple comparisons and multiplicity 75
5.6 Model Assumptions 75
6 SECONDARY ANALYSIS 75
6.1 Secondary outcomes 75
6.1.1 Continuous secondary outcomes 75
6.1.2 Binary secondary outcomes 78
6.1.3 Mediation Analysis 78
6.1.4 Moderation/Subgroup Analysis 79
7 ADDITIONAL EXPLORATORY ANALYSIS NOT SPECIFIED IN THE PROTOCOL 79
7.1 Medications 79
7.2 Service use 80
8 SENSITIVITY ANALYSIS 84
8.1 Outliers and missingness assumptions 84
8.2 Complier Average Causal Effect (CACE) analysis 84
8.3 Impact of the COVID-19 pandemic 85
8.4 Shortened follow-up 85
8.5 Medication effects 85
9 SAFETY ANALYSIS 86
10 VALIDATION 86
11 CHANGES TO THE PROTOCOL OR PREVIOUS VERSIONS OF SAP 86
12 References 87
13 Appendices 88
13.1 Appendix I. Schedule of procedures 88
13.2 Appendix II. Flow diagram of trial participants 90

1 Introduction

1.1 Preface

Chief Investigator: Professor Daniel Freeman

Trial Statisticians: Ly-Mee Yu and Ushma Galal

Data Manager: Jenna Grabey

This SAP supports protocol version 4.6 date: 24.6.2021

1.2 Purpose and scope of the plan

This document details the proposed analyses of primary and secondary objectives for the gameChange study, funded by the NHS National Institute of Health Research (NIHR) i4i programme and the NIHR Oxford Health Biomedical Research Centre. Subsequent analyses of a more exploratory nature will not be bound by this strategy, though they are expected to follow the broad principles laid down here. The principles are not intended to curtail exploratory analysis nor to prohibit accepted practices, but they are intended to establish the rules that will be followed, as closely as possible, when analysing and reporting the trial. All example tables included in the plan are intended to aid the presentation of data at final analysis. However, the statistician should not be bound by these tables and is free to present the results in a suitable way.

The statistical analysis plan will be available on request when the principal papers are submitted for publication in a journal. Suggestions for subsequent analyses by the journal editors or referees will be considered carefully, and carried out as far as possible in line with the principles of the analysis strategy; if reported, the source of the suggestion will be acknowledged.

Any deviations from the statistical analysis plan will be described and justified in the final report of the trial.

1.3 Trial overview

Too many patients with psychosis, despite standard treatment, become isolated and inactive, with negative effects on both mental and physical health. Approximately 80% of patients with schizophrenia experience an episode of depression (Upthegrove et al, 2017). Physical activity levels in patients with schizophrenia are reduced on average by approximately two thirds (Lindamer et al, 2008). Over 90% of patients with schizophrenia are unemployed and spend "less time in functional but also in social and leisure activities and more time resting and 'doing nothing' compared to the general population" (Cella et al, 2016). Life expectancy is on average 14.5 years shorter (Hjorthøj et al, 2017), due to largely preventable conditions such as high blood pressure, diabetes, and heart disease. Partly this physical ill health reflects unhealthy lifestyles including inactivity. Our view is that a substantial part of this inactivity arises from avoidance due to anxiety. In a clinical assessment study of 1800 patients with non-affective psychosis, two-thirds of the patients had levels of anxious avoidance equivalent to patients diagnosed with agoraphobia (Freeman et al, 2019). The anxiety in patients with psychosis can arise from a number of sources: fears that others will harm them, voices telling them of danger, social anxiety fears of humiliation and rejection, and negative beliefs about the self that cause a lack of confidence and a sense of vulnerability.

Virtual reality (interactive computer-generated environments) has been used since the early 1990's to treat anxiety (Rothbaum al, 1994). Meta-analyses indicate that VR treatments for anxiety disorders can produce large treatment effects (Carl et al, 2018) that generalise to the real world (Morina et al, 2015). Previous uses of VR for mental health

problems have all depended on a therapist providing the psychological therapy (Freeman, Reeve et al, 2017). Furthermore, automated treatment has the potential to be scalable, removing a key cause of the highly limited access to psychological therapy for patients with psychosis. VR may also be especially suited to the difficulties of patients with psychosis. Patients with strong fears are much more likely to test out their fear expectations in VR because they know it is a simulation but the learning that they make then transfers to the real world (Morina et al, 2015). VR treatment can also include engaging tasks that make the treatment experience much more pleasurable. A graded approach can easily be applied in VR, allowing the individual to repeatedly experience the situations they find difficult and make new learning. The view is that VR treatments have the potential to be faster, more efficacious, and appealing to patients than traditional face-to-face approaches.

The chief investigator and colleagues have developed - using a socially-inclusive design process - a new automated VR cognitive treatment for patients with psychosis having difficulties being in everyday social situations. It is designed to be easy to use, engaging for patients and staff, and delivered with the latest consumer equipment. Therefore this VR treatment has the potential to be widely implemented in treatment services. Psychological treatment that involves direct coaching in the situations that trouble patients with psychosis is rarely available in mental health services. This study sets out to determine the clinical effects of the VR treatment on real-world performance, activity levels, psychiatric symptoms, and quality of life.

1.4 Objectives

The primary research question is: Does automated VR cognitive treatment added to treatment as usual, compared to treatment as usual alone, lead to a post-treatment reduction in real world distress and avoidance for patients with psychosis attending NHS mental health services?

The primary hypothesis is that:

1. Compared to treatment as usual, VR cognitive therapy added to treatment as usual will reduce distress and avoidance of real world situations (post treatment).

The secondary hypotheses are:

1. Compared to treatment as usual, VR cognitive therapy added to treatment as usual will reduce psychiatric symptoms (paranoia, anxious avoidance, depression, and suicidal ideation), increase activity, and improve quality of life (post-treatment).

2. Treatment effects will be maintained at follow-up.

3. The mediators of VR treatment will be safety beliefs, threat cognitions, and defence behaviours.

4. Treatment effects will be moderated by the occurrence of negative auditory hallucinations in social situations, hopelessness, appearance concerns, and threat cognitions.

There will also be a health economic evaluation of the VR treatment and a qualitative sub-study, which will be reported elsewhere.

Objectives	Outcome Measures	Time-points of evaluation of this outcome measure
**Primary objective:**<br><br>1. VR treatment leads to reduction in distress and avoidance of everyday situations.	The primary outcome will be the O-AS (Lambe at al., 2021).	Weeks 0, 6, and 26.
**Secondary objectives:**<br><br>1. Test clinical improvements by treatment type in activity levels, psychiatric symptoms, quality of life.	1. **Activity levels**: Actigraphy, time-budget measure (Jolley et al, 2006).<br><br>**Psychiatric symptoms**: Agoraphobia Mobility Inventory-Avoidance (Chambless et al., 1985), O-BAT (Freeman et al., 2016), Revised-Green et al Paranoid Thoughts Scale (Green et al., 2008; Freeman et al., 2019); Paranoia Worries Questionnaire (Freeman et al, 2019), PHQ-9 (Kroenke et al, 2001), Columbia Suicide Severity Rating Scale (Posner et al., 2011, Posner et al., 2008).<br><br>**Quality of life**: EQ-5D-5L (http://www.euroqol.org/), ReQol (Keetharuth et al, 2018), Questionnaire on the Progress of Recovery (Neil et al., 2009).	1. Weeks 0, 6, and 26.
2. Determine the cost-effectiveness of the virtual reality treatment.*	2. Client Service Receipt Inventory (Beecham & Knapp, 1992.	2. Weeks 0, 6, and 26.
3. Test mediation of treatment effects by changes in safety beliefs, threat cognitions (vulnerability and	3. Oxford Cognitions and Defences Questionnaire (O-CDQ) (Rosebrock et al, submitted) and strength of safety beliefs,	3. Weeks 0, 6, and 26.

Objectives	Outcome Measures	Time-points of evaluation of this outcome measure
threat anticipation), and defence behaviours.	vulnerability belief, and threat anticipation assessed using three visual analogue scales (Freeman et al., 2016).	
4. Test moderation of treatment effects (negative auditory hallucinations, hopelessness, appearance concerns, and threat cognition).	4. Moderator variables assessed at 0 weeks: Hallucinations scale; Beck Hopelessness Scale (Beck, 1988); Body-esteem Scale for Adolescents and Adults (Mendelson et al, 2001); Oxford Cognitions and Defences Questionnaire (Rosebrock et al, submitted). These are tested for the moderation of the primary outcome at 6 weeks.	4. Week 6.
**Tertiary objectives‡:**<br>1a. To carry out a qualitative study of the experience of the virtual reality therapy<br>1b. To explore and compare the challenges of implementing VR therapy in clinical and home settings.<br>1c. To assess the feasibility of implementing VR therapy into NHS mental health services.<br>2. Assess patient satisfaction with the VR therapy.<br>3. Examine the value proposition of virtual reality treatment for psychosis within the NHS post-trial through exploration of stakeholder priorities.	1a. Semi-structured interview.<br>1c. NoMAD questionnaire (Finch et al, 2015).<br>2. Revised therapy satisfaction scale.<br>3. Secondary analysis of data collected during the trial and interviews with key stakeholders Questionnaires and interviews with key stakeholders.	6-10 weeks.<br>6 weeks.<br>Throughout.

ˣ These objectives/outcomes are part of the health economics analysis and will thus be carried out separately and reported elsewhere.

‡ All the tertiary objectives are part of the qualitative analysis and will thus be analyses separately and reported elsewhere.

2 Trial design

The design is a multicentre, parallel group randomised controlled trial with single blind assessment to test whether the automated VR cognitive treatment added to treatment as usual, compared to treatment as usual alone, leads to a post-treatment reduction in real world distress and avoidance for patients with psychosis attending NHS mental

health services. Treatment as usual will be measured but remain as usual in both groups. Assessments will be carried out at 0 (baseline), 6 (post treatment), and 26 (follow-up) weeks by a researcher blind to treatment allocation.

Pre-COVID period: For all outcomes, the 6 week and 26 week assessments can be conducted up to 10 weeks after the assessment due date, i.e. up to 6+10 weeks post-randomisation for the 6 week assessments and 26+10 weeks for the 26 week assessments (although the aim is to conduct these assessments at 6 and 26 weeks). If it is not possible to complete the follow-up assessments within these windows, it will be considered missing data.

Shortened follow-up during the COVID-19 pandemic: In addition to the above, for participants who were randomised in the last four months of recruitment, the 26 week assessments will be brought forward by up to 6 weeks i.e. they will be conducted from 20 weeks post-randomisation. This is to ensure data collection is completed within the study schedule.

2.1 Outcome measures

2.1.1 Primary outcome

The primary outcome measure will be by means of the Oxford Agoraphobic Avoidance Scale (O-AS) (Lambe et al., 2021) which is assessed at weeks 0, 6 and 26. The primary endpoint for analysis is 6 weeks.

2.1.2 Secondary outcomes

1. Clinical improvements in activity levels, psychiatric symptoms and quality of life will be assessed by the following at 0, 6, and 26 weeks:
   - Activity levels: assessed by Actigraphy and a time-budget measure (Jolley et al., 2006).
   - Psychiatric symptoms: assessed by the:
     a. Agoraphobia Mobility Inventory-Avoidance [AMI-A] (Chambless et al., 1985),
     b. Real-world avoidance and distress [O-BAT] (Freeman et al., 2016),
     c. Revised-Green et al Paranoid Thoughts Scale [R-GPTS] (Green et al., 2008; Freeman et al, 2019);
     d. Paranoia Worries Questionnaire [PWQ] (Freeman et al, 2019),
     e. Patient Health Questionnaire [PHQ-9] (Kroenke et al, 2001),
     f. Columbia Suicide Severity Rating Scale [C-SSRS] (Posner et al., 2011).
2. Quality of life will be assessed using the following tools:
   a. EQ-5D-5L (http://www.euroqol.org/). The EQ-5D-5L index will be calculated using the cross walk method. This index and the VAS score will be summarised and compared between groups,
   b. ReQol,
   c. Progress of recovery [QPR].
3. The following Mediation variables will be assessed at weeks 0, 6 & 26:
   a. Cognition and use of defence behaviours will be assessed using Parts 1 (cognitions) and 3 (defences) of the Oxford Cognitions and Defences Questionnaire [O-CDBQ] (Rosebrock et al, submitted),
   b. Strength of safety beliefs, vulnerability belief, and threat anticipation assessed using three visual analogue scales (Freeman et al., 2016).
4. The following Moderator variables will be assessed at 0 weeks and tested for the moderation of the primary outcome at 6 weeks:
   a. Hallucinations Scale ,
   b. Beck Hopelessness Scale [BHS] (Beck, 1988),
   c. Body-esteem Scale for Adolescents and Adults (Mendelson et al, 2001),
   d. Part 1 of the Oxford Cognitions and Defences Questionnaire [O-CDBQ] (Rosebrock et al, submitted).

2.2 Target population

The trial participants will be the patients with psychosis and self-reported difficulties going outside among other people primarily due to anxiety (assessed using a screening version of the Oxford – Behavioural Assessment Task (O-BAT) self-report questionnaire).

2.2.1 Inclusion Criteria

- Participant is willing and able to give informed consent for participation in the trial.
- Aged 16 years or above.
- Attending a NHS mental health trust for the treatment of psychosis.
- Has a clinical diagnosis of schizophrenia spectrum psychosis (F20-29) or an affective diagnosis with psychotic symptoms (F31.2, 31.5, 32.3, 33.3) (ICD-10, WHO, 2010).
- Having self-reported difficulties going outside their home primarily due to anxiety (and hence would score on the primary outcome) that they would like treated.

2.2.2 Exclusion Criteria

The participant may not enter the trial if ANY of the following apply:

- Unable to attempt an Oxford – Behavioural Task (O-BAT) at baseline (e.g. due to being unpermitted to leave a psychiatric ward).
- Photosensitive epilepsy.
- Significant visual, auditory, or balance impairment.
- Current receipt of another intensive psychological therapy (or about to start it within the 6-week trial therapy window).
- Insufficient comprehension of English.
- In forensic settings or Psychiatric Intensive Care Unit (PICU).
- Organic syndrome.
- Primary diagnosis of alcohol or substance disorder or personality disorder.
- Significant learning disability.
- Current active suicidal plans.
- A participant may also not enter the trial if there is another factor, which, in the judgement of the investigator, would preclude the participant from providing informed consent or from safely engaging with the trial procedures. Recruitment will be suspended for people who have any of the conditions that would make them high or moderate risk (clinically vulnerable) for a severe course of COVID-19 (https://www.nhs.uk/conditions/coronavirus-covid-19/people-at-higher-risk/whos-at-higher-risk-from-coronavirus/). However, people who are at moderate or high risk for a severe course of COVID-19 will be able to join the trial if they have received the COVID-19 vaccine (subject to medical advice). Reason for exclusion will be recorded in line with CONSORT 2010 Statement (Schulz et al, 2010).

2.3 Sample size

Approximately 432 participants will be recruited into this trial, with 216 in each arm. This sample size takes into consideration a maximum attrition rate of 20%, and provides 90% power to detect a difference of around 8 (standard deviation =23) in O-BAT score, from randomisation to 6 weeks (i.e. standardised effect size of 0.35) at 5% level of significance (2-sided).

2.4 Randomisation and blinding in the analysis stage

Participants will be randomised once they have completed the baseline assessment. Participants will be allocated to one of the trial arms using a 1:1 allocation ratio. Randomisation will be carried out by a validated online system, Sortition, designed by the University of Oxford Primary Care Clinical Trials Unit. Randomisation using a permuted blocks algorithm, with randomly varying block size, will be stratified by site (Bristol/Manchester/Newcastle/Nottingham/Oxford) and service type (in-patient/early intervention/community mental health team).

The research assessors will be blinded to group allocation, but the patients and staff member present will not be (they cannot be blinded to what psychological intervention is delivered or received). The staff members setting up and running the VR software will inform patients of the randomisation outcome, to ensure the research assessors remain blinded to group allocation. Precautionary strategies to prevent un-blinding of allocation include the staff member and assessor considering room use and booking arrangements; patients being reminded by the assessor not to talk about their allocation result; and, after the initial assessment, the assessor not looking at the patient's clinical notes. If an allocation is revealed between assessment sessions, this will be logged by the trial coordinator and re-blinding will occur using another assessor. The statisticians will remain blind to allocation.

3 Analysis – General considerations

3.1 Descriptive statistics

Summary descriptions for continuous measurements will be means and standard deviations. Medians and interquartile ranges will be also presented if more appropriate, along with minimum and maximum values. Counts and percentages will be presented for categorical variables, including counts of missing data. Summary statistics will be provided by randomised group and overall.

3.2 Baseline characteristics of participants

Baseline characteristics of the patients (demographics and baseline of the primary and all secondary outcome variables where available) will be reported by randomised group as well as the overall. There will be no tests of statistical significance nor confidence intervals for differences between randomised groups on any baseline variables.

Patient flow from screening through randomisation, follow up and analysis will be presented in a CONSORT flow chart (Appendix II).

TABLE 1: BASELINE CHARACTERISTICS OF PARTICIPANTS BY RANDOMISED GROUP

	**VR THERAPY + TAU N=**	**TAU ONLY N=**	**OVERALL N=**
**Age (years)** *[AGE]*	Mean (SD)<br>Median (IQR)<br>(Range)	Mean (SD)<br>Median (IQR)<br>(Range)	Mean (SD)<br>Median (IQR)<br>(Range)
**Age at first contact with mental health services (years)** *[AGEFR]*	Mean (SD)<br>Median (IQR)<br>(Range)	Mean (SD)<br>Median (IQR)<br>(Range)	Mean (SD)<br>Median (IQR)<br>(Range)
**Sex** *[SEX]*<br>Female<br>Male<br>Other<br>Prefer not to disclose	n (%)	n (%)	n (%)
**Current Marital Status** *[MARITAL]*<br>Single<br>Married/civil partnership<br>Cohabiting<br>Separated<br>Divorced<br>Widowed	n (%)	n (%)	n (%)
**Ethnic group** *[ETHNIC]*<br>White<br>Black British<br>Black African	n (%)	n (%)	n (%)

Black Caribbean<br>Indian<br>Black Other<br>Chinese<br>Pakistani<br>Other<br>Prefer not to disclose			
**Currently taking any medication** *[MEDYN_E2_C2]*<br>Yes<br>No	n (%)	n (%)	n (%)
**Type of medications in use** *[ANTIPSYYN, ANRIDEPYN, ANXIOYN, MOODSYN, HYPNOYN, STIMYN & PRNMEDYN]*	n (%)	n (%)	n (%)
**Site**[1] *[SITEID_E2_C2]*<br>Bristol<br>Manchester<br>Newcastle<br>Nottingham<br>Oxford	n (%)	n (%)	n (%)
**Service type**[1] *[CURSRV_E1_C1]*<br>Community MH team<br>Early intervention (EIP)<br>In-patient	n (%)	n (%)	n (%)
**Mental health diagnosis** *[FCODE_E1_C1]*	n (%)	n (%)	n (%)
**Employment** *[CSRI3_E2_C2]*<br>Employed full-time (paid)<br>Employed part-time (paid)<br>Employed part-time (voluntary)<br>Unemployed (on benefits)<br>Unemployed (not on benefits)<br>Student or in training full-time<br>Student or in training part-time<br>Self-employed<br>Home-maker<br>Retired<br>Other	n (%)	n (%)	n (%)
**Usual/Normal living arrangement** *[CSRI1_E2_C2]*<br>Living alone (+/- children)<br>Living with husband/wife<br>Living with partner<br>Living with parents<br>Living with other relatives<br>Living with others (e.g. friends)	n (%)	n (%)	n (%)
**PRIMARY OUTCOMES AT BASELINE:**			

**O-AS Avoidance & Distress scores**	Mean (SD)<br>Median (IQR)<br>(Range)	Mean (SD)<br>Median (IQR)<br>(Range)	Mean (SD)<br>Median (IQR)<br>(Range)
**SECONDARY OUTCOMES AT BASELINE:**<br>**Summaries of total scores for: Actigraphy, time-budget, AMI-A, O-BAT, R-GPTS, PWQ, PHQ-9,C-SSRS, EQ5D VAS, EQ5D Index Value, ReQol & QPR,**	Mean (SD)<br>Median (IQR)<br>(Range)	Mean (SD)<br>Median (IQR)<br>(Range)	Mean (SD)<br>Median (IQR)<br>(Range)

[1] Stratification factors

3.3 Definition of population for analysis

After randomisation, all participants for whom data are available will be analysed according to their allocated intervention group, irrespective of what intervention they actually receive.

3.4 Pooling of investigational sites

Participants will be recruited from multiple sites and the randomisation stratified on region (Bristol/Manchester/Newcastle/Nottingham/Oxford). Region will be included as a fixed effect in the analysis.

3.5 Data Monitoring Committee And Interim Analyses

A Data Monitoring and Ethics Committee (DMEC) has been formed to monitor the safety and progress of the trial. The DMEC is comprised of independent experts in areas relevant to the trial. The DMEC will meet at least twice a year and will be governed by a charter that adheres to the MRC DMEC guidance. An independent expert will chair the DMEC and will oversee the data and management of Serious Adverse Events.

4 Derivation of variables

4.1 Primary outcome - Oxford Agoraphobic Avoidance Scale (O-AS)

The O-AS is a questionnaire that comprises eight real-world tasks. For each task the person is asked to decide between 'Yes, I could do this now' or 'No, I'd get too anxious' and also to rate the anxiety they would feel doing each task. Each O-AS item is rated on two separate response scales: Avoidance (0-8) and Distress (0-80). The two outcomes for the O-AS are derived as follows:

Avoidance:

Avoidance of any of the eight items is indicative of clinically elevated levels of agoraphobic avoidance. The sum of the responses for the eight questions results in a continuous outcome ranged 0-8, where 0 = all eight responses are 'Yes I can do this now' (indicating no avoidance). The eight responses contributing to the Avoidance outcome are recorded in the following variables:

- *SROBAT7* Stand outside your home on your own for 5mins
- *SROBAT9* Walk down a quiet street on your own
- *SROBAT10* Walk down a busy street with someone you know
- *SROBAT21* Travel on your own on the bus for several stops
- *SROBAT26* Sit in the waiting room of your GP/health centre on your own for 5mins
- *SROBAT31* Purchase an item in a local shop, from a shop assistant
- *SROBAT33* Go to a shopping centre on your own for 15mins
- *SROBAT38* Sit in a café on your own for 10mins

These will be summed to get an overall avoidance score which can be interpreted as follows:
0 = Average avoidance
1= Moderate avoidance
3 = High avoidance
6 = Severe avoidance

Distress:

The eight responses making up the overall Distress score are recorded in the following variables:

- *SROSCL7* Stand outside your home on your own for 5mins
- *SROSCL9* Walk down a quiet street on your own
- *SROSCL10* Walk down a busy street with someone you know
- *SROSCL21* Travel on your own on the bus for several stops
- *SROSCL26* Sit in the waiting room of your GP/health centre on your own for 5mins
- *SROSCL31* Purchase an item in a local shop, from a shop assistant
- *SROSCL33* Go to a shopping centre on your own for 15mins
- *SROSCL38* Sit in a café on your own for 10mins

These will be summed to get an overall distress score which can be interpreted as follows:
≤23 = Average distress

24-46 = Moderate distress
46-66 = High distress
66+ = Severe distress

4.2 Secondary outcomes

ACTIVITY LEVELS

4.2.1 Actigraphy

Actigraphy measures the number of steps taken using activity watches over a 7-day period at each of the three time points. An average number of steps per day will be calculated using the number of steps taken over the 7-day period. There will be a minimum requirement of 3 days' worth of step data to calculate the average. Participants who wear the activity watch for less than 3 days will be considered missing data. Variables to be used are *D1DAT- D7DAT* for the dates and *D1STEPS- D7STEPS* for the step counts

4.2.2 Time Budget

The Time Budget tool is an interviewer rated measure of activity specifically designed for patients with psychosis. It measures activity over a 7-day period using four time points across the day and is completed retrospectively. At each time point, the activity is rated from 0 (low) to 4 (high) according to how complex the activity is and the effort required over and above doing nothing. The total score is a sum of the individual scores over all days and times within each day. It ranges from 0-112.

PSYCHIATRIC SYMPTOMS

4.2.3 Agoraphobia Mobility Inventory-Avoidance [AMI-A]

This is an avoidance measurement tool, where each of the 29 items is scored from 1-5, or N/A if not in the geographical zone or not relevant (i.e. this indicated that the question was asked). For the purposes of analysis, N/As will be treated as missing data. Half-points are allowed. A mean score will be calculated for each participant **only** for the items they responded to (i.e. exclude any items marked as N/A). The outcome will only be missing if all items are missing or N/A.

4.2.4 Oxford Behavioural Avoidance Task [O-BAT]

1. The maximum number of steps (from 1 – 5) at each time point will be determined based on the number of steps completed at that time point *[OBAT5YN-OBAT1YN]*. These will be recoded so that Yes=0 and No=1, implying that higher scores indicate higher avoidance.
2. The level of distress felt when doing the task is scored on a scale from zero to 10 *[BATSCL5 - BATSCL1],* where higher scores indicate greater distress. The scale increases in increments of 0.5 and will be treated as a continuous measure. For the analysis, it will be necessary to

compare the mean distress score of the steps completed at both baseline AND the same steps at follow-up e.g., if a participant reached step 3 at baseline and step 4 at follow-up, the mean distress score of steps 1 – 3 will be compared.

4.2.5 Revised-Green et al Paranoid Thoughts Scale [R-GPTS]

Overall paranoia will be assessed by the Revised GPTS (Green et al., 2008; Freeman et al., 2019) which measures two dimensions of paranoid thinking: ideas about social reference and ideas about social persecution. The social reference subscale (Part A) consists of 8 statements and the persecution subscale (Part B) consists of 10 statements. These statements are rated according to how true the subject believes the statement to be on a Likert scale from 0 (don't believe at all) to 4 (totally believe). The total score for each dimension is obtained by summing all responses, ranging from 0-32 for the social reference subscale and 0-40 for the persecution subscale, with higher scores reflecting higher levels of paranoia. There is also a total score which ranges from 0-72 (Freeman et al., 2019). We will test the scores for Part A and Part B and also the total score.

4.2.6 Paranoia Worries Questionnaire [PWQ]

This is a 5-item scale with a total score of 20. Each item *[PWQ1- PWQ5]* is scored from 0-4 such that higher scores indicate higher levels of paranoia worries

The baseline variables are *PWQ1 - PWQ5* while the variables for both follow-up time point are *WQ1 - WQ5*.

4.2.7 Patient Health Questionnaire [PHQ-9]

The PHQ-9 is a 9-item depression scale where each item is scored from 0-3. The total score is the sum of the individual item scores and can thus be a maximum of 27. Higher scores here indicate higher levels of depression. The continuous total score will be used for analysis while the following categories of depression, derived from the total score, will be summarised descriptively:

- 0-4 = Minimal depression
- 5-9 = Mild depression
- 10-14 = Moderate depression
- 15-19 = Moderately severe depression
- 20-27 = Severe depression

4.2.8 Columbia Suicide Severity Rating Scale [C-SSRS]

The C-SSRS is a semi-structured interview tool that measures suicide ideation and behaviour. It consists of four categories of questions (Suicide ideation, Intensity of ideation, Suicidal behaviour & Preparatory acts). The Suicidal Ideation scale is a 6-point ordinal scale, with scores for those with

ideation ranging from 1 (wish to be dead) to 5 (suicidal intent with plan). Those who denied ideation receive a score of zero. The question number relates to the score, with the most severe ideation coming from the highest numbered item where the response was 'Yes'. This highest score (range 0-5) will be used in the analysis and the data are found in the variables *CSSRS1- CSSRS5*.

The Intensity of Ideation subscale is comprised of five items (i.e., Frequency *[CSSRS5_FRQ]*, Duration *[CSSRS5_ DUR]*, Controllability *[CSSRS5_ CON]*, deterrents *[CSSRS5_ DET]* & Reason for ideation *[CSSRS5_ REAS]*) each rated on a scale from 1 to 5 (total scores ranging from 1 to 25) and relates to the response in the Suicidal Ideation section. These data will be summarised by treatment group, together with the Suicidal Behaviour *[CSSRS6- CSSRS8]* and Preparatory acts or behaviour *[CSSRS9-CSSRS10]* scales. Where appropriate, the data will be graphically presented using stacked bar graphs.

The above analyses will include self-reported responses for the C-SSRS questionnaire.

QUALITY OF LIFE

4.2.9 EQ-5D-5L - index and the VAS

The EQ-5D-5L index will be calculated using the cross walk method. This method uses country-specific weighting of each item for the United Kingdom (see https://www.unmc.edu/centric/_documents/EQ-5D-5L.pdf).

The VAS scores are continuous measures on a scale of 0 to 100.

4.2.10 Recovering quality of life [ReQol-20]

ReQoL is a patient-reported questionnaire that has been developed to assess quality of life for people with different mental and physical health conditions. It consists of 20 mental health questions and one physical health question. Although physical health is important to the quality of life of mental health service users, it is not included in the total because it is distinct from mental health.

For each mental health item, the participants indicate how often they agree with the statement at the time of completing it. The options are scaled from 0-4 ('None of the time' to 'Most of the time'). Variables for the scale are *REQOL1- REQOL20*.

Note: There are 9 positively and 11 negatively framed questions. The following items represent positively worded questions: Q2, Q4, Q5, Q7, Q8, Q10, Q11, Q15, Q19 and are scaled from 0 ('None of the time') to 4 ('Most of the time'). The remaining items (Q1, Q3, Q6, Q9, Q12, Q13, Q14, Q16, Q17, Q18, Q20) represent negatively worded questions and are reverse scored, i.e. 0 = 4, 1 = 3, 2 = 2, 3 = 1 and 4 = 0. The total score is the sum of the scores for the 20 questions and is out of 80, where 0 indicates the poorest quality of life and 80 the highest quality of life.

Missing responses: If a maximum of two questions are unanswered in the whole measure, an overall ReQoL-20 score can still be calculated. In such a case, the mean value of the other responses can be used to fill the gap to calculate the overall index. If three or more questions are unanswered, then the overall index score cannot be calculated.

Question 21 *[REQOL21]* refers to physical health only and will be analysed descriptively.

4.2.11 Questionnaire on progress of recovery [QPR]

This questionnaire aims to shed more light on the process of recovery. Each item is scored from 0-4 and the QPR outcome will be calculated as the sum of all responses from the 15 items within the questionnaire (range 0 to 60).

FOR THE MEDIATION ANALYSIS:

4.2.12 Oxford Cognitions and defences Questionnaire [O-CDBQ]

(Refer to the document 'CDBQ_Final Scale_for CTU.docx' for the items and scoring as these have been updated since the CRF was developed.)

The three parts of the CDBQ will be assessed separately:

1. Part 1 (Fearful thoughts about being outside (worry)). Sum all 14 items *[CDBQ_A1 - CDBQ_A14]* to create a total "threat beliefs" score out of 42. (Higher score indicates higher worry).
2. Part 2 (Keeping away from outside situations (avoidance)). Sum all 11 items to create a total "avoidance" score out of 33 - **please note** the corresponding item numbers as these match the CRF. The ones to include in the calculation are: *CDBQ_B1, CDBQ_ B2, CDBQ_ B3, CDBQ_ B4, CDBQ_ B7, CDBQ_B9, CDBQ_B10, CDBQ_ B12, CDBQ_ B13, CDBQ_ B14, CDBQ_ B15*
3. Part 3 (Dealing with risks when outside). Sum all 8 items to create a total "within situation defences" score out of 24 - **please note** the corresponding item numbers as these match the CRF. Items to include are *CDBQ_ C1, CDBQ_C3, CDBQ_C4, CDBQ_C5, CDBQ_C6, CDBQ_C7, CDBQ_C9, CDBQ_C10*

4.2.13 Visual Analog Scales of safety

Safety behaviours (strength of safety beliefs, vulnerability belief, and threat anticipation) will be assessed separately using three visual analogue scales, each with a score ranged 0-100:

1. Strength of safety beliefs *[VAS1 - "I generally feel safe around other people."]*
2. Vulnerability belief *[VAS2 - "I feel vulnerable."]*
3. Threat anticipation *[VAS3 -"When I go out, something bad will happen."]*

FOR THE MODERATION ANALYSIS:

4.2.14 Hallucinations scale

(Voices Questionnaire) This is a 5-item questionnaire [*VS1 – VS5*] where participants indicate if they have had the experiences on a scale of 0-4 which indicates how often they occurred over the past

three months. The total score is the sum of the individual item responses and has a maximum of 20. Higher scores indicate more experiences of voices.

4.2.15 Beck Hopelessness Scale (Beck, 1988) [BHS]

This questionnaire consists of 20 statements. If the statement describes the participant's attitude for the past week including today, they circle the "T" indicating TRUE. If the statement does not describe their attitude, they circle the "F" indicating FALSE in the column next to this statement.

Note: Optimistic responses are scored as 0 and pessimistic responses are scored as 1. The total is out of 20 where higher scores reflect more hopelessness. The following items represent optimistic responses: 1, 3, 5, 6, 8, 10, 13, 15 and 19. The remaining items represent pessimistic responses. Variables for the scale are *BHS1-BHS20*.

4.2.16 Body-esteem Scale for Adolescents and Adults (Mendelson et al, 2001)

This questionnaire consists of 23 items (however only 21 were asked), where for each item the participants indicate how often they agree with the statement at the time of completing it. The options are scaled from 1-5 (Never - Always). As the original questionnaire is scaled from 0-4, the observed data will need to be changed to this scale first to match the original. Variables for the scale are *BESAA1-BESAA21*.

Note: There are positively and negatively framed questions (1, 2, 3, 4*, 5, 6, 7*, 8, 9*, 10*, 11, 12*, 13, 14, 15, 16*, 17*, 18, 19*, 20, 21). Pessimistic items are indicated by an asterisk and must be recoded for scoring by reversing the scale (i.e., 0 = 4, 1 = 3, 2 = 2, 3 = 1 and 4 = 0). The total score is out of 84 where higher scores reflect more positive body esteem.

4.2.17 Oxford Cognitions and defences questionnaire [O-CDBQ]

Only Part 1 will be used for the moderation analysis and is derived as in section 4.2.12.

4.3 Sensitivity analysis

4.3.1 CACE analysis

The number of VR therapy sessions attended will be calculated from the Therapy log. A session is considered as completed if the response to the question "Participant attended?" is 'Yes' [PATTEN =1]]. A sufficient level of adherence will be defined as attendance of at least 3 VR therapy sessions.

5 PRIMARY ANALYSIS

5.1 Primary outcome

The primary hypothesis is that compared to treatment as usual, VR cognitive therapy coupled with treatment as usual will reduce distress and avoidance of real world situations. The primary hypothesis will be tested for between-group difference in:

1. The O-AS Avoidance outcome at 6 weeks , and
2. The O-AS distress score at 6 weeks

Both outcomes will be analysed using linear mixed-effects regression, modelling the response at 6 weeks and 26 weeks. The baseline outcome measure, stratification variables, and treatment assignment will be fitted as fixed effects with a patient-specific random intercept. An interaction between time and randomised group will also be fitted as a fixed effect to allow estimation of treatment effect at all the time points. The linear mixed effects model will account for missing data assuming data are missing-at-random (MAR). Standard residual diagnostics will be assessed for the appropriateness of the model.

P<0.05 will be used as the level of statistical significance for all tests carried out. Results will be reported as mean differences between treatment groups together with 95% confidence intervals. If either the Avoidance or Distress outcomes are not normally distributed, appropriate transformations or non-parametric statistical methods will be applied.

Treatment differences estimated from linear mixed effects models will additionally be reported as standardised mean differences (mean group difference divided by whole group SD at baseline).

5.2 Handling missing data

Missing data on individual measures will be pro-rated if more than 75% of the items are completed; otherwise, the measure will be considered as missing.

Mixed-effects models implicitly account for data missing at random, however the data missingness mechanism will be explored. Logistic regression models will be used to explore any association between baseline characteristics and availability of the primary outcomes (1=response; 0=non-response). Covariates found to be predictive of missingness will be included in the primary analysis as a sensitivity test.

5.3 Handling outliers

Any outliers will be checked and verified to ensure that they are true values. Outliers will be identified as those observations more than three standard deviations from the mean. Once they have been confirmed, a sensitivity analysis will be carried out to assess the impact of these values on the results by excluding these participants from the primary outcome analysis.

5.4 Handling multi-centre/clustered data

As randomisation is stratified by recruitment site, this will be adjusted for in the analysis by including it as a fixed effect in the statistical models.

5.5 Multiple comparisons and multiplicity

The primary outcome is clearly stated in the protocol and no adjustments for multiple comparisons will be made.

5.6 Model Assumptions

Assumptions of normality and constant variance for linear mixed-effects models will be assessed using residual and other diagnostic plots. Where the assumptions do not appear to be satisfied, a suitable transformation will be applied to the data or a suitable non-parametric method will be used.

6 SECONDARY ANALYSIS

6.1 Secondary outcomes

6.1.1 Continuous secondary outcomes

Unless otherwise stated, all continuous secondary outcomes will be analysed individually in the same way as the primary outcomes. The proposed analysis assumes that these secondary outcomes satisfy the assumptions of the linear mixed effect model. Where these assumptions are not satisfied, the data will be transformed or if a transformation is not possible, a non-parametric approach to analysing the data will be adopted.

For the C-SSRS, the Suicidal Ideation scale outcomes will be tested. For the other scale items, the scoring guideline states that no formal statistical hypothesis testing is recommended for individual studies as only few events are typically observed and so descriptive analyses will then suffice. In the instance that there are too few events for analysis, the Suicide Ideation scale will also be summarised descriptively along with the others.

In addition, the O-BAT will be summarised as follows:

SAMPLE TABLE FOR REAL WORLD DISTRESS OUTCOME – STEPS COMPLETED

	Intervention			Control			Overall		
	Baseline	6 weeks	26 weeks	Baseline	6 weeks	26 weeks	Baseline	6 weeks	26 weeks
**Completed step 1, n (%)**<br>**Distress level**<br>Mean (SD)<br>Median (IQR)<br>Range									

**Completed step 2, n (%)**<br>**Distress level**<br>Mean (SD)<br>Median (IQR)<br>Range									
**Completed step 3, n (%)**<br>**Distress level**<br>Mean (SD)<br>Median (IQR)<br>Range									
**Completed step 4, n (%)**<br>**Distress level**<br>Mean (SD)<br>Median (IQR)<br>Range									
**Completed step 5, n (%)**<br>**Distress level**<br>Mean (SD)<br>Median (IQR)<br>Range									
**Total steps completed, n (%)**<br>**Distress level**<br>Mean (SD)<br>Median (IQR)<br>Range									

SAMPLE TABLE FOR REAL WORLD DISTRESS OUTCOME – HIGHEST STEP COMPLETED

Highest step completed	Intervention			Control			Overall		
	Baseline	6 weeks	26 weeks	Baseline	6 weeks	26 weeks	Baseline	6 weeks	26 weeks
**1, n (%)**									
**Distress level**									
Mean (SD)									
Median (IQR)									
Range									
**2, n (%)**									
**Distress level**									
Mean (SD)									
Median (IQR)									
Range									
**3, n (%)**									
**Distress level**									
Mean (SD)									
Median (IQR)									
Range									
**4, n (%)**									
**Distress level**									
Mean (SD)									
Median (IQR)									
Range									
**5, n (%)**									
**Distress level**									
Mean (SD)									
Median (IQR)									
Range									
**Overall distress, n (%)**									
**Distress level**									
Mean (SD)									
Median (IQR)									
Range									

6.1.2 Binary secondary outcomes

There are no binary secondary outcomes.

6.1.3 Mediation Analysis

The mediation analysis of the primary outcomes will investigate the following putative mediational factors using modern causal inference methods:

(i) O-CDBQ Part1
(ii) O-CDBQ Part3
(iii) Strength of safety beliefs *[VAS1]*
(iv) Vulnerability belief *[VAS2]*
(v) Threat anticipation *[VAS3]*

This involves using parametric regression models to test for mediation of VR therapy on the outcome through the putative mediators. Analyses will adjust for baseline measures of the mediator, outcomes, and possible measured confounders. We will include repeated measurement of mediators and outcomes to account for classical measurement error and baseline confounding. The analysis will be carried out separately on both the Avoidance and Distress scales of the primary outcome.

Measurement error models, as described by Dunn et al, 2015, involve estimating a latent variable for the mediator at each time point representing the "true" value of the mediator. The model is identified by assuming the variance of the measurement error remains constant over time. Figure 1 shows an example path diagram for the measurement error mediation model. The correlation between baseline mediator and treatment allocation is constrained to be zero, under the assumption that there will be no baseline imbalance under randomisation.

Mediation analysis will be carried out on each of the primary outcomes at two time points simultaneously (i) change in O-AS score from baseline to 6 weeks and (ii) change in O-AS from baseline to 26 weeks. The model will be adjusted for baseline values of the mediator and outcome. This model will be estimated using full information maximum likelihood, a method that produces estimates that are valid under the missing at random assumption. As temporal precedence cannot be easily established when mediator and outcome are measured at the same time, this model will also be investigated for possible reverse causality.

The mediation models assume no unobserved confounding of the mediator-outcome relationship and by adjusting for baseline values of the mediator and outcome, a major potential source of confounding has been taken into account. The full information maximum likelihood method assumes multivariate normality. Confidence intervals for the indirect effects will be estimated using bootstrapping. Model fit will be assessed using the root means squared error of approximation (RMSEA) and a likelihood ratio test comparing against a null model. Models will be fitted using the structural equation model builder in STATA.

In the case that the model cannot be identified, it will be conducted using the approach of Baron and Kenny (1986), but will follow the adaptation in Freeman et al. (2017) which makes use of linear mixed effects models. This is valid under a MAR assumption but does not account for measurement error in the mediator.

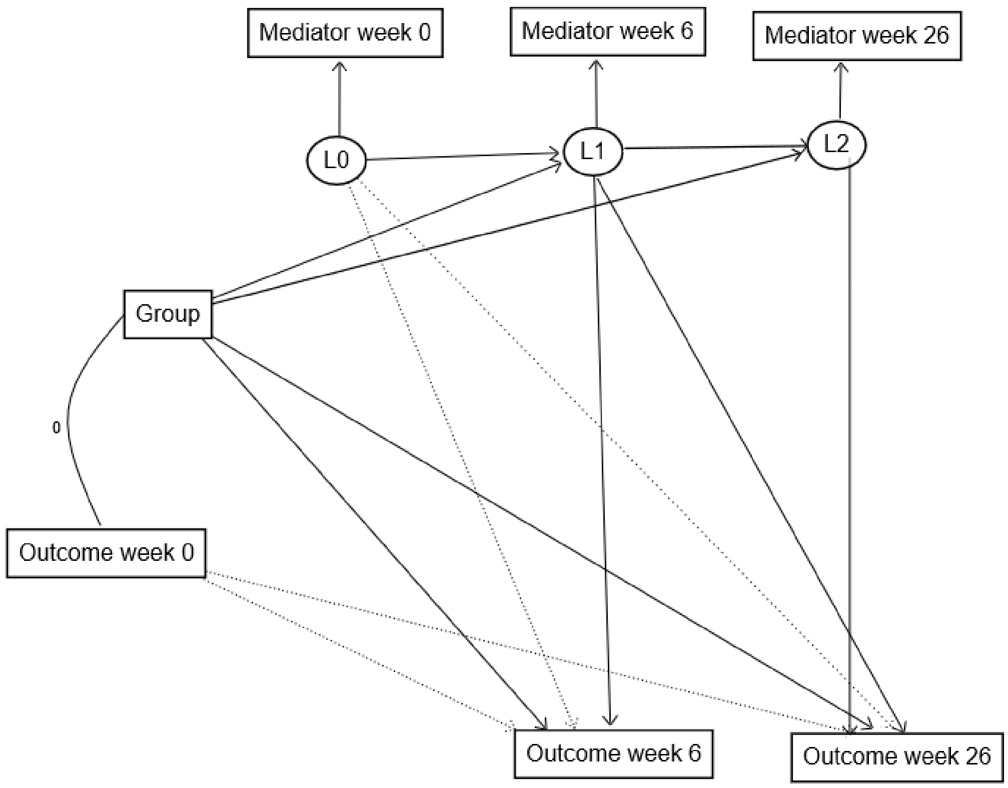

FIGURE 1 EXAMPLE PATH DIAGRAM FOR THE MEASUREMENT ERROR BASED MEDIATION MODEL

6.1.4 Moderation/Subgroup Analysis

For each of the 4 moderator variables, the analysis will be carried out in a similar way to the primary outcome analysis, but modelling the response at the 6 week time point only (thus excluding treatment-by-time interaction). To assess how the relationship between treatment group and the outcome changes as the moderator increases, an interaction between randomised group and the moderator will be fitted and the p-value for the interaction will be reported.

An additional subgroup analysis will be carried out on the type of treatment deliverer (clinical psychologist, assistant psychologist, peer support worker). This data will be made available on a separate spreadsheet and merged with the analysis dataset. The distribution of treatment deliverer across sites will be also be summarised.

7 ADDITIONAL EXPLORATORY ANALYSIS NOT SPECIFIED IN THE PROTOCOL

7.1 Medications

All psychotropic medication prescribed at each time point (Baseline, 6 weeks, 26 weeks) for each participant will be reported. Data will be collected from medical records. The WHO Defined Daily Dose (DDD) (https://www.whocc.no) will be used to calculate medication data. This is the gold standard for international drug utilisation methodology. It provides data for the main categories of psychotropic medication, allowing assessment of concomitant (or alternative) medication in addition to antipsychotics. The DDD is defined as, "the assumed average maintenance dose per day for a drug used for its main indication in adults."

For antipsychotics both the DDD and chlorpromazine equivalent (CPZequiv) can be identified. CPZequiv is calculatd using the calculation table produced by Woods et al (2003) https://pubmed.ncbi.nlm.nih.gov/12823080/.

Medication data analysis

Descriptive analysis will be carried out on the following:

1. Dichotomous variable for psychotropic medication e.g. antipsychotic, antidepressant, anxiolytic, mood stabiliser, hypnotic, stimulant etc.
2. If Y to above, medication dose will need to be converted to the defined daily dose (DDD) in order to compare across different types of medication.
3. There is also another conversion for anti-psychotic medication: chlorpromazine equivalent.
4. Summarise whether participant was prescribed any PRN medication ('pro re nata' means that the administration of medication is not scheduled).
5. Total number of psychotropic medications prescribed.

**Variable description [*Variable Name]***	**Variable Type**
Antipsychotic prescribed *[ANTIPSYYN]*	Dichotomous Yes/No
Antipsychotic DDD *[DDD1]*	Numerical
Antipsychotic CPZequiv *[CPZ1]*	Numerical
Antidepressant prescribed *[ANRIDEPYN]*	Dichotomous Yes/No
Antidepressant DDD *[DDD2]*	Numerical
Anxiolytic prescribed *[ANXIOYN]*	Dichotomous Yes/No
Anxiolytic DDD *[DDD3]*	Numerical
Mood stabiliser prescribed *[MOODSYN]*	Dichotomous Yes/No
Mood stabiliser DDD *[DDD4]*	Numerical
Hypnotic prescribed *[HYPNOYN]*	Dichotomous Yes/No
Hypnotic DDD *[DDD5]*	Numerical
Stimulant prescribed *[STIMYN]*	Dichotomous Yes/No
Stimulant DDD *[DDD6]*	Numerical
PRN medication prescribed *[PRNMEDYN]*	Dichotomous Yes/No
Total number psychotropics prescribed *[PSYCHOTNUM]*	Numerical

7.2 Service use

Service use will be summarised by treatment allocation. Psychiatric admissions data can be obtained from the Medical Record Data Screen.

Variables to be tabulated:

- Psychiatric admission (number of nights) (Medical Record Data Screen Q1 [**WARD_DUR]**)
- Physical health admission (number of nights) (*CSRI original interview version* Q15 [**HOSP_DUR]**; *self-report version* Part 4 Q1 [**HOSP_DUR_PH]**)
- A&E attendance (*CSRI original interview version* Q16:Q1 [**CSRI16_1]**; *self-report version* Part 5 Q1:1 [**CSRI16_1_AS]**)
- Meetings with psychiatrist (*CSRI original interview version* Q16:Q2 [**CSRI16_2]**, Q16:Q3 [**CSRI16_3]**; *self-report version* **[CSRI16_2_AS]**)
- Meetings with care coordinator (CPN or social worker) (*CSRI original interview version* Q16:Q10 [**CSRI16_10]**, Q16:Q13 Q10 [**CSRI16_13]**, Q16:Q17 [**CSRI16_16]**; *self-report version* Part 5 Q1.8 [**CSRI16_10_AS]**, Part 5 Q1.9 [**CSRI16_13_AS],** Part 5 Q1.13 **[CSRI_OTHDET_AS])**
- Meetings with counsellor or therapist (CSRI Q16:Q11 original interview version **[CSRI16_11]**; self-report version Part 5 Q1:10 [**CSRI16_11_AS]**)
- Visits to day hospital/ day-care centre (*CSRI original interview version* Q16:Q5 [**CSRI16_5]**; Q16:Q18 [**CSRI16_17]**; *self-report version* Part 5 Q1.11 [**CSRI16_5_AS]**)
- GP meetings (*CSRI original interview version* Q16:Q6 [**CSRI16_6]**, Q16:Q7 [**CSRI16_7]; *self-report version*** Part 5 Q1.3 **[CSRI16_7_AS],** Part 5 Q1.5 **[CSRI16_6_AS]**)

	**VR THERAPY + TAU**		**TAU only**	
	n (%)	Mean (SD)	n(%)	Mean (SD)
**6 months before the trial**				
Psychiatric inpatient admission: number of nights in hospital (total across all admissions)	(n= admissions)		(n= admissions)	
Physical health inpatient admission: number of nights in hospital (total across all admissions)	(n= admissions)		(n= admissions)	
Attendance at Accident and Emergency				
Meetings with psychiatrist				
Meetings with care coordinator (CPN or social worker)				
Meetings with counsellor or therapist				
Visits to day-care centre / day hospital				
GP meetings				
**During trial participation: Baseline to 6 weeks (post-treatment) assessment**				

Psychiatric inpatient admission: number of nights in hospital (total across all admissions)<br><br>Physical health inpatient admission: number of nights in hospital (total across all admissions)<br><br>Attendance at Accident and Emergency<br><br>Meetings with psychiatrist<br><br>Meetings with care coordinator (CPN or social worker)<br><br>Meetings with counsellor or therapist<br><br>Visits to day-care centre / day hospital<br><br>GP meetings	(n= admissions)<br><br>(n= admissions)	(n= admissions)<br><br>(n= admissions)
**During trial participation: 6 weeks to 26 weeks (follow-up) assessment**		
Psychiatric inpatient admission: number of nights in hospital (total across all admissions)<br><br>Physical health inpatient admission: number of nights in hospital (total across all admissions)<br><br>Attendance at Accident and Emergency<br><br>Meetings with psychiatrist<br><br>Meetings with care coordinator (CPN or social worker)<br><br>Meetings with counsellor or therapist<br><br>Visits to day-care centre / day hospital<br><br>GP meetings	(n= admissions)<br><br>(n= admissions)	(n= admissions)<br><br>(n= admissions)

Standard care (i.e. psychological therapy) by allocation – data obtained from the Medical Records Data Screen [**THRPY_TYPE]**)

	VR THERAPY + TAU events (people)	TAU events (people)	Overall events (people)
Any other therapy – 6 weeks			
CBT**			
CBT for psychosis			
1:1 psychology on ward			
Ward group			
Any other therapy – 26 weeks			

** Examples included. Final categories to be grouped by trial team due to free text box

8 SENSITIVITY ANALYSIS

This will be carried out on both the Avoidance and Distress outcomes.

8.1 Outliers and missingness assumptions

1. If outliers are identified, a sensitivity analysis excluding these outliers will be carried out to determine the impact of these observations on the treatment effect of both the primary outcomes as per the primary outcome analysis specified in section 5.1.
2. As a sensitivity analysis of the primary outcomes, baseline covariates found to be predictive of missingness will be included as main effects in the linear mixed-effects models outlined in section 5.1.
3. A sensitivity analysis will also be carried out for the primary outcomes using methods which do not assume a MAR mechanism, such as pattern-mixture models, to assess the robustness of this assumption. If different results are obtained from the pattern-mixture models compared to the linear mixed-effects models, then it is likely that the MAR assumption is not valid.

8.2 Complier Average Causal Effect (CACE) analysis

As part of the exploration of treatment effects, a CACE analysis of the primary outcomes (O-AS Avoidance and O-AS Distress) at 6 weeks will be carried out to investigate the effect of VR cognitive therapy in patients who sufficiently adhere to the therapy (attend at least 3 VR therapy sessions).

A summary of compliance will be presented for both primary outcomes (Avoidance & Distress) in the control (TAU) group and in the intervention group (VR+TAU).

To obtain an unbiased estimate of the effect of compliance to the intervention on treatment effect, we will estimate the CACE of the mean difference in the primary outcomes between the compliers in the VR+TAU group compared to the would-be compliers in the TAU group (Dunn et al, 2005).

In addition, an instrumental variable approach will be adopted to estimate the CACE estimate of the primary outcomes at 6 weeks (e.g. ivregress in STATA with two stage least squares estimator), adjusting for baseline measures of the outcomes.

Finally, baseline characteristics of the participants included in the CACE analysis will be reported.

8.3 Impact of the COVID-19 pandemic

The UK announced a national lockdown on March 23rd 2020.

March 23rd shall be used as the cut-off date for defining pre and during pandemic periods. Pre-pandemic shall be defined as on or before March 23rd 2020 and during pandemic as after the 23rd March 2020

It is hypothesised that the effect of the COVID-19 pandemic will be detrimental in both arms but that participants in the intervention arm will still have better outcomes. However, this might lead to some treatment effect dilution. Conversely, the intervention might offer some protective effect and lead to a larger treatment effect during the lockdown period. Sensitivity analyses will explore whether there is a difference in the treatment effect at 6 weeks and at 26 weeks when measures are taken after the beginning of lockdown. Two analyses will be conducted on each of the two primary outcomes:

1. This will include all participants who completed their 6-week measures before the beginning of lockdown on the 23rd March 2020 but setting any follow-up measures that were completed after lockdown as missing. This approach will be used to explore whether there is a difference in the treatment effect at the primary endpoint (6 weeks).
2. In order to explore whether there is a difference in the treatment effect at follow-up time-points, a further sensitivity analysis of the primary outcomes will be carried out including only those who completed their 26-week measures before the beginning of lockdown.

Note: Recruitment was suspended for people who have any of the conditions that would make them high or moderate risk (clinically vulnerable) for a severe course of COVID-19. Subsequently individuals at risk who were vaccinated were accepted.

8.4 Shortened follow-up

A sensitivity analysis will be carried out excluding participants randomised from 1 March 2021 as many of these had their 26-week assessments brought forward by up to 6 weeks.

8.5 Medication effects

A sensitivity analysis will explore whether treatment effects might be attributable to prescribing of medication. Prescription of antipsychotic medication will be gathered at baseline, 6 weeks and 26 weeks. This will be converted into a chlorpromazine equivalent score *[CPZ1*]. Similar models to the primary analysis will be fitted to test whether there is a difference in prescribing of medication by treatment arm. (Only if this analysis is statistically significant, a mediation model similar to the one described in section 6.1, will be fitted to test whether any of the treatment effect is mediated by changes in prescribing of medication.)

9 SAFETY ANALYSIS

This is considered to be a low risk trial. Any serious adverse events will be recorded. A Serious adverse event is defined as an event which:

- Results in death or,
- Is a life-threatening illness or injury or,
- Requires [voluntary or involuntary] hospitalisation or prolongation of existing hospitalisation or,
- Results in persistent or significant disability or incapacity or,
- Medical or surgical intervention required to prevent any of the above,
- Leads to foetal distress, foetal death or consists of a congenital anomaly or birth defect or,
- Is otherwise considered medically significant by the investigator.

Summary counts and percentages of the number of adverse and serious adverse events, and the number of people with at least one adverse event will be reported at the end of the trial. Tables will include a summary of the severity and causality for any SAEs. The AE master log will be obtained from the trial team.

10 VALIDATION

The primary analysis and safety data will be validated by a Senior Trial Statistician.

11 CHANGES TO THE PROTOCOL OR PREVIOUS VERSIONS OF SAP

Due to COVID-restrictions, the O-BAT could no longer be used as social distancing guidelines prevented its administration. It was not administered after the 23rd March 2020. The O-AS replaced the O-BAT as the primary outcome measure and a substantial amendment to the protocol was carried out.

Where it is not possible for the questionnaires to be administered face-to-face, they will be administered online, on the phone or via post. Participants are given the option to conduct any of the follow-up assessments by these other methods, following an appropriate risk assessment. For remote assessments, abbreviated or self-report versions of interview measures will be used as necessary e.g. the self-report C-SSRS (Posner et al., 2008).

12 References

Carl, E., Stein, A. T., Levihn-Coon, A., Pogue, J. R., Rothbaum, B., Emmelkamp, P., Asmundson, G. J. G., Carlbring, P., & Powers, M. B. (2018). Virtual reality exposure therapy for anxiety and related disorders: A meta- analysis of randomized controlled trials. Journal of Anxiety Disorders. https://doi.org/10.1002/da.22728

Cella, M., Edwards, C., & Wykes, T. (2016). A question of time: A study of time use in people with schizophrenia. Schizophrenia Research, 176(2–3), 480–484. https://doi.org/10.1016/j.schres.2016.06.033

Lambe, S. et al (2021). The Oxford Agoraphobic Avoidance Scale. Psychological Medicine 1–11. https://doi.org/10.1017/S0033291721002713

Freeman, Dunn et al (2015) Effects of cognitive behaviour therapy for worry on persecutory delusions in patients with psychosis (WIT). Lancet Psychiatry, 2, 305-313.

Freeman, D., Taylor, K., Molodynski, A., & Waite, F. (2019). Treatable clinical intervention targets for patients with schizophrenia. Schizophrenia Research, 211, 44-50.

Hjorthøj, C., Stürup, A. E., McGrath, J. J., & Nordentoft, M. (2017). Years of potential life lost and life expectancy in schizophrenia: a systematic review and meta-analysis. The Lancet Psychiatry, 4(4), 295–301. https://doi.org/10.1016/S2215-0366(17)30078-0

Lindamer, L. A., McKibbin, C., Norman, G. J., Jordan, L., Harrison, K., Abeyesinhe, S., & Patrick, K. (2008). Assessment of physical activity in middle-aged and older adults with schizophrenia. Schizophrenia Research, 104(1–3), 294–301. https://doi.org/10.1016/j.schres.2008.04.040

Morina, N., Ijntema, H., Meyerbröker, K., & Emmelkamp, P. M. G. (2015). Can virtual reality exposure therapy gains be generalized to real-life? A meta-analysis of studies applying behavioral assessments. Behaviour Research and Therapy, 74, 18–24. https://doi.org/10.1016/j.brat.2015.08.010

Mendelson, B. K., White, D. R., & Mendelson, M. J. (1997). Manual for the body-esteem scale for adolescents and adults. (Res. Bull. 16, No. 2). Montreal: Concordia University.

Mendelson, B. K, Mendelson, M. J & White, D. R (2001) Body-Esteem Scale for Adolescents and Adults, Journal of Personality Assessment, 76:1, 90-106, DOI: 10.1207/S15327752JPA7601_6

Nilsson, M.E., Suryawanshi, S., Gassmann-Mayer, C., Dubrava, S., McSorley, P., and Jiang, K. (2013). Columbia–Suicide Severity Rating Scale Scoring and Data Analysis Guide

Rothbaum, B. O., Hodges, L. F., Kooper, R., Opdyke, D., Williford, J. S., & North, M. (1995). Virtual reality graded exposure in the treatment of acrophobia: A case report. Behavior Therapy. Vol 26(3), 26, 547–554. https://doi.org/10.1016/S0005-7894(05)80100-5

Upthegrove, R., Marwaha, S., & Birchwood, M. (2017). Depression and Schizophrenia: Cause, Consequence, or Trans-diagnostic Issue? Schizophrenia Bulletin, 43(2), 240–244. https://doi.org/10.1093/schbul/sbw097

13 Appendices

13.1 Appendix I. Schedule of procedures

Procedures	Screening	Baseline (0 weeks)	VR session 1*	VR session 2*	VR session 3*	VR session 4*	VR session 5*	VR session 6*	Post-therapy (6-16 weeks)	Follow-up (26-36 weeks)
Eligibility assessment	X	X (brief)								
Informed consent		X								
Demographics		X								
Randomisation		X								
O-AS		X							X	X
Activity (actigraphy, time-budget)		X							X	X
R-GPTS		X							X	X
Columbia scale		X							X	X
EQ-5D-5L		X							X	X
ReQol		X							X	X
AMI-A		X							X	X
PHQ-9		X							X	X
Mediators (e.g. safety beliefs)		X							X	X
Moderators (e.g. voices, hopelessness, body-esteem)		X								
Exploratory (coronavirus cognitions)		X							X	X
QPR		X							X	X
Service receipt		X							X	X
VR treatment*			X	X	X	X	X	X		
VR negative effects scale, satisfaction								X		

Adverse event monitoring	X	X	X	X	X	X	X	X	X	X
Treatment as usual	X	X	X	X	X	X	X	X	X	X

In addition to close contact with clinical teams and reporting of adverse events when they are brought to the attention of the team as required, medical notes will be checked at the end of the trial to assess for adverse events and check service receipt.

*Only participants allocated to receive virtual reality therapy will attend VR sessions.

13.2 Appendix II. Flow diagram of trial participants

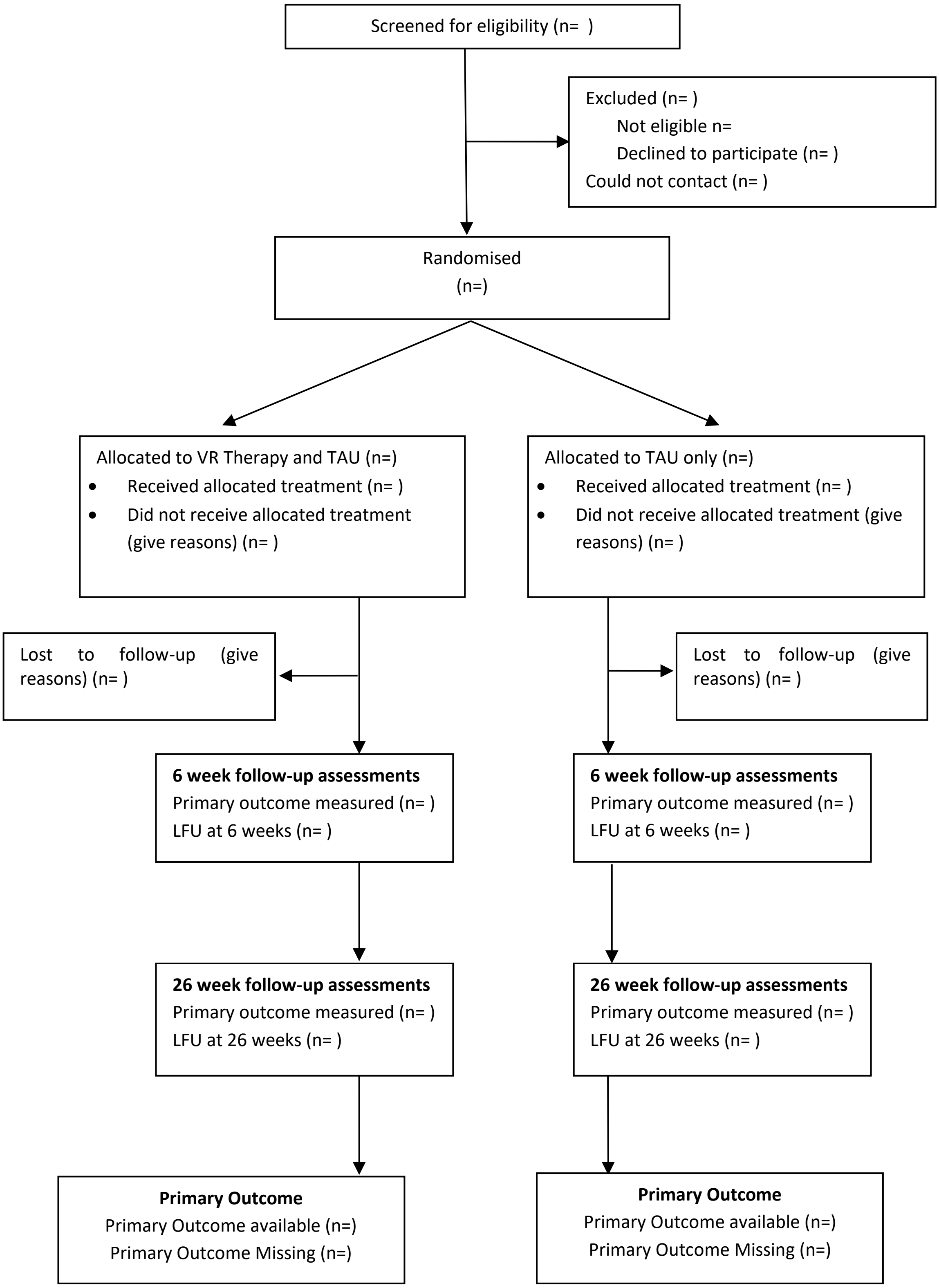

Primary Care Clinical Trials Unit

STATISTICAL ANALYSIS REPORT

gameChange
Improving lives through VR therapy

The gameChange Trial: A randomised controlled trial testing automated virtual reality cognitive therapy for patients with fears in everyday situations

CONFIDENTIAL

Version 3.0

14 February 2022

	NAME	TITLE	SIGNATURE	DATE
Written by:	Ushma Galal	Trial Statistician		14/02/2022
Reviewed by:	Nicola Williams	Senior Trial Statistician		15/02/2022
Reviewed by:	Ly-Mee Yu	Lead Trial Statistician		14/02/2022
Reviewed by:	Victoria Harris (mediation analysis)	Trial Statistician		15/02/2022
Approved by:	Daniel Freeman	Chief Investigator		15/02/2022

Version History

Version:	Version Date:	Changes:
0.1	October 2021	Original draft
0.2	5 November 2021	Updated tables
0.3	6 November 2021	Updated post-comments from LMY
0.4	11 November 2021	Updated post-comments from NW. Updated subgroup results with interaction effects rather than treatment differences
0.5	12 November 2021	Updated with corrected medication data from new download (DC5)
0.6	12 November 2021	O-AS and O-BAT avoidance scores updated with correct coding. Relevant tables updated here
0.7	21 November 2021	O-BAT avoidance analysis updated to mixed models, additional subgroup analysis at 26 weeks added to section 3.11, results in section 3.10 corrected
0.8	22 November 2021	Final draft version – add VH to reviewer list
1.0	22 November 2021	Signed off blinded version
1.1	23 November 2021	Unblinded draft with corrected mediation results, additional CACE tables, additional sensitivity analysis removing outliers, missing values added to Table 42: *Distribution of treatment deliverer across sites*

1.2	25 November 2021	Updates after comments from LMY. Addition of baseline tables for pre-post COVID lockdown populations in Appendix IX
1.3	6 December 2021	Correcting typos, censoring inpatient admission in section 3.10.2
2.0	6 December 2021	Signed off unblinded version
2.1	14 December 2021	Added post-hoc analysis by Victoria Harris
2.2	15 December 2021	Updated post-hoc analysis
2.3	06 January 2022	UG - Updated post-hoc analysis tables with summary stats; updated tables 32/33 ("avoidance" replaced with "distress")
2.4	14 February 2022	Updated with 2 AEs missing in error

Table of Contents

1 Contents

Table of Contents 94

List of tables 96

List of Figures 98

1 Introduction 99

1.1 Validation 99

1.2 Software employed 101

2 Methods 101

2.1 Background Information 101

2.2 Trial/Study design 102

2.3 Objectives 102

2.4 Target population 104

1.1.1 INCLUSION CRITERIA 104

1.1.2 EXCLUSION CRITERIA 104

2.5 Interventions 105

2.6 Outcomes measures 105

2.6.1 Primary outcome 105

2.6.2 Secondary outcomes 105

2.7 Sample size 106

2.8 Randomisation and blinding in the analysis stage 106

2.9 Data cleaning 107

2.10 Analysis for Data Monitoring Committee meetings 107

2.11 Definition of population for analysis 107

2.12 Deviation from SAP 107

3 Results 108

3.1 Representativeness o Study Sample and Patient Throughput 108

3.2 Recruitment 110

3.3 Baseline characteristics of participants 110

3.4 Number analysed 119

3.4.1 Predictors of missing primary outcome data 121

3.5 Primary & Secondary Outcomes Analyses 122

3.6 Mediation analysis 126

3.6.1 O-AS Avoidance Score 128

3.6.2 O-AS Distress Score 131

3.7 Moderation/Subgroup analyses 134
3.8 Sensitivity analyses 137
3.8.1 O-AS Avoidance Score 137
3.8.2 O-AS Distress Score 144
3.9 Safety analyses 151
3.10 Additional exploratory analysis not specified in the protocol 155
3.10.1 Medications 155
3.10.2 Service use 158
3.10.3 Standard care 161
3.11 Additional exploratory analysis not specified in the SAP 163
3.11.1 O-AS Avoidance and Distress by Treatment deliverer 163
3.11.2 O-AS Avoidance and Distress by Site 163
3.11.3 Additional subgroup analyses 165
3.11.4 Post-hoc subgroup of secondary outcomes by O-AS at baseline 169
4 References 183
5 Appendices184
5.1 Appendix I. O-BAT Summary Tables 184
5.2 Appendix II. Patient Health Questionnaire [PHQ-9] - categories 190
5.3 Appendix III. C-SSRS Summary Tables 191
5.4 Appendix IV. Recovering quality of life - physical health 192
5.5 Appendix V. Missingness Investigation 194
5.5.1 O-AS Avoidance Score 194
5.5.2 O-AS Distress Score 202
5.6 Appendix VI. Treatment deliverer 209
5.7 Appendix VII. Baseline tables for CACE analysis population 210
5.8 Appendix VIII. Diagnostic plots 218
5.9 Appendix VIX. Baseline tables for sensitivity analyses 224
5.9.1 Pre-COVID-19 Lockdown 224
5.9.2 COVID-19 Lockdown 232

CONFIDENTIAL

List of tables

Table 1: Baseline characteristics by randomised group 110

Table 2 Completion of follow-up assessments, withdrawals, and lost to follow-up over the study period...... 119

Table 3: Association between randomised group and availability of (pro-rated) primary outcomes 121

Table 4: Summary statistics for the primary and secondary outcomes and the treatment difference between the randomised groups 122

Table 5: Mediation analysis on O-AS Avoidance using mixed-effects models 128

Table 6: Mediation analysis on O-AS Distress using mixed-effects models 131

Table 7: Summary statistics and results for the subgroup analysis on O-AS Avoidance & Distress 135

Table 8: Missingness pattern for O-AS AVOIDANCE at the follow-up time points, by randomised group 137

Table 9: Sensitivity analysis using MNAR assumption for the treatment effect on O-AS Avoidance at 6 weeks139

Table 10: Summary statistics for the sensitivity analyses and the treatment difference between the randomised groups 140

Table 11: Estimates of complier average causal effects 142

Table 12 Summary of compliance for O-AS Avoidance score at 6 weeks 142

Table 13: Missingness pattern for O-AS Distress at the follow-up time points, by randomised group 144

Table 14: Sensitivity analysis using MNAR assumption for the treatment effect on O-AS DIstress at 6 weeks 146

Table 15: Summary statistics for the sensitivity analyses and the treatment difference between the randomised groups 147

Table 16: Estimates of complier average causal effects 149

Table 17 Summary of compliance for O-AS Distress score at 6 weeks 149

Table 18: Summary of Safety Events by randomised group 151

Table 19: Summary of SAEs per participant 152

Table 20: MedDRA coded SAE Frequency Table 153

Table 21: Summary statistics for medications, by randomised group 155

Table 22: Summary statistics for service use, by randomised groups 158

Table 23: Summary of standard care (i.e. psychological therapy) by randomised group 161

Table 24: Summary of primary outcomes at 6 weeks by treatment deliverer 163

Table 25: Summary of primary outcomes at 6 weeks by site 163

Table 26: Further results for the subgroup analysis on O-AS Avoidance at 6 weeks 165

Table 27: Further results for the subgroup analysis on O-AS Avoidance at 26 weeks 165

Table 28: Further results for the subgroup analysis on O-AS Distress at 6 weeks 166

Table 29: Further results for the subgroup analysis on O-AS Distress at 26 weeks 167

Table 30 Further results for the subgroup analysis on Secondary outcomes at 6 weeks by O-AS avoidance... 171

Table 31 Further results for the subgroup analysis on Secondary outcomes at 26 weeks by O-AS avoidance. 175

Table 32 Further results for the subgroup analysis on Secondary outcomes at 6 weeks by O-AS distress....... 179

Table 33 Further results for the subgroup analysis on Secondary outcomes at 26 weeks by O-AS distress..... 181

Table 34: Summary of steps completed for the real world distress outcome (O-BAT), by randomised group. 184

Table 35: Summary of highest step completed for the real world distress outcome (O-BAT), by randomised group 187

Table 36: Categories of PHQ-9 total score by randomised group 190

Table 37: Summary statistics for the C-SSRS Intensity of Ideation, Suicidal Behaviour and Preparatory acts or behaviour scales, by randomised groups 191

Table 38: Categories of ReQOL-21 by randomised group 192

Table 39: Baseline characteristics of participants by completeness for O-AS Avoidance at 6 weeks 194

Table 40: Baseline characteristics of participants by completeness for O-AS Distress at 6 weeks 202

Table 41: Distribution of treatment deliverer across sites 209

Table 42: Baseline characteristics by compliance 210

Table 43: Baseline characteristics for the pre-lockdown population, by randomised group 224

Table 44: Baseline characteristics for the lockdown population, by randomised group 232

List of Figures

Figure 1: Participant Flow Diagram 109

Figure 2 Pattern mixture model results for O-AS Avoidance at 6 weeks 138

Figure 3 Pattern mixture model results for O-AS Distress at 6 weeks 145

Figure 4: Histograms and model residual plots for O-AS Avoidance 218

Figure 5: Histograms and model residual plots for O-AS distress 218

Figure 6: Histograms and model residual plots for ACTIGRAPHY 218

Figure 7 Histograms and model residual plots for the TIME BUDGET 219

Figure 8 Histograms and model residual plots for the AGORAPHOBIA MOBILITY INVENTORY-AVOIDANCE [AMI-A] 219

Figure 9 Histogram for the O-BAT number steps avoided 219

Figure 10 Histograms and model residual plots for the O-BAT mean distress score 220

Figure 11 Histograms and model residual plots for the REVISED-GREEN ET AL PARANOID THOUGHTS SCALE [R-GPTS] part A 220

Figure 12 Histograms and model residual plots for the REVISED-GREEN ET AL PARANOID THOUGHTS SCALE [R-GPTS] part B 220

Figure 13 Histograms and model residual plots for the REVISED-GREEN ET AL PARANOID THOUGHTS SCALE [R-GPTS] overall score 221

Figure 14 Histograms and model residual plots for the PARANOIA WORRIES QUESTIONNAIRE [PWQ] 221

Figure 15 Histograms and model residual plots for PATIENT HEALTH QUESTIONNAIRE (DEPRESSION, ANXIETY AND STRESS SCALE) [PHQ-9] 221

Figure 16 Histograms and model residual plots for the COLUMBIA SUICIDE SEVERITY RATING SCALE [C-SSRS]222

Figure 17 Histograms and model residual plots for the EQ-5D-5L INDEX 222

Figure 18 Histograms and model residual plots for the EQ-5D-5L VAS score 222

Figure 19 Histograms and model residual plots for RECOVERING QUALITY OF LIFE [REQOL-20] 223

Figure 20 Histograms and model residual plots for QUESTIONNAIRE ON PROGRESS OF RECOVERY [QPR] 223

1 Introduction

This document details the analysis for the main paper(s) reporting results from the NHS National Institute of Health Research (NIHR) i4i programme and the NIHR Oxford Health Biomedical Research Centre funded Multicentre Randomised Controlled Trial of a virtual reality (VR) therapy in the treatment/management of psychosis. The results reported in this document follow the strategy set out in the statistical analysis plan. Subsequent analyses of a more exploratory nature will not be bound by this strategy, though they are expected to follow the broad principles laid down there.

The analysis strategy will be available on request when the principal papers are submitted for publication in a journal. Suggestions for subsequent analyses by journal editors or referees, will be considered carefully, and carried out as far as possible in line with the principles of this analysis strategy; if reported, the source of the suggestion will be acknowledged.

This report is based on the statistical analysis plan 'gameChange SAP v1.0 (02Nov2021)'. Any deviations from the statistical analysis plan will be described and justified in this report of the trial.

Trial statistician:

Ushma Galal***:*** ushma.galal@phc.o.ac.uk

Validation statistician(s):

Nicola Williams: Nicola.williams@phc.ox.ac.uk

Ly-Mee Yu: ly-mee.yu@phc.ox.ac.uk@phc.o.ac.uk

Chief Investigator:

Professor Daniel Freeman***:*** daniel.freeman@psych.ox.ac.uk

Trial/Study Managers:

Ariane Petit: ariane.petit@psych.ox.ac.uk

Sinéad Lambe: sinead.lambe@psych.ox.ac.uk

Data Manager:

Jenna Grabey:

1.1 Validation

Validation of results presented in this report was conducted by Nicola Williams. All results/major endpoints/primary endpoint were validated by independent programming using STATA 16. Results

from Stata output were checked for transcription errors. Further details of validation including validation programs are saved on the PC-CTU restricted drive in the project folder in the subfolder "STATS\4. Analysis\6.Validation -Name of Validater".

1.2 Software employed

Stata (SE) version 16.1 SE was used for all analyses.

2 Methods

2.1 Background Information

Too many patients with psychosis, despite standard treatment, become isolated and inactive, with negative effects on both mental and physical health. Approximately 80% of patients with schizophrenia experience an episode of depression (Upthegrove et al, 2017). Physical activity levels in patients with schizophrenia are reduced on average by approximately two thirds (Lindamer et al, 2008). Over 90% of patients with schizophrenia are unemployed and spend “less time in functional but also in social and leisure activities and more time resting and ‘doing nothing’ compared to the general population” (Cella et al, 2016). Life expectancy is on average 14.5 years shorter (Hjorthøj et al, 2017), due to largely preventable conditions such as high blood pressure, diabetes, and heart disease. Partly this physical ill health reflects unhealthy lifestyles including inactivity. Our view is that a substantial part of this inactivity arises from avoidance due to anxiety. In a clinical assessment study of 1800 patients with non-affective psychosis, two-thirds of the patients had levels of anxious avoidance equivalent to patients diagnosed with agoraphobia (Freeman et al, 2019). The anxiety in patients with psychosis can arise from a number of sources: fears that others will harm them, voices telling them of danger, social anxiety fears of humiliation and rejection, and negative beliefs about the self that cause a lack of confidence and a sense of vulnerability.

Virtual reality (interactive computer-generated environments) has been used since the early 1990’s to treat anxiety (Rothbaum al, 1994). Meta-analyses indicate that VR treatments for anxiety disorders can produce large treatment effects (Carl et al, 2018) that generalise to the real world (Morina et al, 2015). Previous uses of VR for mental health problems have all depended on a therapist providing the psychological therapy (Freeman, Reeve et al, 2017). Furthermore, automated treatment has the potential to be scalable, removing a key cause of the highly limited access to psychological therapy for patients with psychosis. VR may also be especially suited to the difficulties of patients with psychosis. Patients with strong fears are much more likely to test out their fear expectations in VR because they know it is a simulation but the learning that they make then transfers to the real world (Morina et al, 2015). VR treatment can also include engaging tasks that make the treatment experience much more pleasurable. A graded approach can easily be applied in VR, allowing the individual to repeatedly experience the situations they find difficult and make new learning. The view is that VR treatments have the potential to be faster, more efficacious, and appealing to patients than traditional face-to-face approaches.

The chief investigator and colleagues have developed - using a socially-inclusive design process - a new automated VR cognitive treatment for patients with psychosis having difficulties being in everyday social situations. It is designed to be easy to use, engaging for patients and staff, and delivered with

the latest consumer equipment. Therefore this VR treatment has the potential to be widely implemented in treatment services. Psychological treatment that involves direct coaching in the situations that trouble patients with psychosis is rarely available in mental health services. This study sets out to determine the clinical effects of the VR treatment on real-world performance, activity levels, psychiatric symptoms, and quality of life.

2.2 Trial/Study design

The design is a multicentre, parallel group randomised controlled trial with single blind assessment to test whether the automated VR cognitive treatment added to treatment as usual, compared to treatment as usual alone, leads to a post-treatment reduction in real world distress and avoidance for patients with psychosis attending NHS mental health services. Treatment as usual will be measured but remain as usual in both groups. Assessments will be carried out at 0 (baseline), 6 (post treatment), and 26 (follow-up) weeks by a researcher blind to treatment allocation.

Pre-COVID period: For all outcomes, the 6 week and 26 week assessments can be conducted up to 10 weeks after the assessment due date, i.e. up to 6+10 weeks post-randomisation for the 6 week assessments and 26+10 weeks for the 26 week assessments (although the aim is to conduct these assessments at 6 and 26 weeks). If it is not possible to complete the follow-up assessments within these windows, it will be considered missing data.

Shortened follow-up during the COVID-19 pandemic: In addition to the above, for participants who were randomised in the last four months of recruitment, the 26-week assessments will be brought forward by up to 6 weeks i.e. they will be conducted from 20 weeks post-randomisation. This is to ensure data collection is completed within the study schedule.

2.3 Objectives

The primary research question is: Does automated VR cognitive treatment added to treatment as usual, compared to treatment as usual alone, lead to a post-treatment reduction in real world distress and avoidance for patients with psychosis attending NHS mental health services?

The primary hypothesis is that:

1. Compared to treatment as usual, VR cognitive therapy added to treatment as usual will reduce distress and avoidance of real world situations (post treatment).

The secondary hypotheses are:

1. Compared to treatment as usual, VR cognitive therapy added to treatment as usual will reduce psychiatric symptoms (paranoia, anxious avoidance, depression, and suicidal ideation), increase activity, and improve quality of life (post-treatment).

2. Treatment effects will be maintained at follow-up.

3. The mediators of VR treatment will be safety beliefs, threat cognitions, and defence behaviours.

4. Treatment effects will be moderated by the occurrence of negative auditory hallucinations in social situations, hopelessness, appearance concerns, and threat cognitions.

2.4 Target population

The trial participants are patients with psychosis and self-reported difficulties going outside among other people primarily due to anxiety (assessed using a screening version of the Oxford – Behavioural Assessment Task (O-BAT) self-report questionnaire).

13.2.1 Inclusion Criteria

- Participant was willing and able to give informed consent for participation in the trial.
- Aged 16 years or above.
- Attending a NHS mental health trust for the treatment of psychosis.
- Had a clinical diagnosis of schizophrenia spectrum psychosis (F20-29) or an affective diagnosis with psychotic symptoms (F31.2, 31.5, 32.3, 33.3) (ICD-10, WHO, 2010).
- Had self-reported difficulties going outside their home primarily due to anxiety (and hence would score on the primary outcome) that they would like treated.

13.2.2 Exclusion Criteria

The participant could not enter the trial if ANY of the following applied:

- Unable to attempt an Oxford – Behavioural Task (O-BAT) at baseline (e.g. due to being unpermitted to leave a psychiatric ward).
- Photosensitive epilepsy.
- Significant visual, auditory, or balance impairment.
- Current receipt of another intensive psychological therapy (or about to start it within the 6-week trial therapy window).
- Insufficient comprehension of English.
- In forensic settings or Psychiatric Intensive Care Unit (PICU).
- Organic syndrome.
- Primary diagnosis of alcohol or substance disorder or personality disorder.
- Significant learning disability.
- Current active suicidal plans.
- A participant could also not enter the trial if there was another factor, which, in the judgement of the investigator, would preclude the participant from providing informed consent or from safely engaging with the trial procedures. Recruitment was to be suspended for people who had any of the conditions that would make them high or moderate risk (clinically vulnerable) for a severe course of COVID-19 (https://www.nhs.uk/conditions/coronavirus-covid-19/people-at-higher-risk/whos-at-higher-risk-from-coronavirus/). However, people who were at moderate or high risk for a severe course of COVID-19 were able to join the trial if they had received the COVID-19 vaccine (subject to medical advice). Reason for exclusion were recorded in line with CONSORT 2010 Statement (Schulz et al, 2010).

2.5 Interventions

The intervention was a new automated Virtual Reality (VR) cognitive treatment, added to treatment as usual, for patients with psychosis having difficulties being in everyday social situations. VR therapy was designed to be easy to use, engaging for patients and staff, and delivered with the latest consumer equipment. It allows an individual to repeatedly experience computer simulations of the situations they find difficult. Psychological treatment that involves direct coaching in the situations that trouble patients with psychosis is rarely available in mental health services so participants in this study had a virtual coach to help them overcome their fears. This study set out to determine the clinical effects of the VR treatment on real-world performance, activity levels, psychiatric symptoms, and quality of life.

Participants allocated to the control arm continued to receive their usual care and the research team offered no additional interventions.

2.6 Outcomes measures

Outcomes were assessed at 6 weeks and 26 weeks. The primary outcome is the effect of the intervention at 6 weeks. However, it was also of interest to compare the linear trend between the two groups and to consider the longer-term effect of the intervention (26 weeks).

2.6.1 Primary outcome

The primary outcome measure is the Oxford Agoraphobic Avoidance Scale (O-AS) (Lambe et al., 2021) which is assessed at weeks 0, 6 and 26. The primary endpoint for analysis is 6 weeks.

2.6.2 Secondary outcomes

2. Clinical improvements in activity levels, psychiatric symptoms and quality of life will be assessed by the following at 0, 6, and 26 weeks:
    - Activity levels: assessed by Actigraphy and a time-budget measure (Jolley et al., 2006).
    - Psychiatric symptoms: assessed by the:
        a. Agoraphobia Mobility Inventory-Avoidance [AMI-A] (Chambless et al., 1985),
        b. Real-world avoidance and distress [O-BAT] (Freeman et al., 2016),
        c. Revised-Green et al Paranoid Thoughts Scale [R-GPTS] (Green et al., 2008; Freeman et al, 2019);
        d. Paranoia Worries Questionnaire [PWQ] (Freeman et al, 2019),
        e. Patient Health Questionnaire [PHQ-9] (Kroenke et al, 2001),
        f. Columbia Suicide Severity Rating Scale [C-SSRS] (Posner et al., 2011).
5. Quality of life will be assessed using the following tools:
    a. EQ-5D-5L (http://www.euroqol.org/). The EQ-5D-5L index will be calculated using the cross walk method. This index and the VAS score will be summarised and compared between groups,
    b. ReQol,
    c. Progress of recovery [QPR].
6. The following Mediation variables will be assessed at weeks 0, 6 & 26:

a. Cognition and use of defence behaviours will be assessed using Parts 1 (cognitions) and 3 (defences) of the Oxford Cognitions and Defences Questionnaire [O-CDBQ] (Rosebrock et al, submitted),
b. Strength of safety beliefs, vulnerability belief, and threat anticipation assessed using three visual analogue scales (Freeman et al., 2016).

7. The following Moderator variables will be assessed at 0 weeks and tested for the moderation of the primary outcome at 6 weeks:
    a. Hallucinations Scale ,
    b. Beck Hopelessness Scale [BHS] (Beck, 1988),
    c. Body-esteem Scale for Adolescents and Adults (Mendelson et al, 2001),
    d. Part 1 of the Oxford Cognitions and Defences Questionnaire [O-CDBQ] (Rosebrock et al, submitted).

2.7 Sample size

Approximately 432 participants were to be recruited into this trial, with 216 in each arm. This sample size takes into consideration a maximum attrition rate of 20%, and provides 90% power to detect a difference of around 8 (standard deviation =23) in O-BAT score, from randomisation to 6 weeks (i.e. standardised effect size of 0.35) at 5% level of significance (2-sided).

2.8 Randomisation and blinding in the analysis stage

Participants were randomised once they had completed the baseline assessment. Participants were allocated to one of the trial arms using a 1:1 allocation ratio. Randomisation was carried out by a validated online system, Sortition, designed by the University of Oxford Primary Care Clinical Trials Unit. Randomisation using a permuted blocks algorithm, with randomly varying block size, was stratified by site (Bristol/Manchester/Newcastle/Nottingham/Oxford) and service type (in-patient/early intervention/community mental health team).

The research assessors were blinded to group allocation, but the patients and staff member present were not (they cannot be blinded to what psychological intervention is delivered or received). The staff members setting up and running the VR software informed patients of the randomisation outcome, to ensure the research assessors remain blinded to group allocation. Precautionary strategies to prevent un-blinding of allocation includes the staff member and assessor considering room use and booking arrangements; patients being reminded by the assessor not to talk about their allocation result; and, after the initial assessment, the assessor not looking at the patient's clinical notes. The statisticians were blind to allocation.

2.9 Data cleaning

Day to day data management was conducted by the PC-CTU data management team. Additional data cleaning was also carried out by the statistics team and these files were saved on a restricted folder, “K:\HB_O\gameChange\STATS\10. Data Cleaning”.

- Missing O-AS scores were pro-rated as per the SAP such that if one or two item scores were missing then the total score was inflated by the number of missing items*mean of the remaining items. If more than 2 items were missing, the total score for that participant could not be determined and was treated as missing data
- The O-BAT and O-AS Avoidance measures were recoded so that 'Yes I can do this now' = 0 and 'No, I can’t do this' = 1. This was so that increased scores implied increase avoidance
- Body esteem scale - As the original questionnaire is scaled from 0-4, the observed data (scaled from 1-5) needed to be changed to this scale first to match the original
- Time to inpatient admission and physical health admission was censored at 182.5 days for the 6-months prior to trial entry, at 42 days (6+10 weeks) for the time between baseline and the 6-week visit and at 140 days for time from 6 week to 26 week visit

2.10 Analysis for Data Monitoring Committee meetings

A Data Monitoring and Ethics Committee (DMEC) was formed to monitor the safety and progress of the trial. All details of analysis for DMC meetings are stored on a restricted folder, “K:\HB_O\gameChange\STATS\4. TSC and DMC\DMC\”.

2.11 Definition of population for analysis

After randomisation, all participants for whom data are available will be analysed according to their allocated intervention group, irrespective of what intervention they actually receive.

2.12 Deviation from SAP

- For the primary outcomes analysis, the SAP states that: “Missing data on individual measures will be pro-rated if more than 75% of the items are completed; otherwise, the measure will be considered as missing.” This was intended to state that the primary outcome would be pro-rated if **75% or more** of the items were completed and the latter definition was the one used for the analysis.
- The aim of a subgroup analysis is to see if the differences between the treatment groups for the primary outcome of interest change with levels of the moderator. Thus, in order to get

meaningful results, the moderator has to be present in both treatment groups. As treatment deliverer only applied to the VR+TAU group, a subgroup analysis on this would not have made sense and was thus omitted.

- Rather than assessing the maximum number of steps completed on the O-BAT, the total number of steps avoided was used in the analysis.

3 Results

3.1 Representativeness o Study Sample and Patient Throughput

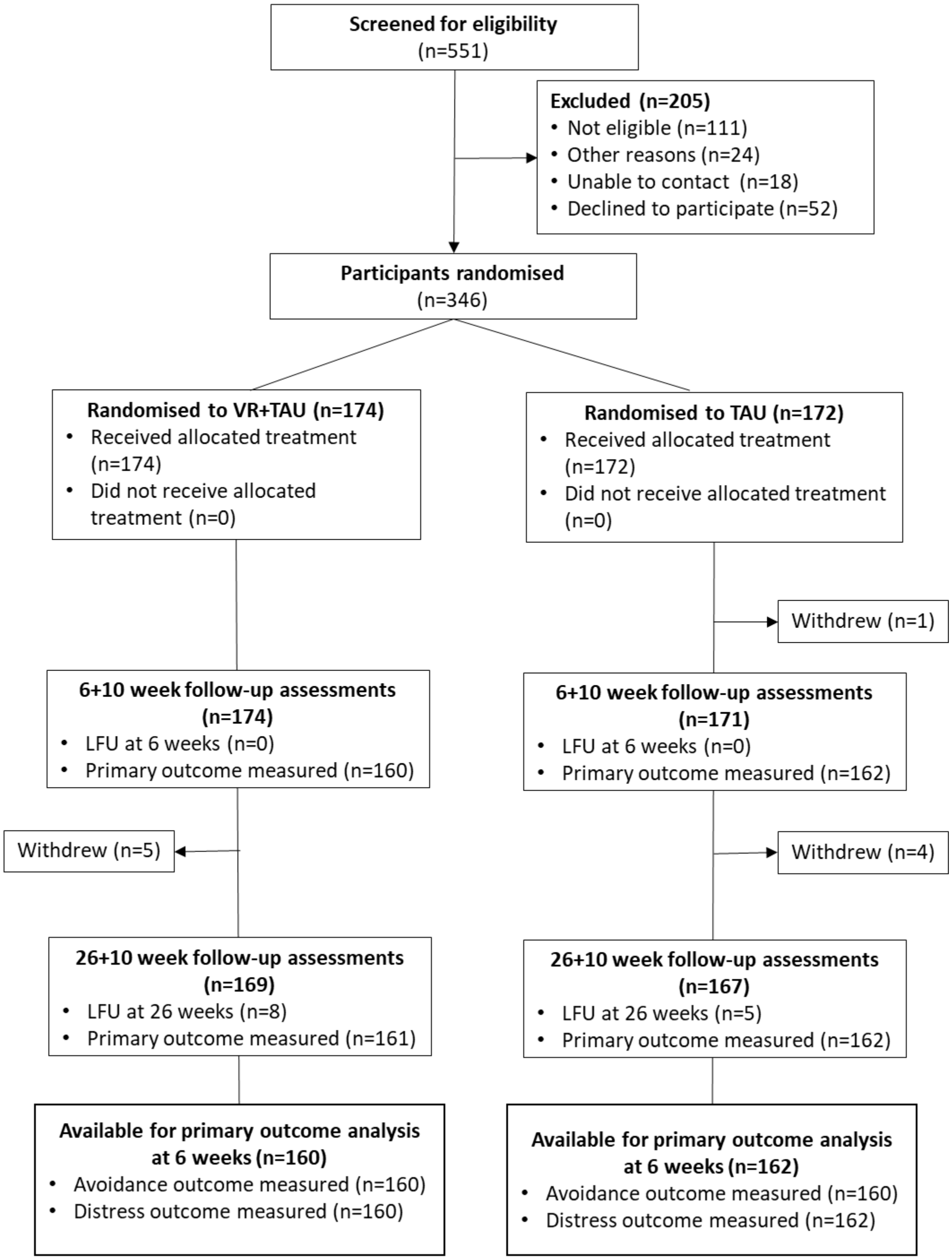

FIGURE 2: PARTICIPANT FLOW DIAGRAM

3.2 Recruitment

The first participant was randomised on 25 July 2019. The trial closed to recruitment on 07/05/2021 with 346 participants randomized.

The trial was not stopped early.

3.3 Baseline characteristics of participants

TABLE 2: BASELINE CHARACTERISTICS BY RANDOMISED GROUP

	VR+TAU (N=174)	TAU (N=172)	Total (N=346)
**Age (years)**			
Mean (SD)	36.6 (12.8)	37.8 (12.2)	37.2 (12.5)
Median (IQR)	35.1 (25.6 to 45.2)	36.1 (28.0 to 46.1)	35.8 (27.1 to 45.6)
Min to Max	17.1 to 69.1	16.6 to 70.7	16.6 to 70.7
Missing	-	-	-
**Age at first contact with mental health services (years)**			
Mean (SD)	24.6 (9.7)	26.2 (11.1)	25.4 (10.4)
Median (IQR)	22.0 (18.0 to 28.0)	23.0 (18.0 to 32.0)	23.0 (18.0 to 31.0)
Min to Max	6.0 to 59.0	7.0 to 60.0	6.0 to 60.0
Missing	3	7	10
**Sex†, n (%)**			
Female	58 (33.3%)	53 (30.8%)	111 (32.1%)
Male	116 (66.7%)	115 (66.9%)	231 (66.8%)
Other	0 (0.0%)	1 (0.6%)	1 (0.3%)
Prefer not to say	0 (0.0%)	2 (1.2%)	2 (0.6%)
Missing	0 (0.0%)	1 (0.6%)	1 (0.3%)
**Current Marital status, n (%)**			

	VR+TAU (N=174)	TAU (N=172)	Total (N=346)
Single	131 (75.3%)	138 (80.2%)	269 (77.7%)
Married/civil partnership	21 (12.1%)	14 (8.1%)	35 (10.1%)
Cohabiting	6 (3.4%)	10 (5.8%)	16 (4.6%)
Separated	2 (1.1%)	1 (0.6%)	3 (0.9%)
Divorced	9 (5.2%)	7 (4.1%)	16 (4.6%)
Widowed	3 (1.7%)	1 (0.6%)	4 (1.2%)
Missing	2 (1.1%)	1 (0.6%)	3 (0.9%)
**Ethnic group, n (%)**			
White	152 (87.4%)	142 (82.6%)	294 (85.0%)
Black British	1 (0.6%)	1 (0.6%)	2 (0.6%)
Black African	1 (0.6%)	2 (1.2%)	3 (0.9%)
Black Caribbean	0 (0.0%)	4 (2.3%)	4 (1.2%)
Indian	0 (0.0%)	2 (1.2%)	2 (0.6%)
Black Other	1 (0.6%)	0 (0.0%)	1 (0.3%)
Pakistani	3 (1.7%)	3 (1.7%)	6 (1.7%)
Other	16 (9.2%)	17 (9.9%)	33 (9.5%)
Missing	0 (0.0%)	1 (0.6%)	1 (0.3%)
**Site[1], n (%)**			
Bristol	37 (21.3%)	37 (21.5%)	74 (21.4%)
Manchester	29 (16.7%)	29 (16.9%)	58 (16.8%)
Newcastle	42 (24.1%)	39 (22.7%)	81 (23.4%)
Nottingham	32 (18.4%)	31 (18.0%)	63 (18.2%)
Oxford	34 (19.5%)	36 (20.9%)	70 (20.2%)

	VR+TAU (N=174)	TAU (N=172)	Total (N=346)
**Service type[1], n (%)**			
Community MH team	107 (61.5%)	102 (59.3%)	209 (60.4%)
Early intervention	64 (36.8%)	69 (40.1%)	133 (38.4%)
In-patient	3 (1.7%)	1 (0.6%)	4 (1.2%)
**Employment, n (%)**			
Employed full-time (paid), n (%)	10 (5.7)	9 (5.2)	19 (5.5)
Employed part-time (paid), n (%)	4 (2.3)	4 (2.3)	8 (2.3)
Employed full-time (voluntary), n (%)	-	-	-
Employed part-time (voluntary), n (%)	2 (1.1)	3 (1.7)	5 (1.4)
Unemployed (on benefits), n (%)	112 (64.4)	122 (70.9)	234 (67.6)
Unemployed (not on benefits), n (%)	8 (4.6)	5 (2.9)	13 (3.8)
Student or in training full-time, n (%)	5 (2.9)	6 (3.5)	11 (3.2)
Student or in training part-time, n (%)	3 (1.7)	1 (0.6)	4 (1.2)
Self-employed, n (%)	4 (2.3)	1 (0.6)	5 (1.4)
Home-maker, n (%)	2 (1.1)	1 (0.6)	3 (0.9)
Carer, n (%)	1 (0.6)	1 (0.6)	2 (0.6)
Retired, n (%)	5 (2.9)	2 (1.2)	7 (2.0)
Other, n (%)	-	3 (1.7)	3 (0.9)
Missing, n (%)	18 (10.3)	14 (8.1)	32 (9.2)

	VR+TAU (N=174)	TAU (N=172)	Total (N=346)
**Usual/Normal living arrangement, n (%)**			
Living alone (+/- children), n (%)	72 (41.4)	72 (41.9)	144 (41.6)
Living with husband/wife, n (%)	16 (9.2)	13 (7.6)	29 (8.4)
Living with partner, n (%)	8 (4.6)	9 (5.2)	17 (4.9)
Living with parents, n (%)	40 (23.0)	42 (24.4)	82 (23.7)
Living with other relatives, n (%)	9 (5.2)	10 (5.8)	19 (5.5)
Living with others (e.g. friends), n (%)	10 (5.7)	11 (6.4)	21 (6.1)
Missing, n (%)	19 (10.9)	15 (8.7)	34 (9.8)
**Mental health diagnosis (F-code), n (%)**			
20, n (%)	32 (18.4)	27 (15.7)	59 (17.1)
20.0, n (%)	25 (14.4)	29 (16.9)	54 (15.6)
20.5, n (%)	-	1 (0.6)	1 (0.3)
20.8, n (%)	-	1 (0.6)	1 (0.3)
20.9, n (%)	17 (9.8)	6 (3.5)	23 (6.6)
21, n (%)	1 (0.6)	2 (1.2)	3 (0.9)
22, n (%)	1 (0.6)	2 (1.2)	3 (0.9)
22.8, n (%)	1 (0.6)	-	1 (0.3)
23, n (%)	-	2 (1.2)	2 (0.6)
23.1, n (%)	2 (1.1)	2 (1.2)	4 (1.2)
23.2, n (%)	-	1 (0.6)	1 (0.3)
23.9, n (%)	3 (1.7)	4 (2.3)	7 (2.0)
25, n (%)	3 (1.7)	2 (1.2)	5 (1.4)

	VR+TAU (N=174)	TAU (N=172)	Total (N=346)
25.0, n (%)	2 (1.1)	-	2 (0.6)
25.1, n (%)	1 (0.6)	2 (1.2)	3 (0.9)
25.9, n (%)	9 (5.2)	7 (4.1)	16 (4.6)
28, n (%)	2 (1.1)	3 (1.7)	5 (1.4)
29, n (%)	57 (32.8)	54 (31.4)	111 (32.1)
29.0, n (%)	5 (2.9)	6 (3.5)	11 (3.2)
31.2, n (%)	1 (0.6)	4 (2.3)	5 (1.4)
31.4, n (%)	-	1 (0.6)	1 (0.3)
31.5, n (%)	2 (1.1)	-	2 (0.6)
32.3, n (%)	7 (4.0)	13 (7.6)	20 (5.8)
33.3, n (%)	3 (1.7)	3 (1.7)	6 (1.7)
**Currently taking any medication[2], n (%)**			
Yes, n (%)	169 (97.1)	166 (96.5)	335 (96.8)
No, n (%)	5 (2.9)	6 (3.5)	11 (3.2)
Missing, n (%)	-	-	-
**Type of medications in use[3]:**			
Antipsychotic			
Yes, n (%)	*161 (92.5)*	*156 (90.7)*	*317 (91.6)*
No, n (%)	*12 (6.9)*	*16 (9.3)*	*28 (8.1)*
Missing, n (%)	*1 (0.6)*	-	*1 (0.3)*
Antidepressant			
Yes, n (%)	*103 (59.2)*	*96 (55.8)*	*199 (57.5)*
No, n (%)	*70 (40.2)*	*76 (44.2)*	*146 (42.2)*

	VR+TAU (N=174)	TAU (N=172)	Total (N=346)
Missing, n (%)	*1 (0.6)*	-	*1 (0.3)*
Anxiolytic			
Yes, n (%)	*15 (8.6)*	*13 (7.6)*	*28 (8.1)*
No, n (%)	*157 (90.2)*	*159 (92.4)*	*316 (91.3)*
Missing, n (%)	*2 (1.1)*	-	*2 (0.6)*
Mood stabiliser			
Yes, n (%)	*18 (10.3)*	*15 (8.7)*	*33 (9.5)*
No, n (%)	*154 (88.5)*	*157 (91.3)*	*311 (89.9)*
Missing, n (%)	*2 (1.1)*	-	*2 (0.6)*
Hypnotic			
Yes, n (%)	*11 (6.3)*	*7 (4.1)*	*18 (5.2)*
No, n (%)	*161 (92.5)*	*165 (95.9)*	*326 (94.2)*
Missing, n (%)	*2 (1.1)*	-	*2 (0.6)*
Stimulant			
Yes, n (%)	*1 (0.6)*	-	*1 (0.3)*
No, n (%)	*171 (98.3)*	*172 (100.0)*	*343 (99.1)*
Missing, n (%)	*2 (1.1)*	-	*2 (0.6)*
**O-AS Avoidance score**			
Mean (SD)	3.2 (2.5)	3.4 (2.7)	3.3 (2.6)
Median (IQR)	3.0 (1.0 to 5.0)	3.0 (1.0 to 6.0)	3.0 (1.0 to 6.0)
Min to Max	0.0 to 8.0	0.0 to 8.0	0.0 to 8.0
Missing	1	-	1
**O-AS Distress score**			

	VR+TAU (N=174)	TAU (N=172)	Total (N=346)
Mean (SD)	51.4 (16.4)	52.6 (17.2)	52.0 (16.8)
Median (IQR)	53.0 (38.0 to 64.0)	55.0 (41.0 to 66.0)	53.5 (39.0 to 65.0)
Min to Max	4.0 to 80.0	10.0 to 80.0	4.0 to 80.0
Missing	-	-	-
**(Actigraphy) Mean number of steps**			
Mean (SD)	4727.4 (3016.6)	4942.9 (3107.3)	4831.6 (3054.3)
Median (IQR)	4265.4 (2795.1 to 6128.2)	4188.9 (2534.6 to 6805.0)	4210.9 (2623.7 to 6387.7)
Min to Max	42.3 to 14776.9	348.9 to 15054.7	42.3 to 15054.7
Missing	79	83	162
**Time budget score**			
Mean (SD)	51.9 (17.4)	53.2 (16.8)	52.5 (17.1)
Median (IQR)	50.0 (40.0 to 63.0)	53.5 (40.0 to 64.0)	51.0 (40.0 to 63.0)
Min to Max	7.0 to 100.0	11.0 to 95.0	7.0 to 100.0
Missing	23	30	53
**Agoraphobia Mobility Inventory-Avoidance (AMI-A) score**			
Mean (SD)	3.3 (0.7)	3.2 (0.8)	3.3 (0.8)
Median (IQR)	3.3 (2.9 to 3.7)	3.2 (2.6 to 3.8)	3.3 (2.8 to 3.7)
Min to Max	1.1 to 4.8	1.1 to 4.8	1.1 to 4.8
Missing	7	8	15
**O-BAT - maximum number of steps avoided**			
Mean (SD)	2.7 (1.3)	2.8 (1.3)	2.7 (1.3)
Median (IQR)	3.0 (2.0 to 4.0)	3.0 (2.0 to 4.0)	3.0 (2.0 to 4.0)
Min to Max	0.0 to 5.0	0.0 to 5.0	0.0 to 5.0

	VR+TAU (N=174)	TAU (N=172)	Total (N=346)
Missing	76	79	155
**O-BAT Mean distress score**			
Mean (SD)	5.5 (1.9)	5.5 (2.1)	5.5 (2.0)
Median (IQR)	5.5 (4.0 to 7.0)	5.6 (4.0 to 7.0)	5.6 (4.0 to 7.0)
Min to Max	1.0 to 10.0	0.0 to 10.0	0.0 to 10.0
Missing	77	80	157
**R-GPTS-A (social reference) score**			
Mean (SD)	14.1 (9.3)	12.6 (9.1)	13.3 (9.2)
Median (IQR)	14.0 (7.0 to 21.0)	11.0 (5.0 to 19.0)	13.0 (6.0 to 20.0)
Min to Max	0.0 to 32.0	0.0 to 32.0	0.0 to 32.0
Missing	16	11	27
**R-GPTS-B (persecution) score**			
Mean (SD)	17.3 (12.7)	14.2 (12.9)	15.7 (12.9)
Median (IQR)	17.0 (5.0 to 29.0)	10.0 (3.0 to 24.0)	14.0 (3.0 to 27.0)
Min to Max	0.0 to 40.0	0.0 to 40.0	0.0 to 40.0
Missing	16	11	27
**R-GPTS (overall) score**			
Mean (SD)	31.3 (20.7)	26.7 (20.8)	29.0 (20.8)
Median (IQR)	32.0 (14.0 to 47.0)	23.0 (8.0 to 44.0)	26.0 (10.0 to 47.0)
Min to Max	0.0 to 72.0	0.0 to 72.0	0.0 to 72.0
Missing	16	11	27
**Paranoia Worries Questionnaire (PWQ) total score**			
Mean (SD)	9.8 (6.2)	8.9 (6.2)	9.4 (6.2)

	VR+TAU (N=174)	TAU (N=172)	Total (N=346)
Median (IQR)	10.0 (5.0 to 15.0)	9.5 (3.0 to 14.0)	10.0 (4.0 to 15.0)
Min to Max	0.0 to 20.0	0.0 to 20.0	0.0 to 20.0
Missing	16	16	32
**Patient Health Questionnaire (PHQ-9) total score**			
Mean (SD)	15.1 (6.0)	14.1 (6.5)	14.6 (6.3)
Median (IQR)	15.0 (11.0 to 20.0)	15.0 (9.0 to 19.0)	15.0 (9.5 to 20.0)
Min to Max	2.0 to 27.0	2.0 to 27.0	2.0 to 27.0
Missing	8	10	18
**Columbia Suicide Severity Rating Scale (C-SSRS) total score**			
Mean (SD)	1.1 (1.3)	0.9 (1.3)	1.0 (1.3)
Median (IQR)	1.0 (0.0 to 2.0)	0.0 (0.0 to 2.0)	0.0 (0.0 to 2.0)
Min to Max	0.0 to 5.0	0.0 to 5.0	0.0 to 5.0
Missing	19	18	37
**EQ-5D-5L INDEX**			
Mean (SD)	0.5 (0.3)	0.5 (0.3)	0.5 (0.3)
Median (IQR)	0.6 (0.3 to 0.8)	0.6 (0.4 to 0.7)	0.6 (0.4 to 0.7)
Min to Max	-0.2 to 1.0	-0.3 to 1.0	-0.3 to 1.0
Missing	2	2	4
**EQ5D VAS score**			
Mean (SD)	51.6 (19.2)	53.2 (19.1)	52.4 (19.2)
Median (IQR)	50.0 (40.0 to 65.0)	50.0 (40.0 to 70.0)	50.0 (40.0 to 70.0)
Min to Max	0.0 to 90.0	6.0 to 95.0	0.0 to 95.0
Missing	3	2	5

	VR+TAU (N=174)	TAU (N=172)	Total (N=346)
**Recovering Quality Of Life (REQOL-20) total score**			
Mean (SD)	33.6 (13.3)	35.5 (13.2)	34.5 (13.2)
Median (IQR)	32.5 (23.0 to 44.0)	36.0 (26.0 to 44.0)	34.0 (25.0 to 44.0)
Min to Max	3.0 to 63.0	5.0 to 72.0	3.0 to 72.0
Missing	8	9	17
**Progress of Recovery (QPR) total score**			
Mean (SD)	27.2 (10.7)	28.1 (11.1)	27.6 (10.9)
Median (IQR)	27.0 (20.0 to 35.0)	29.0 (22.0 to 35.0)	28.0 (20.0 to 35.0)
Min to Max	0.0 to 51.0	0.0 to 56.0	0.0 to 56.0
Missing	1	2	3

† Missing value later identified as Male
[1] Stratification variables
[2] From baseline CRF & medical record data
[3] Not mutually exclusive

3.4 Number analysed

The frequency and percentage of participants completing follow-up assessments, withdrawing, and lost to follow-up are presented in Table 3 by randomised group and overall.

TABLE 3 COMPLETION OF FOLLOW-UP ASSESSMENTS, WITHDRAWALS, AND LOST TO FOLLOW-UP OVER THE STUDY PERIOD

	VR+TAU	TAU	Overall
**Randomised, n (%)**	174	172	346
**At least one 6-week (pro-rated) primary outcome score available, n (%)**	160 (92.0)	162 (94.2)	322 (93.1)
**At least one 26-week (pro-rated) primary outcome score available, n (%)**	157 (90.2)	161 (93.6)	318 (91.9)
**Withdrawn after randomisation, before 6 week follow-up, n (%)**	0	1 (0.6)	0
**Lost to follow-up before 6 week follow-up, n (%)**	0	0	0
**Withdrawn after 6 weeks but before 26 week follow-up, n (%)**	5 (2.9)	4 (2.3)	0
**Lost to follow-up after 6 weeks but before 26 week follow-up, n (%)**	8 (4.6)	5 (2.9)	0

3.4.1 Predictors of missing primary outcome data

Table 4 shows the association between randomised group and availability of the primary outcome. Here, 12 (7%) participants in the TAU only group and 14 (8 %) in the VR+TAU group did not have data to determine the pro-rated primary outcome O-AS Avoidance. The results show that there is no association between randomised group and availability of either of the primary outcomes.

TABLE 4: ASSOCIATION BETWEEN RANDOMISED GROUP AND AVAILABILITY OF (PRO-RATED) PRIMARY OUTCOMES

	VR+TAU (N=174)	TAU (N=172)	Odds ratio (95% CI)[1]	P value
**O-AS Avoidance score**				
**Missing, n (%)**	14 (8.0%)	12 (6.9%)	0.86 [0.38 to 1.91]	0.706
**Available, n (%)**	160 (93.0%)	160 (93.0%)		
**O-AS Distress score**				
**Missing, n (%)**	14 (8.0%)	10 (5.7%)	0.71 [0.30 to 1.63]	0.416
**Available, n (%)**	160 (93.0%)	162 (94.2%)		

[1] Logistic regression of the availability of the primary outcome for VR+TAU versus TAU only. Level of significance = 0.05

The comparisons of the baseline characteristics for participants who did not have primary outcome data vs. those who did, classified by treatment groups, are displayed in Table 40 & Table 41 in ***Appendix V.*** *Missingness Investigation*.

3.5 Primary & Secondary Outcomes Analyses

The primary objective was to compare the effect of treatment on avoidance and distress in everyday situations. This was assessed by the primary outcomes of self-rated avoidance and distress as measured by the Oxford Agoraphobic Avoidance Scale (O-AS), which was evaluated at baseline, 6 weeks and 26 weeks post randomisation. The primary endpoint is the 6-week time point.

Table 5 presents the results from the primary and secondary outcomes analyses. Regression diagnostic plots for these models are in ***Appendix VIII.*** *Diagnostic plots.* All the outcomes are analysed using linear mixed-effects models.

For the primary outcomes, the results show that, on average, there is a statistically significantly lower avoidance score (negative mean difference) and distress score (negative mean difference) in the VR+TAU group compared to TAU only at 6 weeks from randomisation. A similar trend is observed in the 26 weeks follow-up results though not statistically significant. There is no evidence of any treatment difference in the secondary outcomes apart from the O-BAT scores and progression of recovery at 6 months, though the number in the analysis is small for the O-BAT scores analysis so the result should be interpreted with caution.

TABLE 5: SUMMARY STATISTICS FOR THE PRIMARY AND SECONDARY OUTCOMES AND THE TREATMENT DIFFERENCE BETWEEN THE RANDOMISED GROUPS

	**VR+TAU (N=174)**	**TAU (N=172)**	**Adjusted treatment difference [95% CI][1]**	**P value**
**Primary outcomes**				
**O-AS Avoidance score, mean (SD) [n]**				
6 weeks	1.9 (2.2) [160]	2.5 (2.6) [160]	-0.47 [-0.88 to -0.06]; Std. effect: -0.18 [-0.34 to -0.02]	0.026
26 weeks	2.0 (2.3) [157]	2.5 (2.6) [159]	-0.37 [-0.78 to 0.05]; Std. effect: -0.14 [-0.30 to 0.02]	0.083
**O-AS Distress score, mean (SD) [n]**				
6 weeks	41.3 (18.8) [160]	45.8 (20.4) [162]	-4.33 [-7.78 to -0.87]; Std. effect: -0.26 [-0.46 to -0.05]	0.014

	VR+TAU (N=174)	TAU (N=172)	Adjusted treatment difference [95% CI][1]	P value
26 weeks	40.7 (20.6) [156]	43.9 (21.6) [161]	-2.50 [-5.98 to 0.97]; Std. effect: -0.15 [-0.36 to 0.06]	0.158
**Secondary outcomes**				
**(Actigraphy) Mean number of steps per day [2], mean (SD) [n]**				
6 weeks	5260.7 (3528.8) [57]	4717.4 (3647.5) [63]	578.7 [-333.8 to 1491.1]	0.214
26 weeks	5856.1 (2568.1) [26]	5603.9 (2838.3) [27]	751.1 [-533.6 to 2035.8]	0.252
**Time budget score[2], mean (SD) [n]**				
6 weeks	55.8 (16.0) [124]	57.6 (15.5) [119]	-1.75 [-4.73 to 1.23]	0.250
26 weeks	57.3 (18.2) [85]	57.7 (17.5) [85]	-1.01 [-4.55 to 2.52]	0.575
**Agoraphobia Mobility Inventory-Avoidance (AMI-A) score, mean (SD) [n]**				
6 weeks	2.9 (0.8) [152]	3.0 (0.9) [152]	-0.13 [-0.27 to 0.00]	0.055
26 weeks	2.9 (0.8) [146]	3.0 (0.9) [145]	-0.11 [-0.25 to 0.03]	0.117
**O-BAT - number of steps avoided[3], mean (SD) [n]**				
6 weeks	1.6 (1.7) [59]	2.3 (1.6) [55]	-0.89 [-1.38 to -0.39]	<0.001
26 weeks	1.1 (1.8) [9]	2.2 (2.1) [11]	-0.87 [-1.63 to -0.11]	0.025
**O-BAT Mean distress score of completed steps, mean (SD) [n]**				
6 weeks	3.1 (2.4) [55]	3.9 (2.6) [52]	-0.86 [-1.72 to 0.01]	0.052
26 weeks	2.4 (2.9) [8]	2.6 (2.8) [8]	-0.32 [-1.97 to 1.33]	0.705
**R-GPTS-A (social reference) score, mean (SD) [n]**				
6 weeks	10.6 (8.5) [142]	10.7 (8.4) [146]	-1.37 [-2.94 to 0.20]	0.087
26 weeks	11.0 (9.6) [133]	10.4 (9.1) [134]	-0.39 [-2.00 to 1.22]	0.637
**R-GPTS-B (persecution) score, mean (SD) [n]**				
6 weeks	13.0 (11.9) [142]	12.2 (12.6) [146]	-1.66 [-3.73 to 0.40]	0.115
26 weeks	12.8 (12.6) [133]	11.7 (12.6) [134]	-0.62 [-2.74 to 1.49]	0.565

	VR+TAU (N=174)	TAU (N=172)	Adjusted treatment difference [95% CI][1]	P value
**R-GPTS (overall) score, mean (SD) [n]**				
6 weeks	23.6 (19.3) [142]	22.9 (19.9) [146]	-3.14 [-6.49 to 0.21]	0.066
26 weeks	23.8 (21.3) [133]	22.1 (20.4) [134]	-1.10 [-4.52 to 2.33]	0.530
**Paranoia Worries Questionnaire (PWQ) total score, mean (SD) [n]**				
6 weeks	7.7 (6.1) [141]	7.5 (6.1) [145]	-0.47 [-1.60 to 0.66]	0.416
26 weeks	7.3 (6.1) [127]	7.1 (6.5) [134]	-0.15 [-1.32 to 1.01]	0.794
**Patient Health Questionnaire (PHQ-9) total score, mean (SD) [n]**				
6 weeks	12.5 (6.2) [147]	12.1 (6.0) [150]	-0.24 [-1.48 to 0.99]	0.700
26 weeks	12.5 (6.7) [134]	11.6 (6.6) [137]	0.11 [-1.17 to 1.39]	0.866
**Columbia Suicide Severity Rating Scale (C-SSRS) Suicidal Ideation total score, mean (SD) [n]**				
6 weeks	0.9 (1.3) [121]	0.8 (1.4) [123]	-0.14 [-0.33 to 0.04]	0.129
26 weeks	0.8 (1.3) [110]	0.7 (1.3) [113]	-0.06 [-0.25 to 0.14]	0.571
**EQ-5D-5L INDEX, mean (SD) [n]**				
6 weeks	0.6 (0.3) [152]	0.6 (0.3) [155]	0.03 [-0.02 to 0.08]	0.231
26 weeks	0.6 (0.3) [142]	0.6 (0.3) [145]	-0.00 [-0.05 to 0.05]	0.863
**EQ5D VAS score[2], mean (SD) [n]**				
6 weeks	56.6 (19.7) [153]	54.9 (20.7) [156]	3.06 [-1.18 to 7.30]	0.157
26 weeks	56.1 (21.3) [145]	56.7 (22.4) [146]	-0.29 [-4.65 to 4.06]	0.895
**Recovering Quality Of Life (ReQOL-20) total score[2], mean (SD) [n]**				
6 weeks	38.1 (13.8) [149]	39.4 (14.5) [147]	1.06 [-1.53 to 3.65]	0.422
26 weeks	39.5 (15.1) [136]	40.8 (15.2) [137]	0.57 [-2.10 to 3.25]	0.673
**Progress of Recovery (QPR) total score[2], mean (SD) [n]**				
6 weeks	32.4 (11.2) [159]	31.0 (11.3) [159]	2.83 [0.90 to 4.75]	0.004
26 weeks	33.1 (11.7) [148]	32.6 (12.1) [151]	1.71 [-0.25 to 3.67]	0.088

[1] VR+TAU versus TAU only: Mean difference estimated from a linear mixed-effects model adjusting for site, service type and baseline values of the outcome as fixed effects and participant as the random effect for all outcomes. Standardised effect size = estimated mean difference divided by baseline standard deviation.
[2] Positively framed secondary outcomes: positive mean differences indicate that, on average, the VR+TAU group is doing better than TAU alone. The remaining outcomes are negatively framed so that positive mean differences indicate that, on average, the VR+TAU group is doing worse than TAU alone.
[3] Baseline number of steps avoided modelled as a categorical rather than continuous measure.
Level of significance = 0.05

The overall O-AS distress score can also be interpreted as follows:

≤23 = Average distress
24-46 = Moderate distress
46-66 = High distress
66+ = Severe distress

3.6 Mediation analysis

The mediation analyses were initially carried out using structural equation modelling (SEM); however, none of the models converged and thus did not produce reliable estimates. As a result, the analyses were run using the approach of Baron and Kenny (1986), but follow the adaptation in Freeman et al. (2017) which makes use of linear mixed effects models, as specified in the SAP.

A representation of the mediation analysis following the Baron and Kenny (1986) approach of the tested pathways is shown below.

The amount of mediation is called the indirect effect.

Total effect (c) = Direct effect (c') + Indirect effect (ab)

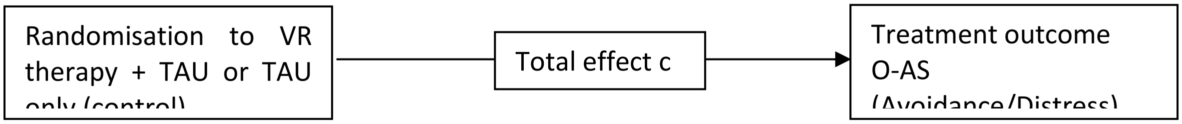

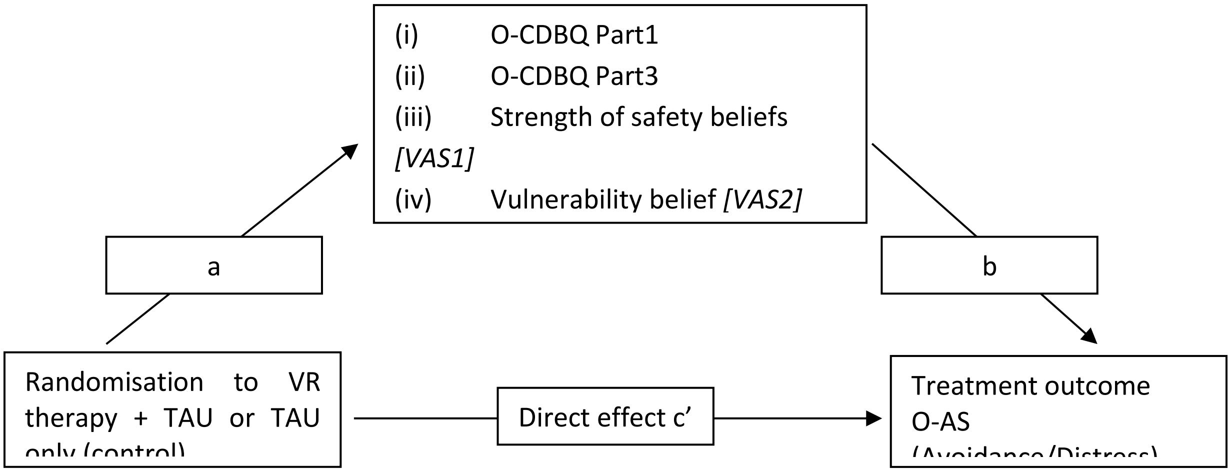

Table 6 & Table 7 show the mediation results for the O-AS Avoidance and Distress outcomes respectively. For each of the mediators, the tables shows the total, direct and indirect effects of treatment group on the outcome.

The indirect effects represent the estimated mediated effects on O-AS Avoidance.

3.6.1 O-AS Avoidance Score

The results show that for the O-CDBQ Part 1(worry) & O-CDBQ Part3 (putting up defences), where higher scores indicate worse outcomes, there no evidence of a direct effect at the either time point but there is some evidence of an indirect/mediated effect at the 6-week time point. As the estimated treatment effects for both scales are negative, the VR+TAU group is doing better than the TAU only group. For the three VAS scores, there is some direct effect but again no indirect effect and thus no evidence of a mediated effect of treatment on the outcome.

There is no statistically significant indirect effect of any of the mediators at 26-weeks.

TABLE 6: MEDIATION ANALYSIS ON O-AS AVOIDANCE USING MIXED-EFFECTS MODELS

Mediator	Effect estimate	6-weeks: Estimate (95% CI); P value	26-weeks: Estimate (95% CI); P value
**O-CDBQ Part1**	**Total effect**	-0.53 (-0.95 to -0.12); P = 0.011	-0.43 (-0.85 to -0.02); P = 0.042
	**Direct effect**	-0.38 (-0.79 to 0.03); P = 0.066	-0.30 (-0.72 to 0.12); P = 0.158
	**Indirect effect**	-0.14 (-0.27 to -0.02); P = 0.022	-0.11 (-0.23 to 0.02); P = 0.089
	**Proportion mediated[1]**	0.27	0.25
**O-CDBQ Part3**	**Total effect**	-0.50 (-0.91 to -0.08); P = 0.019	-0.40 (-0.81 to 0.02); P = 0.063
	**Direct effect**	-0.35 (-0.76 to 0.05); P = 0.087	-0.23 (-0.65 to 0.18); P = 0.268
	**Indirect effect**	-0.15 (-0.29 to -0.02); P = 0.027	-0.14 (-0.28 to 0.00); P = 0.054
	**Proportion mediated[1]**	0.31	0.35
**VAS1 - 'I generally feel safe around other people.'**	**Total effect**	-0.57 (-0.99 to -0.15); P = 0.008	-0.50 (-0.92 to -0.08); P = 0.021

Mediator	Effect estimate	6-weeks: Estimate (95% CI); P value	26-weeks: Estimate (95% CI); P value
	**Direct effect**	-0.50 (-0.92 to -0.08); P = 0.019	-0.45 (-0.89 to -0.02); P = 0.040
	**Indirect effect**	-0.04 (-0.12 to 0.04); P = 0.316	-0.06 (-0.15 to 0.02); P = 0.151
	**Proportion mediated[1]**	0.07	0.13
**VAS2 - 'I feel vulnerable.'**	**Total effect**	-0.57 (-1.00 to -0.15); P = 0.007	-0.50 (-0.93 to -0.08); P = 0.019
	**Direct effect**	-0.50 (-0.93 to -0.07); P = 0.022	-0.49 (-0.93 to -0.05); P = 0.029
	**Indirect effect**	-0.05 (-0.12 to 0.01); P = 0.114	-0.03 (-0.09 to 0.03); P = 0.320
	**Proportion mediated[1]**	0.09	0.06
**VAS3 - 'When I go out, something bad will happen.'**	**Total effect**	-0.58 (-1.00 to -0.16); P = 0.007	-0.51 (-0.93 to -0.09); P = 0.017
	**Direct effect**	-0.46 (-0.87 to -0.05); P = 0.030	-0.47 (-0.89 to -0.04); P = 0.031
	**Indirect effect**	-0.09 (-0.20 to 0.02); P = 0.106	-0.06 (-0.17 to 0.05); P = 0.310
	**Proportion mediated[1]**	0.16	0.11

Indirect/total effect

3.6.2 O-AS Distress Score

For the O-CDBQ Part 1(worry) & O-CDBQ Part3 (putting up defences), where higher scores indicate worse outcomes, there no evidence of a direct effect at the either time point but there is some evidence of an indirect/mediated effect at the 6-week time point for both scales and at the 26-week time point for the O-CDBQ3. The estimated treatment effects for the scales are negative, indicating that the VR+TAU group is doing better than the TAU only group. There are direct effects of treatment on the distress outcome for all three VAS scores at 6-weeks but no indirect effects at either time point. VAS1 is positively framed so higher scores indicate higher feelings of safety, thus; negative treatment differences imply that on average participants in the VR+TAU group feel less safe. VAS2 and 3 are negatively framed, thus negative treatment differences imply that on average participants in the VR+TAU group are doing better.

TABLE 7: MEDIATION ANALYSIS ON O-AS DISTRESS USING MIXED-EFFECTS MODELS

Mediator	Effect estimate	6-weeks:Estimate (95% CI); P value	26-weeks:Estimate (95% CI); P value
**O-CDBQ Part1**	**Total effect**	-4.58 (-8.06 to -1.09); P = 0.010	-2.89 (-6.40 to 0.61); P = 0.106
	**Direct effect**	-2.54 (-5.86 to 0.79); P = 0.135	-1.36 (-4.78 to 2.05); P = 0.434
	**Indirect effect**	-1.62 (-2.96 to -0.27); P = 0.018	-1.21 (-2.58 to 0.16); P = 0.084
	**Proportion mediated[1]**	0.35	0.42
**O-CDBQ Part3**	**Total effect**	-4.38 (-7.87 to -0.89); P = 0.014	-2.60 (-6.11 to 0.91); P = 0.146
	**Direct effect**	-2.17 (-5.42 to 1.07); P = 0.190	-0.61 (-3.94 to 2.72); P = 0.720
	**Indirect effect**	-1.81 (-3.38 to -0.24); P = 0.024	-1.60 (-3.21 to 0.00); P = 0.050
	**Proportion mediated[1]**	0.41	0.62

Mediator	Effect estimate	6-weeks:Estimate (95% CI); P value	26-weeks:Estimate (95% CI); P value
**VAS1 - 'I generally feel safe around other people.'**	**Total effect**	-4.89 (-8.49 to -1.29); P = 0.008	-3.14 (-6.75 to 0.47); P = 0.088
	**Direct effect**	-4.23 (-7.80 to -0.67); P = 0.020	-2.23 (-5.90 to 1.45); P = 0.235
	**Indirect effect**	-0.45 (-1.31 to 0.42); P = 0.311	-0.68 (-1.60 to 0.23); P = 0.143
	**Proportion mediated[1]**	0.09	0.22
**VAS2 - 'I feel vulnerable.'**	**Total effect**	-5.00 (-8.60 to -1.39); P = 0.007	-3.24 (-6.85 to 0.37); P = 0.079
	**Direct effect**	-4.01 (-7.62 to -0.39); P = 0.030	-2.52 (-6.23 to 1.18); P = 0.181
	**Indirect effect**	-0.82 (-1.74 to 0.10); P = 0.081	-0.49 (-1.41 to 0.44); P = 0.302
	**Proportion mediated[1]**	0.16	0.15
**VAS3 - 'When I go out, something bad will happen.'**	**Total effect**	-5.06 (-8.65 to -1.47); P = 0.006	-3.32 (-6.92 to 0.28); P = 0.071
	**Direct effect**	-3.74 (-7.19 to -0.28); P = 0.034	-2.38 (-5.94 to 1.17); P = 0.189
	**Indirect effect**	-1.01 (-2.22 to 0.20); P = 0.101	-0.64 (-1.86 to 0.59); P = 0.307
	**Proportion mediated[1]**	0.20	0.19

1 Indirect/total effect

3.7 Moderation/Subgroup analyses

Subgroup analyses were carried to assess which variables (pre-specified in the protocol) had an impact on the primary outcomes. This trial was not designed to detect subgroup effects and thus lacks statistical power. All subgroup analyses should be considered exploratory in nature. The distribution of treatment deliverer across sites is summarised in

Appendix *VI. Treatment* deliverer. It was not included in the analysis below as all the treatment deliverers were in one treatment group. Regression diagnostic plots for the models reported in Table 8 are available but not presented in this document.

The effect sizes in Table 8 are the estimated interaction effects between treatment group and the moderator and the p-value indicates whether this interaction is statistically significant.

The results show that for the primary outcomes of Avoidance as measured by the O-AS, there is a statistically significant moderator effect for the O-CDBQ Part 1 (worries) questionnaire. As this is a negatively framed scale, negative effects imply that the VR+TAU group is performing better than TAU alone with each unit-wise change in the moderator. For the O-CDBQ1 scale, this implies that with each unit increase in the worry scale, mean avoidance in the VR+TAU group decreases by 0.06 units more than for TAU alone. In other words, as worry increases, so too does the difference in avoidance between the treatment groups.

TABLE 8: SUMMARY STATISTICS AND RESULTS FOR THE SUBGROUP ANALYSIS ON O-AS AVOIDANCE & DISTRESS

	VR+TAU (N=174)	TAU (N=172)	Interaction effect [95% CI][1]	Test of Interaction (P value)‡
**O-AS Avoidance at 6 weeks**				
**Voices questionnaire[2], n**	160	160	0.01 [-0.05 to 0.07]	0.676
**Beck Hopelessness Scale [BHS][2], n**	160	160	0.05 [-0.02 to 0.13]	0.165
**Body-esteem Scale for Adolescents and Adults, n**	160	160	0.01 [-0.01 to 0.04]	0.361
**O-CDBQ Part 1[2], n**	160	160	-0.06 [-0.11 to -0.01]	0.012
**O-AS Distress at 6 weeks**				
**Voices questionnaire[2], n**	160	162	0.08 [-0.40 to 0.56]	0.737
**Beck Hopelessness Scale [BHS][2], n**	160	162	0.17 [-0.44 to 0.79]	0.575
**Body-esteem Scale for Adolescents and Adults, n**	160	162	0.10 [-0.12 to 0.32]	0.350
**O-CDBQ Part 1[2], n**	160	162	-0.37 [-0.74 to 0.01]	0.054

[1] VR+TAU vs. TAU: Linear regression model for the primary outcome; modelled against treatment group, outcome score at baseline, stratification factors (site and service type) and an interaction between randomised group and the subgroup variable.

[2] Negatively framed questions: higher scores indicate worse outcomes
‡ Level of significance = 0.05

3.8 Sensitivity analyses

3.8.1 O-AS Avoidance Score

3.8.1.1 Outliers and missingness assumptions

No outliers were found.

The frequency and percentage of each missing data pattern for the O-AS Avoidance score by randomised group is presented in Table 9. A pattern mixture model was fitted to assess the robustness of the Missing At Random (MAR) assumption required for the linear mixed effects model. Results are shown in Figure 3 and indicate that even across a range of missing data patterns treatment difference would still be significant, indicating the robustness of the main results even with strong departure from MAR assumption.

TABLE 9: MISSINGNESS PATTERN FOR O-AS AVOIDANCE AT THE FOLLOW-UP TIME POINTS, BY RANDOMISED GROUP

Missingness pattern for O-AS Avoidance	Follow-up time point (P = Observed)		Randomised group	
	6 weeks	26 weeks	VR+TAU (N=174)	TAU (N=172)
Missing at all follow-up time points			6 (3.4)	8 (4.7)
Available at 6 weeks only	✓		11 (6.3)	5 (2.9)
Available at 26 weeks only		✓	8 (4.6)	4 (2.3)
All follow-up data is available	✓	✓	149 (85.6)	155 (90.1)

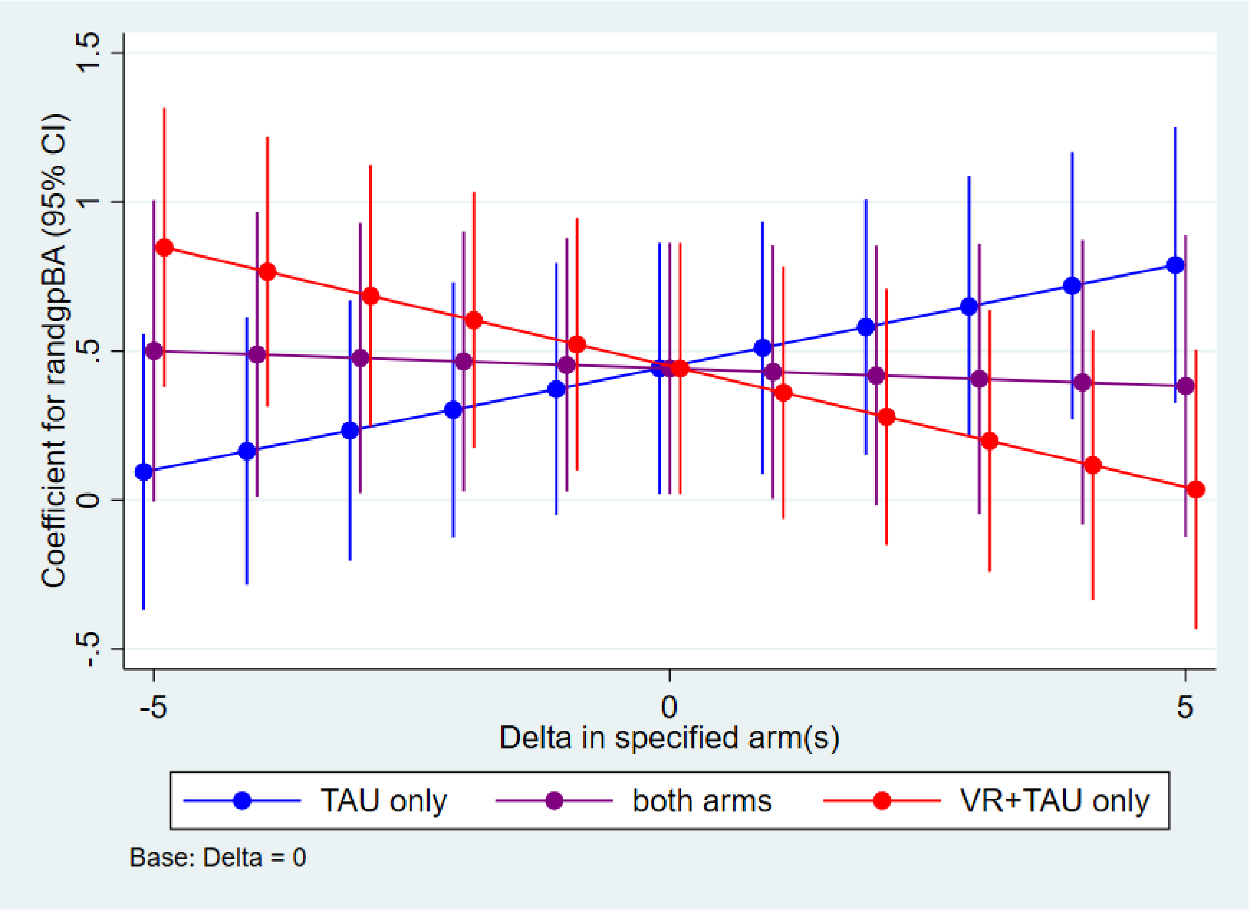

FIGURE 3 PATTERN MIXTURE MODEL RESULTS FOR O-AS AVOIDANCE AT 6 WEEKS

3.8.1.1.1 Primary analysis assuming plausible arm specific differences (assuming data MNAR for O-AS Avoidance)

Using the approach by White et al 2011 to carry out sensitivity analyses to investigate informative missing of O-AS Avoidance at 6 weeks, the following assumptions of differences between responders and non-responder were carried out:

- When the proportion of missing O-AS Avoidance at 6 weeks are assumed to be the same in both arms (i.e. Both arms equally), assumes the mean of unobserved responses for O-AS Avoidance at 6 weeks could be as much as 75% more or 50% less (i.e. -50%) than the mean of observed responses
- When the data is assumed to be informatively missing only in TAU, assumes the mean of unobserved responses for O-AS Avoidance at 6 weeks could be as much as 50% more or 50% less (i.e. -50%) than the mean of observed responses

- When the data is assumed to be informatively missing only in VR+TAU assumes the mean of unobserved responses for O-AS Avoidance at 6 weeks could be as much as 50% more or 50% less (i.e. -50%) than the mean of observed responses
- Additionally, more moderate sensitivity analyses includes:
    - Data is informatively missing in both arms, assumes 50%*
    - Data is informatively missing in TAU assumed as much as 25% more*
    - Data is informatively missing in VR+TAU assumed as much as 25% more*

Table 10 shows results when we assume plausible arm specific differences of missing O-AS Avoidance score at 6 months between responders and non-responders. The results indicate that even with asymmetrical differences between responders and non-responders conclusions remain similar to the primary analysis.

TABLE 10: SENSITIVITY ANALYSIS USING MNAR ASSUMPTION FOR THE TREATMENT EFFECT ON O-AS AVOIDANCE AT 6 WEEKS

Non-responders differ in	Assumed difference between non-responders and responders	Adjusted mean difference [95% CI]†	P-value‡
**Both arms equally**	-50	-0.45 [-0.87 to -0.03]	0.038
	50*	-0.44 [-0.86 to -0.01]	0.043
	75	-0.43 [-0.86 to -0.01]	0.044
**Only in VR+TAU**	-50	-0.50 [-0.92 to -0.08]	0.021
	25*	-0.42 [-0.85 to -0.00]	0.049
	50	-0.41 [-0.83 to 0.01]	0.058
**Only in TAU**	-50	-0.39 [-0.82 to 0.03]	0.068
	25*	-0.46 [-0.88 to -0.04]	0.034
	50	-0.47 [-0.89 to -0.05]	0.029

†VR+TAU versus TAU: Models adjusted for baseline O-AS Avoidance score. ‡Level of significance = 0.05

3.8.1.2 Other sensitivity analyses

The results below show that there is still a significant treatment difference (VR+TAU performing even better than prior to adjustment – treatment difference increases from -0.47 to -0.51) after adjusting for baseline Time Budget measurements; that there is no evidence of a treatment difference when considering only participants who completed their 6-week measures before the beginning of lockdown; nor when considering only those who completed their 26-week follow-up before lockdown. There is also no evidence of a difference when omitting those with shortened follow-up nor are there any medication prescription effects on differences between the treatment groups. There is also no evidence of treatment difference if outliers are omitted.

TABLE 11: SUMMARY STATISTICS FOR THE SENSITIVITY ANALYSES AND THE TREATMENT DIFFERENCE BETWEEN THE RANDOMISED GROUPS

O-AS Avoidance	VR+TAU (N=174) mean (SD) [n]	TAU (N=172) mean (SD) [n]	Adjusted mean difference [95% CI][1]	P value
**Adjusting for baseline characteristics associated with non-response[2]**				
6 weeks	1.9 (2.2) [160]	2.5 (2.6) [160]	-0.51 [-0.95 to -0.07]	0.022
26 weeks	2.0 (2.3) [157]	2.5 (2.6) [159]	-0.43 [-0.87 to 0.01]	0.054
**Impact of the COVID-19 pandemic - 6-week measures completed before the beginning of lockdown[3]**				
6 weeks	1.9 (2.2) [65]	2.8 (2.6) [60]	-0.56 [-1.19 to 0.08]	0.085
26 weeks	1.4 (1.8) [13]	3.3 (3.1) [12]	-0.92 [-1.81 to -0.03]	0.042
**Impact of the COVID-19 pandemic - 26-week measures completed before the beginning of lockdown**				
6 weeks	1.4 (2.1) [13]	2.9 (3.1) [12]	-0.33 [-1.17 to 0.51]	0.442
26 weeks	1.4 (1.8) [13]	3.3 (3.1) [12]	-0.66 [-1.51 to 0.18]	0.123
**Shortened follow-up - exclude those randomised from 1 March 2021**				
6 weeks	2.0 (2.2) [129]	2.6 (2.6) [124]	-0.45 [-0.91 to 0.00]	0.052
26 weeks	2.2 (2.4) [126]	2.6 (2.6) [124]	-0.34 [-0.80 to 0.12]	0.149
**Medication effects - Prescription of antipsychotic medication expressed as a chlorpromazine equivalent score†**				
6 weeks	1.9 (2.2) [160]	2.5 (2.6) [160]	-0.60 [-1.28 to 0.07]	0.080
26 weeks	2.0 (2.3) [157]	2.5 (2.6) [159]	-0.35 [-1.05 to 0.34]	0.318
**Outlier analysis - removing observations more than three standard deviations from the mean**				

O-AS Avoidance	VR+TAU (N=174) mean (SD) [n]	TAU (N=172) mean (SD) [n]	Adjusted mean difference [95% CI][1]	P value
6 weeks	1.6 (1.8) [148]	1.9 (2.1) [138]	-0.28 [-0.66 to 0.11]	0.158
26 weeks	1.6 (1.8) [145]	1.8 (2.0) [137]	-0.29 [-0.67 to 0.09]	0.140

[1] VR+TAU versus TAU: estimated from a linear mixed-effects model adjusting for site, service type and baseline values of the outcome as fixed effects and participant as the random effect
[2] Models further adjust for baseline time budget scores
[3] Analysis on all participants who completed their 6-week measures before the beginning of lockdown on the 23rd March 2020 but setting any follow-up measures that were completed after lockdown as missing.
† VR+TAU versus TAU: estimated from a linear mixed-effects model adjusting for site, service type and baseline values of the outcome as fixed effects and participant as the random effect. Model includes a three-way interaction between randomised group, CPZ score and time
Level of significance = 0.05

Regression diagnostic plots are not presented here but are available if of interest.

Please refer to ***Appendix VIX.*** *Baseline tables for sensitivity analyses* for tables of baseline characteristics for participants recruited pre-COVID-19 lockdown and during the lockdown5.9, respectively.

3.8.1.3 Complier Average Causal Effect (CACE) analysis

Table 12 shows the CACE estimate for O-AS at 6 weeks from a simple linear regression model. Table 13 gives a summary of compliance and calculation of CACE is shown below the table. The effect of receiving VR therapy + TAU shows a significant decrease in avoidance compared to TAU only for both the unadjusted and adjusted models.

TABLE 12: ESTIMATES OF COMPLIER AVERAGE CAUSAL EFFECTS

	Unadjusted mean difference (95% CI); P value	Adjusted mean difference (95% CI); P value†
**Linear regression model**		
**VR+TAU vs. TAU**	-0.60 (-1.13 to -0.06), 0.029	-0.43 (-0.85 to -0.01), 0.047
**Instrumental variable regression model**		
**VR+TAU vs. TAU**	-0.7 (-1.4 to -0.1), 0.028	-0.5 (-1.0 to -0.0), 0.043

† Models adjusted for baseline values of the outcome. Stata command: “ivregress 2sls outcome baselineOut (compliance=randgp)”

TABLE 13 SUMMARY OF COMPLIANCE FOR O-AS AVOIDANCE SCORE AT 6 WEEKS

	Completion of at 3 VR therapy sessions		
**Randomised group**	**No**	**Yes**	**Total**
**VR+TAU**	28 (17.5 %) **(Never-takers)**	132 (82.5 %) **(Compliers)**	160
**TAU**	160 (100 %) **(Compliers)**	0 **(Always takers)**	160
**Total**	188	132	320

$\textbf{Compliance rate} = \big(1 - (0.175)\big) = 0.825$

$\boldsymbol{Estimate} = -0.43 \qquad \textbf{CACE} = \frac{\boldsymbol{Estimate}}{\boldsymbol{Compliance\ rate}}, \qquad \textbf{CACE} = \frac{-0.43}{\mathbf{0.825}} = -0.52$

Please see

Appendix VII. Baseline tables for CACE analysis population for summary tables of baseline characteristics for the participants included in the CACE analysis stratified by randomised group.

3.8.2 O-AS Distress Score

3.8.2.1 Outliers and missingness assumptions

No outliers were found.

The frequency and percentage of each missing data pattern for the O-AS Distress score by randomised group is presented in Table 14. A pattern mixture model was fitted to assess the robustness of the Missing At Random (MAR) assumption required for the linear mixed effects model. Results are shown in Figure 4 and indicate that even across a range of missing data patterns treatment difference would still be significant, indicating the robustness of the main results even with strong departure from MAR assumption.

TABLE 14: MISSINGNESS PATTERN FOR O-AS DISTRESS AT THE FOLLOW-UP TIME POINTS, BY RANDOMISED GROUP

Missingness pattern for O-AS Distress	Follow-up time point (P = Observed)		Randomised group	
	6 weeks	26 weeks	VR+TAU (N=174)	TAU (N=172)
Missing at all follow-up time points			7 (4.0)	6 (3.5)
Available at 6 weeks only	✓		11 (6.3)	5 (2.9)
Available at 26 weeks only		✓	7 (4.0)	4 (2.3)
All follow-up data is available	✓	✓	149 (85.6)	157 (91.3)

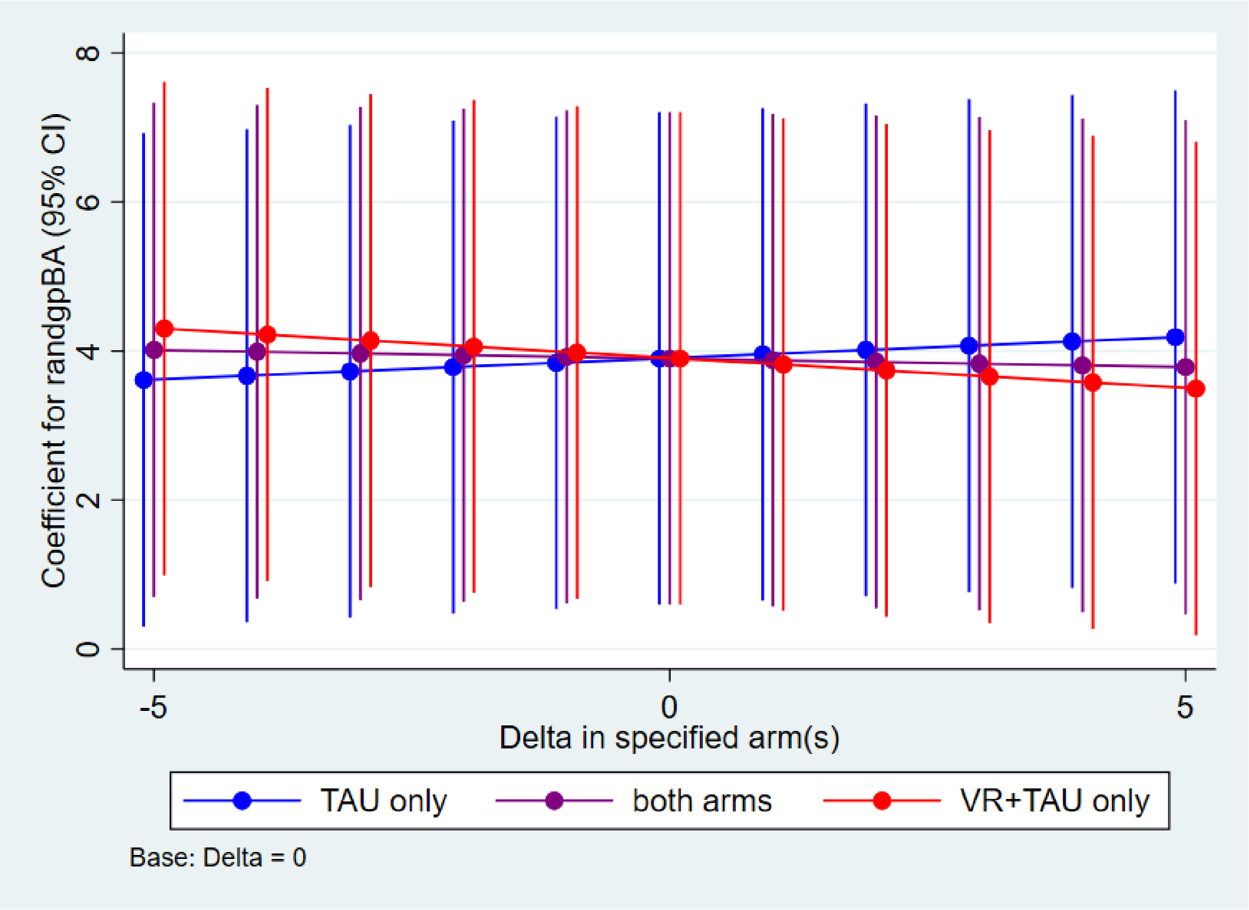

FIGURE 4 PATTERN MIXTURE MODEL RESULTS FOR O-AS DISTRESS AT 6 WEEKS

3.8.2.1.1 Primary analysis assuming plausible arm specific differences (assuming data MNAR for O-AS Distress at 6 weeks)

Using the approach by White et al 2011 to carry out sensitivity analyses to investigate informative missing of O-AS Avoidance at 6 weeks, the following assumptions of differences between responders and non-responder were carried out:

- When the proportion of missing O-AS Avoidance at 6 weeks are assumed to be the same in both arms (i.e. Both arms equally), assumes the mean of unobserved responses for O-AS Avoidance at 6 weeks could be as much as 75% more or 50% less (i.e. -50%) than the mean of observed responses
- When the data is assumed to be informatively missing only in TAU, assumes the mean of unobserved responses for O-AS Avoidance at 6 weeks could be as much as 50% more or 50% less (i.e. -50%) than the mean of observed responses

- When the data is assumed to be informatively missing only in VR+TAU assumes the mean of unobserved responses for O-AS Avoidance at 6 weeks could be as much as 50% more or 50% less (i.e. -50%) than the mean of observed responses
- Additionally, more moderate sensitivity analyses includes:
    - Data is informatively missing in both arms, assumes 50%*
    - Data is informatively missing in TAU assumed as much as 25% more*
    - Data is informatively missing in VR+TAU assumed as much as 25% more*

Table 15 shows results when we assume plausible arm specific differences of missing O-AS Distress score at 6 months between responders and non-responders. The results indicate that even with asymmetrical differences between responders and non-responders conclusions remain similar to the primary analysis.

TABLE 15: SENSITIVITY ANALYSIS USING MNAR ASSUMPTION FOR THE TREATMENT EFFECT ON O-AS DISTRESS AT 6 WEEKS

Non-responders differ in	Assumed difference between non-responders and responders	Adjusted mean difference [95% CI]†	P-value‡
**Both arms equally**	-50	-3.91 [-7.22 to -0.61]	0.020
	50*	-3.89 [-7.20 to -0.58]	0.021
	75	-3.89 [-7.19 to -0.58]	0.021
**Only in VR+TAU**	-50	-3.95 [-7.26 to -0.65]	0.019
	25*	-3.88 [-7.19 to -0.57]	0.022
	50	-3.87 [-7.17 to -0.56]	0.022
**Only in TAU**	-50	-3.86 [-7.16 to -0.55]	0.022
	25*	-3.91 [-7.22 to -0.61]	0.021
	50	-3.92 [-7.23 to -0.62]	0.020

†VR+TAU versus TAU: Models adjusted for baseline O-AS Avoidance score. ‡Level of significance = 0.05

3.8.2.2 *Other sensitivity analyses*

The results below show that there is still a significant treatment difference after adjusting for baseline Time Budget measurements (VR+TAU group still performing better but now with a larger decrease in distress than prior to adjustment for time budget – treatment difference increases from -4.33 to -5.14 at 6-weeks); that there is some evidence of a treatment difference when considering only participants who completed their 6-week measures before the beginning of lockdown (treatment difference increases from -4.33 to -6.12 in favour of VR+TAU) and when omitting those with shortened follow-up (treatment effect remains the same i.e. in favour of VR+TAU, but the CI is slightly larger). There is no evidence of a difference when considering only those who completed their 26-week follow-up before lockdown, nor are there any medication prescription effects on differences between the treatment groups

TABLE 16: SUMMARY STATISTICS FOR THE SENSITIVITY ANALYSES AND THE TREATMENT DIFFERENCE BETWEEN THE RANDOMISED GROUPS

O-AS Distress	VR+TAU (N=174) mean (SD) [n]	TAU (N=172) mean (SD) [n]	Adjusted mean difference [95% CI][1]	P value
**Adjusting for characteristics associated with non-response[2]**				
6 weeks	41.3 (18.8) [160]	45.8 (20.4) [162]	-5.14 [-8.83 to -1.46]	0.006
26 weeks	40.7 (20.6) [156]	43.9 (21.6) [161]	-3.55 [-7.25 to 0.15]	0.060
**Impact of the COVID-19 pandemic - 6-week measures completed before the beginning of lockdown[3]**				
6 weeks	40.9 (18.1) [65]	46.6 (20.1) [60]	-6.12 [-11.65 to -0.59]	0.030
26 weeks	34.9 (18.6) [13]	44.8 (29.0) [12]	-6.53 [-14.43 to 1.38]	0.106
**Impact of the COVID-19 pandemic - 26-week measures completed before the beginning of lockdown**				
6 weeks	37.7 (16.5) [13]	46.4 (26.3) [12]	4.50 [-9.83 to 18.83]	0.538
26 weeks	34.9 (18.6) [13]	44.8 (29.0) [12]	3.32 [-11.01 to 17.65]	0.650
**Shortened follow-up - exclude those randomised from 1 March 2021**				
6 weeks	42.2 (18.1) [129]	46.7 (20.2) [126]	-4.33 [-8.28 to -0.38]	0.032
26 weeks	41.4 (20.1) [126]	45.0 (22.3) [126]	-3.16 [-7.12 to 0.81]	0.119
**Medication effects - Prescription of antipsychotic medication expressed as a chlorpromazine equivalent score†**				
6 weeks	41.3 (18.8) [160]	45.8 (20.4) [162]	-4.89 [-10.44 to 0.66]	0.084

O-AS Distress	VR+TAU (N=174) mean (SD) [n]	TAU (N=172) mean (SD) [n]	Adjusted mean difference [95% CI][1]	P value
26 weeks	40.7 (20.6) [156]	43.9 (21.6) [161]	-0.23 [-5.91 to 5.44]	0.936

[1] VR+TAU versus TAU: estimated from a linear mixed-effects model adjusting for site, service type and baseline values of the outcome as fixed effects and participant as the random effect
[2] Models further adjust for baseline time budget scores
[3] Analysis on all participants who completed their 6-week measures before the beginning of lockdown on the 23rd March 2020 but setting any follow-up measures that were completed after lockdown as missing.
† VR+TAU versus TAU: estimated from a linear mixed-effects model adjusting for site, service type and baseline values of the outcome as fixed effects and participant as the random effect. Model includes a three-way interaction between randomised group, CPZ score and time
Level of significance = 0.05

Regression diagnostic plots are not presented here but are available.

Please refer to ***Appendix VIX.*** *Baseline tables for sensitivity analyses* for tables of baseline characteristics for participants recruited pre-COVID-19 lockdown and during the lockdown5.9, respectively

3.8.2.3 Complier Average Causal Effect (CACE) analysis

Table 17 shows the CACE estimate for O-AS at 6 weeks from a simple linear regression model. Table 18 gives a summary of compliance and calculation of CACE is shown below the table. The effect of receiving VR therapy + TAU shows a significant reduction in distress compared to TAU alone for the unadjusted and adjusted models .

TABLE 17: ESTIMATES OF COMPLIER AVERAGE CAUSAL EFFECTS

	**Unadjusted mean difference (95% CI); P value**	**Adjusted mean difference (95% CI); P value†**
**Linear regression model**		
VR+TAU vs. TAU	-4.57 (-8.87 to -0.26), 0.038	-3.90 (-7.20 to -0.59), 0.021
**Instrumental variable regression model**		
VR+TAU vs. TAU	-5.5 (-10.7 to -0.4), 0.036	-4.7 (-8.7 to -0.8), 0.019

† Models adjusted for baseline values of the outcome. Stata command: "ivregress 2sls outcome baselineOut (compliance=randgp)"

TABLE 18 SUMMARY OF COMPLIANCE FOR O-AS DISTRESS SCORE AT 6 WEEKS

	**Completion of at 3 VR therapy sessions**		
**Randomised group**	**No**	**Yes**	**Total**
**VR+TAU**	28 (17.5 %)<br>**(Never-takers)**	132 (82.5 %)<br>**(Compliers)**	160
**TAU**	162 (100 %)<br>**(Compliers)**	0<br>**(Always takers)**	162
**Total**	190	132	322

$$\textbf{Compliance rate} = \left(1 - (0.175)\right) = 0.825$$

$$\boldsymbol{Estimate} = -3.90 \qquad \textbf{CACE} = \frac{\boldsymbol{Estimate}}{\boldsymbol{Compliance\ rate}}, \qquad \textbf{CACE} = \frac{\mathbf{-3.90}}{\mathbf{0.825}} = -4.73$$

Please see

Appendix VII. Baseline tables for CACE analysis population for summary tables of baseline characteristics for the participants included in the CACE analysis stratified by randomised group.

3.9 Safety analyses

Frequencies of adverse and serious adverse events for each treatment group are reported below.

All randomised participants were included in the safety analysis. Fisher's exact test was used to compare the proportion of events as well as the proportion of participants experiencing at least one event between the randomised groups. There are no significant differences in safety events between the treatment groups

35 participants had more than one AE/SAE.

TABLE 19: SUMMARY OF SAFETY EVENTS BY RANDOMISED GROUP

	VR+TAU	TAU	
**Number of Events**	**(N=183)**	**(N=185)**	**P Value[1]**
**AEs, n (%)**	25 (13.7)	29 (15.7)	0.659
**SAEs, n (%)**	12 (6.6)	8 (4.3)	0.368
**Number of participants experiencing at least one event**	**(N=174)**	**(N=172)**	**P Value[1]**
**AE, n (%)**	21 (12.1)	19 (11.0)	0.867
**SAE, n (%)**	9 (5.2)	7 (4.1)	0.799

[1] From Fisher's exact test

Table 20 gives a summary of SAEs per participant while the MedDRA coded SAE list is in Table 21.

TABLE 20: SUMMARY OF SAES PER PARTICIPANT

This table contains sensitive participant information and was remove for publication purposes.

TABLE 21: MEDDRA CODED SAE FREQUENCY TABLE

System Organ Class	Preferred Term	VR+TAU participants n=9 / Number of events 12	TAU only participants n=7 / Number of events 8	Total participants N=16 / Number of events 20
Gastrointestinal disorders	Abdominal pain upper	1 (6.3%) / 1	0 (0.0%) / 0	1 (6.3%) / 1
Hepatobiliary disorders	Cholelithiasis	1 (6.3%) / 1	0 (0.0%) / 0	1 (6.3%) / 1
Infections and infestations	Infection	0 (0.0%) / 0	1 (6.3%) / 1	1 (6.3%) / 1
Infections and infestations	Localised infection	1 (6.3%) / 1	0 (0.0%) / 0	1 (6.3%) / 1
Injury, poisoning and procedural complications	Multiple fractures	0 (0.0%) / 0	1 (6.3%) / 1	1 (6.3%) / 1
Injury, poisoning and procedural complications	Overdose	1 (6.3%) / 1	0 (0.0%) / 0	1 (6.3%) / 1
Nervous system disorders	Mental impairment	2 (12.5%) / 2	2 (12.5%) / 2	4 (25.0%) / 4
Psychiatric disorders	Acute psychosis	1 (6.3%) / 1	0 (0.0%) / 0	1 (6.3%) / 1
Psychiatric disorders	Intentional self-injury	2 (12.5%) / 2	0 (0.0%) / 0	2 (12.5%) / 2
Psychiatric disorders	Psychotic disorder	1 (6.3%) / 1	1 (6.3%) / 1	2 (12.5%) / 2
Psychiatric disorders	Suicidal ideation	0 (0.0%) / 0	1 (6.3%) / 1	1 (6.3%) / 1
Psychiatric disorders	Suicide attempt	0 (0.0%) / 0	1 (6.3%) / 1	1 (6.3%) / 1
Psychiatric disorders	Thinking abnormal	1 (6.3%) / 1	0 (0.0%) / 0	1 (6.3%) / 1

System Organ Class	Preferred Term	VR+TAU participants n=9 / Number of events 12	TAU only participants n=7 / Number of events 8	Total participants N=16 / Number of events 20
Surgical and medical procedures	Infusion	1 (6.3%) / 1	0 (0.0%) / 0	1 (6.3%) / 1
Vascular disorders	Circulatory collapse	0 (0.0%) / 0	1 (6.3%) / 1	1 (6.3%) / 1

Note: the percentages are all out of the total number of participants (n=16)

3.10 Additional exploratory analysis not specified in the protocol

3.10.1 Medications

All psychotropic medication prescribed at each time point (Baseline, 6 weeks, 26 weeks) for each participant are reported below. The WHO Defined Daily Dose (DDD) (https://www.whocc.no) is also presented for each medication type. It is defined as, "the assumed average maintenance dose per day for a drug used for its main indication in adults."

For antipsychotics, both the DDD and chlorpromazine equivalent (CPZequiv) can be identified. The CPZequiv was calculated using the table produced by Woods et al (2003) (https://pubmed.ncbi.nlm.nih.gov/12823080/). is also presented in the table.

DDD values have a range of 0-10 while the CPZ has a range of 0-2000.

TABLE 22: SUMMARY STATISTICS FOR MEDICATIONS, BY RANDOMISED GROUP

**Medication**	**VR+TAU (N=174)**	**TAU (N=172)**
**Baseline**		
**Antipsychotic, n (%)**	161 (92.5)	156 (90.7)
DDD, mean (SD) [n]	*1.3 (0.8) [160]*	*1.3 (0.9) [156]*
CPZ, mean (SD) [n]	*370.5 (298.3) [159]*	*362.4 (314.0) [155]*
**Stimulant, n (%)**	1 (0.6)	-
DDD, mean (SD) [n]	-	*1.2 (.) [1]*
**Antidepressant, n (%)**	103 (59.2)	96 (55.8)
DDD, mean (SD) [n]	*2.1 (1.2) [103]*	*2.1 (1.3) [96]*
**Anxiolytic, n (%)**	15 (8.6)	13 (7.6)
DDD, mean (SD) [n]	*0.9 (0.7) [15]*	*1.5 (1.0) [13]*
**Mood stabiliser, n (%)**	18 (10.3)	15 (8.7)
DDD, mean (SD) [n]	*0.7 (0.4) [18]*	*0.8 (0.3) [15]*
**Hypnotic, n (%)**	11 (6.3)	7 (4.1)
DDD, mean (SD) [n]	*1.0 (0.4) [11]*	*1.0 (0.0) [7]*
**PRN Medications prescribed, n (%)**	25 (14.4)	28 (16.3)

**Medication**	**VR+TAU (N=174)**	**TAU (N=172)**
**Total number of psychotropic medications prescribed, mean (SD) [n]**	*2.2 (1.2) [172]*	*2.2 (1.2) [170]*
**6 weeks**		
**Antipsychotic, n (%)**	155 (89.1)	155 (90.1)
DDD, mean (SD) [n]	*1.3 (0.8) [154]*	*1.3 (0.9) [155]*
CPZ, mean (SD) [n]	*385.1 (304.4) [154]*	*387.0 (326.3) [154]*
**Stimulant, n (%)**	1 (0.6)	1 (0.6)
DDD, mean (SD) [n]	*1.2 (.) [1]*	*1.0 (.) [1]*
**Antidepressant, n (%)**	94 (54.0)	94 (54.7)
DDD, mean (SD) [n]	*2.0 (1.2) [94]*	*2.2 (1.3) [94]*
**Anxiolytic, n (%)**	12 (6.9)	14 (8.1)
DDD, mean (SD) [n]	*0.9 (0.8) [12]*	*1.5 (1.0) [14]*
**Mood stabiliser, n (%)**	16 (9.2)	17 (9.9)
DDD, mean (SD) [n]	*0.7 (0.4) [16]*	*0.8 (0.3) [17]*
**Hypnotic, n (%)**	7 (4.0)	5 (2.9)
DDD, mean (SD) [n]	*0.8 (0.3) [6]*	*1.0 (0.0) [5]*
**PRN Medications prescribed, n (%)**	24 (13.8)	30 (17.4)
**Total number of psychotropic medications prescribed, mean (SD) [n]**	*2.1 (1.1) [172]*	*2.2 (1.2) [170]*
**26 weeks**		
**Antipsychotic, n (%)**	152 (87.4)	150 (87.2)
DDD, mean (SD) [n]	*1.3 (0.9) [152]*	*1.3 (0.9) [150]*
CPZ, mean (SD) [n]	*370.5 (298.3) [159]*	*362.4 (314.0) [155]*
**Stimulant, n (%)**	-	-
**Antidepressant, n (%)**	96 (55.2)	98 (57.0)

**Medication**	**VR+TAU**<br><br>**(N=174)**	**TAU**<br><br>**(N=172)**
DDD, mean (SD) [n]	*2.0 (1.1) [96]*	*2.1 (1.3) [98]*
**Anxiolytic, n (%)**	15 (8.6)	19 (11.0)
DDD, mean (SD) [n]	*1.3 (0.8) [15]*	*1.2 (0.9) [19]*
**Mood stabiliser, n (%)**	15 (8.6)	18 (10.5)
DDD, mean (SD) [n]	*0.7 (0.4) [15]*	*1.2 (1.6) [18]*
**Hypnotic, n (%)**	5 (2.9)	2 (1.2)
DDD, mean (SD) [n]	*1.4 (0.5) [5]*	*1.0 (0.0) [2]*
**PRN Medications prescribed, n (%)**	28 (16.1)	30 (17.4)
**Total number of psychotropic medications prescribed, mean (SD) [n]**	*2.1 (1.2) [170]*	*2.2 (1.2) [169]*

3.10.2 Service use

TABLE 23: SUMMARY STATISTICS FOR SERVICE USE, BY RANDOMISED GROUPS

Service Use	VR+TAU<br>(N=174)	TAU<br>(N=172)
**6 months before the trial**		
**Psychiatric inpatient admission (total number of nights), (n/N), mean (SD), [min to max], {n (%)}[1]**	(18/18), 50.4 (52.3), [0.0 to 182.5], {18 (10.3)}	(15/19), 49.0 (49.5), [1.0 to 182.0], {15 (8.7)}
**Physical health admission (total number of nights), mean (SD) [n][2]**	46.8 (57.4) [22]	41.2 (53.4) [23]
**Attendance at Accident and Emergency, n (%)**	30 (17.2)	38 (22.1)
**Meetings with psychiatrist, n (%)**	119 (68.4)	111 (64.5)
**Meetings with care coordinator (CPN or social worker), n (%)**	151 (86.8)	149 (86.6)
**Meetings with counsellor or therapist, n (%)**	43 (24.7)	49 (28.5)
**Visits to day-care centre / day hospital, n (%)**	10 (5.7)	8 (4.7)
**GP meetings, n (%)**	105 (60.3)	97 (56.4)
**During trial participation: Baseline to 6 weeks (post-treatment) assessment**		
**Psychiatric inpatient admission (total number of nights), (n/N), mean (SD), [min to max], {n (%)}[1]**	(3/3), 35.7 (11.0), [23.0 to 42.0], {3 (1.7)}	(1/1), 42.0 (.), [42.0 to 42.0], {1 (0.6)}

**Service Use**	**VR+TAU (N=174)**	**TAU (N=172)**
**Physical health admission (total number of nights), mean (SD) [n]**	26.3 (20.1) [4]	3.0 (1.7) [3]
**Attendance at Accident and Emergency, n (%)**	5 (2.9)	7 (4.1)
**Meetings with psychiatrist, n (%)**	44 (25.3)	52 (30.2)
**Meetings with care coordinator (CPN or social worker), n (%)**	126 (72.4)	124 (72.1)
**Meetings with counsellor or therapist, n (%)**	9 (5.2)	14 (8.1)
**Visits to day-care centre / day hospital, n (%)**	6 (3.4)	4 (2.3)
**GP meetings, n (%)**	41 (23.6)	49 (28.5)
**During trial participation: 6 weeks to 26 weeks (follow-up) assessment**		
**Psychiatric inpatient admission (total number of nights), (n/N), mean (SD), [min to max], {n (%)}[1]**	(6/6), 46.7 (46.1), [21.0 to 140.0], {6 (3.4)}	(4/4), 54.0 (60.1), [8.0 to 140.0], {4 (2.3)}
**Physical health admission (total number of nights), mean (SD) [n]**	22.1 (33.4) [8]	8.7 (13.3) [3]
**Attendance at Accident and Emergency, n (%)**	10 (5.7)	10 (5.8)
**Meetings with psychiatrist, n (%)**	61 (35.1)	59 (34.3)
**Meetings with care coordinator (CPN or social worker), n (%)**	113 (64.9)	106 (61.6)
**Meetings with counsellor or therapist, n (%)**	21 (12.1)	21 (12.2)

**Service Use**	**VR+TAU**<br><br>**(N=174)**	**TAU**<br><br>**(N=172)**
**Visits to day-care centre / day hospital, n (%)**	3 (1.7)	5 (2.9)
**GP meetings, n (%)**	51 (29.3)	51 (29.7)

[1] Percentage out of total number in each treatment group respectively. Summary stats for the whole group are: N, mean (SD), [range] = (baseline) 33, 50.7 (53.1), [0.0 to 214.0], (post-treatment) 4, 37.3 (9.5), [23.0 to 42.0], (follow-up) 10, 50.4 (50.6), [8.0 to 144.0]
[2] Durations were censored at 182.5 days for any admissions longer than 6 months
[3] Durations were censored at 42 days for any admissions longer than 6 weeks
† Durations were censored at 140 days for any admissions longer than 20 (26 minus 6) weeks

3.10.3 Standard care

TABLE 24: SUMMARY OF STANDARD CARE (I.E. PSYCHOLOGICAL THERAPY) BY RANDOMISED GROUP

Standard care	VR+TAU (N=174)	TAU (N=172)	Total (N=346)
**Any other therapy – baseline**			
**Cognitive behaviour therapy-Psychosis**	8 (4.6)	6 (3.5)	14 (4.0)
**Cognitive behaviour therapy (other)**	13 (7.5)	8 (4.7)	21 (6.1)
**Family therapy**	12 (6.9)	2 (1.2)	14 (4.0)
**Trauma related therapy**	4 (2.3)	3 (1.7)	7 (2.0)
**Art therapy**	2 (1.1)	1 (0.6)	3 (0.9)
**Other psychological therapy**	4 (2.3)	7 (4.1)	11 (3.2)
**Group therapy**	-	3 (1.7)	3 (0.9)
**Any other therapy – 6 weeks**			
**Cognitive behaviour therapy-Psychosis**	1 (0.6)	1 (0.6)	2 (0.6)
**Cognitive behaviour therapy (other)**	-	1 (0.6)	1 (0.3)
**Family therapy**	3 (1.7)	3 (1.7)	6 (1.7)
**Trauma related therapy**	-	1 (0.6)	1 (0.3)
**Art therapy**	1 (0.6)	-	1 (0.3)
**Other psychological therapy**	1 (0.6)	2 (1.2)	3 (0.9)
**Group therapy**	2 (1.1)	-	2 (0.6)
**Any other therapy – 26 weeks**			
**Cognitive behaviour therapy-Psychosis**	6 (3.4)	6 (3.5)	12 (3.5)
**Cognitive behaviour therapy (other)**	7 (4.0)	8 (4.7)	15 (4.3)
**Family therapy**	5 (2.9)	3 (1.7)	8 (2.3)
**Trauma related therapy**	2 (1.1)	1 (0.6)	3 (0.9)
**Art therapy**	1 (0.6)	-	1 (0.3)
**Other psychological therapy**	5 (2.9)	5 (2.9)	10 (2.9)

Standard care	VR+TAU (N=174)	TAU (N=172)	Total (N=346)
**Group therapy**	1 (0.6)	-	1 (0.3)

3.11 Additional exploratory analysis not specified in the SAP

3.11.1 O-AS Avoidance and Distress by Treatment deliverer

TABLE 25: SUMMARY OF PRIMARY OUTCOMES AT 6 WEEKS BY TREATMENT DELIVERER

	O-AS Avoidance, mean (SD) [n]	O-AS Distress, mean (SD) [n]
Psychologist	2.0 (2.2) [104]	42.1 (18.6) [104]
Assistant psychologist	0.7 (1.3) [18]	28.4 (18.2) [18]
Peer worker	2.1 (2.2) [28]	44.9 (17.1) [28]
Other	3.0 (2.6) [3]	33.3 (11.7) [3]

3.11.2 O-AS Avoidance and Distress by Site

TABLE 26: SUMMARY OF PRIMARY OUTCOMES AT 6 WEEKS BY SITE

	VR+TAU (N=174)	TAU (N=172)
**O-AS Avoidance, mean (SD) [n]**		
Site 1	1.8 (2.1)	2.1 (2.2)
Site 2	2.7 (2.2)	3.3 (2.9)
Site 3	2.2 (2.7)	3.2 (2.8)
Site 4	2.0 (2.0)	2.3 (2.7)
Site 5	1.0 (1.4)	1.9 (2.5)
**O-AS Distress, mean (SD) [n]**		
Site 1	43.1 (17.4)	44.2 (16.9)
Site 2	45.6 (17.6)	45.8 (23.7)
Site 3	45.8 (19.5)	53.6 (18.6)
Site 4	39.4 (18.3)	47.0 (18.8)
Site 5	31.1 (17.7)	37.9 (22.4)

3.11.3 Additional subgroup analyses

3.11.3.1 O-AS Avoidance

TABLE 27: FURTHER RESULTS FOR THE SUBGROUP ANALYSIS ON O-AS AVOIDANCE AT 6 WEEKS

O-AS Avoidance at 6 weeks	VR+TAU (N=174)	TAU (N=172)	Adjusted mean difference [95% CI][1]	Test of Interaction (P value)‡
**Quartiles of O-CDBQ Part 1**				
≤12	34	47	0.19 [-0.63 to 1.00]	0.082
13-19	42	47	-0.56 [-1.33 to 0.21]	
20-26	40	35	-0.40 [-1.25 to 0.45]	
27-42	41	29	-1.39 [-2.26 to -0.51]	
**Severity ranges of O-AS at baseline[2]**				
0: Average avoidance	27	32	0.26 [-0.74 to 1.25]	0.014
1-2: Moderate avoidance	53	37	0.08 [-0.75 to 0.91]	
3-5: High avoidance	43	48	-0.34 [-1.14 to 0.47]	
≥6: Severe avoidance	36	43	-1.63 [-2.49 to -0.77]	

[1] VR+TAU vs. TAU: Linear regression model for the primary outcome; modelled against treatment group, outcome score at baseline, stratification factors (site and service type) and an interaction between randomised group and the subgroup variable.

[2] VR+TAU vs. TAU: Linear regression model for the primary outcome; modelled against treatment group, stratification factors (site and service type) and an interaction between randomised group and severity of the primary at baseline. ‡Level of significance = 0.05

TABLE 28: FURTHER RESULTS FOR THE SUBGROUP ANALYSIS ON O-AS AVOIDANCE AT 26 WEEKS

O-AS Avoidance at 26 weeks	Group VR+TAU (N=174)	Group TAU (N=172)	Adjusted mean difference [95% CI][1]	Test of Interaction (P value)‡
**Quartiles of O-CDBQ Part 1**				
≤12	34	47	-0.06 [-0.91 to 0.79]	0.756
13-19	41	46	-0.67 [-1.49 to 0.14]	
20-26	35	36	-0.57 [-1.47 to 0.34]	
27-42	44	28	-0.50 [-1.41 to 0.41]	

O-AS Avoidance at 26 weeks	Group VR+TAU (N=174)	Group TAU (N=172)	Adjusted mean difference [95% CI][1]	Test of Interaction (P value)‡
**Severity ranges of O-AS at baseline[2]**				
0: Average avoidance	25	31	-0.00 [-1.02 to 1.01]	<0.001
1-2: Moderate avoidance	50	38	0.10 [-0.73 to 0.93]	
3-5: High avoidance	45	48	0.33 [-0.45 to 1.12]	
≥6: Severe avoidance	36	42	-2.06 [-2.91 to -1.20]	

[1] VR+TAU vs. TAU: Linear regression model for the primary outcome; modelled against treatment group, outcome score at baseline, stratification factors (site and service type) and an interaction between randomised group and the subgroup variable.

[2] VR+TAU vs. TAU: Linear regression model for the primary outcome; modelled against treatment group, stratification factors (site and service type) and an interaction between randomised group and severity of the primary at baseline. ‡Level of significance = 0.05

3.11.3.2 O-AS Distress

TABLE 29: FURTHER RESULTS FOR THE SUBGROUP ANALYSIS ON O-AS DISTRESS AT 6 WEEKS

O-AS Distress at 6 weeks	Group VR+TAU (N=174)	Group TAU (N=172)	Adjusted mean difference [95% CI][1]	Test of Interaction (P value)‡
**Quartiles of O-CDBQ Part 1**				
≤12	34	47	1.94 [-4.65 to 8.54]	0.079
13-19	42	47	-4.67 [-10.89 to 1.54]	
20-26	40	37	-4.10 [-10.78 to 2.57]	
27-42	41	29	-10.91 [-17.99 to -3.84]	
**Severity ranges of O-AS at baseline[2]**				
≤23: Average avoidance	7	11	-0.78 [-15.51 to 13.95]	0.219
24-46: Moderate avoidance	56	43	-0.30 [-6.49 to 5.88]	
46-66: High avoidance	63	71	-4.11 [-9.44 to 1.22]	

O-AS Distress at 6 weeks	Group VR+TAU (N=174)	Group TAU (N=172)	Adjusted mean difference [95% CI][1]	Test of Interaction (P value)‡
66+: Severe avoidance	34	37	-10.17 [-17.34 to -3.00]	

[1] VR+TAU vs. TAU: Linear regression model for the primary outcome; modelled against treatment group, outcome score at baseline, stratification factors (site and service type) and an interaction between randomised group and the subgroup variable.
[2] VR+TAU vs. TAU: Linear regression model for the primary outcome; modelled against treatment group, stratification factors (site and service type) and an interaction between randomised group and severity of the primary at baseline. ‡Level of significance = 0.05

TABLE 30: FURTHER RESULTS FOR THE SUBGROUP ANALYSIS ON O-AS DISTRESS AT 26 WEEKS

O-AS Distress at 26 weeks	Group VR+TAU (N=174)	Group TAU (N=172)	Adjusted mean difference [95% CI][1]	Test of Interaction (P value)‡
**Quartiles of O-CDBQ Part 1**				
≤12	33	48	3.95 [-3.59 to 11.50]	0.039
13-19	41	46	-4.84 [-12.03 to 2.35]	
20-26	35	36	-1.40 [-9.35 to 6.55]	
27-42	44	29	-11.89 [-19.87 to -3.92]	
**Severity ranges of O-AS at baseline[2]**				
≤23: Average avoidance	7	11	7.36 [-9.48 to 24.20]	0.050
24-46: Moderate avoidance	53	44	3.78 [-3.33 to 10.88]	
46-66: High avoidance	63	69	-6.37 [-12.48 to -0.27]	
66+: Severe avoidance	33	37	-8.47 [-16.73 to -0.21]	

[1] VR+TAU vs. TAU: Linear regression model for the primary outcome; modelled against treatment group, outcome score at baseline, stratification factors (site and service type) and an interaction between randomised group and the subgroup variable.
[2] VR+TAU vs. TAU: Linear regression model for the primary outcome; modelled against treatment group, stratification factors (site and service type) and an interaction between randomised group and severity of the primary at baseline. ‡Level of significance = 0.05

3.11.4 Post-hoc subgroup of secondary outcomes by O-AS at baseline

The table below provides summary statistics for the Secondary outcomes at baseline, by O-AS avoidance categories:

	VR+TAU (N=174)	TAU (N=172)
**Agoraphobia Mobility Inventory-Avoidance [AMI-A]**		
0: Average avoidance, mean (SD) [n]	2.7 (0.7) [27]	2.8 (0.6) [32]
1-2: Moderate avoidance, mean (SD) [n]	3.0 (0.7) [52]	2.7 (0.6) [39]
3-5: High avoidance, mean (SD) [n]	3.4 (0.4) [48]	3.3 (0.6) [47]
≥6: Severe avoidance, mean (SD) [n]	3.9 (0.6) [39]	4.0 (0.7) [46]
**R-GPTS Part A**		
0: Average avoidance, mean (SD) [n]	12.2 (9.5) [25]	11.8 (8.4) [32]
1-2: Moderate avoidance, mean (SD) [n]	13.7 (9.5) [50]	11.2 (9.1) [38]
3-5: High avoidance, mean (SD) [n]	13.6 (7.9) [44]	12.4 (8.9) [46]
≥6: Severe avoidance, mean (SD) [n]	15.9 (10.3) [38]	14.4 (9.8) [45]
**R-GPTS Part B**		
0: Average avoidance, mean (SD) [n]	14.8 (12.4) [25]	11.5 (10.3) [32]
1-2: Moderate avoidance, mean (SD) [n]	16.1 (13.2) [50]	10.7 (10.5) [38]
3-5: High avoidance, mean (SD) [n]	18.3 (12.0) [44]	16.3 (13.5) [46]
≥6: Severe avoidance, mean (SD) [n]	19.1 (13.2) [38]	16.8 (15.0) [45]
**R-GPTS Overall total**		
0: Average avoidance, mean (SD) [n]	27.0 (21.4) [25]	23.3 (17.5) [32]
1-2: Moderate avoidance, mean (SD) [n]	29.8 (21.4) [50]	21.9 (18.6) [38]
3-5: High avoidance, mean (SD) [n]	32.0 (18.1) [44]	28.6 (21.0) [46]
≥6: Severe avoidance, mean (SD) [n]	35.0 (22.4) [38]	31.2 (23.6) [45]

	VR+TAU (N=174)	TAU (N=172)
**Paranoia worries Questionnaire [PWQ]**		
0: Average avoidance, mean (SD) [n]	7.6 (6.0) [25]	7.5 (5.3) [30]
1-2: Moderate avoidance, mean (SD) [n]	9.5 (6.4) [49]	7.3 (5.7) [37]
3-5: High avoidance, mean (SD) [n]	10.2 (6.8) [45]	9.8 (6.4) [45]
≥6: Severe avoidance, mean (SD) [n]	11.4 (5.3) [38]	10.3 (6.6) [44]
**Patient health Questionnaire (Depression, anxiety and stress scale) [PHQ-9]**		
0: Average avoidance, mean (SD) [n]	14.2 (6.2) [26]	14.3 (6.0) [32]
1-2: Moderate avoidance, mean (SD) [n]	14.4 (6.2) [52]	11.9 (6.5) [38]
3-5: High avoidance, mean (SD) [n]	15.2 (5.2) [49]	12.9 (6.4) [47]
≥6: Severe avoidance, mean (SD) [n]	16.6 (6.7) [38]	16.9 (6.1) [45]
**Suicidal Ideation**		
0: Average avoidance, mean (SD) [n]	0.5 (1.2) [25]	0.8 (1.3) [30]
1-2: Moderate avoidance, mean (SD) [n]	1.3 (1.3) [48]	0.7 (1.1) [35]
3-5: High avoidance, mean (SD) [n]	1.0 (1.2) [46]	1.1 (1.4) [45]
≥6: Severe avoidance, mean (SD) [n]	1.2 (1.3) [35]	1.2 (1.3) [44]
**Quality of life**		
0: Average avoidance, mean (SD) [n]	0.7 (0.2) [28]	0.6 (0.2) [33]
1-2: Moderate avoidance, mean (SD) [n]	0.6 (0.3) [55]	0.6 (0.2) [40]
3-5: High avoidance, mean (SD) [n]	0.5 (0.3) [49]	0.6 (0.3) [50]
≥6: Severe avoidance, mean (SD) [n]	0.4 (0.3) [39]	0.4 (0.3) [47]
**The EQ5D VAS**		
0: Average avoidance, mean (SD) [n]	62.9 (17.3) [28]	51.7 (16.9) [33]
1-2: Moderate avoidance, mean (SD) [n]	52.9 (18.4) [55]	59.8 (17.1) [39]
3-5: High avoidance, mean (SD) [n]	47.5 (19.7) [48]	52.9 (21.1) [51]

	VR+TAU (N=174)	TAU (N=172)
≥6: Severe avoidance, mean (SD) [n]	46.3 (18.1) [39]	49.0 (19.0) [47]
**Recovering quality of life [REQOL-20]**		
0: Average avoidance, mean (SD) [n]	41.3 (13.7) [27]	39.6 (9.3) [32]
1-2: Moderate avoidance, mean (SD) [n]	35.0 (11.3) [51]	38.1 (14.2) [40]
3-5: High avoidance, mean (SD) [n]	32.0 (12.6) [48]	37.2 (13.8) [46]
≥6: Severe avoidance, mean (SD) [n]	28.4 (13.9) [39]	28.6 (11.5) [45]
**Questionnaire on progress of recovery [QPR]**		
0: Average avoidance, mean (SD) [n]	33.0 (7.0) [29]	32.5 (8.1) [33]
1-2: Moderate avoidance, mean (SD) [n]	27.9 (10.3) [54]	29.9 (10.6) [40]
3-5: High avoidance, mean (SD) [n]	26.0 (10.8) [50]	29.5 (11.6) [50]
≥6: Severe avoidance, mean (SD) [n]	23.4 (11.8) [39]	21.9 (10.7) [47]

TABLE 31 FURTHER RESULTS FOR THE SUBGROUP ANALYSIS ON SECONDARY OUTCOMES AT 6 WEEKS BY O-AS AVOIDANCE

	VR+TAU (N=174)	TAU (N=172)	Adjusted mean difference [95% CI][1]	Test of Interaction (P value)[1]
**Agoraphobia Mobility Inventory-Avoidance [AMI-A]**				
0: Average avoidance, mean (SD) [n]	2.5 (0.7) [25]	2.6 (0.7) [31]	0.06 [-0.26 to 0.38]	0.214
1-2: Moderate avoidance, mean (SD) [n]	2.8 (0.6) [51]	2.5 (0.7) [34]	-0.09 [-0.36 to 0.17]	
3-5: High avoidance, mean (SD) [n]	3.0 (0.7) [40]	3.0 (0.8) [46]	-0.05 [-0.31 to 0.20]	
≥6: Severe avoidance, mean (SD) [n]	3.3 (1.0) [35]	3.8 (0.7) [41]	-0.35 [-0.62 to -0.09]	
**R-GPTS Part A**				

	VR+TAU (N=174)	TAU (N=172)	Adjusted mean difference [95% CI][1]	Test of Interaction (P value)[1]
0: Average avoidance, mean (SD) [n]	9.4 (8.1) [24]	9.6 (7.8) [31]	0.10 [-3.27 to 3.46]	0.045
1-2: Moderate avoidance, mean (SD) [n]	10.5 (7.7) [47]	8.6 (7.3) [34]	-1.03 [-3.90 to 1.83]	
3-5: High avoidance, mean (SD) [n]	11.8 (9.3) [37]	10.1 (8.8) [42]	0.71 [-2.11 to 3.54]	
≥6: Severe avoidance, mean (SD) [n]	10.5 (9.2) [33]	13.8 (8.8) [39]	-4.73 [-7.62 to -1.84]	
**R-GPTS Part B**				
0: Average avoidance, mean (SD) [n]	12.4 (12.1) [24]	9.9 (10.6) [31]	1.53 [-2.91 to 5.97]	0.001
1-2: Moderate avoidance, mean (SD) [n]	12.7 (11.1) [47]	7.8 (9.9) [34]	-1.14 [-4.92 to 2.65]	
3-5: High avoidance, mean (SD) [n]	15.8 (12.7) [37]	12.5 (12.7) [42]	1.79 [-1.93 to 5.52]	
≥6: Severe avoidance, mean (SD) [n]	11.0 (12.0) [33]	17.5 (14.4) [39]	-8.16 [-11.97 to -4.35]	
**R-GPTS Overall total**				
0: Average avoidance, mean (SD) [n]	21.8 (19.4) [24]	19.5 (16.8) [31]	1.59 [-5.45 to 8.63]	0.002
1-2: Moderate avoidance, mean (SD) [n]	23.2 (17.5) [47]	16.4 (16.4) [34]	-2.44 [-8.44 to 3.56]	
3-5: High avoidance, mean (SD) [n]	27.6 (20.5) [37]	22.7 (20.2) [42]	2.43 [-3.48 to 8.33]	
≥6: Severe avoidance, mean (SD) [n]	21.5 (20.4) [33]	31.4 (22.4) [39]	-13.07 [-19.11 to -7.03]	
**Paranoia worries questionnaire [PWQ]**				
0: Average avoidance, mean (SD) [n]	7.2 (5.7) [24]	5.7 (4.7) [31]	0.89 [-1.54 to 3.31]	0.006
1-2: Moderate avoidance, mean (SD) [n]	6.4 (5.4) [45]	5.1 (5.5) [33]	-0.64 [-2.73 to 1.45]	

	VR+TAU (N=174)	TAU (N=172)	Adjusted mean difference [95% CI][1]	Test of Interaction (P value)[1]
3-5: High avoidance, mean (SD) [n]	9.7 (6.4) [38]	8.1 (6.2) [43]	1.73 [-0.24 to 3.70]	
≥6: Severe avoidance, mean (SD) [n]	7.8 (6.7) [33]	10.3 (6.5) [38]	-3.14 [-5.20 to -1.08]	
**Patient health questionnaire (Depression, anxiety and stress scale) [PHQ-9]**				
0: Average avoidance, mean (SD) [n]	12.0 (6.6) [23]	11.3 (5.4) [32]	0.83 [-1.93 to 3.60]	0.646
1-2: Moderate avoidance, mean (SD) [n]	11.6 (5.4) [51]	10.3 (5.7) [35]	0.06 [-2.24 to 2.36]	
3-5: High avoidance, mean (SD) [n]	13.9 (6.4) [38]	12.0 (6.6) [43]	0.34 [-1.93 to 2.62]	
≥6: Severe avoidance, mean (SD) [n]	12.9 (6.9) [34]	14.4 (5.5) [40]	-1.33 [-3.68 to 1.02]	
**Suicidal Ideation**				
0: Average avoidance, mean (SD) [n]	0.3 (0.8) [23]	0.5 (0.9) [24]	-0.03 [-0.44 to 0.39]	0.444
1-2: Moderate avoidance, mean (SD) [n]	0.9 (1.1) [36]	0.6 (1.2) [30]	-0.30 [-0.67 to 0.06]	
3-5: High avoidance, mean (SD) [n]	0.9 (1.3) [35]	0.7 (1.2) [34]	0.08 [-0.26 to 0.43]	
≥6: Severe avoidance, mean (SD) [n]	1.1 (1.6) [27]	1.4 (1.7) [35]	-0.19 [-0.56 to 0.17]	
**Quality of life**				
0: Average avoidance, mean (SD) [n]	0.7 (0.3) [24]	0.7 (0.2) [32]	-0.01 [-0.13 to 0.11]	0.524
1-2: Moderate avoidance, mean (SD) [n]	0.6 (0.3) [51]	0.7 (0.2) [34]	0.00 [-0.10 to 0.10]	
3-5: High avoidance, mean (SD) [n]	0.6 (0.3) [41]	0.6 (0.3) [47]	0.09 [-0.01 to 0.18]	
≥6: Severe avoidance, mean (SD) [n]	0.5 (0.3) [35]	0.5 (0.3) [42]	0.01 [-0.09 to 0.11]	
**The EQ5D VAS**				

	VR+TAU (N=174)	TAU (N=172)	Adjusted mean difference [95% CI][1]	Test of Interaction (P value)[1]
0: Average avoidance, mean (SD) [n]	59.2 (22.6) [25]	57.5 (20.1) [32]	-2.96 [-12.64 to 6.71]	0.184
1-2: Moderate avoidance, mean (SD) [n]	58.8 (18.0) [51]	59.1 (19.0) [35]	7.73 [-0.48 to 15.95]	
3-5: High avoidance, mean (SD) [n]	52.1 (19.0) [41]	54.8 (22.1) [47]	-1.31 [-9.08 to 6.46]	
≥6: Severe avoidance, mean (SD) [n]	56.4 (20.6) [35]	49.4 (20.5) [42]	7.02 [-1.21 to 15.25]	
**Recovering quality of life [REQOL-20]**				
0: Average avoidance, mean (SD) [n]	40.4 (13.0) [24]	43.4 (11.8) [31]	-2.97 [-8.45 to 2.50]	0.023
1-2: Moderate avoidance, mean (SD) [n]	39.5 (11.2) [49]	45.0 (13.6) [35]	-1.96 [-6.57 to 2.66]	
3-5: High avoidance, mean (SD) [n]	35.4 (12.4) [40]	40.1 (15.0) [42]	0.51 [-4.05 to 5.08]	
≥6: Severe avoidance, mean (SD) [n]	37.5 (18.7) [35]	30.6 (13.2) [39]	6.90 [2.20 to 11.60]	
**Questionnaire on progress of recovery [QPR]**				
0: Average avoidance, mean (SD) [n]	35.1 (10.2) [26]	33.5 (8.4) [32]	1.49 [-2.83 to 5.82]	0.819
1-2: Moderate avoidance, mean (SD) [n]	34.0 (9.0) [54]	34.9 (8.9) [35]	1.84 [-1.82 to 5.49]	
3-5: High avoidance, mean (SD) [n]	30.6 (11.6) [43]	31.4 (12.5) [48]	2.35 [-1.13 to 5.83]	
≥6: Severe avoidance, mean (SD) [n]	30.2 (13.8) [35]	25.6 (11.7) [44]	3.95 [0.23 to 7.68]	

[1] VR+TAU vs. TAU: Linear regression model for the primary outcome; modelled against treatment group, outcome score at baseline, stratification factors (site and service type) and an interaction between randomised group and the subgroup variable.

‡Level of significance = 0.05

TABLE 32 FURTHER RESULTS FOR THE SUBGROUP ANALYSIS ON SECONDARY OUTCOMES AT 26 WEEKS BY O-AS AVOIDANCE

	VR+TAU (N=174)	TAU (N=172)	Adjusted mean difference [95% CI][1]	Test of Interaction (P value)‡
**Agoraphobia Mobility Inventory-Avoidance [AMI-A]**				
0: Average avoidance, mean (SD) [n]	2.3 (0.8) [22]	2.5 (0.8) [30]	-0.10 [-0.45 to 0.26]	0.004
1-2: Moderate avoidance, mean (SD) [n]	2.9 (0.6) [50]	2.5 (0.8) [36]	0.09 [-0.19 to 0.37]	
3-5: High avoidance, mean (SD) [n]	3.1 (0.5) [39]	2.9 (0.8) [44]	0.05 [-0.23 to 0.33]	
≥6: Severe avoidance, mean (SD) [n]	3.2 (1.0) [34]	3.9 (0.6) [35]	-0.61 [-0.91 to -0.31]	
**R-GPTS Part A**				
0: Average avoidance, mean (SD) [n]	7.6 (7.1) [19]	8.7 (8.3) [29]	-0.33 [-4.64 to 3.98]	0.523
1-2: Moderate avoidance, mean (SD) [n]	11.4 (8.7) [45]	9.3 (8.6) [35]	0.37 [-3.01 to 3.75]	
3-5: High avoidance, mean (SD) [n]	11.4 (10.4) [34]	10.4 (8.9) [38]	0.48 [-3.08 to 4.03]	
≥6: Severe avoidance, mean (SD) [n]	11.9 (11.3) [34]	13.1 (10.1) [32]	-2.90 [-6.53 to 0.74]	
**R-GPTS Part B**				
0: Average avoidance, mean (SD) [n]	10.3 (10.5) [19]	8.9 (10.5) [29]	0.70 [-4.83 to 6.22]	0.185
1-2: Moderate avoidance, mean (SD) [n]	13.1 (12.2) [45]	8.4 (9.6) [35]	0.72 [-3.63 to 5.07]	
3-5: High avoidance, mean (SD) [n]	13.9 (13.4) [34]	12.9 (12.7) [38]	1.53 [-3.02 to 6.09]	
≥6: Severe avoidance, mean (SD) [n]	12.9 (13.8) [34]	16.4 (15.5) [32]	-4.98 [-9.63 to -0.32]	
**R-GPTS Overall total**				
0: Average avoidance, mean (SD) [n]	17.9 (16.4) [19]	17.6 (17.7) [29]	0.40 [-8.74 to 9.54]	0.246

	VR+TAU (N=174)	TAU (N=172)	Adjusted mean difference [95% CI][1]	Test of Interaction (P value)‡
1-2: Moderate avoidance, mean (SD) [n]	24.5 (19.9) [45]	17.7 (17.2) [35]	0.98 [-6.20 to 8.16]	
3-5: High avoidance, mean (SD) [n]	25.4 (23.0) [34]	23.3 (19.7) [38]	1.97 [-5.56 to 9.51]	
≥6: Severe avoidance, mean (SD) [n]	24.8 (24.3) [34]	29.5 (24.8) [32]	-7.96 [-15.67 to -0.26]	
**Paranoia worries Questionnaire [PWQ]**				
0: Average avoidance, mean (SD) [n]	5.7 (5.7) [19]	5.8 (5.3) [29]	-0.22 [-3.32 to 2.89]	0.219
1-2: Moderate avoidance, mean (SD) [n]	7.0 (5.7) [42]	5.2 (6.0) [35]	0.53 [-1.94 to 3.00]	
3-5: High avoidance, mean (SD) [n]	8.3 (6.5) [33]	7.8 (6.7) [40]	0.95 [-1.55 to 3.44]	
≥6: Severe avoidance, mean (SD) [n]	7.5 (6.7) [32]	9.6 (7.2) [30]	-2.64 [-5.29 to 0.00]	
**Patient health Questionnaire (Depression, anxiety and stress scale) [PHQ-9]**				
0: Average avoidance, mean (SD) [n]	11.8 (7.2) [19]	10.5 (6.1) [30]	1.25 [-2.15 to 4.64]	0.295
1-2: Moderate avoidance, mean (SD) [n]	12.0 (6.1) [46]	9.5 (6.1) [35]	0.80 [-1.85 to 3.46]	
3-5: High avoidance, mean (SD) [n]	13.2 (6.3) [35]	11.2 (6.7) [38]	0.83 [-1.89 to 3.56]	
≥6: Severe avoidance, mean (SD) [n]	12.8 (7.9) [33]	15.1 (6.5) [34]	-2.27 [-5.08 to 0.55]	
**Suicidal Ideation**				
0: Average avoidance, mean (SD) [n]	0.3 (0.8) [22]	0.5 (1.1) [24]	-0.02 [-0.46 to 0.43]	0.723
1-2: Moderate avoidance, mean (SD) [n]	0.8 (1.1) [31]	0.5 (1.1) [30]	-0.18 [-0.57 to 0.22]	
3-5: High avoidance, mean (SD) [n]	0.9 (1.4) [32]	0.5 (1.0) [30]	0.13 [-0.25 to 0.52]	

	VR+TAU (N=174)	TAU (N=172)	Adjusted mean difference [95% CI][1]	Test of Interaction (P value)‡
≥6: Severe avoidance, mean (SD) [n]	1.0 (1.7) [25]	1.1 (1.7) [29]	-0.09 [-0.50 to 0.32]	
**Quality of life**				
0: Average avoidance, mean (SD) [n]	0.6 (0.3) [22]	0.6 (0.2) [30]	-0.05 [-0.17 to 0.08]	0.314
1-2: Moderate avoidance, mean (SD) [n]	0.6 (0.3) [48]	0.7 (0.3) [36]	-0.07 [-0.16 to 0.03]	
3-5: High avoidance, mean (SD) [n]	0.6 (0.3) [38]	0.6 (0.3) [42]	0.04 [-0.06 to 0.14]	
≥6: Severe avoidance, mean (SD) [n]	0.5 (0.3) [33]	0.4 (0.3) [37]	0.05 [-0.06 to 0.15]	
**The EQ5D VAS**				
0: Average avoidance, mean (SD) [n]	55.5 (26.4) [22]	56.7 (20.6) [30]	-6.76 [-18.04 to 4.52]	0.442
1-2: Moderate avoidance, mean (SD) [n]	60.3 (17.8) [49]	65.1 (16.6) [36]	0.20 [-8.82 to 9.22]	
3-5: High avoidance, mean (SD) [n]	53.9 (20.9) [39]	57.0 (24.7) [42]	-2.39 [-11.38 to 6.59]	
≥6: Severe avoidance, mean (SD) [n]	52.8 (22.8) [34]	48.3 (23.7) [38]	4.96 [-4.42 to 14.34]	
**Recovering quality of life [REQOL-20]**				
0: Average avoidance, mean (SD) [n]	43.9 (15.8) [19]	45.3 (15.2) [29]	-3.00 [-10.27 to 4.28]	0.104
1-2: Moderate avoidance, mean (SD) [n]	40.6 (13.2) [47]	46.0 (15.1) [36]	-1.95 [-7.56 to 3.66]	
3-5: High avoidance, mean (SD) [n]	36.7 (14.0) [35]	40.7 (14.0) [39]	-0.65 [-6.51 to 5.20]	
≥6: Severe avoidance, mean (SD) [n]	38.1 (18.0) [34]	31.3 (12.5) [33]	6.96 [0.94 to 12.99]	
**Questionnaire on progress of recovery [QPR]**				
0: Average avoidance, mean (SD) [n]	37.1 (10.5) [23]	36.1 (8.3) [30]	1.10 [-4.04 to 6.24]	0.626

	VR+TAU (N=174)	TAU (N=172)	Adjusted mean difference [95% CI][1]	Test of Interaction (P value)‡
1-2: Moderate avoidance, mean (SD) [n]	33.9 (8.8) [50]	36.4 (9.1) [37]	0.47 [-3.63 to 4.57]	
3-5: High avoidance, mean (SD) [n]	31.7 (12.8) [40]	33.1 (12.6) [45]	0.57 [-3.48 to 4.63]	
≥6: Severe avoidance, mean (SD) [n]	30.6 (14.4) [34]	25.6 (13.8) [39]	4.02 [-0.35 to 8.39]	

[1] VR+TAU vs. TAU: Linear regression model for the primary outcome; modelled against treatment group, outcome score at baseline, stratification factors (site and service type) and an interaction between randomised group and the subgroup variable.

‡Level of significance = 0.05

TABLE 33 FURTHER RESULTS FOR THE SUBGROUP ANALYSIS ON SECONDARY OUTCOMES AT 6 WEEKS BY O-AS DISTRESS

	Group VR+TAU (N=174)	Group TAU (N=172)	Adjusted mean difference [95% CI][1]	Test of Interaction (P value)‡
**Agoraphobia Mobility Inventory-Avoidance [AMI-A]**				
≤23: Average distress	7	11	-0.00 [-0.57 to 0.56]	0.203
24-46: Moderate distress	52	40	-0.09 [-0.34 to 0.15]	
46-66: High distress	59	67	-0.02 [-0.23 to 0.19]	
66+: Severe distress	34	34	-0.39 [-0.67 to -0.11]	
**R-GPTS Part A**				
≤23: Average distress	7	10	1.77 [-4.37 to 7.90]	0.603
24-46: Moderate distress	47	39	-1.01 [-3.76 to 1.75]	
46-66: High distress	57	64	-1.51 [-3.81 to 0.80]	
66+: Severe distress	31	33	-2.78 [-5.93 to 0.36]	
**R-GPTS Part B**				
≤23: Average distress	7	10	4.51 [-3.60 to 12.62]	0.022
24-46: Moderate distress	47	39	-0.57 [-4.20 to 3.07]	
46-66: High distress	57	64	-0.80 [-3.83 to 2.24]	
66+: Severe distress	31	33	-7.19 [-11.34 to -3.03]	
**R-GPTS Overall total**				
≤23: Average distress	7	10	6.24 [-6.66 to 19.14]	0.072
24-46: Moderate distress	47	39	-1.53 [-7.32 to 4.25]	
46-66: High distress	57	64	-2.47 [-7.31 to 2.36]	
66+: Severe distress	31	33	-10.42 [-17.03 to -3.80]	
**Paranoia worries questionnaire [PWQ]**				
≤23: Average distress	7	10	3.69 [-0.71 to 8.09]	0.131
24-46: Moderate distress	46	40	-0.17 [-2.16 to 1.83]	
46-66: High distress	57	63	-0.53 [-2.21 to 1.14]	
66+: Severe distress	31	32	-2.14 [-4.38 to 0.10]	
**Patient health questionnaire (Depression, anxiety and stress scale) [PHQ-9]**				
≤23: Average distress	7	10	2.00 [-3.01 to 7.02]	0.460
24-46: Moderate distress	49	41	0.19 [-1.98 to 2.37]	
46-66: High distress	59	66	0.22 [-1.62 to 2.06]	
66+: Severe distress	32	33	-1.78 [-4.30 to 0.73]	
**Suicidal Ideation**				
≤23: Average distress	5	10	-0.02 [-0.81 to 0.78]	0.994
24-46: Moderate distress	41	29	-0.13 [-0.48 to 0.23]	
46-66: High distress	49	58	-0.13 [-0.41 to 0.15]	
66+: Severe distress	26	26	-0.14 [-0.55 to 0.26]	
**Quality of life**				
≤23: Average distress	6	10	-0.00 [-0.22 to 0.22]	0.871
24-46: Moderate distress	51	42	0.03 [-0.06 to 0.12]	
46-66: High distress	61	67	0.04 [-0.03 to 0.12]	
66+: Severe distress	34	36	-0.01 [-0.11 to 0.09]	
**The EQ5D VAS**				
≤23: Average distress	7	10	5.88 [-12.20 to 23.97]	0.722
24-46: Moderate distress	51	42	1.73 [-5.78 to 9.23]	

	Group VR+TAU (N=174)	Group TAU (N=172)	Adjusted mean difference [95% CI][1]	Test of Interaction (P value)‡
46-66: High distress	61	68	0.68 [-5.72 to 7.08]	
66+: Severe distress	34	36	6.45 [-2.06 to 14.95]	
**Recovering quality of life [REQOL-20]**				
≤23: Average distress	7	10	-10.82 [-20.83 to -0.80]	0.009
24-46: Moderate distress	50	41	0.86 [-3.43 to 5.15]	
46-66: High distress	58	63	-0.69 [-4.42 to 3.03]	
66+: Severe distress	34	33	6.96 [2.04 to 11.89]	
**Questionnaire on progress of recovery [QPR]**				
≤23: Average distress	7	11	0.91 [-7.06 to 8.88]	0.668
24-46: Moderate distress	55	42	3.68 [0.28 to 7.09]	
46-66: High distress	63	69	1.39 [-1.51 to 4.29]	
66+: Severe distress	34	37	3.79 [-0.11 to 7.70]	

[1] VR+TAU vs. TAU: Linear regression model for the primary outcome; modelled against treatment group, outcome score at baseline, stratification factors (site and service type) and an interaction between randomised group and the subgroup variable.

‡Level of significance = 0.05

TABLE 34 FURTHER RESULTS FOR THE SUBGROUP ANALYSIS ON SECONDARY OUTCOMES AT 26 WEEKS BY O-AS DISTRESS

	Group VR+TAU (N=174)	Group TAU (N=172)	Adjusted mean difference [95% CI][1]	Test of Interaction (P value)‡
**Agoraphobia Mobility Inventory-Avoidance [AMI-A]**				
≤23: Average distress	7	11	0.08 [-0.53 to 0.69]	0.058
24-46: Moderate distress	52	40	0.13 [-0.14 to 0.39]	
46-66: High distress	55	64	-0.17 [-0.41 to 0.06]	
66+: Severe distress	32	30	-0.42 [-0.74 to -0.11]	
**R-GPTS Part A**				
≤23: Average distress	7	10	2.81 [-4.47 to 10.09]	0.476
24-46: Moderate distress	47	39	0.57 [-2.67 to 3.81]	
46-66: High distress	50	57	-0.53 [-3.40 to 2.35]	
66+: Severe distress	29	28	-2.82 [-6.81 to 1.17]	
**R-GPTS Part B**				
≤23: Average distress	7	10	5.86 [-3.43 to 15.14]	0.142
24-46: Moderate distress	47	39	2.10 [-2.03 to 6.22]	
46-66: High distress	50	57	-1.34 [-4.99 to 2.31]	
66+: Severe distress	29	28	-4.07 [-9.14 to 0.99]	
**R-GPTS Overall total**				
≤23: Average distress	7	10	8.62 [-6.76 to 24.00]	0.186
24-46: Moderate distress	47	39	2.75 [-4.10 to 9.59]	
46-66: High distress	50	57	-1.94 [-8.00 to 4.12]	
66+: Severe distress	29	28	-7.18 [-15.60 to 1.23]	
**Paranoia worries questionnaire [PWQ]**				
≤23: Average distress	7	10	1.56 [-3.59 to 6.72]	0.122
24-46: Moderate distress	47	40	1.64 [-0.66 to 3.94]	
46-66: High distress	45	56	-2.01 [-4.13 to 0.11]	
66+: Severe distress	28	28	-0.64 [-3.42 to 2.15]	
**Patient health questionaire (Depression, anxiety and stress scale) [PHQ-9]**				
≤23: Average distress	7	10	2.67 [-2.99 to 8.32]	0.232
24-46: Moderate distress	49	39	1.67 [-0.80 to 4.15]	
46-66: High distress	50	58	-1.45 [-3.67 to 0.77]	
66+: Severe distress	28	30	0.54 [-2.49 to 3.56]	
**Suicidal Ideation**				
≤23: Average distress	5	11	-0.19 [-1.00 to 0.62]	0.921
24-46: Moderate distress	39	28	-0.11 [-0.48 to 0.27]	
46-66: High distress	44	53	0.04 [-0.27 to 0.34]	
66+: Severe distress	22	21	-0.04 [-0.51 to 0.43]	
**Quality of life**				
≤23: Average distress	7	10	0.12 [-0.09 to 0.33]	0.380
24-46: Moderate distress	51	41	-0.06 [-0.15 to 0.03]	
46-66: High distress	52	61	0.02 [-0.06 to 0.11]	
66+: Severe distress	32	33	-0.02 [-0.13 to 0.08]	
**The EQ5D VAS**				
≤23: Average distress	7	10	1.72 [-18.31 to 21.74]	0.899
24-46: Moderate distress	52	41	-2.75 [-11.10 to 5.60]	
46-66: High distress	54	62	1.50 [-5.95 to 8.95]	

	Group VR+TAU (N=174)	Group TAU (N=172)	Adjusted mean difference [95% CI][1]	Test of Interaction (P value)‡
66+: Severe distress	32	33	-0.49 [-10.26 to 9.28]	
**Recovering quality of life [REQOL-20]**				
≤23: Average distress	7	10	-7.84 [-20.15 to 4.46]	0.117
24-46: Moderate distress	49	39	-3.46 [-8.83 to 1.90]	
46-66: High distress	51	58	3.67 [-1.12 to 8.47]	
66+: Severe distress	29	30	2.52 [-3.95 to 9.00]	
**Questionnaire on progress of recovery [QPR]**				
≤23: Average distress	7	11	1.41 [-7.59 to 10.42]	0.291
24-46: Moderate distress	52	41	-1.41 [-5.31 to 2.48]	
46-66: High distress	58	66	3.66 [0.29 to 7.03]	
66+: Severe distress	31	33	1.90 [-2.75 to 6.55]	

[1] VR+TAU vs. TAU: Linear regression model for the primary outcome; modelled against treatment group, outcome score at baseline, stratification factors (site and service type) and an interaction between randomised group and the subgroup variable.

‡Level of significance = 0.05

4 References

Lambe, S. et al (2021). The Oxford Agoraphobic Avoidance Scale. Psychological Medicine 1–11. https://doi.org/10.1017/S0033291721002713

Freeman, Dunn et al (2015) Effects of cognitive behaviour therapy for worry on persecutory delusions in patients with psychosis (WIT). Lancet Psychiatry, 2, 305-313.

5 Appendices

5.1 Appendix I. O-BAT Summary Tables

TABLE 35: SUMMARY OF STEPS COMPLETED FOR THE REAL WORLD DISTRESS OUTCOME (O-BAT), BY RANDOMISED GROUP

	VR+TAU (N=174)			TAU (N=172)			Total (N=346)		
	**Baseline**	**6 weeks**	**26 weeks**	**Baseline**	**6 weeks**	**26 weeks**	**Baseline**	**6 weeks**	**26 weeks**
**Completed level 1, n (%)**	97 (55.7%)	55 (31.6%)	8 (4.6%)	92 (52.9%)	52 (29.9%)	8 (4.6%)	189 (108.6%)	107 (61.5%)	16 (9.2%)
**Distress level**									
Mean (SD)	4.7 (2.3)	2.7 (2.4)	1.9 (2.8)	4.8 (2.3)	3.5 (2.6)	2.1 (2.5)	4.8 (2.3)	3.1 (2.5)	2.0 (2.6)
Median (IQR)	5.0 (3.0 to 7.0)	2.0 (1.0 to 4.0)	0.5 (0.0 to 3.0)	5.0 (3.0 to 6.8)	3.0 (1.0 to 6.0)	0.8 (0.0 to 4.5)	5.0 (3.0 to 7.0)	2.0 (1.0 to 5.0)	0.8 (0.0 to 3.5)
Range	[0.0 to 10.0]	[0.0 to 10.0]	[0.0 to 8.0]	[0.0 to 10.0]	[0.0 to 9.0]	[0.0 to 6.0]	[0.0 to 10.0]	[0.0 to 10.0]	[0.0 to 8.0]
**Completed level 2, n (%)**	65 (37.4%)	48 (27.6%)	8 (4.6%)	59 (33.9%)	40 (23.0%)	8 (4.6%)	124 (71.3%)	88 (50.6%)	16 (9.2%)
**Distress level**									
Mean (SD)	5.8 (2.0)	3.5 (2.2)	3.3 (2.8)	5.6 (2.3)	3.9 (2.6)	3.0 (3.1)	5.7 (2.2)	3.6 (2.4)	3.1 (2.8)
Median (IQR)	6.0 (5.0 to 7.0)	3.0 (2.0 to 5.0)	4.0 (0.5 to 4.5)	6.0 (5.0 to 7.0)	4.0 (1.5 to 6.0)	2.5 (0.0 to 5.5)	6.0 (5.0 to 7.0)	3.5 (2.0 to 5.0)	3.5 (0.0 to 5.0)
Range	[1.0 to 10.0]	[0.0 to 9.0]	[0.0 to 8.0]	[0.0 to 10.0]	[0.0 to 9.0]	[0.0 to 8.0]	[0.0 to 10.0]	[0.0 to 9.0]	[0.0 to 8.0]

	VR+TAU (N=174)			TAU (N=172)			Total (N=346)		
	**Baseline**	**6 weeks**	**26 weeks**	**Baseline**	**6 weeks**	**26 weeks**	**Baseline**	**6 weeks**	**26 weeks**
**Completed level 3, n (%)**	36 (20.7%)	39 (22.4%)	7 (4.0%)	32 (18.4%)	27 (15.5%)	7 (4.0%)	68 (39.1%)	66 (37.9%)	14 (8.0%)
**Distress level**									
Mean (SD)	7.0 (1.8)	3.8 (2.6)	2.8 (2.5)	5.9 (2.5)	3.9 (2.8)	3.3 (3.0)	6.5 (2.2)	3.8 (2.7)	3.0 (2.6)
Median (IQR)	7.0 (6.0 to 8.0)	4.0 (2.0 to 5.0)	2.0 (0.5 to 5.0)	6.0 (5.0 to 8.0)	5.0 (2.0 to 6.0)	2.0 (0.0 to 6.0)	7.0 (5.0 to 8.0)	4.0 (2.0 to 5.0)	2.0 (0.5 to 6.0)
Range	[2.0 to 10.0]	[0.0 to 10.0]	[0.0 to 6.0]	[0.0 to 10.0]	[0.0 to 10.0]	[0.0 to 7.0]	[0.0 to 10.0]	[0.0 to 10.0]	[0.0 to 7.0]
**Completed level 4, n (%)**	18 (10.3%)	35 (20.1%)	6 (3.4%)	15 (8.6%)	20 (11.5%)	4 (2.3%)	33 (19.0%)	55 (31.6%)	10 (5.7%)
**Distress level**									
Mean (SD)	6.3 (2.7)	4.0 (2.6)	1.8 (1.2)	5.5 (2.4)	4.1 (2.6)	1.8 (1.5)	5.9 (2.6)	4.1 (2.6)	1.8 (1.2)
Median (IQR)	7.0 (4.0 to 8.0)	4.0 (2.0 to 5.5)	1.8 (1.0 to 3.0)	5.0 (3.0 to 8.0)	4.0 (2.0 to 6.5)	2.0 (0.5 to 3.0)	6.0 (4.0 to 8.0)	4.0 (2.0 to 6.0)	1.8 (1.0 to 3.0)
Range	[0.0 to 10.0]	[0.0 to 10.0]	[0.0 to 3.0]	[3.0 to 10.0]	[0.0 to 8.0]	[0.0 to 3.0]	[0.0 to 10.0]	[0.0 to 10.0]	[0.0 to 3.0]
**Completed level 5, n (%)**	9 (5.2%)	26 (14.9%)	6 (3.4%)	9 (5.2%)	10 (5.7%)	4 (2.3%)	18 (10.3%)	36 (20.7%)	10 (5.7%)
**Distress level**									

	VR+TAU (N=174)			TAU (N=172)			Total (N=346)		
	Baseline	6 weeks	26 weeks	Baseline	6 weeks	26 weeks	Baseline	6 weeks	26 weeks
Mean (SD)	5.6 (2.9)	4.6 (2.4)	3.0 (2.3)	5.3 (2.4)	3.8 (3.5)	2.5 (2.1)	5.4 (2.6)	4.3 (2.7)	2.8 (2.1)
Median (IQR)	5.0 (5.0 to 7.0)	4.5 (3.0 to 6.0)	2.3 (1.0 to 5.0)	5.0 (3.0 to 7.0)	3.3 (0.0 to 6.0)	2.5 (1.0 to 4.0)	5.0 (3.0 to 7.0)	4.0 (2.8 to 6.0)	2.5 (1.0 to 5.0)
Range	[1.0 to 10.0]	[0.0 to 9.0]	[1.0 to 6.5]	[2.0 to 9.5]	[0.0 to 10.0]	[0.0 to 5.0]	[1.0 to 10.0]	[0.0 to 10.0]	[0.0 to 6.5]
**Total Completed[1], n (%)**	225 (129.3%)	203 (116.7%)	35 (20.1%)	207 (119.0%)	149 (85.6%)	31 (17.8%)	432 (248.3%)	352 (202.3%)	66 (37.9%)
**Distress level**									
Mean (SD)	5.5 (2.3)	3.6 (2.5)	2.5 (2.4)	5.3 (2.4)	3.8 (2.7)	2.6 (2.5)	5.4 (2.4)	3.6 (2.6)	2.6 (2.4)
Median (IQR)	5.0 (4.0 to 7.0)	3.0 (2.0 to 5.0)	2.0 (0.5 to 4.0)	5.0 (4.0 to 7.0)	3.5 (1.5 to 6.0)	2.0 (0.0 to 5.0)	5.0 (4.0 to 7.0)	3.0 (2.0 to 5.3)	2.0 (0.0 to 5.0)
Range	[0.0 to 10.0]	[0.0 to 10.0]	[0.0 to 8.0]	[0.0 to 10.0]	[0.0 to 10.0]	[0.0 to 8.0]	[0.0 to 10.0]	[0.0 to 10.0]	[0.0 to 8.0]

[1] Summary of all levels completed by each participant and overall level of distress

TABLE 36: SUMMARY OF HIGHEST STEP COMPLETED FOR THE REAL WORLD DISTRESS OUTCOME (O-BAT), BY RANDOMISED GROUP

Highest step completed	VR+TAU (N=174)			TAU (N=172)			Total (N=346)		
	Baseline	6 weeks	26 weeks	Baseline	6 weeks	26 weeks	Baseline	6 weeks	26 weeks
**Completed level 1, n (%)**	32 (18.4%)	7 (4.0%)		33 (19.0%)	12 (6.9%)		65 (37.4%)	0	
**Distress level**									
Mean (SD)	5.3 (2.0)	4.6 (3.2)		6.0 (2.0)	5.6 (2.8)		5.7 (2.0)	-	
Median (IQR)	5.0 (3.3 to 7.0)	5.0 (2.0 to 7.0)		6.0 (4.0 to 7.0)	6.0 (4.0 to 7.5)		6.0 (4.0 to 7.0)	-	
Range	[2.0 to 9.0]	[1.0 to 10.0]		[3.0 to 10.0]	[0.0 to 9.0]		[2.0 to 10.0]	-	
**Completed level 2, n (%)**	28 (16.1%)	9 (5.2%)	1 (0.6%)	27 (15.5%)	12 (6.9%)	1 (0.6%)	55 (31.6%)	21 (12.1%)	2 (1.1%)
**Distress level**									
Mean (SD)	6.2 (2.0)	3.9 (2.1)	8.0 (.)	6.1 (2.5)	5.1 (1.8)	0.0 (.)	6.2 (2.2)	4.6 (2.0)	4.0 (5.7)
Median (IQR)	7.0 (5.0 to 8.0)	4.0 (3.0 to 4.0)	8.0 (8.0 to 8.0)	6.0 (5.0 to 8.0)	5.0 (3.5 to 6.0)	0.0 (0.0 to 0.0)	6.0 (5.0 to 8.0)	4.0 (3.0 to 6.0)	4.0 (0.0 to 8.0)
Range	[1.0 to 10.0]	[0.0 to 8.0]	[8.0 to 8.0]	[0.0 to 10.0]	[3.0 to 8.0]	[0.0 to 0.0]	[0.0 to 10.0]	[0.0 to 8.0]	[0.0 to 8.0]
**Completed level 3, n (%)**	18 (10.3%)	5 (2.9%)	1 (0.6%)	17 (9.8%)	8 (4.6%)	3 (1.7%)	35 (20.1%)	13 (7.5%)	4 (2.3%)

Highest step completed	VR+TAU (N=174)			TAU (N=172)			Total (N=346)		
	Baseline	6 weeks	26 weeks	Baseline	6 weeks	26 weeks	Baseline	6 weeks	26 weeks
**Distress level**									
Mean (SD)	7.7 (1.1)	5.8 (3.0)	5.0 (.)	6.5 (2.6)	5.5 (3.3)	6.3 (0.6)	7.1 (2.1)	5.6 (3.1)	6.0 (0.8)
Median (IQR)	7.0 (7.0 to 9.0)	4.0 (4.0 to 8.0)	5.0 (5.0 to 5.0)	7.0 (6.0 to 8.0)	5.5 (3.5 to 8.0)	6.0 (6.0 to 7.0)	7.0 (7.0 to 8.0)	5.0 (4.0 to 8.0)	6.0 (5.5 to 6.5)
Range	[6.0 to 10.0]	[3.0 to 10.0]	[5.0 to 5.0]	[0.0 to 9.0]	[0.0 to 10.0]	[6.0 to 7.0]	[0.0 to 10.0]	[0.0 to 10.0]	[5.0 to 7.0]
**Completed level 4, n (%)**	10 (5.7%)	9 (5.2%)		6 (3.4%)	10 (5.7%)		16 (9.2%)	0	
**Distress level**									
Mean (SD)	6.8 (2.2)	6.0 (2.5)		6.7 (2.7)	4.3 (2.6)		6.8 (2.3)	-	
Median (IQR)	7.3 (7.0 to 8.0)	5.0 (5.0 to 7.0)		6.5 (5.0 to 9.0)	4.0 (2.0 to 7.0)		7.3 (5.0 to 8.5)	-	
Range	[2.0 to 9.0]	[2.0 to 10.0]		[3.0 to 10.0]	[1.0 to 8.0]		[2.0 to 10.0]	-	
**Completed level 5, n (%)**	9 (5.2%)	26 (14.9%)	6 (3.4%)	9 (5.2%)	10 (5.7%)	4 (2.3%)	18 (10.3%)	36 (20.7%)	10 (5.7%)
**Distress level**									
Mean (SD)	5.6 (2.9)	4.6 (2.4)	3.0 (2.3)	5.3 (2.4)	3.8 (3.5)	2.5 (2.1)	5.4 (2.6)	4.3 (2.7)	2.8 (2.1)

Highest step completed	VR+TAU (N=174)			TAU (N=172)			Total (N=346)		
	Baseline	6 weeks	26 weeks	Baseline	6 weeks	26 weeks	Baseline	6 weeks	26 weeks
Median (IQR)	5.0 (5.0 to 7.0)	4.5 (3.0 to 6.0)	2.3 (1.0 to 5.0)	5.0 (3.0 to 7.0)	3.3 (0.0 to 6.0)	2.5 (1.0 to 4.0)	5.0 (3.0 to 7.0)	4.0 (2.8 to 6.0)	2.5 (1.0 to 5.0)
Range	[1.0 to 10.0]	[0.0 to 9.0]	[1.0 to 6.5]	[2.0 to 9.5]	[0.0 to 10.0]	[0.0 to 5.0]	[1.0 to 10.0]	[0.0 to 10.0]	[0.0 to 6.5]
**Total Completed[1], n (%)**	97 (55.7%)	56 (32.2%)	8 (4.6%)	92 (52.9%)	52 (29.9%)	8 (4.6%)	189 (108.6%)	108 (62.1%)	16 (9.2%)
**Distress level**									
Mean (SD)	6.2 (2.2)	4.8 (2.5)	3.9 (2.7)	6.1 (2.3)	4.9 (2.8)	3.6 (2.8)	6.1 (2.2)	4.8 (2.6)	3.8 (2.6)
Median (IQR)	7.0 (5.0 to 8.0)	4.5 (3.0 to 6.5)	4.0 (1.3 to 5.8)	6.0 (5.0 to 8.0)	5.0 (3.0 to 7.0)	4.0 (1.0 to 6.0)	7.0 (5.0 to 8.0)	5.0 (3.0 to 7.0)	4.0 (1.3 to 6.0)
Range	[1.0 to 10.0]	[0.0 to 10.0]	[1.0 to 8.0]	[0.0 to 10.0]	[0.0 to 10.0]	[0.0 to 7.0]	[0.0 to 10.0]	[0.0 to 10.0]	[0.0 to 8.0]

[1] Summary over all maximum levels completed by each participant and level of distress corresponding to these

5.2 Appendix II. Patient Health Questionnaire [PHQ-9] - categories

The PHQ-9 is a 9-item depression scale where each item is scored from 0-3. The total score is the sum of the individual item scores and can thus be a maximum of 27. Higher scores here indicate higher levels of depression. The continuous total score will be used for analysis while the following categories of depression, derived from the total score, will be summarised descriptively:

- 0-4 = Minimal depression
- 5-9 = Mild depression
- 10-14 = Moderate depression
- 15-19 = Moderately severe depression
- 20-27 = Severe depression

TABLE 37: CATEGORIES OF PHQ-9 TOTAL SCORE BY RANDOMISED GROUP

	VR+TAU (N=174)	TAU (N=172)	Total (N=346)
**Categories of PHQ-9 total score at baseline, n (%)**			
Minimal depression, n (%)	3 (1.7)	16 (9.3)	19 (5.5)
Mild depression, n (%)	33 (19.0)	30 (17.4)	63 (18.2)
Moderate depression, n (%)	42 (24.1)	34 (19.8)	76 (22.0)
Moderately severe depression, n (%)	40 (23.0)	46 (26.7)	86 (24.9)
Severe depression, n (%)	48 (27.6)	36 (20.9)	84 (24.3)
Missing, n (%)	8 (4.6)	10 (5.8)	18 (5.2)
**Categories of PHQ-9 total score at 6 weeks, n (%)**			
Minimal depression, n (%)	15 (8.6)	12 (7.0)	27 (7.8)
Mild depression, n (%)	36 (20.7)	50 (29.1)	86 (24.9)
Moderate depression, n (%)	41 (23.6)	28 (16.3)	69 (19.9)
Moderately severe depression, n (%)	33 (19.0)	46 (26.7)	79 (22.8)
Severe depression, n (%)	22 (12.6)	14 (8.1)	36 (10.4)
Missing, n (%)	27 (15.5)	22 (12.8)	49 (14.2)
**Categories of PHQ-9 total score at 26 weeks, n (%)**			
Minimal depression, n (%)	17 (9.8)	24 (14.0)	41 (11.8)
Mild depression, n (%)	30 (17.2)	28 (16.3)	58 (16.8)

	VR+TAU (N=174)	TAU (N=172)	Total (N=346)
Moderate depression, n (%)	38 (21.8)	37 (21.5)	75 (21.7)
Moderately severe depression, n (%)	23 (13.2)	31 (18.0)	54 (15.6)
Severe depression, n (%)	26 (14.9)	17 (9.9)	43 (12.4)
Missing, n (%)	40 (23.0)	35 (20.3)	75 (21.7)

5.3 Appendix III. C-SSRS Summary Tables

The Intensity of Ideation subscale is comprised of five items (i.e., Frequency [CSSRS5_FRQ], Duration [CSSRS5_ DUR], Controllability [CSSRS5_ CON], deterrents [CSSRS5_ DET] & Reason for ideation [CSSRS5_ REAS]) each rated on a scale from 1 to 5 (total scores ranging from 1 to 25) and relates to the response in the Suicidal Ideation section. The total scores at each time point are summarised by treatment group, together with the Suicidal Behaviour [CSSRS6- CSSRS8] and Preparatory acts or behaviour [CSSRS9- CSSRS10] scales in Table 38.

TABLE 38: SUMMARY STATISTICS FOR THE C-SSRS INTENSITY OF IDEATION, SUICIDAL BEHAVIOUR AND PREPARATORY ACTS OR BEHAVIOUR SCALES, BY RANDOMISED GROUPS

C-SSRS scales	VR+TAU (N=174)	TAU (N=172)
**Baseline**		
**Intensity of Ideation total score, mean (SD) [n]**	13.3 (3.1) [80]	12.7 (4.6) [75]
**Suicidal Behaviour - Actual attempt, n (%)**	-	2 (0.4)
**Suicidal Behaviour - Interrupted attempt, n (%)**	-	-
**Suicidal Behaviour - Aborted or self-interrupted attempt, n (%)**	1 (0.2)	3 (0.6)
**Preparatory acts or behaviour, n (%)**	-	1 (0.2)
**6 weeks**		
**Intensity of Ideation total score, mean (SD) [n]**	12.7 (4.5) [57]	12.5 (3.8) [53]
**Suicidal Behaviour - Actual attempt, n (%)**	-	2 (0.4)
**Suicidal Behaviour - Interrupted attempt, n (%)**	-	-
**Suicidal Behaviour - Aborted or self-interrupted attempt, n (%)**	1 (0.2)	3 (0.6)

**C-SSRS scales**	**VR+TAU (N=174)**	**TAU (N=172)**
**Preparatory acts or behaviour, n (%)**	-	1 (0.2)
**26 weeks**		
**Intensity of Ideation total score, mean (SD) [n]**	12.0 (4.1) [41]	11.9 (4.2) [39]
**Suicidal Behaviour - Actual attempt, n (%)**	-	2 (0.4)
**Suicidal Behaviour - Interrupted attempt, n (%)**	-	-
**Suicidal Behaviour - Aborted or self-interrupted attempt, n (%)**	1 (0.2)	3 (0.6)
**Preparatory acts or behaviour, n (%)**	-	1 (0.2)
**Suicide, n (%)**	-	-

5.4 Appendix IV. Recovering quality of life - physical health

Question 21 *[REQOL21]* refers to physical health only and was analysed descriptively.

TABLE 39: CATEGORIES OF REQOL-21 BY RANDOMISED GROUP

**REQOL-21 at:**	**VR+TAU (N=174)**	**TAU (N=172)**	**Total (N=346)**
**Baseline, n (%)**			
No problems, n (%)	50 (28.7)	49 (28.5)	99 (28.6)
Slight problems, n (%)	48 (27.6)	46 (26.7)	94 (27.2)
Moderate problems, n (%)	45 (25.9)	43 (25.0)	88 (25.4)
Severe problems, n (%)	20 (11.5)	25 (14.5)	45 (13.0)
Very severe problems, n (%)	3 (1.7)	-	3 (0.9)
Missing, n (%)	8 (4.6)	9 (5.2)	17 (4.9)
**6 weeks, n (%)**			

REQOL-21 at:	VR+TAU (N=174)	TAU (N=172)	Total (N=346)
No problems, n (%)	55 (31.6)	49 (28.5)	104 (30.1)
Slight problems, n (%)	44 (25.3)	39 (22.7)	83 (24.0)
Moderate problems, n (%)	33 (19.0)	42 (24.4)	75 (21.7)
Severe problems, n (%)	14 (8.0)	17 (9.9)	31 (9.0)
Very severe problems, n (%)	2 (1.1)	2 (1.2)	4 (1.2)
Missing, n (%)	26 (14.9)	23 (13.4)	49 (14.2)
**26 weeks, n (%)**			
No problems, n (%)	43 (24.7)	51 (29.7)	94 (27.2)
Slight problems, n (%)	41 (23.6)	36 (20.9)	77 (22.3)
Moderate problems, n (%)	34 (19.5)	36 (20.9)	70 (20.2)
Severe problems, n (%)	12 (6.9)	12 (7.0)	24 (6.9)
Very severe problems, n (%)	5 (2.9)	1 (0.6)	6 (1.7)
Missing, n (%)	39 (22.4)	36 (20.9)	75 (21.7)

5.5 Appendix V. Missingness Investigation

In the tables below, baseline characteristics are summarised for those participants with a missing primary outcome. Frequencies and percentages for the binary/categorical, and summary statistics for the continuous baseline characteristics are presented; split by randomised group and if the participants had the primary outcome or not. This is done separately for the two parts of the O-AS. Associated P-values for predicting missingness of the primary outcome (randomised groups combined) obtained from individual logistic regressions for each baseline characteristic are also presented. Missingness by treatment group is presented for information only, no statistical comparisons of missingness by treatment group were undertaken. The logistic regression models consider data from the all randomised participants.

5.5.1 O-AS Avoidance Score

Separate logistic regression models were fitted for each baseline covariate to obtain the P-value for the association of missingness with primary outcome for the overall trial population. Only baseline time budget was found to be predictive of missingness (P = 0. 05).

TABLE 40: BASELINE CHARACTERISTICS OF PARTICIPANTS BY COMPLETENESS FOR O-AS AVOIDANCE AT 6 WEEKS

Baseline Characteristic	Predictive of missingness (P Value)[1]	VR+TAU (N=174)		TAU (N=172)	
		Missing (n=14)	Not missing (n=160)	Missing (n=12)	Not missing (n=160)
**Age (years)**	0.065				
Mean (SD)		31.2 (7.1)	37.1 (13.1)	34.7 (10.3)	38.1 (12.3)
Median (IQR)		31.8 (23.9 to 37.4)	35.6 (26.0 to 47.4)	33.7 (29.9 to 37.2)	36.9 (27.8 to 47.1)
Min to Max		22.7 to 40.8	17.1 to 69.1	19.0 to 61.2	16.6 to 70.7
Missing		-	-	-	-
**Age at first contact with mental health services (years)**	0.692				
Mean (SD)		25.8 (5.8)	24.6 (10.0)	23.3 (8.6)	26.4 (11.2)
Median (IQR)		25.0 (21.0 to 29.0)	22.0 (18.0 to 28.0)	21.5 (18.5 to 28.0)	23.0 (18.0 to 32.0)
Min to Max		18.0 to 35.0	6.0 to 59.0	7.0 to 38.0	7.0 to 60.0
Missing		1	2	-	7
**Sex[2], n (%)**	0.063				
Female		1/14 (7.1)	57/160 (35.6)	3/12 (25.0)	50/160 (31.3)

Baseline Characteristic	Predictive of missingness (P Value)[1]	VR+TAU (N=174)		TAU (N=172)	
		Missing (n=14)	Not missing (n=160)	Missing (n=12)	Not missing (n=160)
Male		13/14 (92.9)	103/160 (64.4)	9/12 (75.0)	106/160 (66.3)
Missing		-	-	-	4/160 (2.5)
**Current Marital status, n (%)**	0.226				
Single		11/14 (78.6)	120/160 (75.0)	11/12 (91.7)	127/160 (79.4)
Married/civil partnership		1/14 (7.1)	20/160 (12.5)	-	14/160 (8.8)
Cohabiting		1/14 (7.1)	5/160 (3.1)	1/12 (8.3)	9/160 (5.6)
Separated		-	2/160 (1.3)	-	1/160 (0.6)
Divorced		-	9/160 (5.6)	-	7/160 (4.4)
Widowed		-	3/160 (1.9)	-	1/160 (0.6)
Missing		1/14 (7.1)	1/160 (0.6)	-	1/160 (0.6)
**Ethnic group, n (%)**	0.971				
White		12/14 (85.7)	140/160 (87.5)	10/12 (83.3)	132/160 (82.5)
Black British		-	1/160 (0.6)	-	1/160 (0.6)
Black African		-	1/160 (0.6)	-	2/160 (1.3)
Black Caribbean		-	-	-	4/160 (2.5)
Indian		-	-	1/12 (8.3)	1/160 (0.6)
Black Other		-	1/160 (0.6)	-	-
Pakistani		2/14 (14.3)	1/160 (0.6)	-	3/160 (1.9)
Other		-	16/160 (10.0)	1/12 (8.3)	16/160 (10.0)
Missing		-	-	-	1/160 (0.6)
**Site, n (%)**	0.860				
Bristol		5/14 (35.7)	32/160 (20.0)	-	37/160 (23.1)
Manchester		3/14 (21.4)	26/160 (16.3)	4/12 (33.3)	25/160 (15.6)
Newcastle		-	42/160 (26.3)	3/12 (25.0)	36/160 (22.5)
Nottingham		2/14 (14.3)	30/160 (18.8)	2/12 (16.7)	29/160 (18.1)
Oxford		4/14 (28.6)	30/160 (18.8)	3/12 (25.0)	33/160 (20.6)

Baseline Characteristic	Predictive of missingness (P Value)[1]	VR+TAU (N=174)		TAU (N=172)	
		Missing (n=14)	Not missing (n=160)	Missing (n=12)	Not missing (n=160)
**Service type, n (%)**	0.085				
Community MH team		6/14 (42.9)	101/160 (63.1)	5/12 (41.7)	97/160 (60.6)
Early intervention		8/14 (57.1)	56/160 (35.0)	7/12 (58.3)	62/160 (38.8)
In-patient		-	3/160 (1.9)	-	1/160 (0.6)
**Employment, n (%)**	0.995				
Employed full-time (paid), n (%)		-	10/160 (6.3)	-	9/160 (5.6)
Employed part-time (paid), n (%)		-	4/160 (2.5)	-	4/160 (2.5)
Employed full-time (voluntary), n (%)		-	-	-	-
Employed part-time (voluntary), n (%)		1/14 (7.1)	1/160 (0.6)	-	3/160 (1.9)
Unemployed (on benefits), n (%)		11/14 (78.6)	101/160 (63.1)	10/12 (83.3)	112/160 (70.0)
Unemployed (not on benefits), n (%)		-	8/160 (5.0)	-	5/160 (3.1)
Student or in training full-time, n (%)		2/14 (14.3)	3/160 (1.9)	1/12 (8.3)	5/160 (3.1)
Student or in training part-time, n (%)		-	3/160 (1.9)	-	1/160 (0.6)
Self-employed, n (%)		-	4/160 (2.5)	-	1/160 (0.6)
Home-maker, n (%)		-	2/160 (1.3)	-	1/160 (0.6)
Carer, n (%)		-	1/160 (0.6)	-	1/160 (0.6)
Retired, n (%)		-	5/160 (3.1)	-	2/160 (1.3)
Other, n (%)		-	-	-	3/160 (1.9)
**Usual/Normal living arrangement, n (%)**	0.831	-	18/160 (11.3)	1/12 (8.3)	13/160 (8.1)
Living alone (+/- children), n (%)		7/14 (50.0)	65/160 (40.6)	6/12 (50.0)	66/160 (41.3)

Baseline Characteristic	Predictive of missingness (P Value)[1]	VR+TAU (N=174)		TAU (N=172)	
		Missing (n=14)	Not missing (n=160)	Missing (n=12)	Not missing (n=160)
Living with husband/wife, n (%)		1/14 (7.1)	15/160 (9.4)	-	13/160 (8.1)
Living with partner, n (%)		-	8/160 (5.0)	-	9/160 (5.6)
Living with parents, n (%)		4/14 (28.6)	36/160 (22.5)	3/12 (25.0)	39/160 (24.4)
Living with other relatives, n (%)		1/14 (7.1)	8/160 (5.0)	-	10/160 (6.3)
Living with others (e.g. friends), n (%)		1/14 (7.1)	9/160 (5.6)	2/12 (16.7)	9/160 (5.6)
Missing, n (%)		-	19/160 (11.9)	1/12 (8.3)	14/160 (8.8)
**Mental health diagnosis (F-code), n (%)**	0.658				
20		3/14 (21.4)	29/160 (18.1)	3/12 (25.0)	24/160 (15.0)
20.0		1/14 (7.1)	24/160 (15.0)	1/12 (8.3)	28/160 (17.5)
20.5		-	-	-	1/160 (0.6)
20.8		-	-	1/12 (8.3)	-
20.9		1/14 (7.1)	16/160 (10.0)	-	6/160 (3.8)
21		-	1/160 (0.6)	-	2/160 (1.3)
22		-	1/160 (0.6)	-	2/160 (1.3)
22.8		-	1/160 (0.6)	-	-
23		-	-	-	2/160 (1.3)
23.1		-	2/160 (1.3)	-	2/160 (1.3)
23.2		-	-	-	1/160 (0.6)
23.9		-	3/160 (1.9)	-	4/160 (2.5)
25		-	3/160 (1.9)	-	2/160 (1.3)
25.0		-	2/160 (1.3)	-	-
25.1		-	1/160 (0.6)	-	2/160 (1.3)
25.9		-	9/160 (5.6)	-	7/160 (4.4)

Baseline Characteristic	Predictive of missingness (P Value)[1]	VR+TAU (N=174)		TAU (N=172)	
		Missing (n=14)	Not missing (n=160)	Missing (n=12)	Not missing (n=160)
28		-	2/160 (1.3)	-	3/160 (1.9)
29		7/14 (50.0)	50/160 (31.3)	6/12 (50.0)	48/160 (30.0)
29.0		1/14 (7.1)	4/160 (2.5)	1/12 (8.3)	5/160 (3.1)
31.2		-	1/160 (0.6)	-	4/160 (2.5)
31.4		-	-	-	1/160 (0.6)
31.5		-	2/160 (1.3)	-	-
32.3		1/14 (7.1)	6/160 (3.8)	-	13/160 (8.1)
33.3		-	3/160 (1.9)	-	3/160 (1.9)
**Currently taking any medication, n (%)**	0.841				
Yes		14/14 (100.0)	155/160 (96.9)	11/12 (91.7)	155/160 (96.9)
No		-	5/160 (3.1)	1/12 (8.3)	5/160 (3.1)
**Type of medications in use[3]**					
**Antipsychotic**	0.420				
Yes, n (%)		14/14 (100.0)	147/160 (91.9)	11/12 (91.7)	145/160 (90.6)
No, n (%)		-	12/160 (7.5)	-	15/160 (9.4)
Missing, n (%)		-	1/160 (0.6)	-	0/160 (0.0)
**Antidepressant**	0.220				
Yes, n (%)		10/14 (71.4)	93/160 (58.1)	8/12 (66.7)	88/160 (55.0)
No, n (%)		4/14 (28.6)	66/160 (41.3)	4/12 (33.3)	72/160 (45.0)
Missing, n (%)		-	1/160 (0.6)	-	0/160 (0.0)
**Anxiolytic**	0.418				
Yes, n (%)		-	15/160 (9.4)	-	12/160 (7.5)
No, n (%)		14/14 (100.0)	143/160 (89.4)	11/12 (91.7)	148/160 (92.5)
Missing, n (%)		-	2/160 (1.3)	-	0/160 (0.0)
**Mood stabiliser**	-				

Baseline Characteristic	Predictive of missingness (P Value)[1]	VR+TAU (N=174)		TAU (N=172)	
		Missing (n=14)	Not missing (n=160)	Missing (n=12)	Not missing (n=160)
Yes, n (%)		-	18/160 (11.3)	-	15/160 (9.4)
No, n (%)		14/14 (100.0)	140/160 (87.5)	12/12 (100.0)	145/160 (90.6)
Missing, n (%)		-	2/160 (1.3)	-	0/160 (0.0)
**Hypnotic**	0.742				
Yes, n (%)		1/14 (7.1)	10/160 (6.3)	0/12 (0.0)	7/160 (4.4)
No, n (%)		13/14 (92.9)	148/160 (92.5)	12/12 (100.0)	153/160 (95.6)
Missing, n (%)		-	2/160 (1.3)	-	0/160 (0.0)
**Stimulant**	-				
Yes, n (%)		-	1/160 (0.6)	-	0/160 (0.0)
No, n (%)		14/14 (100.0)	157/160 (98.1)	12/12 (100.0)	160/160 (100.0)
**Primary & Secondary outcomes at baseline**					
**O-AS Avoidance score, mean (SD) [n]**	0.444	3.6 (2.4) [14]	3.1 (2.5) [159]	3.7 (2.5) [12]	3.4 (2.7) [160]
**O-AS Distress score, mean (SD) [n]**	0.327	51.1 (17.7) [14]	51.4 (16.4) [160]	59.7 (11.2) [12]	52.1 (17.5) [160]
**(Actigraphy) Mean number of steps, mean (SD) [n]**	0.476	3568.5 (1711.4) [5]	4791.7 (3065.9) [90]	8006.2 (2770.2) [4]	4798.8 (3061.5) [85]
**Time budget score, mean (SD) [n]**	0.050	43.3 (15.9) [12]	52.6 (17.4) [139]	48.3 (21.8) [8]	53.5 (16.5) [134]
**Agoraphobia Mobility Inventory-Avoidance (AMI-A) score, mean (SD) [n]**	0.176	3.4 (0.6) [14]	3.3 (0.7) [153]	3.5 (0.4) [10]	3.2 (0.8) [154]
**O-BAT - maximum number of steps completed, mean (SD) [n]**	0.232	2.5 (0.6) [4]	2.7 (1.3) [94]	3.4 (0.7) [9]	2.7 (1.3) [84]

Baseline Characteristic	Predictive of missingness (P Value)[1]	VR+TAU (N=174)		TAU (N=172)	
		Missing (n=14)	Not missing (n=160)	Missing (n=12)	Not missing (n=160)
O-BAT Mean distress score of completed steps, mean (SD) [n]	0.927	5.0 (2.1) [4]	5.5 (1.9) [93]	5.8 (2.0) [9]	5.5 (2.1) [83]
R-GPTS-A (social reference) score, mean (SD) [n]	0.213	15.8 (10.4) [12]	13.9 (9.2) [146]	15.4 (10.1) [11]	12.3 (9.0) [150]
R-GPTS-B (persecution) score, mean (SD) [n]	0.102	19.6 (13.6) [12]	17.1 (12.6) [146]	20.4 (13.5) [11]	13.7 (12.8) [150]
R-GPTS (overall) score, mean (SD) [n]	0.119	35.4 (22.6) [12]	31.0 (20.6) [146]	35.7 (23.4) [11]	26.0 (20.5) [150]
Paranoia Worries Questionnaire (PWQ) total score, mean (SD) [n]	0.084	10.8 (6.6) [12]	9.8 (6.2) [146]	12.9 (3.3) [9]	8.7 (6.2) [147]
Patient Health Questionnaire (PHQ-9) total score, mean (SD) [n]	0.748	14.5 (5.8) [13]	15.2 (6.1) [153]	15.6 (6.5) [11]	14.0 (6.5) [151]
Columbia Suicide Severity Rating Scale (C-SSRS) total score, mean (SD) [n]	0.990	0.6 (0.9) [14]	1.1 (1.3) [141]	1.6 (1.8) [9]	0.9 (1.2) [145]
EQ-5D-5L INDEX, mean (SD) [n]	0.388	0.5 (0.3) [13]	0.5 (0.3) [159]	0.5 (0.3) [12]	0.6 (0.3) [158]
EQ5D VAS score, mean (SD) [n]	0.408	48.1 (15.2) [13]	51.9 (19.5) [158]	50.7 (20.4) [12]	53.4 (19.1) [158]
Recovering Quality Of Life (REQOL-20) total score, mean (SD) [n]	0.343	35.4 (15.3) [14]	33.4 (13.1) [152]	28.4 (10.9) [12]	36.1 (13.2) [151]
Progress of Recovery (QPR) total score, mean (SD) [n]	0.964	31.2 (9.4) [14]	26.8 (10.8) [159]	22.8 (9.2) [11]	28.4 (11.2) [159]

[1] P value obtained from a logistic regression of missingness of primary outcome against each of the baseline characteristics (randomised groups combined). Level of significance = 0.05
[2] Categories with small numbers treated as missing data
[3] Not mutually exclusive

5.5.2 O-AS Distress Score

Separate logistic regression models were fitted for each baseline covariate to obtain the P-value for the association of missingness with primary outcome for the overall trial population. Only baseline time budget was found to be predictive of missingness (P = 0. 043).

TABLE 41: BASELINE CHARACTERISTICS OF PARTICIPANTS BY COMPLETENESS FOR O-AS DISTRESS AT 6 WEEKS

Baseline Characteristic	Predictive of missingness (P Value)[1]	VR+TAU (N=174)		TAU (N=172)	
		Missing (n=14)	Not missing (n=160)	Missing (n=10)	Not missing (n=162)
**Age (years)**	0.051				
Mean (SD)		31.2 (7.1)	37.1 (13.1)	33.9 (11.1)	38.1 (12.2)
Median (IQR)		31.8 (23.9 to 37.4)	35.6 (26.0 to 47.4)	32.3 (28.2 to 35.8)	36.9 (27.9 to 47.0)
Min to Max		22.7 to 40.8	17.1 to 69.1	19.0 to 61.2	16.6 to 70.7
Missing		-	-	-	-
**Age at first contact with mental health services (years)**	0.384				
Mean (SD)		25.8 (5.8)	24.6 (10.0)	20.6 (6.5)	26.5 (11.2)
Median (IQR)		25.0 (21.0 to 29.0)	22.0 (18.0 to 28.0)	21.0 (18.0 to 27.0)	23.0 (18.0 to 34.0)
Min to Max		18.0 to 35.0	6.0 to 59.0	7.0 to 29.0	7.0 to 60.0
Missing		1	2	-	7
**Sex[2], n (%)**	0.097				
Female		1/14 (7.1)	57/160 (35.6)	3/10 (30.0)	50/162 (30.9)
Male		13/14 (92.9)	103/160 (64.4)	7/10 (70.0)	108/162 (66.7)
Missing		-	-	-	4/162 (2.5)
**Current Marital status, n (%)**	0.275				
Single		11/14 (78.6)	120/160 (75.0)	9/10 (90.0)	129/162 (79.6)
Married/civil partnership		1/14 (7.1)	20/160 (12.5)	-	14/162 (8.6)
Cohabiting		1/14 (7.1)	5/160 (3.1)	1/10 (10.0)	9/162 (5.6)
Separated		-	2/160 (1.3)	-	1/162 (0.6)

Baseline Characteristic	Predictive of missingness (P Value)[1]	VR+TAU (N=174)		TAU (N=172)	
		Missing (n=14)	Not missing (n=160)	Missing (n=10)	Not missing (n=162)
Divorced		-	9/160 (5.6)	-	7/162 (4.3)
Widowed		-	3/160 (1.9)	-	1/162 (0.6)
Missing		1/14 (7.1)	1/160 (0.6)	-	1/162 (0.6)
**Ethnic group, n (%)**	0.640				
White		12/14 (85.7)	140/160 (87.5)	9/10 (90.0)	133/162 (82.1)
Black British		-	1/160 (0.6)	-	1/162 (0.6)
Black African		-	1/160 (0.6)	-	2/162 (1.2)
Black Caribbean		-	-	-	4/162 (2.5)
Indian		-	-	1/10 (10.0)	1/162 (0.6)
Black Other		-	1/160 (0.6)	-	-
Pakistani		2/14 (14.3)	1/160 (0.6)	-	3/162 (1.9)
Other		-	16/160 (10.0)	-	17/162 (10.5)
Missing		-	-	-	1/162 (0.6)
**Site, n (%)**	0.742				
Bristol		5/14 (35.7)	32/160 (20.0)	-	37/162 (22.8)
Manchester		3/14 (21.4)	26/160 (16.3)	3/10 (30.0)	26/162 (16.0)
Newcastle		-	42/160 (26.3)	2/10 (20.0)	37/162 (22.8)
Nottingham		2/14 (14.3)	30/160 (18.8)	2/10 (20.0)	29/162 (17.9)
Oxford		4/14 (28.6)	30/160 (18.8)	3/10 (30.0)	33/162 (20.4)
**Service type, n (%)**	0.087				
Community MH team		6/14 (42.9)	101/160 (63.1)	4/10 (40.0)	98/162 (60.5)
Early intervention		8/14 (57.1)	56/160 (35.0)	6/10 (60.0)	63/162 (38.9)
In-patient		-	3/160 (1.9)	-	1/162 (0.6)
**Employment, n (%)**	0.960				
Employed full-time (paid), n (%)		-	10/160 (6.3)	-	9/162 (5.6)

Baseline Characteristic	Predictive of missingness (P Value)[1]	VR+TAU (N=174)		TAU (N=172)	
		Missing (n=14)	Not missing (n=160)	Missing (n=10)	Not missing (n=162)
Employed part-time (paid), n (%)		-	4/160 (2.5)	-	4/162 (2.5)
Employed full-time (voluntary), n (%)		-	-	-	-
Employed part-time (voluntary), n (%)		1/14 (7.1)	1/160 (0.6)	-	3/162 (1.9)
Unemployed (on benefits), n (%)		11/14 (78.6)	101/160 (63.1)	8/10 (80.0)	114/162 (70.4)
Unemployed (not on benefits), n (%)		-	8/160 (5.0)	-	5/162 (3.1)
Student or in training full-time, n (%)		2/14 (14.3)	3/160 (1.9)	1/10 (10.0)	5/162 (3.1)
Student or in training part-time, n (%)		-	3/160 (1.9)	-	1/162 (0.6)
Self-employed, n (%)		-	4/160 (2.5)	-	1/162 (0.6)
Home-maker, n (%)		-	2/160 (1.3)	-	1/162 (0.6)
Carer, n (%)		-	1/160 (0.6)	-	1/162 (0.6)
Retired, n (%)		-	5/160 (3.1)	-	2/162 (1.2)
Other, n (%)		-	-	-	3/162 (1.9)
**Usual/Normal living arrangement, n (%)**	0.988	-	18/160 (11.3)	1/10 (10.0)	13/162 (8.0)
Living alone (+/- children), n (%)		7/14 (50.0)	65/160 (40.6)	5/10 (50.0)	67/162 (41.4)
Living with husband/wife, n (%)		1/14 (7.1)	15/160 (9.4)	-	13/162 (8.0)
Living with partner, n (%)		-	8/160 (5.0)	-	9/162 (5.6)
Living with parents, n (%)		4/14 (28.6)	36/160 (22.5)	3/10 (30.0)	39/162 (24.1)
Living with other relatives, n (%)		1/14 (7.1)	8/160 (5.0)	-	10/162 (6.2)

Baseline Characteristic	Predictive of missingness (P Value)[1]	VR+TAU (N=174)		TAU (N=172)	
		Missing (n=14)	Not missing (n=160)	Missing (n=10)	Not missing (n=162)
Living with others (e.g. friends), n (%)		1/14 (7.1)	9/160 (5.6)	1/10 (10.0)	10/162 (6.2)
Missing, n (%)		-	19/160 (11.9)	1/10 (10.0)	14/162 (8.6)
**Mental health diagnosis (F-code), n (%)**	0.577				
20		3/14 (21.4)	29/160 (18.1)	2/10 (20.0)	25/162 (15.4)
20.0		1/14 (7.1)	24/160 (15.0)	1/10 (10.0)	28/162 (17.3)
20.5		-	-	-	1/162 (0.6)
20.8		-	-	1/10 (10.0)	-
20.9		1/14 (7.1)	16/160 (10.0)	-	6/162 (3.7)
21		-	1/160 (0.6)	-	2/162 (1.2)
22		-	1/160 (0.6)	-	2/162 (1.2)
22.8		-	1/160 (0.6)	-	-
23		-	-	-	2/162 (1.2)
23.1		-	2/160 (1.3)	-	2/162 (1.2)
23.2		-	-	-	1/162 (0.6)
23.9		-	3/160 (1.9)	-	4/162 (2.5)
25		-	3/160 (1.9)	-	2/162 (1.2)
25.0		-	2/160 (1.3)	-	-
25.1		-	1/160 (0.6)	-	2/162 (1.2)
25.9		-	9/160 (5.6)	-	7/162 (4.3)
28		-	2/160 (1.3)	-	3/162 (1.9)
29		7/14 (50.0)	50/160 (31.3)	5/10 (50.0)	49/162 (30.2)
29.0		1/14 (7.1)	4/160 (2.5)	1/10 (10.0)	5/162 (3.1)
31.2		-	1/160 (0.6)	-	4/162 (2.5)
31.4		-	-	-	1/162 (0.6)
31.5		-	2/160 (1.3)	-	-

Baseline Characteristic	Predictive of missingness (P Value)[1]	VR+TAU (N=174)		TAU (N=172)	
		Missing (n=14)	Not missing (n=160)	Missing (n=10)	Not missing (n=162)
32.3		1/14 (7.1)	6/160 (3.8)	-	13/162 (8.0)
33.3		-	3/160 (1.9)	-	3/162 (1.9)
**Currently taking any medication, n (%)**	0.776				
Yes		14/14 (100.0)	155/160 (96.9)	9/10 (90.0)	157/162 (96.9)
No		-	5/160 (3.1)	1/10 (10.0)	5/162 (3.1)
**Type of medications in use[3]**					
**Antipsychotic**	0.473				
Yes, n (%)		14/14 (100.0)	147/160 (91.9)	9/10 (90.0)	147/162 (90.7)
No, n (%)		-	12/160 (7.5)	-	15/162 (9.3)
Missing, n (%)		-	1/160 (0.6)	-	0/162 (0.0)
**Antidepressant**	0.358				
Yes, n (%)		10/14 (71.4)	93/160 (58.1)	6/10 (60.0)	90/162 (55.6)
No, n (%)		4/14 (28.6)	66/160 (41.3)	4/10 (40.0)	72/162 (44.4)
Missing, n (%)		-	1/160 (0.6)	-	0/162 (0.0)
**Anxiolytic**	0.471				
Yes, n (%)		-	15/160 (9.4)	-	12/162 (7.4)
No, n (%)		14/14 (100.0)	143/160 (89.4)	9/10 (90.0)	150/162 (92.6)
Missing, n (%)		-	2/160 (1.3)	-	0/162 (0.0)
**Mood stabiliser**	-				
Yes, n (%)		-	18/160 (11.3)	-	15/162 (9.3)
No, n (%)		14/14 (100.0)	140/160 (87.5)	10/10 (100.0)	147/162 (90.7)
Missing, n (%)		-	2/160 (1.3)	-	0/162 (0.0)
**Hypnotic**	0.808				
Yes, n (%)		1/14 (7.1)	10/160 (6.3)	0/10 (0.0)	7/162 (4.3)
No, n (%)		13/14 (92.9)	148/160 (92.5)	10/10 (100.0)	155/162 (95.7)

Baseline Characteristic	Predictive of missingness (P Value)[1]	VR+TAU (N=174)		TAU (N=172)	
		Missing (n=14)	Not missing (n=160)	Missing (n=10)	Not missing (n=162)
Missing, n (%)		-	2/160 (1.3)	-	0/162 (0.0)
**Stimulant**	-				
Yes, n (%)		-	1/160 (0.6)	-	0/162 (0.0)
No, n (%)		14/14 (100.0)	157/160 (98.1)	10/10 (100.0)	162/162 (100.0)
**Primary & Secondary outcomes at baseline**					
**O-AS Avoidance score, mean (SD) [n]**	0.787	3.6 (2.4) [14]	3.1 (2.5) [159]	3.1 (2.3) [10]	3.4 (2.7) [162]
**O-AS Distress score, mean (SD) [n]**	0.580	51.1 (17.7) [14]	51.4 (16.4) [160]	57.5 (10.7) [10]	52.3 (17.5) [162]
**(Actigraphy) Mean number of steps, mean (SD) [n]**	0.476	3568.5 (1711.4) [5]	4791.7 (3065.9) [90]	8006.2 (2770.2) [4]	4798.8 (3061.5) [85]
**Time budget score, mean (SD) [n]**	0.043	43.3 (15.9) [12]	52.6 (17.4) [139]	47.4 (23.4) [7]	53.5 (16.4) [135]
**Agoraphobia Mobility Inventory-Avoidance (AMI-A) score, mean (SD) [n]**	0.201	3.4 (0.6) [14]	3.3 (0.7) [153]	3.5 (0.5) [9]	3.2 (0.8) [155]
**O-BAT - maximum number of steps completed, mean (SD) [n]**	0.487	2.5 (0.6) [4]	2.7 (1.3) [94]	3.3 (0.8) [7]	2.7 (1.3) [86]
**O-BAT Mean distress score of completed steps, mean (SD) [n]**	0.951	5.0 (2.1) [4]	5.5 (1.9) [93]	5.7 (2.2) [7]	5.5 (2.1) [85]
**R-GPTS-A (social reference) score, mean (SD) [n]**	0.343	15.8 (10.4) [12]	13.9 (9.2) [146]	14.2 (11.0) [9]	12.5 (9.0) [152]
**R-GPTS-B (persecution) score, mean (SD) [n]**	0.246	19.6 (13.6) [12]	17.1 (12.6) [146]	17.9 (13.8) [9]	13.9 (12.8) [152]
**R-GPTS (overall) score, mean (SD) [n]**	0.256	35.4 (22.6) [12]	31.0 (20.6) [146]	32.1 (24.6) [9]	26.4 (20.6) [152]

Baseline Characteristic	Predictive of missingness (P Value)[1]	VR+TAU (N=174)		TAU (N=172)	
		Missing (n=14)	Not missing (n=160)	Missing (n=10)	Not missing (n=162)
**Paranoia Worries Questionnaire (PWQ) total score, mean (SD) [n]**	0.095	10.8 (6.6) [12]	9.8 (6.2) [146]	13.0 (3.5) [8]	8.7 (6.2) [148]
**Patient Health Questionnaire (PHQ-9) total score, mean (SD) [n]**	0.880	14.5 (5.8) [13]	15.2 (6.1) [153]	14.3 (6.3) [9]	14.1 (6.5) [153]
**Columbia Suicide Severity Rating Scale (C-SSRS) total score, mean (SD) [n]**	0.716	0.6 (0.9) [14]	1.1 (1.3) [141]	1.4 (1.8) [8]	0.9 (1.2) [146]
**EQ-5D-5L INDEX, mean (SD) [n]**	0.450	0.5 (0.3) [13]	0.5 (0.3) [159]	0.5 (0.3) [10]	0.6 (0.3) [160]
**EQ5D VAS score, mean (SD) [n]**	0.297	48.1 (15.2) [13]	51.9 (19.5) [158]	48.7 (16.6) [10]	53.5 (19.3) [160]
**Recovering Quality Of Life (REQOL-20) total score, mean (SD) [n]**	0.582	35.4 (15.3) [14]	33.4 (13.1) [152]	29.8 (11.1) [10]	35.9 (13.2) [153]
**Progress of Recovery (QPR) total score, mean (SD) [n]**	0.842	31.2 (9.4) [14]	26.8 (10.8) [159]	23.6 (9.3) [10]	28.4 (11.2) [160]

[1] P value obtained from a logistic regression of missingness of primary outcome against each of the baseline characteristics (randomised groups combined). Level of significance = 0.05
[2] Categories with small numbers treated as missing data
[3] Not mutually exclusive

5.6 Appendix VI. Treatment deliverer

The distribution of treatment deliverer across sites is summarised below:

TABLE 42: DISTRIBUTION OF TREATMENT DELIVERER ACROSS SITES

Profession of lead therapist	Bristol (N=74)	Manchester (N=58)	Newcastle (N=81)	Nottingham (N=63)	Oxford (N=70)	Total (N=346)
Psychologist, n (%)	28 (37.8)	8 (13.8)	36 (44.4)	28 (44.4)	11 (15.7)	111 (84.1)
Assistant psychologist, n (%)	-	-	-	-	21 (30.0)	21 (15.9)
Peer worker, n (%)	9 (12.2)	21 (36.2)	2 (2.5)	-	-	32 (24.2)
Other, n (%)	-	-	-	3 (4.8)	-	3 (2.3)
Missing, n (%)	37 (50.0)	29 (50.0)	43 (53.1)	32 (50.8)	38 (54.3)	179 (135.6)

5.7 Appendix VII. Baseline tables for CACE analysis population

Baseline characteristics of the participants included in the CACE analysis are reported below, stratified by compliance group.

TABLE 43: BASELINE CHARACTERISTICS BY COMPLIANCE

	Compliers (N=142)	Non-compliers (N=204)	Total (N=346)
**Age (years)**			
Mean (SD)	36.5 (12.7)	37.7 (12.4)	37.2 (12.5)
Median (IQR)	35.6 (25.6 to 44.8)	35.8 (27.7 to 46.8)	35.8 (27.1 to 45.6)
Min to Max	17.1 to 66.1	16.6 to 70.7	16.6 to 70.7
Missing	-	-	-
**Age at first contact with mental health services (years)**			
Mean (SD)	24.8 (9.8)	25.8 (10.9)	25.4 (10.4)
Median (IQR)	23.0 (18.0 to 29.0)	23.0 (18.0 to 32.0)	23.0 (18.0 to 31.0)
Min to Max	6.0 to 55.0	7.0 to 60.0	6.0 to 60.0
Missing	2	8	10
**Sex, n (%)**			
Female	47 (33.1%)	64 (31.4%)	111 (32.1%)
Male	95 (66.9%)	136 (66.7%)	231 (66.8%)
Other	0 (0.0%)	1 (0.5%)	1 (0.3%)
Prefer not to say	0 (0.0%)	2 (1.0%)	2 (0.6%)
Missing	0 (0.0%)	1 (0.5%)	1 (0.3%)
**Current Marital status, n (%)**			
Single	108 (76.1%)	161 (78.9%)	269 (77.7%)
Married/civil partnership	15 (10.6%)	20 (9.8%)	35 (10.1%)
Cohabiting	6 (4.2%)	10 (4.9%)	16 (4.6%)
Separated	2 (1.4%)	1 (0.5%)	3 (0.9%)
Divorced	7 (4.9%)	9 (4.4%)	16 (4.6%)
Widowed	2 (1.4%)	2 (1.0%)	4 (1.2%)
Missing	2 (1.4%)	1 (0.5%)	3 (0.9%)

	Compliers (N=142)	Non-compliers (N=204)	Total (N=346)
**Ethnic group, n (%)**			
White	122 (85.9%)	172 (84.3%)	294 (85.0%)
Black British	1 (0.7%)	1 (0.5%)	2 (0.6%)
Black African	1 (0.7%)	2 (1.0%)	3 (0.9%)
Black Caribbean	0 (0.0%)	4 (2.0%)	4 (1.2%)
Indian	0 (0.0%)	2 (1.0%)	2 (0.6%)
Black Other	1 (0.7%)	0 (0.0%)	1 (0.3%)
Pakistani	3 (2.1%)	3 (1.5%)	6 (1.7%)
Other	14 (9.9%)	19 (9.3%)	33 (9.5%)
Missing	0 (0.0%)	1 (0.5%)	1 (0.3%)
**Site[1], n (%)**			
Bristol	28 (19.7%)	46 (22.5%)	74 (21.4%)
Manchester	24 (16.9%)	34 (16.7%)	58 (16.8%)
Newcastle	37 (26.1%)	44 (21.6%)	81 (23.4%)
Nottingham	24 (16.9%)	39 (19.1%)	63 (18.2%)
Oxford	29 (20.4%)	41 (20.1%)	70 (20.2%)
**Service type[1], n (%)**			
Community MH team	87 (61.3%)	122 (59.8%)	209 (60.4%)
Early intervention	52 (36.6%)	81 (39.7%)	133 (38.4%)
In-patient	3 (2.1%)	1 (0.5%)	4 (1.2%)
**Employment, n (%)**			
Employed full-time (paid), n (%)	10 (7.0)	9 (4.4)	19 (5.5)
Employed part-time (paid), n (%)	3 (2.1)	5 (2.5)	8 (2.3)
Employed full-time (voluntary), n (%)	-	-	-
Employed part-time (voluntary), n (%)	1 (0.7)	4 (2.0)	5 (1.4)
Unemployed (on benefits), n (%)	91 (64.1)	143 (70.1)	234 (67.6)

	Compliers (N=142)	Non-compliers (N=204)	Total (N=346)
Unemployed (not on benefits), n (%)	7 (4.9)	6 (2.9)	13 (3.8)
Student or in training full-time, n (%)	3 (2.1)	8 (3.9)	11 (3.2)
Student or in training part-time, n (%)	2 (1.4)	2 (1.0)	4 (1.2)
Self-employed, n (%)	3 (2.1)	2 (1.0)	5 (1.4)
Home-maker, n (%)	1 (0.7)	2 (1.0)	3 (0.9)
Carer, n (%)	1 (0.7)	1 (0.5)	2 (0.6)
Retired, n (%)	4 (2.8)	3 (1.5)	7 (2.0)
Other, n (%)	-	3 (1.5)	3 (0.9)
Missing, n (%)	16 (11.3)	16 (7.8)	32 (9.2)
**Usual/Normal living arrangement, n (%)**			
Living alone (+/- children), n (%)	57 (40.1)	87 (42.6)	144 (41.6)
Living with husband/wife, n (%)	11 (7.7)	18 (8.8)	29 (8.4)
Living with partner, n (%)	8 (5.6)	9 (4.4)	17 (4.9)
Living with parents, n (%)	34 (23.9)	48 (23.5)	82 (23.7)
Living with other relatives, n (%)	7 (4.9)	12 (5.9)	19 (5.5)
Living with others (e.g. friends), n (%)	8 (5.6)	13 (6.4)	21 (6.1)
Missing, n (%)	17 (12.0)	17 (8.3)	34 (9.8)
**Mental health diagnosis (F-code), n (%)**			
20, n (%)	26 (18.3)	33 (16.2)	59 (17.1)
20.0, n (%)	17 (12.0)	37 (18.1)	54 (15.6)
20.5, n (%)	-	1 (0.5)	1 (0.3)
20.8, n (%)	-	1 (0.5)	1 (0.3)
20.9, n (%)	15 (10.6)	8 (3.9)	23 (6.6)
21, n (%)	1 (0.7)	2 (1.0)	3 (0.9)
22, n (%)	1 (0.7)	2 (1.0)	3 (0.9)
22.8, n (%)	1 (0.7)	-	1 (0.3)

	Compliers (N=142)	Non-compliers (N=204)	Total (N=346)
23, n (%)	-	2 (1.0)	2 (0.6)
23.1, n (%)	2 (1.4)	2 (1.0)	4 (1.2)
23.2, n (%)	-	1 (0.5)	1 (0.3)
23.9, n (%)	3 (2.1)	4 (2.0)	7 (2.0)
25, n (%)	3 (2.1)	2 (1.0)	5 (1.4)
25.0, n (%)	1 (0.7)	1 (0.5)	2 (0.6)
25.1, n (%)	1 (0.7)	2 (1.0)	3 (0.9)
25.9, n (%)	9 (6.3)	7 (3.4)	16 (4.6)
28, n (%)	2 (1.4)	3 (1.5)	5 (1.4)
29, n (%)	46 (32.4)	65 (31.9)	111 (32.1)
29.0, n (%)	4 (2.8)	7 (3.4)	11 (3.2)
31.2, n (%)	-	5 (2.5)	5 (1.4)
31.4, n (%)	-	1 (0.5)	1 (0.3)
31.5, n (%)	1 (0.7)	1 (0.5)	2 (0.6)
32.3, n (%)	6 (4.2)	14 (6.9)	20 (5.8)
33.3, n (%)	3 (2.1)	3 (1.5)	6 (1.7)
**Currently taking any medication[2], n (%)**			
Yes, n (%)	137 (96.5)	198 (97.1)	335 (96.8)
No, n (%)	5 (3.5)	6 (2.9)	11 (3.2)
Missing, n (%)	-	-	-
**Type of medications in use[3]:**			
Antipsychotic			
Yes, n (%)	*130 (91.5)*	*187 (91.7)*	*317 (91.6)*
No, n (%)	*11 (7.7)*	*17 (8.3)*	*28 (8.1)*
Missing, n (%)	*1 (0.7)*	-	*1 (0.3)*
Antidepressant			
Yes, n (%)	*80 (56.3)*	*119 (58.3)*	*199 (57.5)*
No, n (%)	*61 (43.0)*	*85 (41.7)*	*146 (42.2)*

	Compliers (N=142)	Non-compliers (N=204)	Total (N=346)
Missing, n (%)	*1 (0.7)*	*-*	*1 (0.3)*
Anxiolytic			
Yes, n (%)	*11 (7.7)*	*17 (8.3)*	*28 (8.1)*
No, n (%)	*129 (90.8)*	*187 (91.7)*	*316 (91.3)*
Missing, n (%)	*2 (1.4)*	*-*	*2 (0.6)*
Mood stabiliser			
Yes, n (%)	*16 (11.3)*	*17 (8.3)*	*33 (9.5)*
No, n (%)	*124 (87.3)*	*187 (91.7)*	*311 (89.9)*
Missing, n (%)	*2 (1.4)*	*-*	*2 (0.6)*
Hypnotic			
Yes, n (%)	*7 (4.9)*	*11 (5.4)*	*18 (5.2)*
No, n (%)	*133 (93.7)*	*193 (94.6)*	*326 (94.2)*
Missing, n (%)	*2 (1.4)*	*-*	*2 (0.6)*
Stimulant			
Yes, n (%)	*1 (0.7)*	*-*	*1 (0.3)*
No, n (%)	*139 (97.9)*	*204 (100.0)*	*343 (99.1)*
Missing, n (%)	*2 (1.4)*	*-*	*2 (0.6)*
**O-AS Avoidance score**			
Mean (SD)	3.3 (2.6)	3.3 (2.6)	3.3 (2.6)
Median (IQR)	3.0 (1.0 to 6.0)	3.0 (1.0 to 5.5)	3.0 (1.0 to 6.0)
Min to Max	0.0 to 8.0	0.0 to 8.0	0.0 to 8.0
Missing	1	-	1
**O-AS Distress score**			
Mean (SD)	51.9 (16.9)	52.1 (16.8)	52.0 (16.8)
Median (IQR)	53.5 (39.0 to 65.0)	53.5 (39.5 to 65.5)	53.5 (39.0 to 65.0)
Min to Max	4.0 to 80.0	10.0 to 80.0	4.0 to 80.0
Missing	-	-	-
**(Actigraphy) Mean number of steps**			

	Compliers (N=142)	Non-compliers (N=204)	Total (N=346)
Mean (SD)	4866.9 (3226.6)	4806.8 (2942.1)	4831.6 (3054.3)
Median (IQR)	4073.0 (2690.1 to 6553.9)	4276.5 (2614.4 to 6232.5)	4210.9 (2623.7 to 6387.7)
Min to Max	42.3 to 14776.9	136.4 to 15054.7	42.3 to 15054.7
Missing	66	96	162
**Time budget score**			
Mean (SD)	51.8 (17.2)	53.1 (17.1)	52.5 (17.1)
Median (IQR)	50.0 (39.5 to 63.0)	54.0 (41.0 to 64.0)	51.0 (40.0 to 63.0)
Min to Max	15.0 to 100.0	7.0 to 99.0	7.0 to 100.0
Missing	18	35	53
**Agoraphobia Mobility Inventory-Avoidance (AMI-A) score**			
Mean (SD)	3.3 (0.7)	3.2 (0.8)	3.3 (0.8)
Median (IQR)	3.3 (2.9 to 3.7)	3.2 (2.7 to 3.7)	3.3 (2.8 to 3.7)
Min to Max	1.1 to 4.8	1.1 to 4.8	1.1 to 4.8
Missing	4	11	15
**O-BAT - maximum number of steps avoided**			
Mean (SD)	2.8 (1.2)	2.7 (1.3)	2.7 (1.3)
Median (IQR)	3.0 (2.0 to 4.0)	3.0 (2.0 to 4.0)	3.0 (2.0 to 4.0)
Min to Max	0.0 to 5.0	0.0 to 5.0	0.0 to 5.0
Missing	67	88	155
**O-BAT Mean distress score**			
Mean (SD)	5.4 (2.0)	5.6 (2.0)	5.5 (2.0)
Median (IQR)	5.4 (4.0 to 7.0)	5.8 (4.0 to 7.0)	5.6 (4.0 to 7.0)
Min to Max	1.0 to 9.7	0.0 to 10.0	0.0 to 10.0
Missing	68	89	157
**R-GPTS-A (social reference) score**			
Mean (SD)	14.3 (9.3)	12.6 (9.1)	13.3 (9.2)
Median (IQR)	14.0 (7.0 to 21.0)	11.5 (5.0 to 20.0)	13.0 (6.0 to 20.0)

	Compliers (N=142)	Non-compliers (N=204)	Total (N=346)
Min to Max	0.0 to 32.0	0.0 to 32.0	0.0 to 32.0
Missing	11	16	27
**R-GPTS-B (persecution) score**			
Mean (SD)	17.6 (12.6)	14.4 (12.9)	15.7 (12.9)
Median (IQR)	17.0 (5.0 to 29.0)	10.5 (2.5 to 25.0)	14.0 (3.0 to 27.0)
Min to Max	0.0 to 40.0	0.0 to 40.0	0.0 to 40.0
Missing	11	16	27
**R-GPTS (overall) score**			
Mean (SD)	31.8 (20.7)	27.0 (20.8)	29.0 (20.8)
Median (IQR)	33.0 (14.0 to 48.0)	24.0 (8.0 to 44.5)	26.0 (10.0 to 47.0)
Min to Max	0.0 to 72.0	0.0 to 72.0	0.0 to 72.0
Missing	11	16	27
**Paranoia Worries Questionnaire (PWQ) total score**			
Mean (SD)	10.2 (6.3)	8.8 (6.1)	9.4 (6.2)
Median (IQR)	10.0 (5.0 to 15.0)	9.0 (3.0 to 13.5)	10.0 (4.0 to 15.0)
Min to Max	0.0 to 20.0	0.0 to 20.0	0.0 to 20.0
Missing	12	20	32
**Patient Health Questionnaire (PHQ-9) total score**			
Mean (SD)	15.3 (6.1)	14.1 (6.4)	14.6 (6.3)
Median (IQR)	15.0 (11.5 to 20.0)	14.0 (9.0 to 19.0)	15.0 (9.5 to 20.0)
Min to Max	2.0 to 27.0	2.0 to 27.0	2.0 to 27.0
Missing	6	12	18
**Columbia Suicide Severity Rating Scale (C-SSRS) total score**			
Mean (SD)	1.0 (1.3)	1.0 (1.3)	1.0 (1.3)
Median (IQR)	1.0 (0.0 to 2.0)	0.0 (0.0 to 2.0)	0.0 (0.0 to 2.0)
Min to Max	0.0 to 5.0	0.0 to 5.0	0.0 to 5.0
Missing	16	21	37
**EQ-5D-5L INDEX**			

	Compliers (N=142)	Non-compliers (N=204)	Total (N=346)
Mean (SD)	0.5 (0.3)	0.6 (0.3)	0.5 (0.3)
Median (IQR)	0.6 (0.3 to 0.7)	0.6 (0.4 to 0.8)	0.6 (0.4 to 0.7)
Min to Max	-0.2 to 1.0	-0.3 to 1.0	-0.3 to 1.0
Missing	2	2	4
**EQ5D VAS score**			
Mean (SD)	51.0 (19.1)	53.3 (19.2)	52.4 (19.2)
Median (IQR)	50.0 (40.0 to 65.0)	50.0 (40.0 to 70.0)	50.0 (40.0 to 70.0)
Min to Max	0.0 to 85.0	6.0 to 95.0	0.0 to 95.0
Missing	3	2	5
**Recovering Quality Of Life (REQOL-20) total score**			
Mean (SD)	32.9 (13.5)	35.7 (12.9)	34.5 (13.2)
Median (IQR)	32.0 (23.0 to 42.1)	36.0 (26.0 to 44.0)	34.0 (25.0 to 44.0)
Min to Max	3.0 to 63.0	5.0 to 72.0	3.0 to 72.0
Missing	5	12	17
**Progress of Recovery (QPR) total score**			
Mean (SD)	26.5 (10.4)	28.4 (11.2)	27.6 (10.9)
Median (IQR)	26.0 (20.0 to 34.0)	30.0 (22.0 to 36.0)	28.0 (20.0 to 35.0)
Min to Max	3.0 to 51.0	0.0 to 56.0	0.0 to 56.0
Missing	-	3	3

[1] Stratification variables
[2] From baseline CRF & medical record data
[3] Not mutually exclusive

5.8 Appendix VIII. Diagnostic plots

Post estimate plots of the model residuals from the linear mixed effects models for the primary and secondary analyses are shown below.

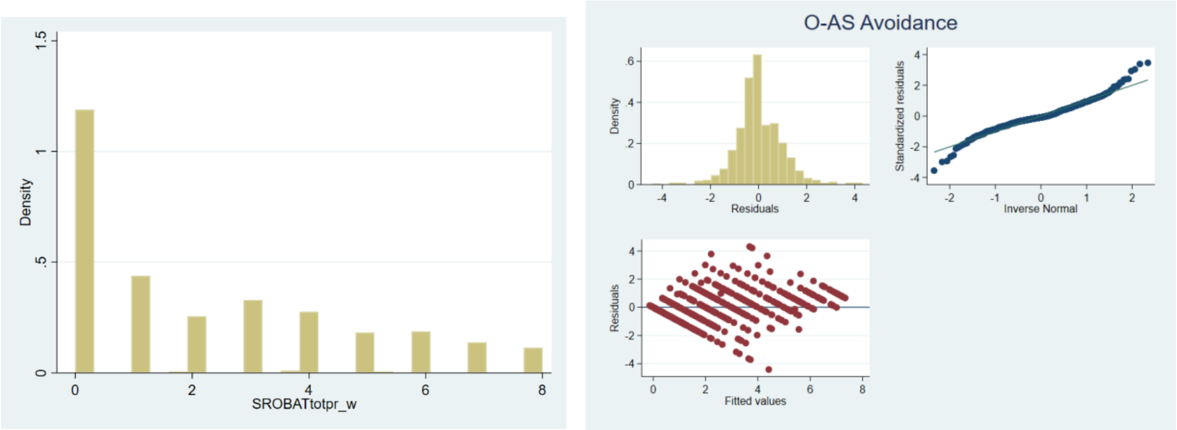

FIGURE 5: HISTOGRAMS AND MODEL RESIDUAL PLOTS FOR O-AS AVOIDANCE

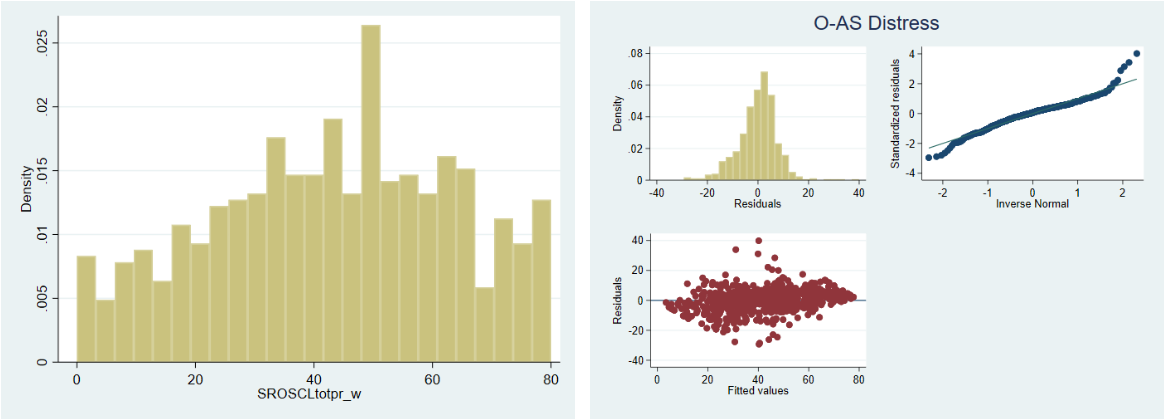

FIGURE 6: HISTOGRAMS AND MODEL RESIDUAL PLOTS FOR O-AS DISTRESS

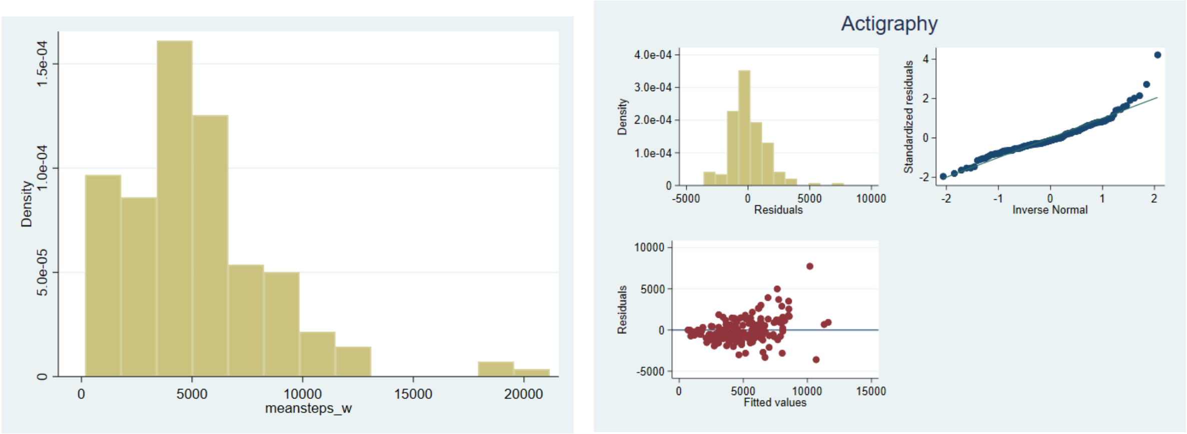

FIGURE 7: HISTOGRAMS AND MODEL RESIDUAL PLOTS FOR ACTIGRAPHY

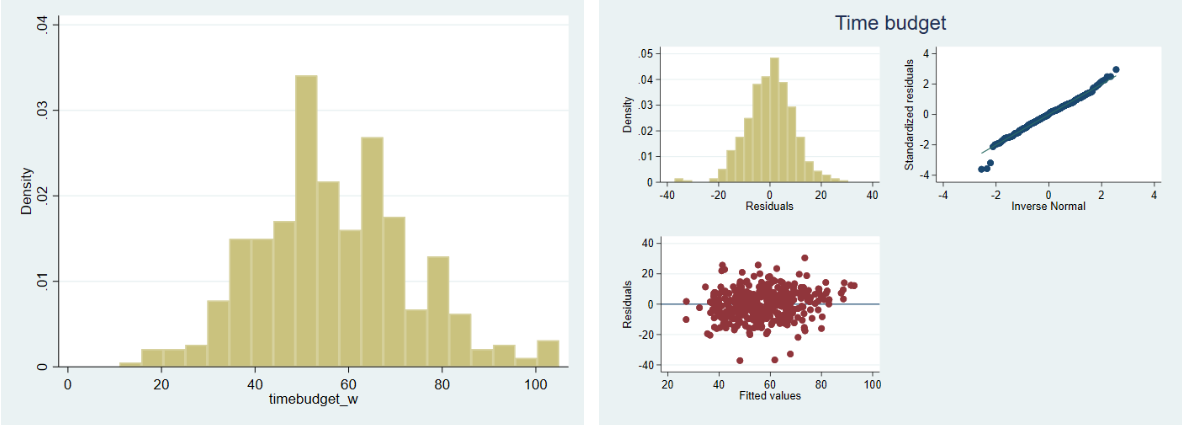

FIGURE 8 HISTOGRAMS AND MODEL RESIDUAL PLOTS FOR THE TIME BUDGET

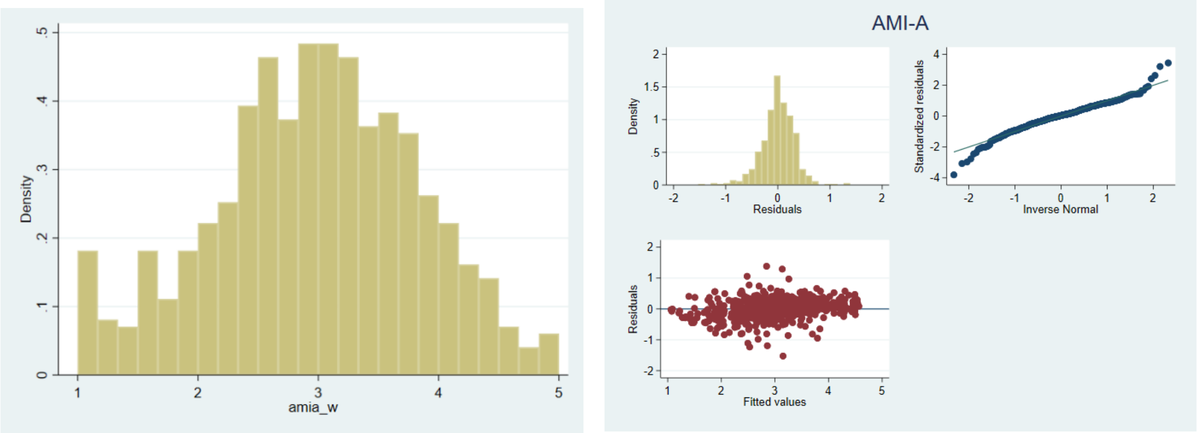

FIGURE 9 HISTOGRAMS AND MODEL RESIDUAL PLOTS FOR THE AGORAPHOBIA MOBILITY INVENTORY-AVOIDANCE [AMI-A]

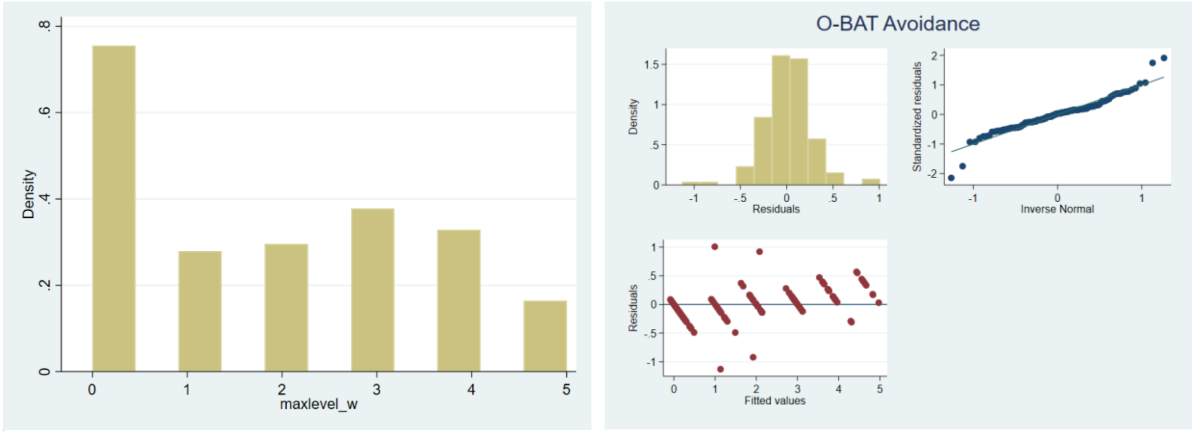

FIGURE 10 HISTOGRAM FOR THE O-BAT NUMBER STEPS AVOIDED

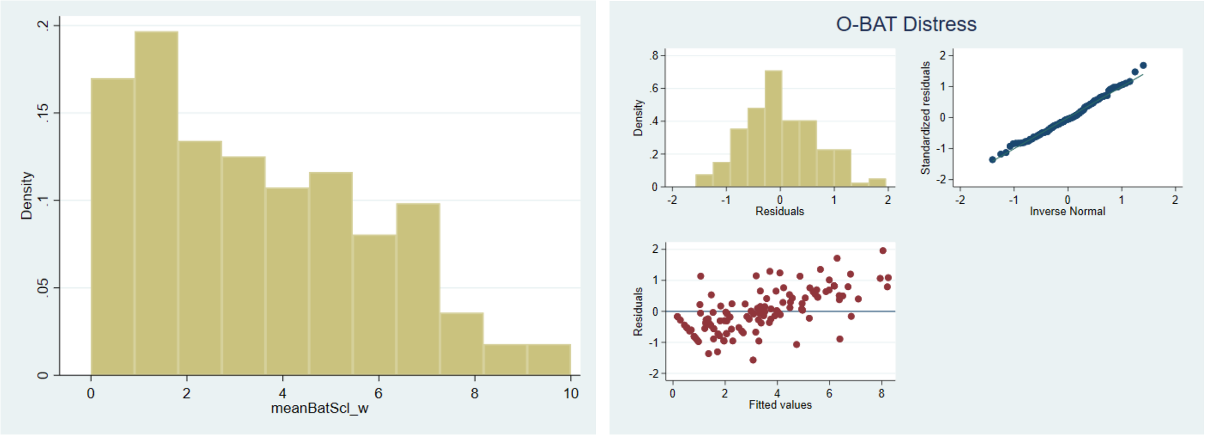

FIGURE 11 HISTOGRAMS AND MODEL RESIDUAL PLOTS FOR THE O-BAT MEAN DISTRESS SCORE

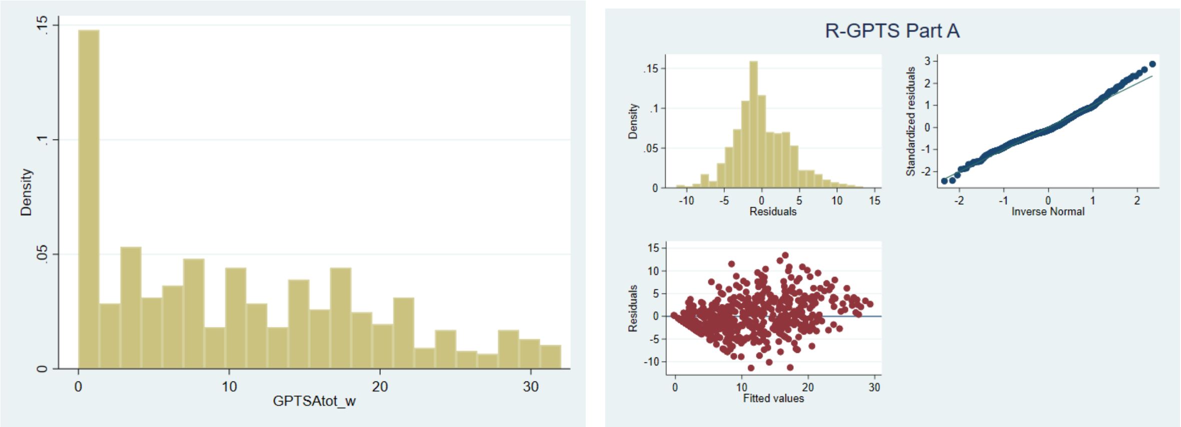

FIGURE 12 HISTOGRAMS AND MODEL RESIDUAL PLOTS FOR THE REVISED-GREEN ET AL PARANOID THOUGHTS SCALE [R-GPTS] PART A

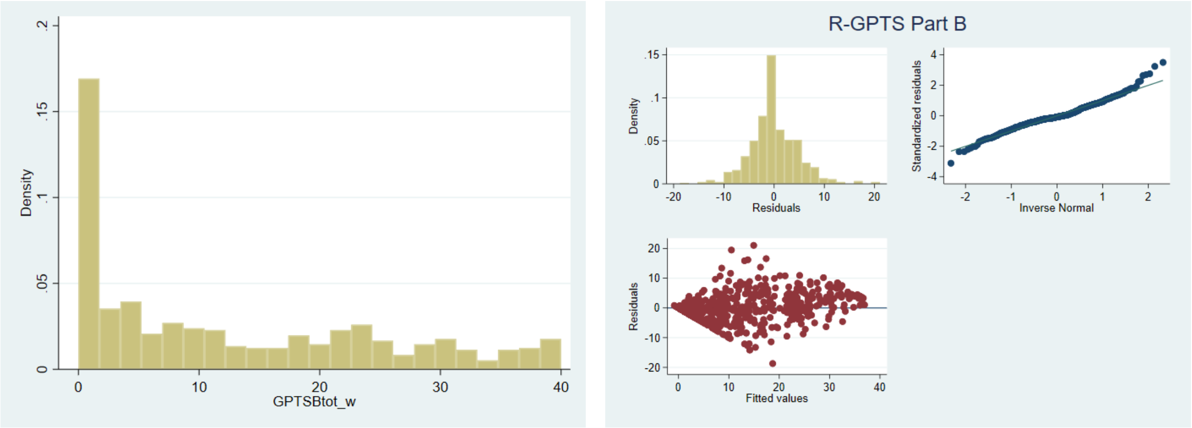

FIGURE 13 HISTOGRAMS AND MODEL RESIDUAL PLOTS FOR THE REVISED-GREEN ET AL PARANOID THOUGHTS SCALE [R-GPTS] PART B

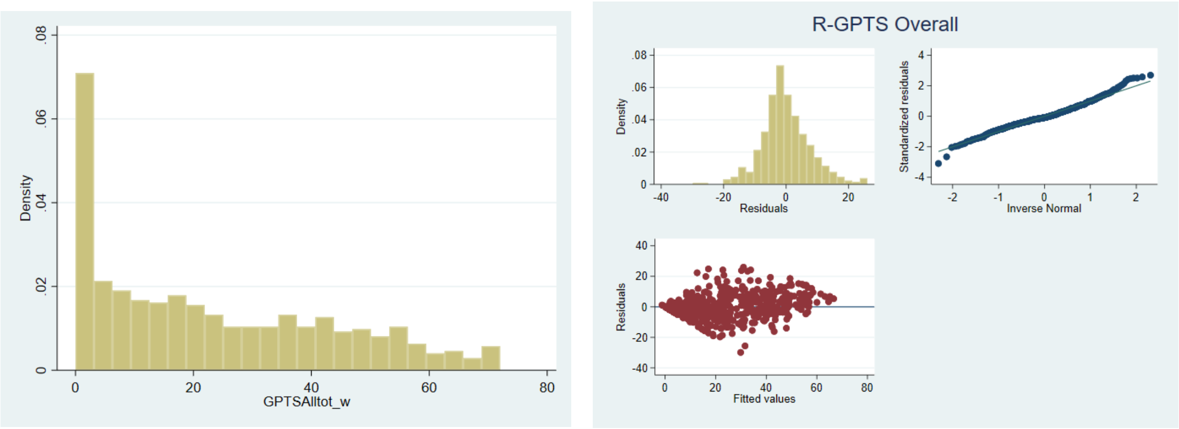

FIGURE 14 HISTOGRAMS AND MODEL RESIDUAL PLOTS FOR THE REVISED-GREEN ET AL PARANOID THOUGHTS SCALE [R-GPTS] OVERALL SCORE

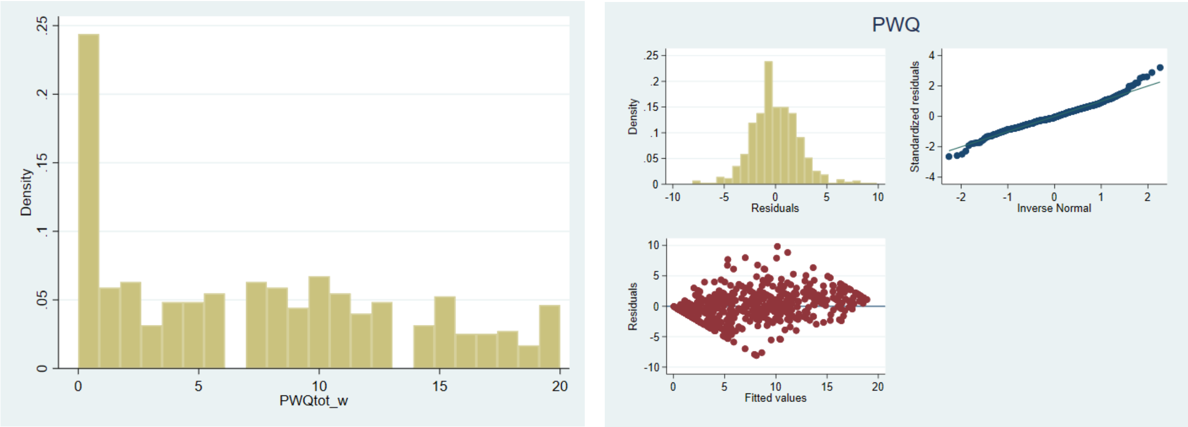

FIGURE 15 HISTOGRAMS AND MODEL RESIDUAL PLOTS FOR THE PARANOIA WORRIES QUESTIONNAIRE [PWQ]

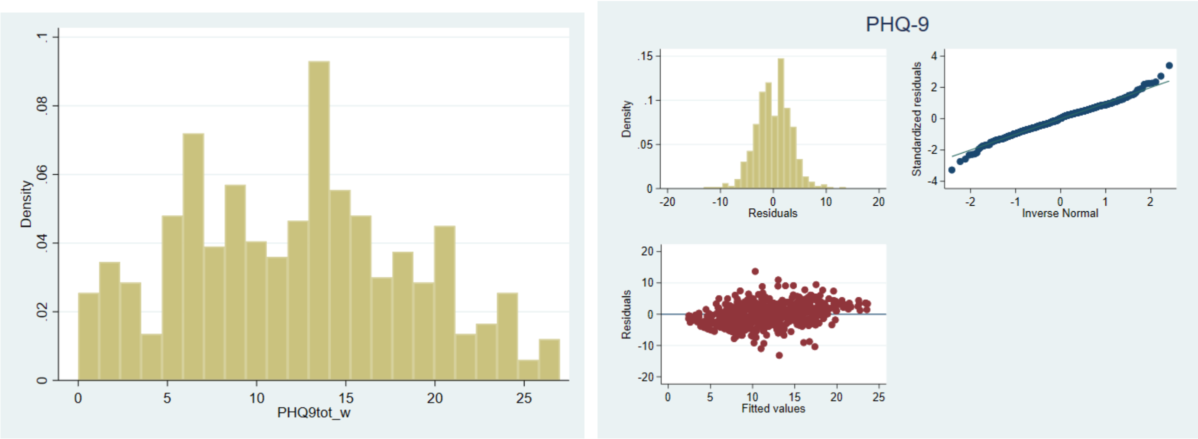

FIGURE 16 HISTOGRAMS AND MODEL RESIDUAL PLOTS FOR PATIENT HEALTH QUESTIONNAIRE (DEPRESSION, ANXIETY AND STRESS SCALE) [PHQ-9]

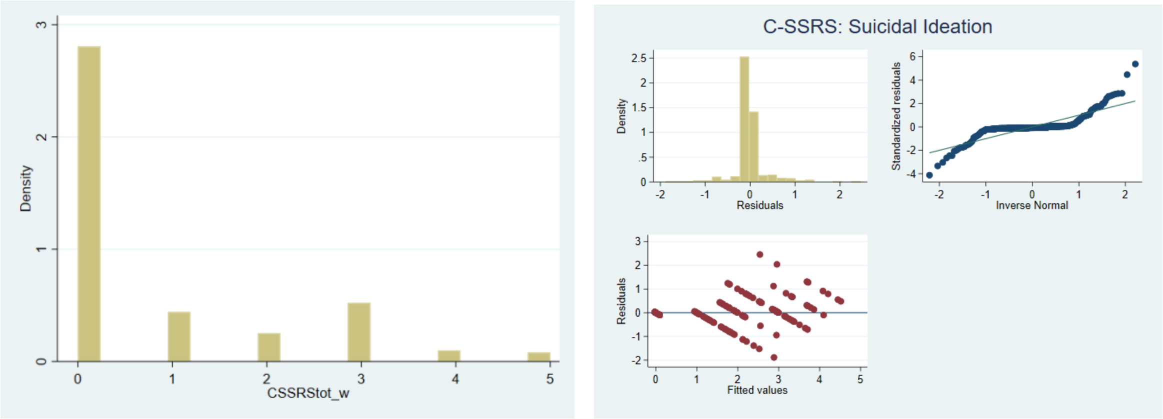

FIGURE 17 HISTOGRAMS AND MODEL RESIDUAL PLOTS FOR THE COLUMBIA SUICIDE SEVERITY RATING SCALE [C-SSRS]

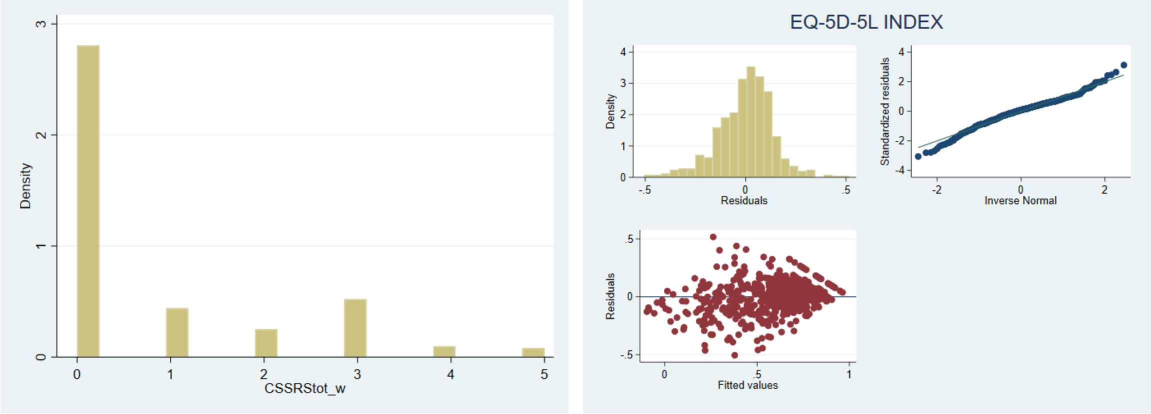

FIGURE 18 HISTOGRAMS AND MODEL RESIDUAL PLOTS FOR THE EQ-5D-5L INDEX

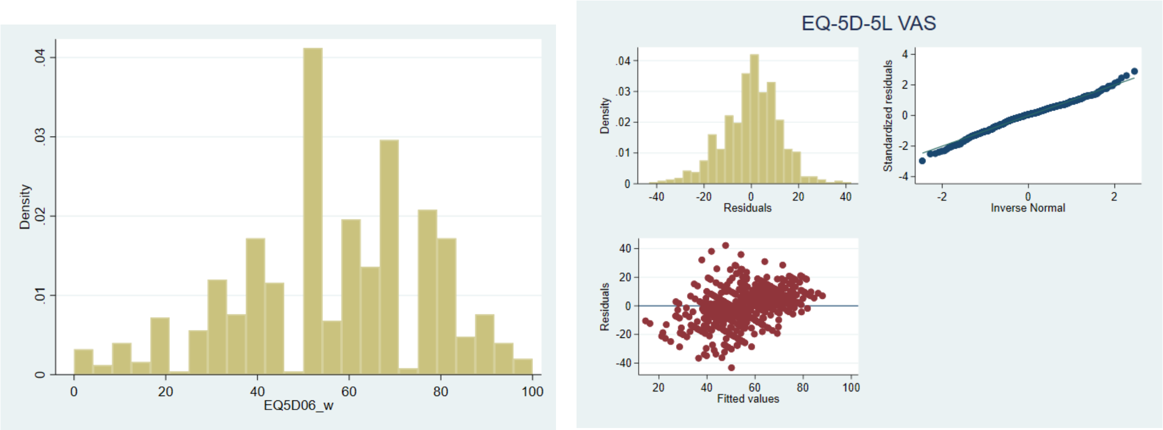

FIGURE 19 HISTOGRAMS AND MODEL RESIDUAL PLOTS FOR THE EQ-5D-5L VAS SCORE

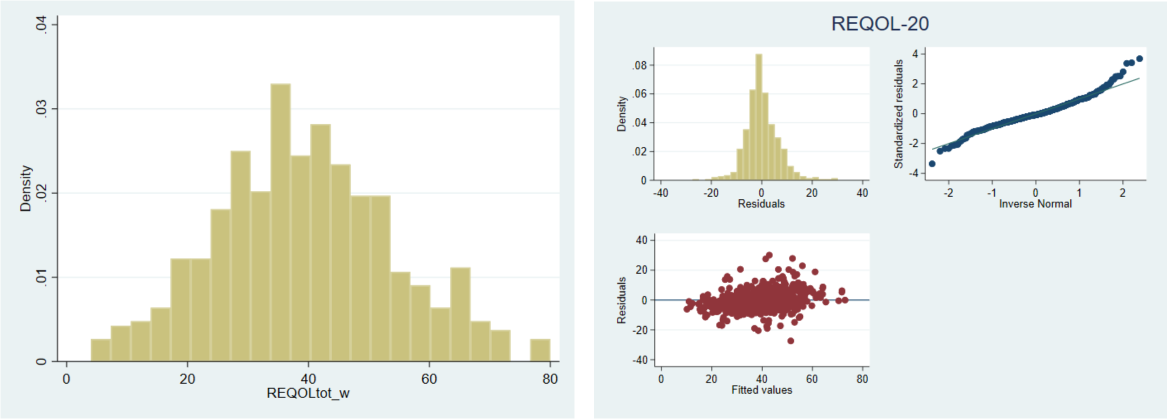

FIGURE 20 HISTOGRAMS AND MODEL RESIDUAL PLOTS FOR RECOVERING QUALITY OF LIFE [REQOL-20]

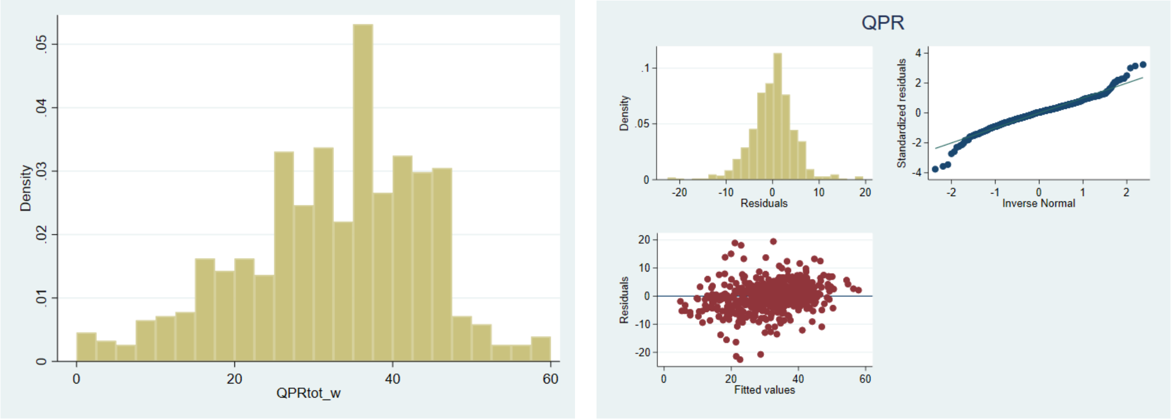

FIGURE 21 HISTOGRAMS AND MODEL RESIDUAL PLOTS FOR QUESTIONNAIRE ON PROGRESS OF RECOVERY [QPR]

5.9 Appendix VIX. Baseline tables for sensitivity analyses

The tables below summarise baseline characteristics separately for pre-lockdown and lockdown durations, where lockdown in the UK was implemented on 23 march 2020.

5.9.1 Pre-COVID-19 Lockdown

TABLE 44: BASELINE CHARACTERISTICS FOR THE PRE-LOCKDOWN POPULATION, BY RANDOMISED GROUP

	VR+TAU (N=98)	TAU (N=93)	Total (N=191)
**Age (years)**			
Mean (SD)	36.8 (13.3)	38.2 (12.7)	37.5 (13.0)
Median (IQR)	33.9 (25.1 to 47.7)	36.8 (28.2 to 46.6)	35.8 (25.9 to 47.7)
Min to Max	18.3 to 69.1	16.6 to 70.7	16.6 to 70.7
Missing	-	-	-
**Age at first contact with mental health services (years)**			
Mean (SD)	24.5 (10.2)	25.8 (11.0)	25.1 (10.6)
Median (IQR)	21.5 (18.0 to 28.5)	23.0 (18.0 to 34.0)	22.0 (18.0 to 30.0)
Min to Max	6.0 to 59.0	7.0 to 52.0	6.0 to 59.0
Missing	2	-	2
**Sex†, n (%)**			
Female	28 (28.6%)	25 (26.9%)	53 (27.7%)
Male	70 (71.4%)	67 (72.0%)	137 (71.7%)
Other	0 (0.0%)	1 (1.1%)	1 (0.5%)
Prefer not to say	. (.%)	. (.%)	. (.%)
Missing	. (.%)	. (.%)	. (.%)
**Current Marital status, n (%)**			
Single	75 (76.5%)	75 (80.6%)	150 (78.5%)
Married/civil partnership	9 (9.2%)	8 (8.6%)	17 (8.9%)
Cohabiting	4 (4.1%)	5 (5.4%)	9 (4.7%)
Separated	2 (2.0%)	1 (1.1%)	3 (1.6%)
Divorced	6 (6.1%)	3 (3.2%)	9 (4.7%)
Widowed	2 (2.0%)	1 (1.1%)	3 (1.6%)

	VR+TAU (N=98)	TAU (N=93)	Total (N=191)
Missing	. (.%)	. (.%)	. (.%)
**Ethnic group, n (%)**			
White	84 (85.7%)	80 (86.0%)	164 (85.9%)
Black British	1 (1.0%)	0 (0.0%)	1 (0.5%)
Black African	1 (1.0%)	2 (2.2%)	3 (1.6%)
Black Caribbean	0 (0.0%)	1 (1.1%)	1 (0.5%)
Indian	0 (0.0%)	2 (2.2%)	2 (1.0%)
Black Other	1 (1.0%)	0 (0.0%)	1 (0.5%)
Pakistani	0 (0.0%)	1 (1.1%)	1 (0.5%)
Other	11 (11.2%)	7 (7.5%)	18 (9.4%)
Missing	. (.%)	. (.%)	. (.%)
**Site[1], n (%)**			
Bristol	19 (19.4%)	18 (19.4%)	37 (19.4%)
Manchester	21 (21.4%)	17 (18.3%)	38 (19.9%)
Newcastle	26 (26.5%)	24 (25.8%)	50 (26.2%)
Nottingham	15 (15.3%)	16 (17.2%)	31 (16.2%)
Oxford	17 (17.3%)	18 (19.4%)	35 (18.3%)
**Service type[1], n (%)**			
Community MH team	60 (61.2%)	58 (62.4%)	118 (61.8%)
Early intervention	35 (35.7%)	34 (36.6%)	69 (36.1%)
In-patient	3 (3.1%)	1 (1.1%)	4 (2.1%)
**Employment, n (%)**			
Employed full-time (paid), n (%)	3 (3.1)	7 (7.5)	10 (5.2)
Employed part-time (paid), n (%)	2 (2.0)	4 (4.3)	6 (3.1)
Employed full-time (voluntary), n (%)	-	-	-
Employed part-time (voluntary), n (%)	2 (2.0)	1 (1.1)	3 (1.6)
Unemployed (on benefits), n (%)	71 (72.4)	66 (71.0)	137 (71.7)

	VR+TAU (N=98)	TAU (N=93)	Total (N=191)
Unemployed (not on benefits), n (%)	5 (5.1)	5 (5.4)	10 (5.2)
Student or in training full-time, n (%)	3 (3.1)	3 (3.2)	6 (3.1)
Student or in training part-time, n (%)	2 (2.0)	-	2 (1.0)
Self-employed, n (%)	2 (2.0)	1 (1.1)	3 (1.6)
Home-maker, n (%)	-	1 (1.1)	1 (0.5)
Carer, n (%)	-	-	-
Retired, n (%)	3 (3.1)	1 (1.1)	4 (2.1)
Other, n (%)	-	2 (2.2)	2 (1.0)
Missing, n (%)	5 (5.1)	2 (2.2)	7 (3.7)
**Usual/Normal living arrangement, n (%)**			
Living alone (+/- children), n (%)	46 (46.9)	39 (41.9)	85 (44.5)
Living with husband/wife, n (%)	9 (9.2)	8 (8.6)	17 (8.9)
Living with partner, n (%)	4 (4.1)	6 (6.5)	10 (5.2)
Living with parents, n (%)	22 (22.4)	21 (22.6)	43 (22.5)
Living with other relatives, n (%)	6 (6.1)	9 (9.7)	15 (7.9)
Living with others (e.g. friends), n (%)	6 (6.1)	8 (8.6)	14 (7.3)
Missing, n (%)	5 (5.1)	2 (2.2)	7 (3.7)
**Mental health diagnosis (F-code), n (%)**			
20, n (%)	18 (18.4)	13 (14.0)	31 (16.2)
20.0, n (%)	16 (16.3)	20 (21.5)	36 (18.8)
20.5, n (%)	-	-	0 (0.0)
20.8, n (%)	-	-	0 (0.0)
20.9, n (%)	11 (11.2)	4 (4.3)	15 (7.9)
21, n (%)	-	-	0 (0.0)
22, n (%)	1 (1.0)	-	1 (0.5)
22.8, n (%)	1 (1.0)	-	1 (0.5)

	VR+TAU (N=98)	TAU (N=93)	Total (N=191)
23, n (%)	-	1 (1.1)	1 (0.5)
23.1, n (%)	-	-	0 (0.0)
23.2, n (%)	-	-	0 (0.0)
23.9, n (%)	3 (3.1)	-	3 (1.6)
25, n (%)	1 (1.0)	2 (2.2)	3 (1.6)
25.0, n (%)	1 (1.0)	-	1 (0.5)
25.1, n (%)	1 (1.0)	1 (1.1)	2 (1.0)
25.9, n (%)	4 (4.1)	5 (5.4)	9 (4.7)
28, n (%)	2 (2.0)	1 (1.1)	3 (1.6)
29, n (%)	30 (30.6)	26 (28.0)	56 (29.3)
29.0, n (%)	3 (3.1)	6 (6.5)	9 (4.7)
31.2, n (%)	-	2 (2.2)	2 (1.0)
31.4, n (%)	-	1 (1.1)	1 (0.5)
31.5, n (%)	1 (1.0)	-	1 (0.5)
32.3, n (%)	3 (3.1)	8 (8.6)	11 (5.8)
33.3, n (%)	2 (2.0)	3 (3.2)	5 (2.6)
**Currently taking any medication[2], n (%)**			
Yes, n (%)	95 (96.9)	89 (95.7)	184 (96.3)
No, n (%)	3 (3.1)	4 (4.3)	7 (3.7)
Missing, n (%)	-	-	-
**Type of medications in use[3]:**			
Antipsychotic			
Yes, n (%)	*90 (91.8)*	*84 (90.3)*	*174 (91.1)*
No, n (%)	*7 (7.1)*	*9 (9.7)*	*16 (8.4)*
Missing, n (%)	*1 (1.0)*	-	*1 (0.5)*
Antidepressant			
Yes, n (%)	*61 (62.2)*	*52 (55.9)*	*113 (59.2)*
No, n (%)	*36 (36.7)*	*41 (44.1)*	*77 (40.3)*

	VR+TAU (N=98)	TAU (N=93)	Total (N=191)
Missing, n (%)	*1 (1.0)*	*-*	*1 (0.5)*
Anxiolytic			
Yes, n (%)	*12 (12.2)*	*9 (9.7)*	*21 (11.0)*
No, n (%)	*85 (86.7)*	*84 (90.3)*	*169 (88.5)*
Missing, n (%)	*1 (1.0)*	*-*	*1 (0.5)*
Mood stabiliser			
Yes, n (%)	*7 (7.1)*	*8 (8.6)*	*15 (7.9)*
No, n (%)	*90 (91.8)*	*85 (91.4)*	*175 (91.6)*
Missing, n (%)	*1 (1.0)*	*-*	*1 (0.5)*
Hypnotic			
Yes, n (%)	*5 (5.1)*	*5 (5.4)*	*10 (5.2)*
No, n (%)	*92 (93.9)*	*88 (94.6)*	*180 (94.2)*
Missing, n (%)	*1 (1.0)*	*-*	*1 (0.5)*
Stimulant			
Yes, n (%)	*1 (1.0)*	*-*	*1 (0.5)*
No, n (%)	*96 (98.0)*	*93 (100.0)*	*189 (99.0)*
Missing, n (%)	*1 (1.0)*	*-*	*1 (0.5)*
**O-AS Avoidance score**			
Mean (SD)	3.1 (2.4)	3.5 (2.8)	3.3 (2.6)
Median (IQR)	3.0 (1.0 to 5.0)	3.0 (1.0 to 6.0)	3.0 (1.0 to 6.0)
Min to Max	0.0 to 8.0	0.0 to 8.0	0.0 to 8.0
Missing	-	-	-
**O-AS Distress score**			
Mean (SD)	52.7 (15.5)	53.6 (17.0)	53.2 (16.2)
Median (IQR)	54.0 (39.0 to 65.0)	56.0 (42.0 to 66.0)	55.0 (41.0 to 66.0)
Min to Max	18.0 to 80.0	13.0 to 80.0	13.0 to 80.0
Missing	-	-	-
**(Actigraphy) Mean number of steps**			

	VR+TAU (N=98)	TAU (N=93)	Total (N=191)
Mean (SD)	4552.5 (3193.0)	5275.6 (3377.6)	4892.5 (3286.9)
Median (IQR)	4073.0 (2254.9 to 5963.6)	4951.2 (2177.6 to 7425.4)	4233.0 (2254.9 to 6646.1)
Min to Max	42.3 to 14776.9	348.9 to 15054.7	42.3 to 15054.7
Missing	36	38	74
**Time budget score**			
Mean (SD)	49.1 (16.5)	53.0 (15.9)	51.0 (16.3)
Median (IQR)	48.0 (38.5 to 60.0)	50.5 (41.0 to 63.0)	48.0 (40.0 to 61.0)
Min to Max	7.0 to 100.0	21.0 to 89.0	7.0 to 100.0
Missing	10	11	21
**Agoraphobia Mobility Inventory-Avoidance (AMI-A) score**			
Mean (SD)	3.3 (0.7)	3.2 (0.8)	3.3 (0.7)
Median (IQR)	3.4 (3.0 to 3.7)	3.1 (2.7 to 3.7)	3.2 (2.8 to 3.7)
Min to Max	1.5 to 4.8	1.5 to 4.8	1.5 to 4.8
Missing	7	7	14
**O-BAT - maximum number of steps avoided**			
Mean (SD)	2.7 (1.3)	2.8 (1.3)	2.7 (1.3)
Median (IQR)	3.0 (2.0 to 4.0)	3.0 (2.0 to 4.0)	3.0 (2.0 to 4.0)
Min to Max	0.0 to 5.0	0.0 to 5.0	0.0 to 5.0
Missing	-	-	-
**O-BAT Mean distress score**			
Mean (SD)	5.5 (1.9)	5.5 (2.1)	5.5 (2.0)
Median (IQR)	5.5 (4.0 to 7.0)	5.6 (4.0 to 7.0)	5.6 (4.0 to 7.0)
Min to Max	1.0 to 10.0	0.0 to 10.0	0.0 to 10.0
Missing	1	1	2
**R-GPTS-A (social reference) score**			
Mean (SD)	13.8 (8.9)	12.6 (8.7)	13.2 (8.8)
Median (IQR)	14.0 (6.0 to 20.0)	12.0 (5.0 to 19.0)	13.0 (6.0 to 20.0)

	VR+TAU (N=98)	TAU (N=93)	Total (N=191)
Min to Max	0.0 to 31.0	0.0 to 32.0	0.0 to 32.0
Missing	11	6	17
**R-GPTS-B (persecution) score**			
Mean (SD)	16.6 (12.4)	15.0 (12.8)	15.8 (12.6)
Median (IQR)	16.0 (4.0 to 29.0)	12.0 (3.0 to 26.0)	15.0 (4.0 to 28.0)
Min to Max	0.0 to 38.0	0.0 to 40.0	0.0 to 40.0
Missing	11	6	17
**R-GPTS (overall) score**			
Mean (SD)	30.4 (19.9)	27.6 (20.4)	29.0 (20.2)
Median (IQR)	32.0 (13.0 to 45.0)	24.0 (8.0 to 47.0)	26.0 (10.0 to 46.0)
Min to Max	0.0 to 69.0	0.0 to 72.0	0.0 to 72.0
Missing	11	6	17
**Paranoia Worries Questionnaire (PWQ) total score**			
Mean (SD)	9.9 (6.3)	9.4 (5.9)	9.6 (6.1)
Median (IQR)	9.0 (5.5 to 15.0)	10.0 (5.0 to 14.0)	10.0 (5.0 to 15.0)
Min to Max	0.0 to 20.0	0.0 to 20.0	0.0 to 20.0
Missing	10	10	20
**Patient Health Questionnaire (PHQ-9) total score**			
Mean (SD)	15.2 (5.9)	14.4 (6.3)	14.8 (6.1)
Median (IQR)	15.0 (11.0 to 20.0)	15.0 (10.0 to 19.0)	15.0 (10.0 to 20.0)
Min to Max	4.0 to 27.0	2.0 to 26.0	2.0 to 27.0
Missing	3	5	8
**Columbia Suicide Severity Rating Scale (C-SSRS) total score**			
Mean (SD)	1.3 (1.4)	1.1 (1.3)	1.2 (1.4)
Median (IQR)	1.0 (0.0 to 3.0)	1.0 (0.0 to 2.0)	1.0 (0.0 to 2.0)
Min to Max	0.0 to 5.0	0.0 to 5.0	0.0 to 5.0
Missing	7	7	14
**EQ-5D-5L INDEX**			

	VR+TAU (N=98)	TAU (N=93)	Total (N=191)
Mean (SD)	0.6 (0.3)	0.5 (0.3)	0.5 (0.3)
Median (IQR)	0.6 (0.4 to 0.7)	0.6 (0.4 to 0.7)	0.6 (0.4 to 0.7)
Min to Max	-0.2 to 0.9	-0.3 to 1.0	-0.3 to 1.0
Missing	-	1	1
**EQ5D VAS score**			
Mean (SD)	49.8 (19.3)	48.9 (17.8)	49.4 (18.5)
Median (IQR)	50.0 (35.0 to 65.0)	50.0 (40.0 to 60.0)	50.0 (35.0 to 60.0)
Min to Max	0.0 to 90.0	15.0 to 91.0	0.0 to 91.0
Missing	-	-	-
**Recovering Quality Of Life (REQOL-20) total score**			
Mean (SD)	33.2 (12.0)	35.2 (12.5)	34.2 (12.3)
Median (IQR)	32.5 (26.0 to 42.0)	35.5 (25.0 to 43.5)	34.0 (25.0 to 42.0)
Min to Max	6.7 to 59.0	13.0 to 72.0	6.7 to 72.0
Missing	6	5	11
**Progress of Recovery (QPR) total score**			
Mean (SD)	26.6 (10.2)	27.4 (9.7)	27.0 (10.0)
Median (IQR)	27.0 (20.0 to 33.0)	28.0 (20.0 to 34.0)	27.5 (20.0 to 33.5)
Min to Max	0.0 to 47.0	4.0 to 52.0	0.0 to 52.0
Missing	1	2	3

† Missing value later identified as Male
[1] Stratification variables
[2] From baseline CRF & medical record data
[3] Not mutually exclusive

5.9.2 COVID-19 Lockdown

TABLE 45: BASELINE CHARACTERISTICS FOR THE LOCKDOWN POPULATION, BY RANDOMISED GROUP

	VR+TAU (N=76)	TAU (N=79)	Total (N=155)
**Age (years)**			
Mean (SD)	36.3 (12.2)	37.3 (11.6)	36.8 (11.9)
Median (IQR)	35.6 (27.5 to 42.8)	35.9 (27.7 to 45.7)	35.9 (27.7 to 44.4)
Min to Max	17.1 to 66.1	17.0 to 62.4	17.0 to 66.1
Missing	-	-	-
**Age at first contact with mental health services (years)**			
Mean (SD)	24.9 (9.1)	26.6 (11.2)	25.7 (10.2)
Median (IQR)	23.0 (19.0 to 28.0)	23.5 (18.0 to 32.0)	23.0 (18.0 to 31.0)
Min to Max	7.0 to 55.0	7.0 to 60.0	7.0 to 60.0
Missing	1	7	8
**Sex, n (%)**			
Female	30 (39.5%)	28 (35.4%)	58 (37.4%)
Male	46 (60.5%)	48 (60.8%)	94 (60.6%)
Other	0 (0.0%)	1 (1.3%)	1 (0.6%)
Prefer not to say	0 (0.0%)	1 (1.3%)	1 (0.6%)
Missing	0 (0.0%)	1 (1.3%)	1 (0.6%)
**Current Marital status, n (%)**			
Single	56 (73.7%)	63 (79.7%)	119 (76.8%)
Married/civil partnership	12 (15.8%)	6 (7.6%)	18 (11.6%)
Cohabiting	2 (2.6%)	5 (6.3%)	7 (4.5%)
Separated	3 (3.9%)	4 (5.1%)	7 (4.5%)
Divorced	1 (1.3%)	0 (0.0%)	1 (0.6%)
Widowed	2 (2.6%)	1 (1.3%)	3 (1.9%)
Missing	. (.%)	. (.%)	. (.%)
**Ethnic group, n (%)**			

	VR+TAU (N=76)	TAU (N=79)	Total (N=155)
White	68 (89.5%)	62 (78.5%)	130 (83.9%)
Black British	0 (0.0%)	1 (1.3%)	1 (0.6%)
Black African	0 (0.0%)	3 (3.8%)	3 (1.9%)
Black Caribbean	3 (3.9%)	2 (2.5%)	5 (3.2%)
Indian	5 (6.6%)	10 (12.7%)	15 (9.7%)
Black Other	0 (0.0%)	1 (1.3%)	1 (0.6%)
Pakistani	. (.%)	. (.%)	. (.%)
Other	. (.%)	. (.%)	. (.%)
Missing	. (.%)	. (.%)	. (.%)
**Site[1], n (%)**			
Bristol	18 (23.7%)	19 (24.1%)	37 (23.9%)
Manchester	8 (10.5%)	12 (15.2%)	20 (12.9%)
Newcastle	16 (21.1%)	15 (19.0%)	31 (20.0%)
Nottingham	17 (22.4%)	15 (19.0%)	32 (20.6%)
Oxford	17 (22.4%)	18 (22.8%)	35 (22.6%)
**Service type[1], n (%)**			
Community MH team	47 (61.8%)	44 (55.7%)	91 (58.7%)
Early intervention	29 (38.2%)	35 (44.3%)	64 (41.3%)
**Employment, n (%)**			
Employed full-time (paid), n (%)	7 (9.2)	2 (2.5)	9 (5.8)
Employed part-time (paid), n (%)	2 (2.6)	-	2 (1.3)
Employed full-time (voluntary), n (%)	-	-	-
Employed part-time (voluntary), n (%)	-	2 (2.5)	2 (1.3)
Unemployed (on benefits), n (%)	41 (53.9)	56 (70.9)	97 (62.6)
Unemployed (not on benefits), n (%)	3 (3.9)	-	3 (1.9)

	VR+TAU (N=76)	TAU (N=79)	Total (N=155)
Student or in training full-time, n (%)	2 (2.6)	3 (3.8)	5 (3.2)
Student or in training part-time, n (%)	1 (1.3)	1 (1.3)	2 (1.3)
Self-employed, n (%)	2 (2.6)	-	2 (1.3)
Home-maker, n (%)	2 (2.6)	-	2 (1.3)
Carer, n (%)	1 (1.3)	1 (1.3)	2 (1.3)
Retired, n (%)	2 (2.6)	1 (1.3)	3 (1.9)
Other, n (%)	-	1 (1.3)	1 (0.6)
Missing, n (%)	13 (17.1)	12 (15.2)	25 (16.1)
**Usual/Normal living arrangement, n (%)**			
Living alone (+/- children), n (%)	26 (34.2)	33 (41.8)	59 (38.1)
Living with husband/wife, n (%)	7 (9.2)	5 (6.3)	12 (7.7)
Living with partner, n (%)	4 (5.3)	3 (3.8)	7 (4.5)
Living with parents, n (%)	18 (23.7)	21 (26.6)	39 (25.2)
Living with other relatives, n (%)	3 (3.9)	1 (1.3)	4 (2.6)
Living with others (e.g. friends), n (%)	4 (5.3)	3 (3.8)	7 (4.5)
Missing, n (%)	14 (18.4)	13 (16.5)	27 (17.4)
**Mental health diagnosis (F-code), n (%)**			
20, n (%)	14 (18.4)	14 (17.7)	28 (18.1)
20.0, n (%)	9 (11.8)	9 (11.4)	18 (11.6)
20.5, n (%)	-	1 (1.3)	1 (0.6)
20.8, n (%)	-	1 (1.3)	1 (0.6)
20.9, n (%)	6 (7.9)	2 (2.5)	8 (5.2)
21, n (%)	1 (1.3)	2 (2.5)	3 (1.9)
22, n (%)	-	2 (2.5)	2 (1.3)
22.8, n (%)	-	-	0 (0.0)
23, n (%)	-	1 (1.3)	1 (0.6)

	VR+TAU (N=76)	TAU (N=79)	Total (N=155)
23.1, n (%)	2 (2.6)	2 (2.5)	4 (2.6)
23.2, n (%)	-	1 (1.3)	1 (0.6)
23.9, n (%)	-	4 (5.1)	4 (2.6)
25, n (%)	2 (2.6)	-	2 (1.3)
25.0, n (%)	1 (1.3)	-	1 (0.6)
25.1, n (%)	-	1 (1.3)	1 (0.6)
25.9, n (%)	5 (6.6)	2 (2.5)	7 (4.5)
28, n (%)	-	2 (2.5)	2 (1.3)
29, n (%)	27 (35.5)	28 (35.4)	55 (35.5)
29.0, n (%)	2 (2.6)	-	2 (1.3)
31.2, n (%)	1 (1.3)	2 (2.5)	3 (1.9)
31.4, n (%)	-	-	0 (0.0)
31.5, n (%)	1 (1.3)	-	1 (0.6)
32.3, n (%)	4 (5.3)	5 (6.3)	9 (5.8)
33.3, n (%)	1 (1.3)	-	1 (0.6)
**Currently taking any medication², n (%)**			
Yes, n (%)	74 (97.4)	77 (97.5)	151 (97.4)
No, n (%)	2 (2.6)	2 (2.5)	4 (2.6)
Missing, n (%)	-	-	-
**Type of medications in use³:**			
Antipsychotic			
Yes, n (%)	*71 (93.4)*	*72 (91.1)*	*143 (92.3)*
No, n (%)	*5 (6.6)*	*7 (8.9)*	*12 (7.7)*
Missing, n (%)	-	-	-
Antidepressant			
Yes, n (%)	*42 (55.3)*	*44 (55.7)*	*86 (55.5)*
No, n (%)	*34 (44.7)*	*35 (44.3)*	*69 (44.5)*
Missing, n (%)	-	-	-

	VR+TAU (N=76)	TAU (N=79)	Total (N=155)
Anxiolytic			
Yes, n (%)	*3 (3.9)*	*4 (5.1)*	*7 (4.5)*
No, n (%)	*72 (94.7)*	*75 (94.9)*	*147 (94.8)*
Missing, n (%)	*1 (1.3)*	*-*	*1 (0.6)*
Mood stabiliser			
Yes, n (%)	*11 (14.5)*	*7 (8.9)*	*18 (11.6)*
No, n (%)	*64 (84.2)*	*72 (91.1)*	*136 (87.7)*
Missing, n (%)	*1 (1.3)*	*-*	*1 (0.6)*
Hypnotic			
Yes, n (%)	*6 (7.9)*	*2 (2.5)*	*8 (5.2)*
No, n (%)	*69 (90.8)*	*77 (97.5)*	*146 (94.2)*
Missing, n (%)	*1 (1.3)*	*-*	*1 (0.6)*
Stimulant			
Yes, n (%)	*-*	*-*	*-*
No, n (%)	*75 (98.7)*	*79 (100.0)*	*154 (99.4)*
Missing, n (%)	*1 (1.3)*	*-*	*1 (0.6)*
**O-AS Avoidance score**			
Mean (SD)	3.2 (2.7)	3.3 (2.6)	3.2 (2.6)
Median (IQR)	2.0 (1.0 to 6.0)	3.0 (1.0 to 5.0)	3.0 (1.0 to 5.0)
Min to Max	0.0 to 8.0	0.0 to 8.0	0.0 to 8.0
Missing	1	-	1
**O-AS Distress score**			
Mean (SD)	49.7 (17.5)	51.3 (17.5)	50.5 (17.5)
Median (IQR)	51.5 (36.0 to 62.0)	53.0 (40.0 to 66.0)	53.0 (37.0 to 64.0)
Min to Max	4.0 to 80.0	10.0 to 80.0	4.0 to 80.0
Missing	-	-	-
**(Actigraphy) Mean number of steps**			
Mean (SD)	5055.8 (2669.3)	4404.7 (2568.3)	4725.4 (2619.2)

	VR+TAU (N=76)	TAU (N=79)	Total (N=155)
Median (IQR)	4375.3 (3246.4 to 6522.6)	3988.7 (2662.4 to 5326.3)	4163.9 (2889.1 to 5652.0)
Min to Max	592.0 to 12747.0	580.6 to 10301.3	580.6 to 12747.0
Missing	43	45	88
**Time budget score**			
Mean (SD)	55.7 (18.0)	53.5 (18.0)	54.6 (17.9)
Median (IQR)	52.0 (43.0 to 70.0)	54.5 (39.5 to 64.5)	54.0 (42.0 to 67.0)
Min to Max	23.0 to 99.0	11.0 to 95.0	11.0 to 99.0
Missing	13	19	32
**Agoraphobia Mobility Inventory-Avoidance (AMI-A) score**			
Mean (SD)	3.3 (0.8)	3.2 (0.9)	3.2 (0.8)
Median (IQR)	3.3 (2.8 to 3.7)	3.2 (2.4 to 3.8)	3.3 (2.8 to 3.8)
Min to Max	1.1 to 4.7	1.1 to 4.8	1.1 to 4.8
Missing	-	1	1
**O-BAT - maximum number of steps avoided**			
Mean (SD)	-	-	-
Median (IQR)	-	-	-
Min to Max	-	-	-
Missing	76	79	155
**O-BAT Mean distress score**			
Mean (SD)	-	-	-
Median (IQR)	-	-	-
Min to Max	-	-	-
Missing	76	79	155
**R-GPTS-A (social reference) score**			
Mean (SD)	14.4 (9.8)	12.6 (9.6)	13.4 (9.7)
Median (IQR)	14.0 (7.0 to 23.0)	10.5 (4.0 to 20.0)	13.0 (5.0 to 21.0)
Min to Max	0.0 to 32.0	0.0 to 32.0	0.0 to 32.0

	VR+TAU (N=76)	TAU (N=79)	Total (N=155)
Missing	5	5	10
**R-GPTS-B (persecution) score**			
Mean (SD)	18.1 (13.1)	13.1 (13.1)	15.5 (13.3)
Median (IQR)	19.0 (5.0 to 29.0)	8.0 (2.0 to 23.0)	12.0 (3.0 to 26.0)
Min to Max	0.0 to 40.0	0.0 to 40.0	0.0 to 40.0
Missing	5	5	10
**R-GPTS (overall) score**			
Mean (SD)	32.4 (21.7)	25.7 (21.3)	29.0 (21.7)
Median (IQR)	33.0 (15.0 to 52.0)	20.0 (7.0 to 43.0)	25.0 (10.0 to 47.0)
Min to Max	0.0 to 72.0	0.0 to 72.0	0.0 to 72.0
Missing	5	5	10
**Paranoia Worries Questionnaire (PWQ) total score**			
Mean (SD)	9.8 (6.2)	8.4 (6.5)	9.1 (6.3)
Median (IQR)	10.0 (4.0 to 15.0)	9.0 (2.0 to 13.0)	10.0 (3.0 to 14.0)
Min to Max	0.0 to 20.0	0.0 to 20.0	0.0 to 20.0
Missing	6	6	12
**Patient Health Questionnaire (PHQ-9) total score**			
Mean (SD)	15.0 (6.3)	13.7 (6.8)	14.3 (6.6)
Median (IQR)	15.0 (11.0 to 20.0)	14.0 (8.0 to 19.0)	14.0 (9.0 to 20.0)
Min to Max	2.0 to 27.0	2.0 to 27.0	2.0 to 27.0
Missing	5	5	10
**Columbia Suicide Severity Rating Scale (C-SSRS) total score**			
Mean (SD)	0.8 (1.0)	0.7 (1.1)	0.7 (1.1)
Median (IQR)	0.0 (0.0 to 1.0)	0.0 (0.0 to 1.0)	0.0 (0.0 to 1.0)
Min to Max	0.0 to 3.0	0.0 to 4.0	0.0 to 4.0
Missing	12	11	23
**EQ-5D-5L INDEX**			
Mean (SD)	0.5 (0.3)	0.6 (0.3)	0.5 (0.3)

	VR+TAU (N=76)	TAU (N=79)	Total (N=155)
Median (IQR)	0.6 (0.3 to 0.8)	0.6 (0.4 to 0.8)	0.6 (0.3 to 0.8)
Min to Max	-0.2 to 1.0	-0.1 to 1.0	-0.2 to 1.0
Missing	2	1	3
**EQ5D VAS score**			
Mean (SD)	53.9 (19.1)	58.3 (19.4)	56.2 (19.3)
Median (IQR)	50.0 (40.0 to 70.0)	60.0 (45.0 to 75.0)	55.0 (45.0 to 70.0)
Min to Max	0.0 to 85.0	6.0 to 95.0	0.0 to 95.0
Missing	3	2	5
**Recovering Quality Of Life (REQOL-20) total score**			
Mean (SD)	34.0 (14.7)	35.8 (14.0)	34.9 (14.3)
Median (IQR)	33.0 (23.0 to 48.0)	36.0 (27.0 to 47.0)	35.0 (25.0 to 48.0)
Min to Max	3.0 to 63.0	5.0 to 72.0	3.0 to 72.0
Missing	2	4	6
**Progress of Recovery (QPR) total score**			
Mean (SD)	27.8 (11.4)	28.8 (12.6)	28.3 (12.0)
Median (IQR)	27.0 (20.0 to 37.0)	30.0 (23.0 to 37.0)	29.0 (22.0 to 37.0)
Min to Max	4.0 to 51.0	0.0 to 56.0	0.0 to 56.0
Missing	-	-	-

[1] Stratification variables
[2] From baseline CRF & medical record data
[3] Not mutually exclusive

AUTHOR: Ushma Galal

DATE: 12 Feb 2022 20:25:21

SOFTWARE: Stata Version 16.1 (SE)

Table 1. Summary statistics for the mediation and moderation outcomes

	Group TAU (N=172)	Group VR+TAU (N=174)
**Mediation outcomes at each time point, mean (SD) [n]**		
**O-CDBQ Part1**		
Baseline	18.3 (8.7) [170]	20.2 (9.4) [171]
6 weeks	16.2 (9.1) [151]	15.9 (9.6) [149]
26 weeks	15.2 (10.3) [138]	15.4 (10.6) [137]
**O-CDBQ Part3**		
Baseline	14.3 (5.5) [169]	15.1 (5.6) [171]
6 weeks	12.8 (5.9) [150]	12.1 (5.6) [149]
26 weeks	12.6 (7.0) [138]	11.9 (6.7) [137]
**VAS1 - 'I generally feel safe around other people.'**		
Baseline	44.9 (27.0) [160]	44.9 (27.2) [159]
6 weeks	51.8 (28.1) [144]	53.1 (25.3) [142]
26 weeks	52.6 (29.7) [132]	57.1 (26.5) [130]
**VAS2 - 'I feel vulnerable.'**		
Baseline	60.0 (26.4) [160]	62.0 (26.7) [159]
6 weeks	55.4 (28.4) [144]	50.9 (27.0) [142]
26 weeks	51.7 (29.7) [132]	50.1 (29.9) [130]
**VAS3 - 'When I go out, something bad will happen.'**		
Baseline	59.6 (28.7) [160]	62.0 (28.1) [159]
6 weeks	51.7 (30.0) [144]	48.9 (27.0) [142]
26 weeks	48.6 (33.1) [132]	47.1 (28.9) [130]
**Moderation outcomes at baseline, mean (SD) [n]**		

	Group TAU<br><br>(N=172)	Group VR+TAU<br><br>(N=174)
**Voices questionnaire**	7.9 (7.1) [158]	9.6 (7.5) [158]
**Beck Hopelessness Scale**	9.3 (5.5) [155]	8.3 (6.0) [157]
**Body-esteem Scale for Adolescents and Adults**	32.1 (15.3) [152]	29.5 (16.1) [154]

Table 2. Summary statistics and results for the additional post-hoc subgroup analysis on O-AS Avoidance & Distress

	TAU (N=172)	VR+TAU (N=174)	Interaction effect [95% CI][1]	Test of Interaction (P value)‡
**O-AS Avoidance at 6 weeks**				
**Age (years), mean (SD) [n]**	160	160	-0.03 [-0.06 to 0.01]	0.111
**Sex, mean (SD) [n]**				
Female	3.1 (2.7) [50]	2.0 (2.2) [57]	-0.73 [-1.45 to -0.00]	0.262
Male	2.2 (2.6) [106]	1.9 (2.2) [103]	-0.22 [-0.73 to 0.29]	
**O-AS Distress at 6 weeks**				
**Age (years), mean (SD) [n]**	162	160	-0.12 [-0.39 to 0.14]	0.352
**Sex, mean (SD) [n]**				
Female	47.1 (21.9) [50]	38.8 (18.7) [57]	-5.79 [-11.56 to -0.03]	0.429
Male	44.8 (19.8) [108]	42.6 (18.8) [103]	-2.95 [-7.03 to 1.13]	

[1] VR+TAU vs. TAU: Linear regression model for the primary outcome; modelled against treatment group, outcome score at baseline, stratification factors (site and service type) and an interaction between randomised group and the subgroup variable.

Pictures of the gameChange VR therapy

1. The virtual coach in the virtual office

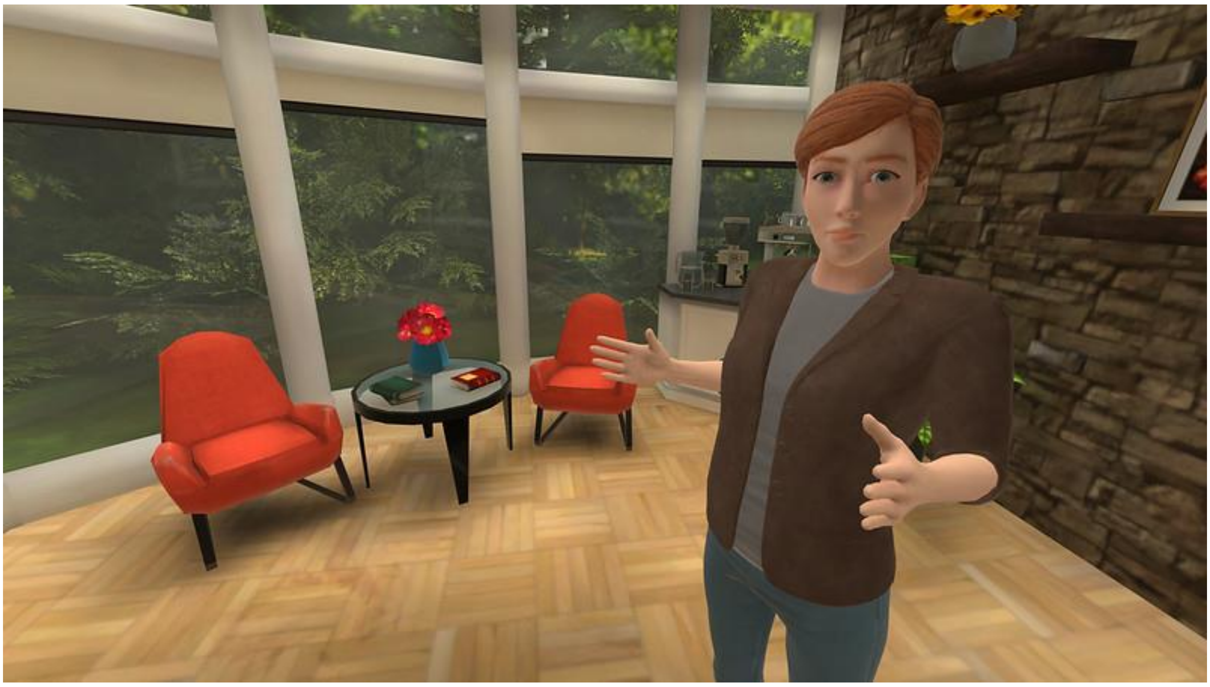

2. Leaving the virtual front door onto the street scene

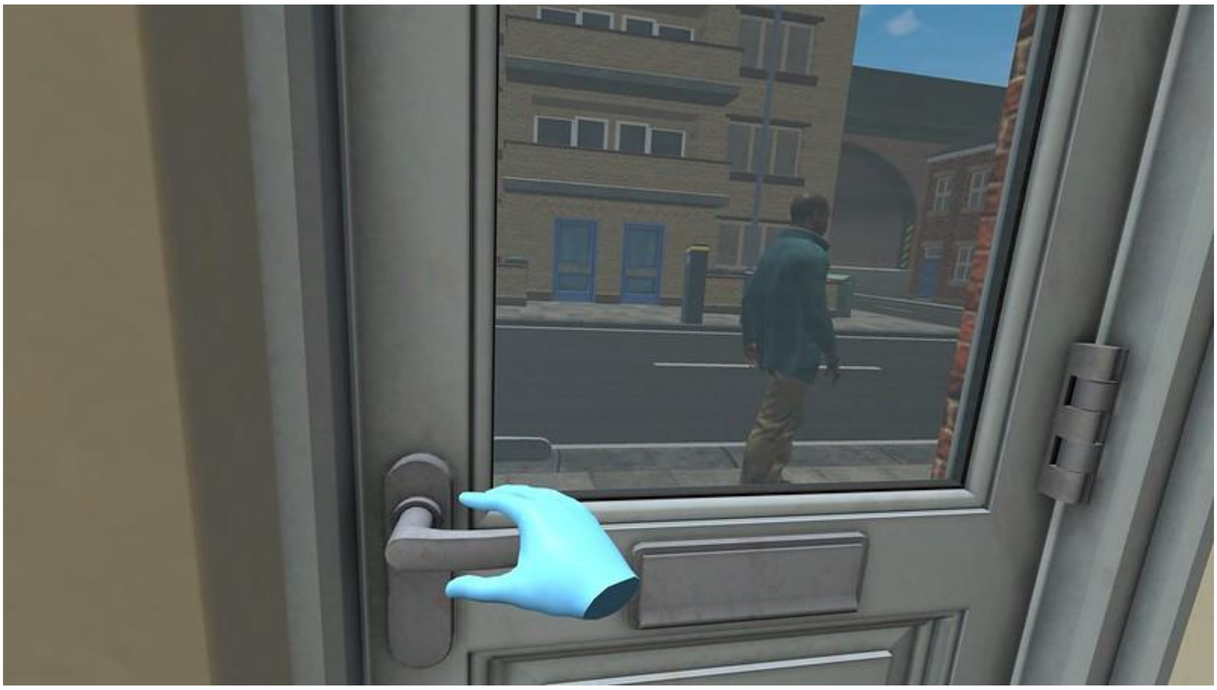

3. Getting onto the virtual bus

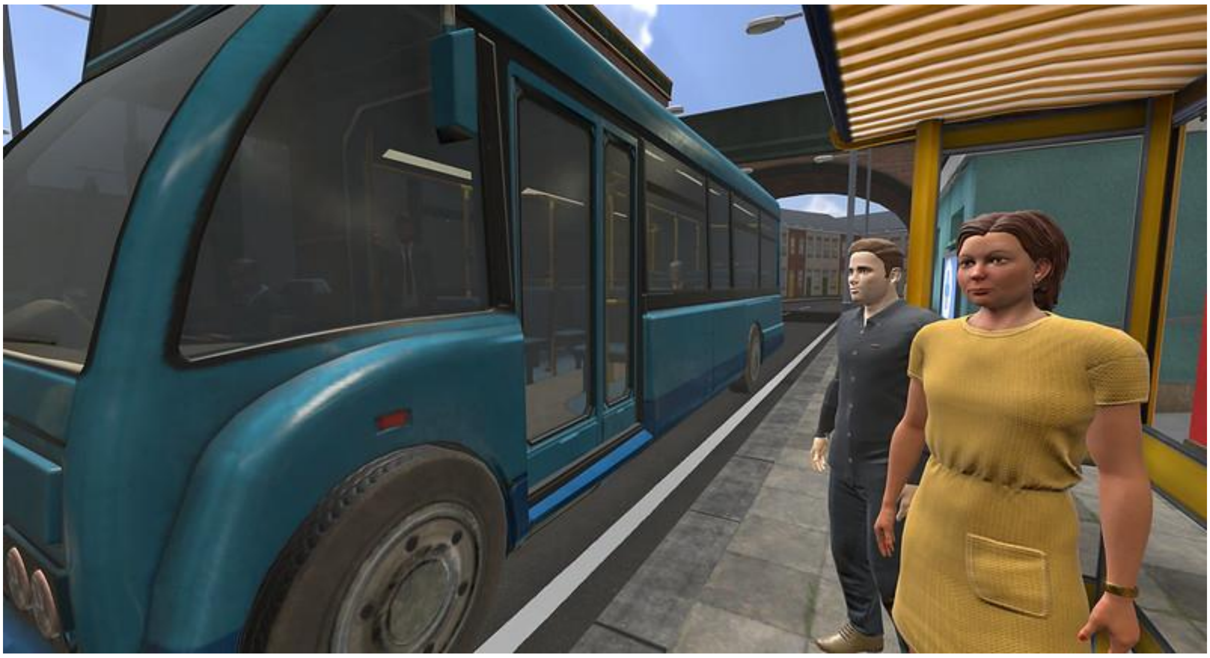

4. The virtual shop

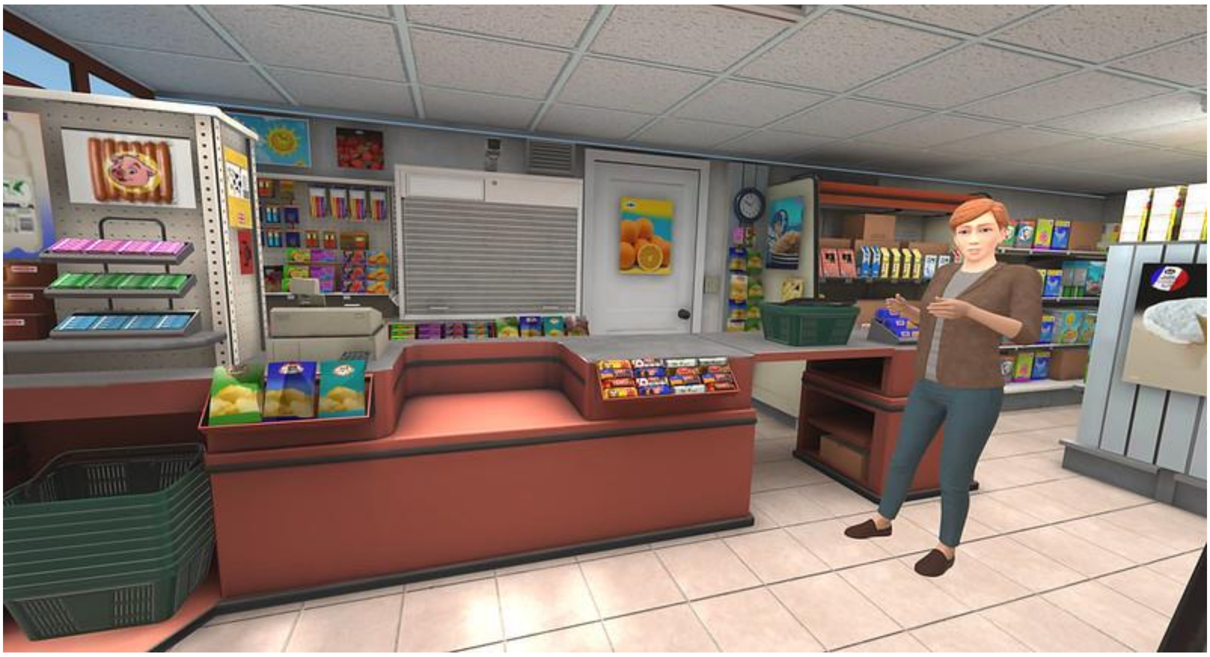

5. The virtual pub

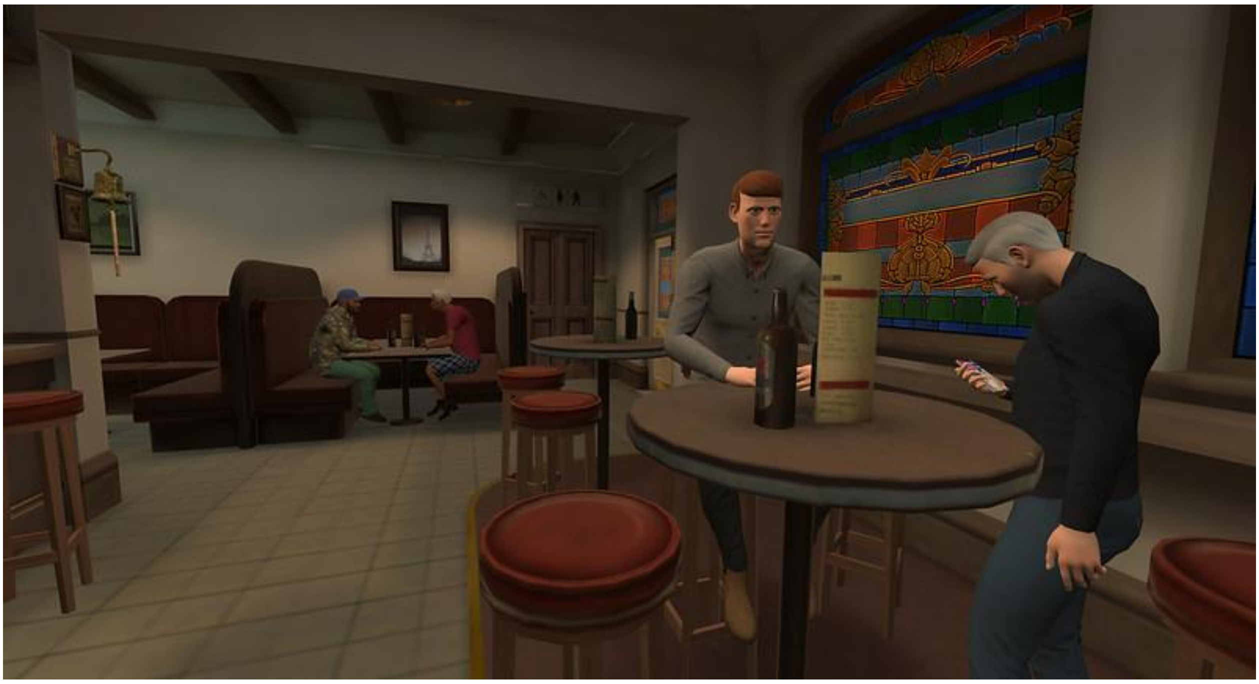

6. The virtual doctor’s surgery

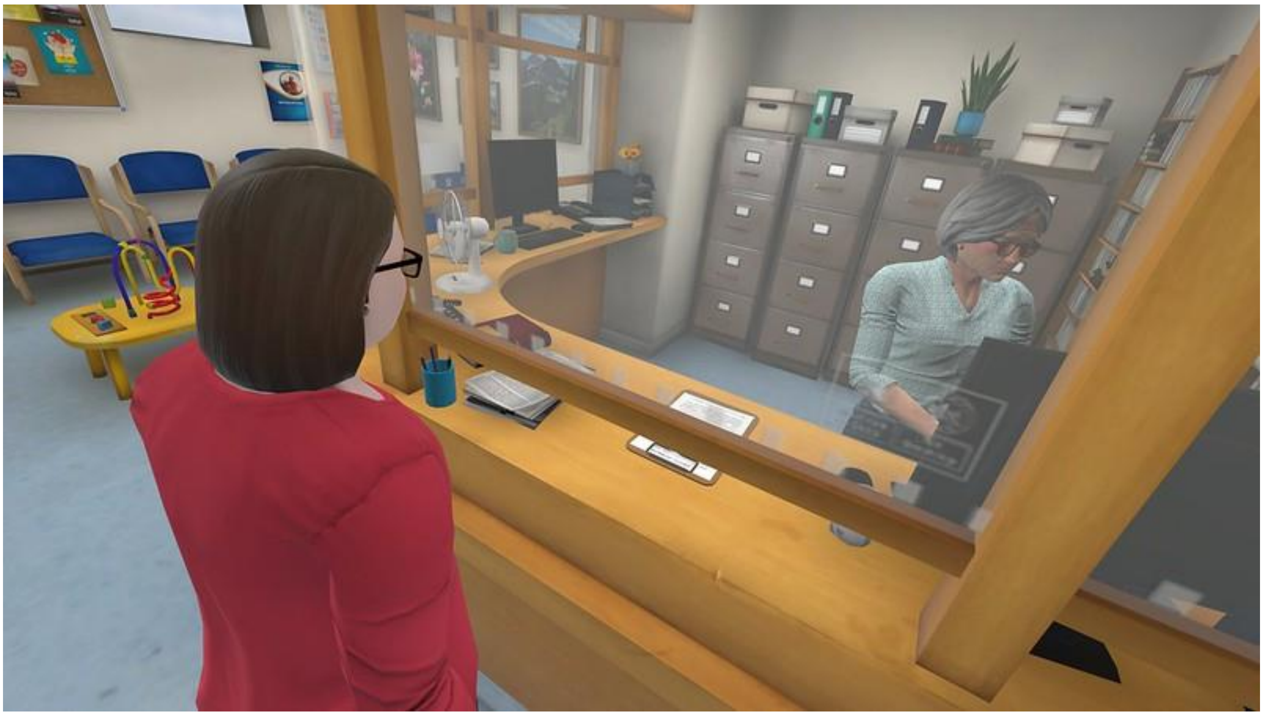

7. The virtual café

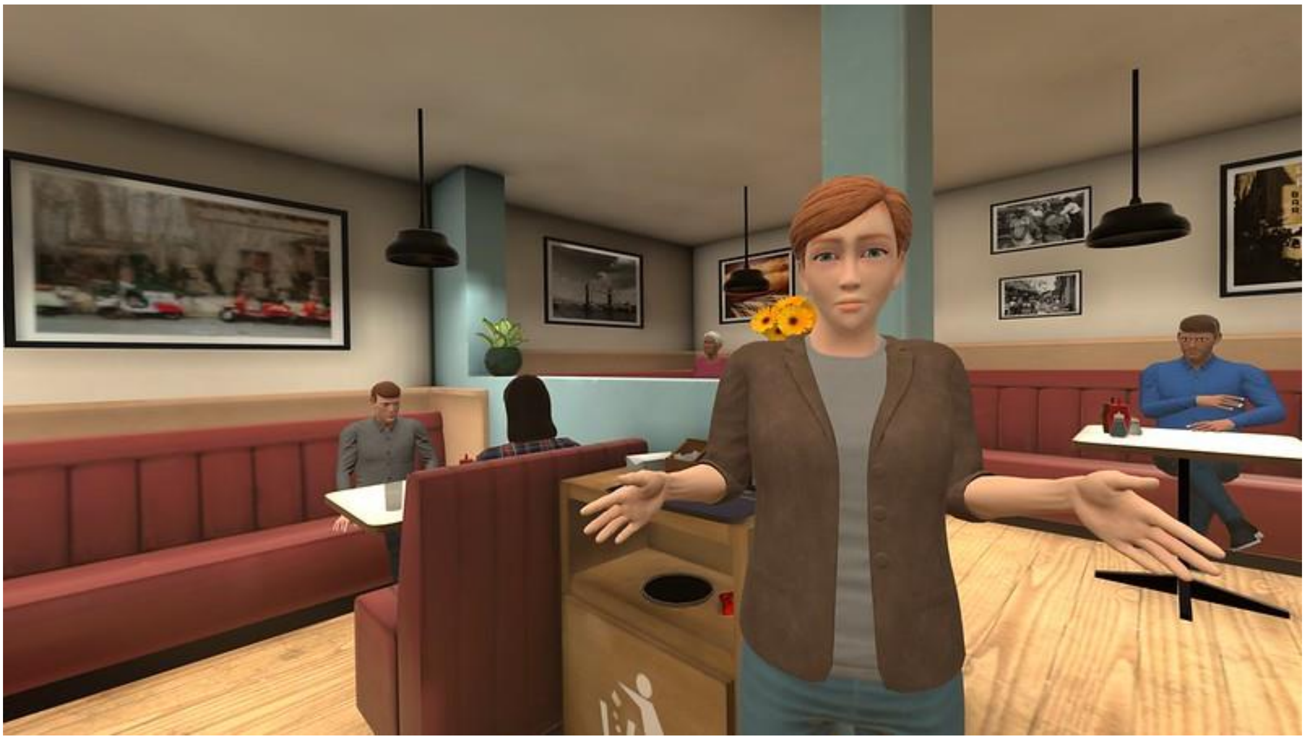

Supplement: Supplementary appendix [file mmc1.pdf]
